# Benzo-thia-fused [*n*]thienoacenequinodimethanes with small to moderate diradical characters: the role of pro-aromaticity *versus* anti-aromaticity†
†Electronic supplementary information (ESI) available: Synthetic procedures and characterization data for all new compounds, general experimental method, additional spectroscopic data, DFT calculation details, crystallographic data. CCDC 1419444–1419447. For ESI and crystallographic data in CIF or other electronic format see DOI: 10.1039/c5sc04706d


**DOI:** 10.1039/c5sc04706d

**Published:** 2016-01-19

**Authors:** Xueliang Shi, Estefanía Quintero, Sangsu Lee, Linzhi Jing, Tun Seng Herng, Bin Zheng, Kuo-Wei Huang, Juan T. López Navarrete, Jun Ding, Dongho Kim, Juan Casado, Chunyan Chi

**Affiliations:** a Department of Chemistry , National University of Singapore , 3 Science Drive 3 , 117543 , Singapore . Email: chmcc@nus.edu.sg; b Department of Physical Chemistry , University of Malaga , Campus de Teatinos s/n , 229071 Malaga , Spain . Email: casado@uma.es; c Department of Chemistry , Yonsei University , Seoul 120-749 , Korea . Email: dongho@yonsei.ac.kr; d Department of Materials Science & Engineering , National University of Singapore , 119260 , Singapore . Email: msedingj@nus.edu.sg; e Division of Physical Science and Engineering and KAUST Catalysis Center , King Abdullah University of Science and Technology (KAUST) , Thuwal 23955-6900 , Kingdom of Saudi Arabia

## Abstract

Open-shell singlet diradicaloids have recently received much attention due to their unique optical, electronic and magnetic properties and promising applications in materials science. Among various diradicaloids, quinoidal π-conjugated molecules have become the prevailing design. However, the need for a fundamental understanding on how the fusion mode and pro-aromaticity/anti-aromaticity affect their diradical character and physical properties remains unaddressed. In this work, a series of pro-aromatic benzo-thia-fused [*n*]thienoacenequinodimethanes (**Th*n*-TIPS** (*n* = 1–3) and **BDTh-TIPS**) were synthesized and compared with the previously reported anti-aromatic bisindeno-[*n*]thienoacenes (**S*n*-TIPS**, *n* = 1–4). The ground-state geometric and electronic structures of these new quinoidal molecules were systematically investigated by X-ray crystallographic analysis, variable temperature NMR, ESR, SQUID, Raman, and electronic absorption spectroscopy, assisted by DFT calculations. It was found that the diradical character index (*y*_0_) increased from nearly zero for **Th1-TIPS** to 2.4% for **Th2-TIPS**, 18.2% for **Th3-TIPS**, and 38.2% for **BDTh-TIPS**, due to the enhanced aromatic stabilization. Consequently, with the extension of molecular size, the one-photon absorption spectra are gradually red-shifted, the two-photon absorption (TPA) cross section values increase, and the singlet excited state lifetimes decrease. By comparison with the corresponding anti-aromatic analogues **S*n*-TIPS** (*n* = 1–3), the pro-aromatic **Th*n*-TIPS** (*n* = 1–3) exhibit larger diradical character, longer singlet excited state lifetime and larger TPA cross section value. At the same time, they display distinctively different electronic absorption spectra and improved electrochemical amphotericity. Spectroelectrochemical studies revealed a good linear relationship between the optical energy gaps and the molecular length in the neutral, radical cationic and dicationic forms. Our research work discloses a significant difference between the pro-aromatic and anti-aromatic quinoidal compounds and provides guidance for the design of new diradicaloids with desirable properties.

## Abstract 2

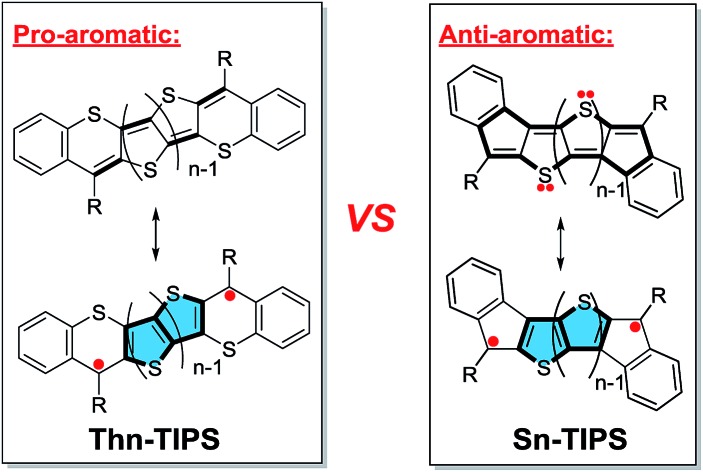
Pro-aromatic **Th*n*-TIPS** exhibits distinctly different physical properties from anti-aromatic **S*n*-TIPS**.

## Introduction

Quinoidal π-conjugated compounds exhibit distinctively different features from those of aromatic molecules, for example, low band gap, redox amphotericity, and open-shell diradical character, which render them useful for application in organic near-infrared (NIR) dyes, *n*-channel or ambipolar field effect transistors (FETs), non-linear optics and spin-based electronics.^1^ In particular, their natural tendency to recover the aromaticity of the non-aromatic quinoidal units could lead to an open-shell singlet diradical ground state where the singlet diradical solution has lower energy than the closed-shell singlet. In recent years, various open-shell singlet diradicaloids based on quinoidal π-conjugated molecules have been developed, including quinoidal oligothiophenes,^2^ quinoidal thienoacenes,^3^ and many quinoidal polycyclic hydrocarbons such as bisphenalenyls,^4^ zethrenes,^5^ indenofluorenes,^6^ and extended *para*-quinodimethane (*p*-QDM) oligomers.^7^ The typical designs include: (1) construction of oligomers of quinoidal benzene or thiophene; and (2) incorporation of a quinodimethane unit into an aromatic π-framework. To ensure stability, kinetic blocking of the most reactive sites is usually necessary. Previous studies also revealed that several factors such as aromatic stabilization and steric strain release could significantly affect the singlet diradical character and consequently their physical properties and chemical reactivity. Generally, larger aromatic stabilization energy and lower steric strain in the diradical form favor an open-shell singlet diradical ground electronic state. These studies also demonstrated that pro-aromatic and anti-aromatic π-conjugated molecules have an irresistible tendency to be diradicals. “Anti-aromaticity” is a characteristic of compounds that contain 4*n* π-electrons in a cyclic planar, or nearly planar, system of alternating single and double bonds. A “pro-aromatic” compound refers to a non-aromatic molecule containing a quinodimethane like structure which has high tendency to become an open-shell diradical and/or zwitterionic form containing one or more aromatic sextets. The diradical character index (*y*_0_) and singlet–triplet energy gap (Δ*E*_S–T_) are two important parameters that can be correlated to third-order non-linear optical (NLO) properties and magnetic activity of open-shell singlet diradicaloids.^8^ However, a fundamental understanding of how the fusion mode and the pro-aromaticity/anti-aromaticity affect their ground-state electronic structures and consequently their physical properties has not yet been attained.

**Fig. 1:**
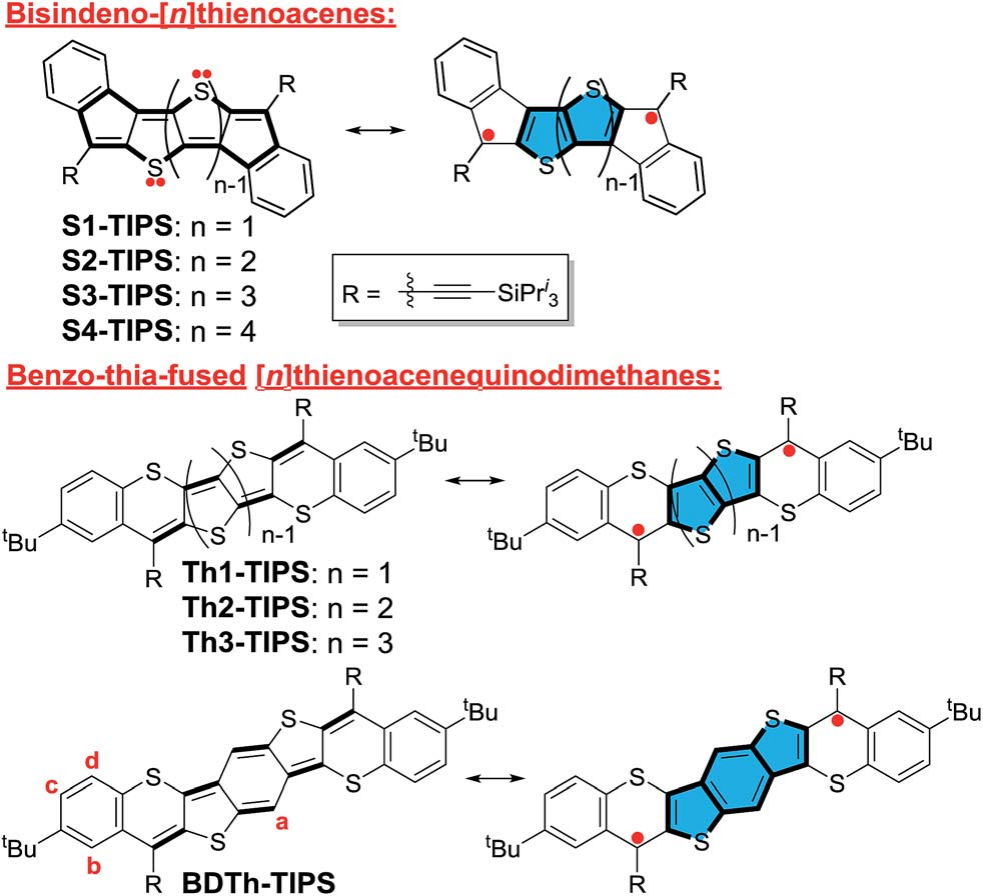
Structures of anti-aromatic bisindeno-[*n*]thienoacenes and pro-aromatic quinoidal benzo-thia-fused [*n*]thienoacenequinodimethanes.

Recently, our group and Haley's group independently reported the synthesis and properties of a series of quinoidal bisindeno-[*n*]thienoacenes (**S*n*-TIPS**) (*n* = 1–4) (Fig. 1), which are better regarded as an anti-aromatic system because the NICS(1)_zz_ values of the cyclopenta- and the central thiophene rings are significantly positive.^9^ It turns out that the monomer to trimer (*n* = 1–3) all have negligible diradical character and only the tetramer has a significant diradical character (*y*_0_ = 20.2% at the UCAM-B3LYP/6-31G* level of theory). Our attention was then switched to their analogues, the benzo-thia-fused [*n*]thienoacenequinodimethanes (**Th*n*-TIPS** (*n* = 1–3) and **BDTh-TIPS**), in which the diradical resonance form is supposed to contribute significantly to the ground-state structure due to the recovery of aromaticity of the central quinoidal thienoacene unit (Fig. 1). In principle, a monocycle containing 4*n* π electrons can be also drawn for these molecules, implying a potential anti-aromatic character. However, our calculations indicate that the NICS(1)_zz_ values of the central thiophene/benzene rings are significantly negative (*vide infra*), and thus they are better regarded as a pro-aromatic system with small anti-aromaticity. Comparison between these two systems will provide us with further knowledge about the influence of aromaticity or anti-aromaticity in the electronic and physical properties. Therefore, in this work, we will present systematic studies on: (1) the efficient synthesis of these pro-aromatic quinoidal molecules; (2) their ground-state geometric and electronic structures probed by various experimental methods and theoretical calculations; (3) their optical, electrochemical and magnetic properties; (4) a detailed comparison with the anti-aromatic compounds, **S*n*-TIPS**; and (5) the relationships between the pro-aromaticity/anti-aromaticity, diradical character and physical properties. Our intention is to provide better insight into the design of quinoidal diradicaloids with desirable properties.

## Results and discussion

### Synthesis

**Scheme 1:**
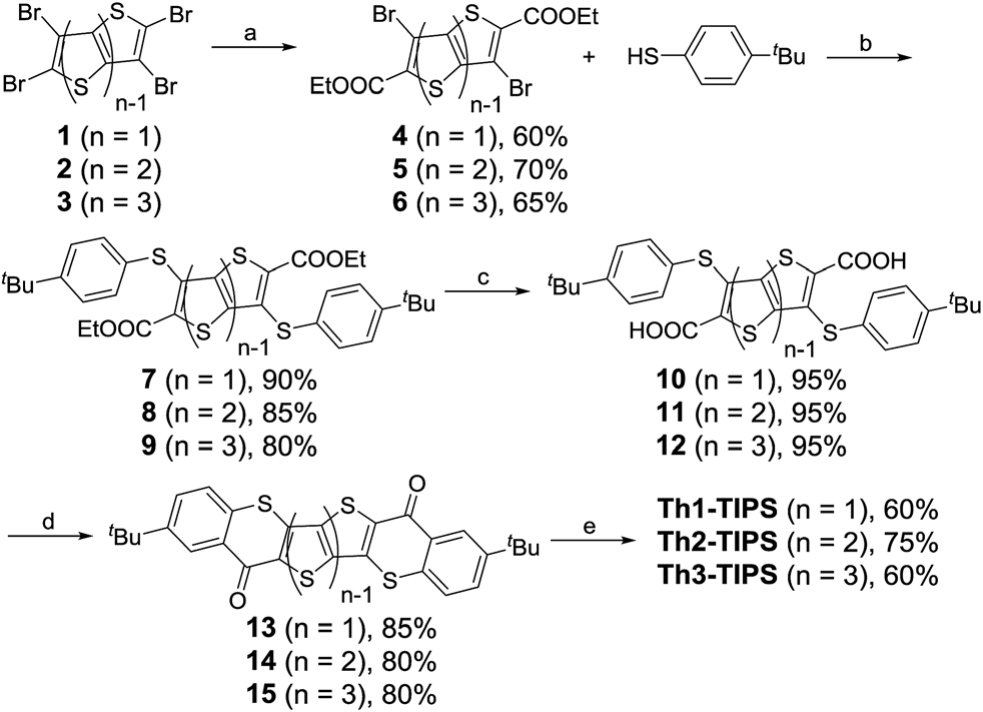
Synthetic route to **Th*n*-TIPS**. Reagents and conditions: (a) (i) *n*-BuLi, dry THF, –78 °C; (ii) NCCOOEt, –78 °C – r.t., overnight; (iii) H_2_O, 0 °C; (b) Pd_2_(dba)_3_, dppf, DMF, ^i^Pr_2_NEt, 100 °C, overnight; (c) (i) NaOH, EtOH, reflux overnight; (ii) 10% HCl (aq.); (d) (i) SOCl_2_, DMF, DCM, reflux; (ii) AlCl_3_, dry DCM, 0 °C – r.t., overnight; (e) (i) TIPSCCLi, dry THF, 0 °C – r.t.; (ii) SnCl_2_, 6 h.

**Scheme 2:**
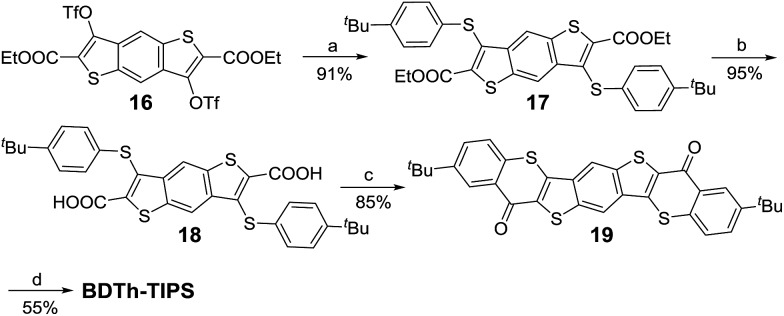
Synthetic route to **BDTh-TIPS**. Reagents and conditions: (a) 4-*tert*-butylbenzenethiol, K_2_CO_3_, DMF, r.t., 12 h; (b) (i) NaOH, EtOH, reflux overnight; (ii) 10% HCl (aq.); (c) (i) SOCl_2_, DMF, dry DCM, reflux; (ii) AlCl_3_, dry dichloromethane, 0 °C – r.t., overnight; (d) (i) TIPSCCLi, THF, 0 °C – r.t.; (ii) SnCl_2_, 6 h.

The synthetic strategy towards these quinoidal compounds was based on the synthesis of the corresponding diketones by intramolecular Friedel–Crafts acylation reaction, followed by nucleophilic addition with triisopropylsilylethynyl lithium (Li-TIPSE) and then by reduction of the intermediate diols with SnCl_2_ (Schemes 1 and 2).

The key intermediates for the synthesis of **Th*n*-TIPS** (*n* = 1–3) are the dibromo-diesters **4–6**, which were first synthesized in 60–70% yield by regio-selective lithiation of the corresponding tetrabromides **1–3** followed by quenching with ethyl cyanoformate in a one-pot protocol (Scheme 1).3b,^10^ Compounds **4–6** underwent palladium-catalyzed cross-coupling reaction^11^ with 4-*tert*-butylbenzenethiol to give compounds **7–9** in 80–90% yield. Compounds **7–9** were then hydrolyzed and acidified to form diacids **10–12** in 95% yield and the diacids were converted into the corresponding carboxylic acid chlorides by reaction with thionyl chloride in dry dichloromethane (DCM). Subsequent double Friedel–Crafts acylation with aluminium chloride afforded the desired diketones **13–15** in 80–85% yield. Compound **Th*n*-TIPS** (*n* = 1–3) was then obtained in an overall 60–75% yield by addition of Li-TIPSE to the corresponding diketones **13–15** followed by reductive dehydroxylation with SnCl_2_.

The synthetic route to **BDTh-TIPS** shown in Scheme 2 is slightly different from that to **Th*n*-TIPS** (*n* = 1–3). Instead of dibromo-diesters **4–6**, the ditriflate-diester compound **16**^12^ was used to carry out a direct nucleophilic substitution reaction with 4-*tert*-butylbenzenethiol under basic conditions to give compound **17** in 91% yield as the triflate is a good leaving group. Subsequent reactions following a similar protocol to that shown in Scheme 1 gave the target compound **BDTh-TIPS** in an acceptable yield. Compounds **Th*n*-TIPS** (*n* = 1–3) and **BDTh-TIPS** showed good stability both in solution and in the solid state, and their structures were unambiguously confirmed by X-ray crystallographic analysis (*vide infra*).

### Electronic absorption spectra

**Fig. 2:**
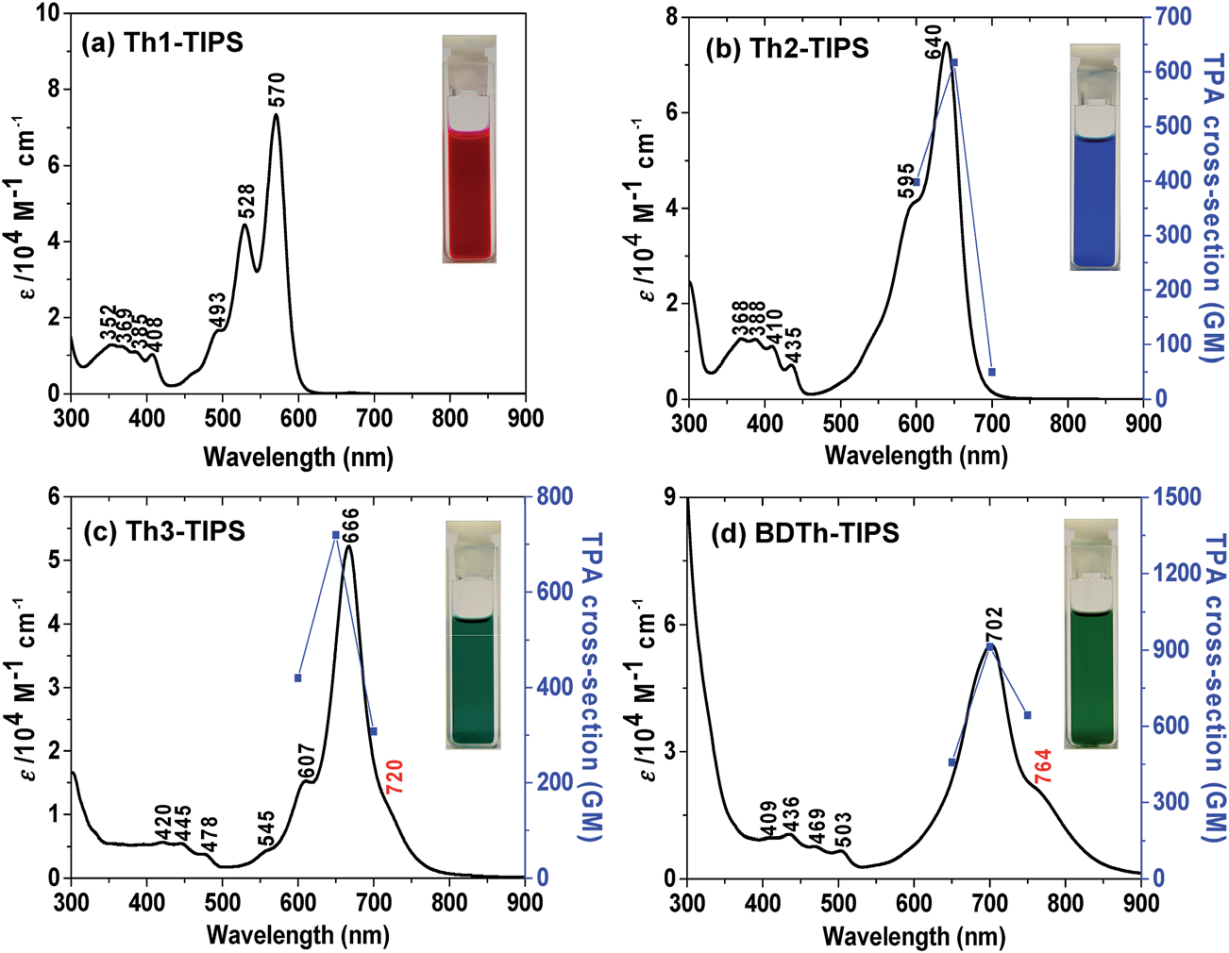
One-photon absorption spectra (solid lines and left vertical axes) in DCM and two-photon absorption spectra in toluene (blue symbols and right vertical axes) of (a) **Th1-TIPS**, (b) **Th2-TIPS**, (c) **Th3-TIPS** and (d) **BDTh-TIPS**. The TPA spectrum of **Th1-TIPS** was not recorded due to the limit of measurement range. TPA spectra are plotted at λ_ex_/2. Inserted are the photos of the solutions.

Recent studies demonstrated that electronic absorption spectra give an initial insight to identify an open-shell singlet diradical ground electronic state based on the presence of a weak low-energy absorption band in the NIR region.^13^ The UV-vis-NIR absorption spectra of all compounds are shown in Fig. 2 and the data are collected in Table 1. **Th1-TIPS** and **Th2-TIPS** show an intense band with maxima at 570 nm and 640 nm, respectively, which is very similar to the p-band of many closed-shell polycyclic aromatic hydrocarbons such as acenes and rylenes^14^ and closed-shell quinoidal compounds such as heptazethrene.5*e* The structure of this band is dramatically different from that of their counterparts **S1-TIPS** and **S2-TIPS**, in which a broad absorption band was observed in this region attributed to their anti-aromatic character.9*b* The main absorption peak red-shifts to 666 nm in **Th3-TIPS** and 702 nm in **BDTh-TIPS**, and the spectra again are very different from their anti-aromatic analogue **S3-TIPS**.

**Table 1:** Summary of the photophysical and electrochemical data^*a*^

Comp	*λ*_abs_ (nm)	*ε* (10^4^ M^–1^ cm^–1^)	*E* ox 1/2 (V)	*E* red 1/2 (V)	HOMO (eV)	LUMO (eV)	*E* EC g (eV)	*E* opt g (eV)	*τ* (ps)	*σ* ^(2)^_max_ (GM) (*λ*_ex_)
**Th1-TIPS**	570	7.32	0.05	–1.83	–4.77	–3.06	1.71	2.05	3 (*τ*_1_)	—
			0.55	–2.11					5800 (*τ*_2_)	
**Th2-TIPS**	640	7.46	–0.13	–1.71	–4.56	–3.17	1.39	1.90	1.2 (*τ*_1_)	620 (1300 nm)
			0.32	–1.90					2800 (*τ*_2_)	
**Th3-TIPS**	666	5.23	–0.25	–1.59	–4.42	–3.35	1.07	1.65	20 (*τ*_1_)	720 (1300 nm)
			0.14	–1.80					1700 (*τ*_2_)	
**BDTh-TIPS**	702	5.52	–0.26	–0.83	–4.39	–3.40	0.99	1.46	40 (*τ*_1_)	910 (1400 nm)
			0.07	–1.73					820 (*τ*_2_)	

^*a*^ *λ*_abs_: absorption maxima. *ε*: molar extinction coefficient for the corresponding absorption maximum. *E*ox1/2 and *E*red1/2: half-wave potentials of the oxidative and reductive waves, respectively, with potentials *vs.* Fc/Fc^+^ couple. HOMO = –(4.8 + *E*onsetox) and LUMO = –(4.8 + *E*onsetred), where *E*onsetox and *E*onsetred are the onset potentials of the first oxidative and reductive waves, respectively. *E*ECg: electrochemical band gap. *E*optg: optical band gap estimated from the absorption onset. *τ*: singlet excited lifetime obtained from TA. *σ*_max_^(2)^: maximum TPA cross section. *λ*_ex_: excitation wavelength in TPA measurements.

The most distinctive feature, however, is that the spectra of **Th3-TIPS** and **BDTh-TIPS** become broadened, and weak low-energy absorptions, as shoulders of the main bands, appear at 720 and 764 nm, respectively. This is strong evidence of the change of the ground state of this series of molecules with extension of the molecular size. This change was also observed in the zethrene series^5^ and the lowest energy absorption shoulder originates from the presence of a low-lying singlet excited state dominated by a doubly excited electronic configuration (H,H → L,L).^13^ This observation indicates an increase of diradical character with the increase of the molecular length which can be explained by the recovery of more aromatic thiophene rings in the diradical form. The more broadened and red-shifted spectrum observed in **BDTh-TIPS** compared with its analogue **Th3-TIPS** also implies a larger diradical character, which can be accounted for by the more aromatic character of the benzene ring compared to the thiophene ring. In addition, only **Th1-TIPS** exhibits a modest fluorescence signal at 593 nm (Fig. S1 in ESI†).

### Magnetic measurements

**Fig. 3:**
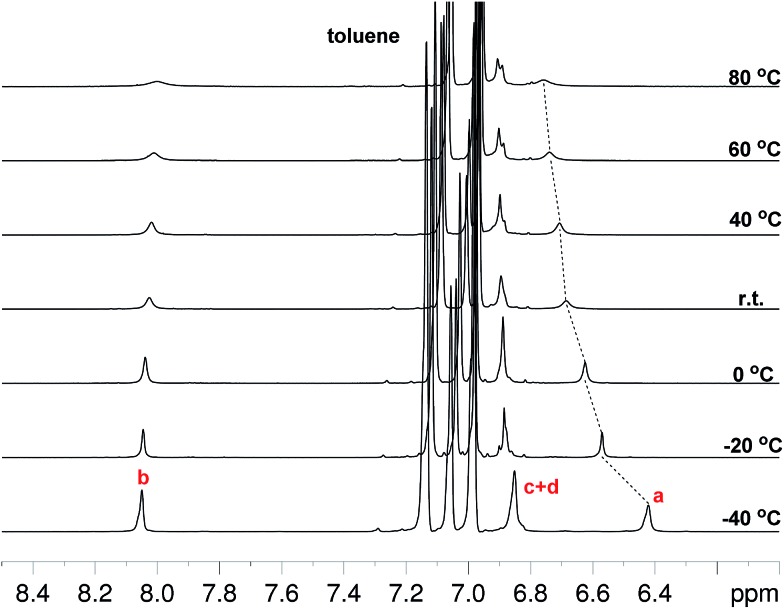
VT ^1^H NMR spectra (aromatic region) of **BDTh-TIPS** in toluene-*d*_8_ and assignment of all aromatic protons. The resonance assignment referred to the structure displayed in Fig. 1.

Compounds **Th*n*-TIPS** (*n* = 1–3) all show well-resolved ^1^H NMR spectra in solution even at elevated temperatures. However, significant signal broadening was observed for **BDTh-TIPS** in toluene-*d*_8_ at room temperature and this became more obvious upon heating, while cooling of the solution resulted in signal sharpening (Fig. 3). This is a typical phenomenon for many open-shell singlet diradicaloids and the NMR signal broadening results from a thermally excited triplet species which is usually slightly higher in energy than the singlet diradical form. The gradual low-field shift of the resonance for protons in the central benzene ring (protons a in Fig. 1) with the increase of temperature also indicates a transformation to a larger aromatic character at higher temperatures.

**Fig. 4:**
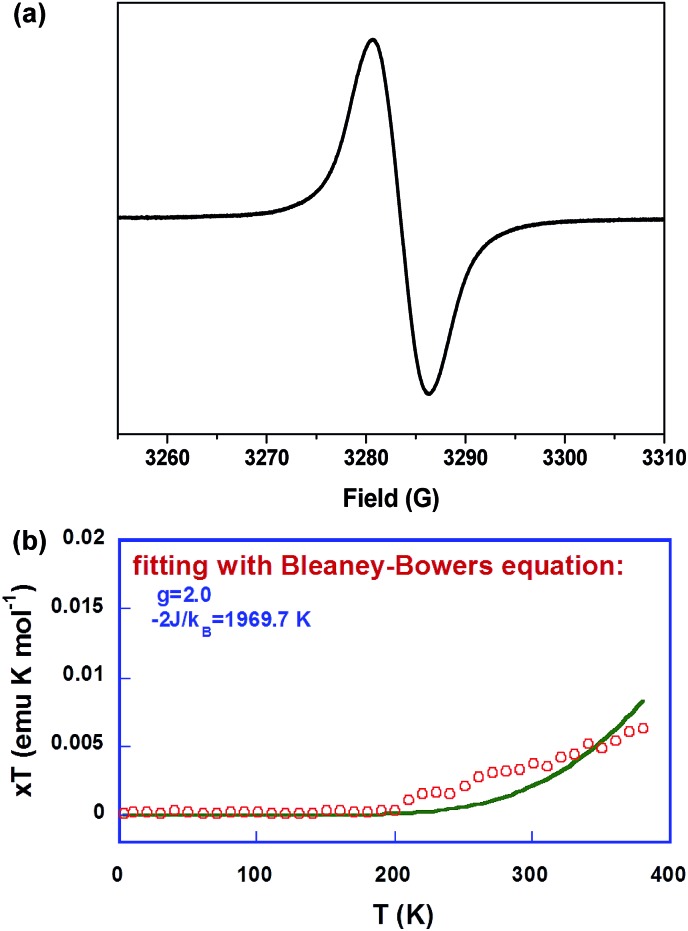
(a) ESR spectrum of **BDTh-TIPS** in toluene solution measured at room temperature. (b) *χT*–*T* plot for the solid **BDTh-TIPS**. The measured data was plotted as open circles, and the fitting curve was drawn using the Bleaney–Bowers equation with *g* = 2.00.

In accordance with the NMR measurements, solutions of **Th*n*-TIPS** (*n* = 1–3) all exhibited no ESR signal, which can be correlated to either a closed-shell singlet ground state or an open-shell singlet diradical ground state but with a large singlet-triplet energy gap. However, **BDTh-TIPS** in toluene displayed a broad single-line ESR spectrum with *g*_e_ = 2.0038 and no half-field spectrum (Δ*m*_s_ = ±2 transition) could be detected due to the large spin delocalization (*vide infra*), and a similar phenomenon was observed for other delocalized singlet diradicaloids (Fig. 4a). Temperature-dependent magnetic susceptibility data on the powder of **BDTh-TIPS** at 5–380 K were collected using a superconducting quantum interference device (SQUID) magnetometer. Fig. 4b plots *χT* as a function of *T*. An increasing susceptibility above 200 K was observed for the powder **BDTh-TIPS**, and fitting the data by using the Bleaney–Bowers equation^15^ gave an approximate singlet–triplet energy gap (Δ*E*_S–T_ or 2*J*/*k*_B_) of –1969.7 K (–0.17 eV or –3.91 kcal mol^–1^). Therefore, **BDTh-TIPS** has a singlet open-shell ground state, and at room temperature its first triplet excited state can be populated because of the small singlet–triplet energy gap, which explains the ESR signal.

### X-ray crystallographic analysis

**Fig. 5:**
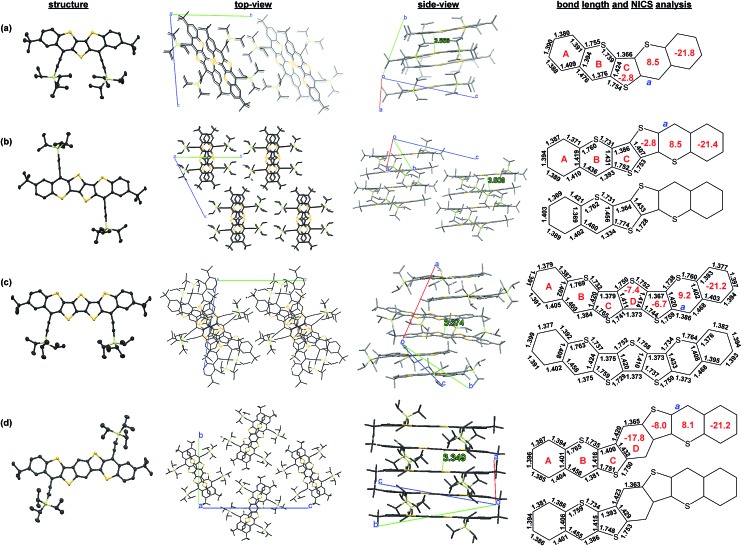
ORTEP drawings, 3D packing structures (both top-view and side-view), and selected bond lengths of (a) **Th1-TIPS**, (b) **Th2-TIPS**, (c) **Th3-TIPS** and (d) **BDTh-TIPS**. Hydrogen atoms are omitted for clearance. The red numbers in the rings denote the calculated NICS(1)_zz_ values.

Single crystals suitable for X-ray crystallographic analysis were obtained for **Th*n*-TIPS** (*n* = 1–3) and **BDTh-TIPS** by slow diffusion of acetonitrile or methanol into their solutions.^16^ The Oak Ridge Thermal Ellipsoid Plot (ORTEP) drawings and 3D packing structures are shown in Fig. 5. The π-conjugated frameworks of all molecules are nearly planar. All three **Th*n*-TIPS** (*n* = 1–3) molecules crystallize in a triclinic lattice system, with space group *P*1[combining macron], whereas **BDTh-TIPS** crystallizes in a monoclinic lattice system, with space group *P*2(1)/*n*. For **Th1-TIPS**, two molecules form an anti-parallel packed dimer *via* π–π interactions with a distance about 3.57 Å. The average distance between adjacent dimers is more than 4.2 Å hence no close π-stacking was observed between each dimer. For **Th2-TIPS**, two molecules also form an anti-parallel packed dimer *via* π–π interaction (distance: 3.51 Å), which further arranges into a columnar structure with an inter-dimer distance of 3.59 Å. Similarly, two of the **Th3-TIPS** molecules also form a dimer *via* π–π interaction (distance: 3.37 Å) and the dimers are further packed into columnar structure *via* multiple close π–π interactions but the neighbouring dimers are rotated for about 70° to suppress steric hindrance. **BDTh-TIPS** shows a π-stacked columnar structure with a close π–π distance of about 3.35 Å. The observed close packing in **Th2-TIPS**, **Th3-TIPS** and **BDTh-TIPS** indicates that they could serve as good semiconductors in FETs in future studies.

Bond length analysis was performed to better analyze the ground-state geometry. Although these molecules are in central *C*_2v_ plane symmetry, two sets of molecules with different bond lengths are observed for **Th2-TIPS**, **Th3-TIPS** and **BDTh-TIPS** in the crystalline form due to the packing effect (Fig. 5). In all cases, large bond length alternation was observed for the central thienoacenequinodimethane unit, indicating a dominant quinoidal structure. The *exo*-methylene bond has a length of 1.376 Å, 1.393/1.334 Å, 1.384/1.386/1.373/1.375 Å and 1.381/1.386 Å for **Th1-TIPS**, **Th2-TIPS**, **Th3-TIPS** and **BDTh-TIPS**, respectively. DFT calculations (*vide infra*) predict the respective length as 1.363, 1.374, 1.389, 1.399 Å, implying an increase of diradical character with chain length, which is in accordance with the above electronic absorption spectroscopic and NMR/ESR/SQUID studies. The outermost two benzene rings in all four molecules display small bond length alternation, indicating their large aromatic character and minor impact onto the electronic properties of this series of molecules.

### DFT calculations

**Fig. 6:**
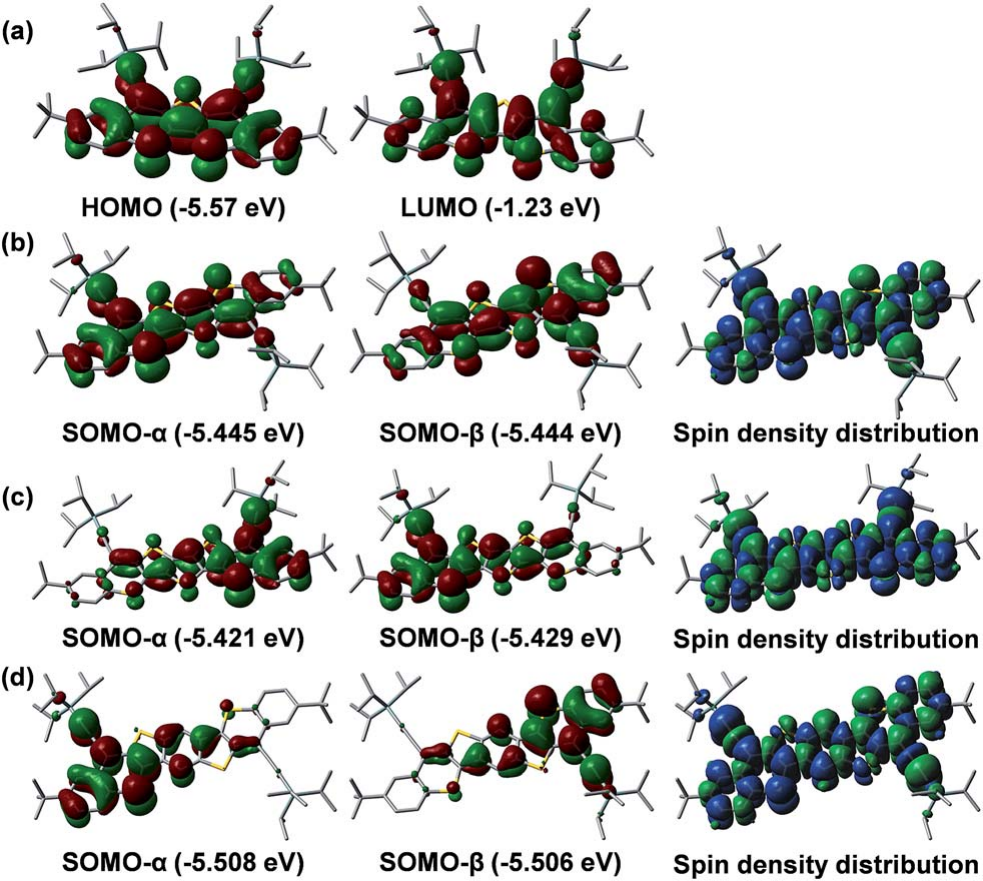
Calculated frontier MO profiles of **Th1-TIPS** (a) and SOMO profiles and spin density distribution of the singlet diradical of **Th2-TIPS** (b), **Th3-TIPS** (c) and **BDTh-TIPS** (d).

Broken symmetry DFT calculations (UCAM-B3LYP/6-31G*) were conducted to understand the ground-state electronic structures. The results showed that the shortest molecule **Th1-TIPS** indeed favors a closed-shell ground state. However, for **Th2-TIPS**, **Th3-TIPS** and **BDTh-TIPS**, the energies of their open-shell singlet diradical states are 9.27/1.36, 5.14/3.56 and 3.38/8.26 kcal mol^–1^ lower than the triplet biradical/closed-shell state, respectively, thus defining their singlet diradical ground electronic states. The HOMO and LUMO wave-functions of **Th1-TIPS** are delocalized along the whole π-conjugated framework (Fig. 6a). The highest SOMO-α and SOMO-β profiles showed an increasing disjoint character from **Th2-TIPS** to **Th3-TIPS** and then to **BDTh-TIPS**, with the spins evenly distributed along the whole π-conjugated framework (Fig. 6b–d). The singlet diradical character index (*y*_0_) values were calculated as 0.024, 0.182 and 0.382 for **Th2-TIPS**, **Th3-TIPS** and **BDTh-TIPS**, respectively, while negligible for **Th1-TIPS**. Therefore, the singlet diradical character increases with the extension of molecular size. Although the molecular length is almost the same in **Th3-TIPS** and **BDTh-TIPS**, the latter shows a significantly larger diradical character, which can be ascribed to their different quinoidal structures. That is, instead of the thiophene ring in **Th3-TIPS**, the benzene ring in **BDTh-TIPS** would lead to more aromatic resonance energy recovery in the diradical form and hence promote the diradical character. Another noticeable observation is that **Th2-TIPS** and **Th3-TIPS** have a remarkably larger diradical character than their anti-aromatic counterparts **S2-TIPS** (*y*_0_ = 0) and **S3-TIPS** (*y*_0_ = 0.03), which have negligible diradical character.9*b* Considering their structural similarity, such differences must be associated with their differential fusion modes. It appears that replacing two indene units in **S*n*-TIPS** by two benzo-thia units in **Th*n*-TIPS** significantly promotes the diradical character and consequently affects their physical properties. This can be correlated to a fundamental change from anti-aromatic **S*n*-TIPS** to pro-aromatic **Th*n*-TIPS**. These results also demonstrated that the diradical character and physical properties of quinoidal compounds are not only dependent on the length of the central quinoidal unit, but also the overall pro-aromaticity and anti-aromaticity of the molecules. It seems that pro-aromatic molecules show larger diradical character in comparison to their anti-aromatic analogues with a similar molecular size. In fact, the diradical character of **BDTh-TIPS** is larger than for the higher order anti-aromatic **S4-TIPS** (*y*_0_ = 0.202).9*b*

Nucleus independent chemical shift (NICS) calculations were conducted to provide the trend of aromaticity of each ring (Fig. 5). From **Th1-TIPS** to **Th2-TIPS**, to **Th3-TIPS** and to **BDTh-TIPS**, the NICS(1)_zz_ values for the central quinoidal rings (ring C and D) become more negative, indicating an increase of aromaticity with the extension of the molecular size, which is in accordance with the increased diradical character. At the same time, in contrast to their counterparts **S*n*-TIPS** compounds, the NICS(1)_zz_ values for the central quinoidal rings of **Th*n*-TIPS** and **BDTh-TIPS** are more negative, indicating a larger aromaticity in **Th*n*-TIPS** and **BDTh-TIPS** while **S*n*-TIPS** are typically anti-aromatic. The sulfur-containing six member rings (ring B) show positive NICS(1)_zz_ values, indicating a non-aromatic to anti-aromatic character. Such sulfur containing six member rings are further annulated and stabilized by two aromatic benzene rings (ring A) possessing large negative NICS(1)_zz_ values.

### Raman spectroscopic measurements

**Fig. 7:**
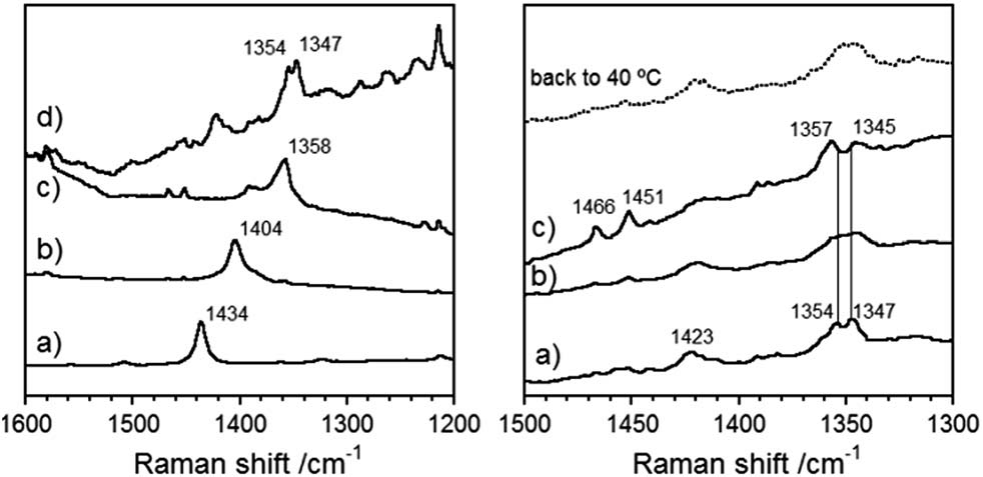
Left: 785 nm Raman spectrain solid state of: (a) **Th1-TIPS**, (b) **Th2-TIPS**, (c) **Th3-TIPS** and (d) **BDTh-TIPS**. Right: Raman spectra of **BDTh-TIPS** in solid state at (a) –140 °C, (b) 40 °C, (c) +140 °C.

Raman spectroscopy has been proven to be very useful for the characterization of the ground electronic state of π-conjugated molecules and in particular those oligomer series with quinoidal/aromatic transformations.^17^ Therefore, the Raman spectra of **Th*n*-TIPS** and **BDTh-TIPS** were recorded in a powder form with different excitation wavelengths (Fig. 7). Generally, there are two main characteristics in the frequency and intensity behavior: (1) there is a main band that dominates the spectra of **Th1-TIPS**–**Th3-TIPS** and that progressively downshifts from 1434 cm^–1^ in **Th1-TIPS** to 1358 cm^–1^ in **Th3-TIPS** whose frequency positions are typical of quinoidal tetracyano oligothiophenes.2*d* The overall shift is of 76 cm^–1^, which compares with the 61 cm^–1^ in the **S1-TIPS**–**S3-TIPS** compounds, and is in agreement with a more efficient electron delocalized **Th*n*-TIPS** systems free of any anti-aromatic electron pinning effect; (2) passing from **Th3-TIPS** to **BDTh-TIPS**, the main Raman band is split in two 1354/1347 cm^–1^ components indicating that from the main band in **Th3-TIPS** at 1358 cm^–1^ the whole 76 cm^–1^ downshift on **Th1-TIPS** → **Th3-TIPS** collapses or is close to collapse on **Th3-TIPS** → **BDTh-TIPS**. Furthermore, a new band at 1423 cm^–1^ appears in **BDTh-TIPS** which is at frequencies in between those of **Th1-TIPS** and **Th2-TIPS** allowing it to be assigned as a quinoidal feature in **BDTh-TIPS**. This spectral behaviour can be explained by a saturation of the quinoidization effect from **Th*n*-TIPS** (*n* = 1–3) to **BDTh-TIPS** resulting from a pseudo-aromatization in the central benzene of the conjugated backbone accompanied by a portion of quinoidal contribution on the thiophene rings. A similar behaviour has been reported in a quinoidal tetracyano-quaterthiophene and ascribed to the formation of a diradicaloid singlet ground electronic state with a pseudo-aromatic (pseudo-quinoidal) central (peripheral) structure on its rings.2*d*

Variable-temperature Raman experiments on **BDTh-TIPS** in Fig. 7 are performed to scan the evolution of the diradical ground electronic state (singlet) into the lowest energy lying excited state (triplet). On heating up to 140 °C, the most noticeable finding is the coalescence of the 1423 cm^–1^ thieno-quinoidal band and the simultaneous emergence of two new medium bands at 1466/1451 cm^–1^. Characteristic Raman bands around 1460 cm^–1^ have been reported for aromatic thienoacene cores^18^ meaning that the clearance of the quinoidal band (1423 cm^–1^) is at the expenses of the rise of aromatic ones (1466/1451 cm^–1^). In addition the pre-existing most intense Raman scatterings around 1350 cm^–1^ enlarge their separation from low temperature (7 cm^–1^) to high (12 cm^–1^) temperature revealing the transformation between two species, one at low temperature with a less defined aromatic structure (pseudo-aromatic or singlet with incipient diradical structure) and another at high temperatures with more distinguished aromatic feature typical of diradical triplets. This study is also in agreement with the above mentioned VT NMR/ESR/SQUID measurements.

### TA and TPA measurements

**Fig. 8:**
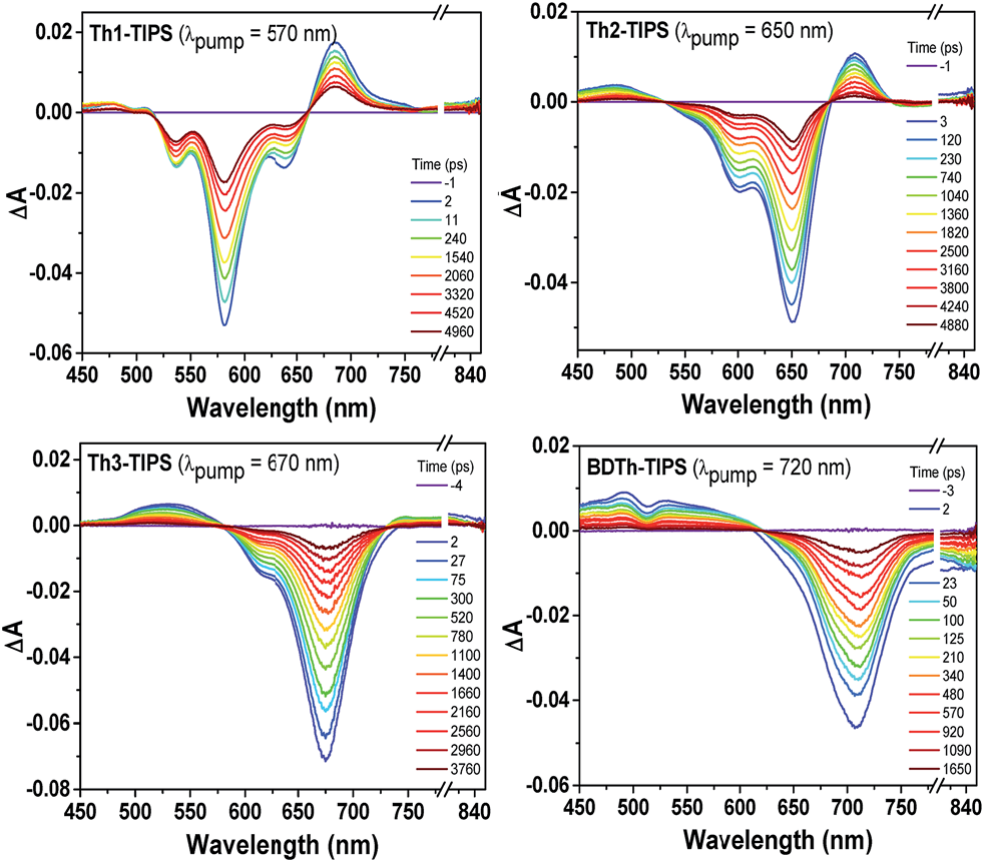
Femtosecond transient absorption spectra of **Th*n*-TIPS** (*n* = 1–3) and **BDTh-TIPS** measured in toluene with photoexcitation at 570, 650, 670, and 720 nm, respectively.

Femtosecond transient absorption (TA) measurements were then utilized to explore the excited-state dynamics of **Th*n*-TIPS** (*n* = 1–3) and **BDTh-TIPS**. Their TA spectra in toluene exhibit a strong ground-state bleaching (GSB) band near their steady-state absorption maximum (Fig. 8). On the other hand, they all display weak excited-state absorption (ESA) bands which are similar to the pro-aromatic diradicaloids such as heptazethrene and octazethrene derivatives5*e* but distinctively different from their anti-aromatic counterparts **S*n*-TIPS**.9*b* The decay curves can be fitted by a bi-exponential process and the singlet excited-state lifetimes of **Th*n*-TIPS** (*n* = 1–3) and **BDTh-TIPS** were estimated to be 5800, 2800, 1700 and 820 ps (Table 1 and Fig. S2 in ESI†), respectively, which are much longer than for the anti-aromatic **S*n*-TIPS** (7–12 ps),9*b* again highlighting the dramatic difference between the pro-aromatic and anti-aromatic electronic structures and their impact on the electronic excited states. Notably, the singlet excited-state lifetimes decrease with the increment of molecular length, which is consistent with a gradually increased diradical character. As a consequence, modest fluorescence was observed for **Th1-TIPS** but no emission was detected for **Th2-TIPS**, **Th3-TIPS**, and **BDTh-TIPS**.^19^

π-Conjugated molecules with a small and moderate diradical character are theoretically predicted by Nakano *et al.* to show a largely enhanced third-order NLO response with large TPA cross sections.^20^ Thus, TPA measurements were also conducted for **Th*n*-TIPS** (*n* = 2 and 3) and **BDTh-TIPS** in toluene by using Z-scan technique in the NIR region from 1200 to 1500 nm where the OPA contribution is negligible (Fig. 2, Table 1 and Fig. S3 in ESI†). The maximum TPA cross section values (*σ*_max_^(2)^) increase from 620 GM (*λ*_ex_: 1300 nm) for **Th2-TIPS** to 720 GM (*λ*_ex_: 1300 nm) for **Th3-TIPS** and to 910 GM (*λ*_ex_: 1400 nm) for **BDTh-TIPS** with the increment of the diradical character, and they are much larger than those of their anti-aromatic counterparts **S*n*-TIPS** (*σ*_max_^(2)^ = 420 GM for **S2-TIPS**, and *σ*_max_^(2)^ = 520 GM for **S3-TIPS**). Unfortunately, the short OPA absorption maximum of **Th1-TIPS** pushes its TPA measurements out of our instrument measuring range. Anyway, again, we showed that the pro-aromatic compounds exhibited a stronger third-order NLO response than their anti-aromatic counterparts with a similar molecular size, and the larger the diradical character, the larger the TPA cross section value.

### Electrochemical and spectroelectrochemical studies

**Fig. 9:**
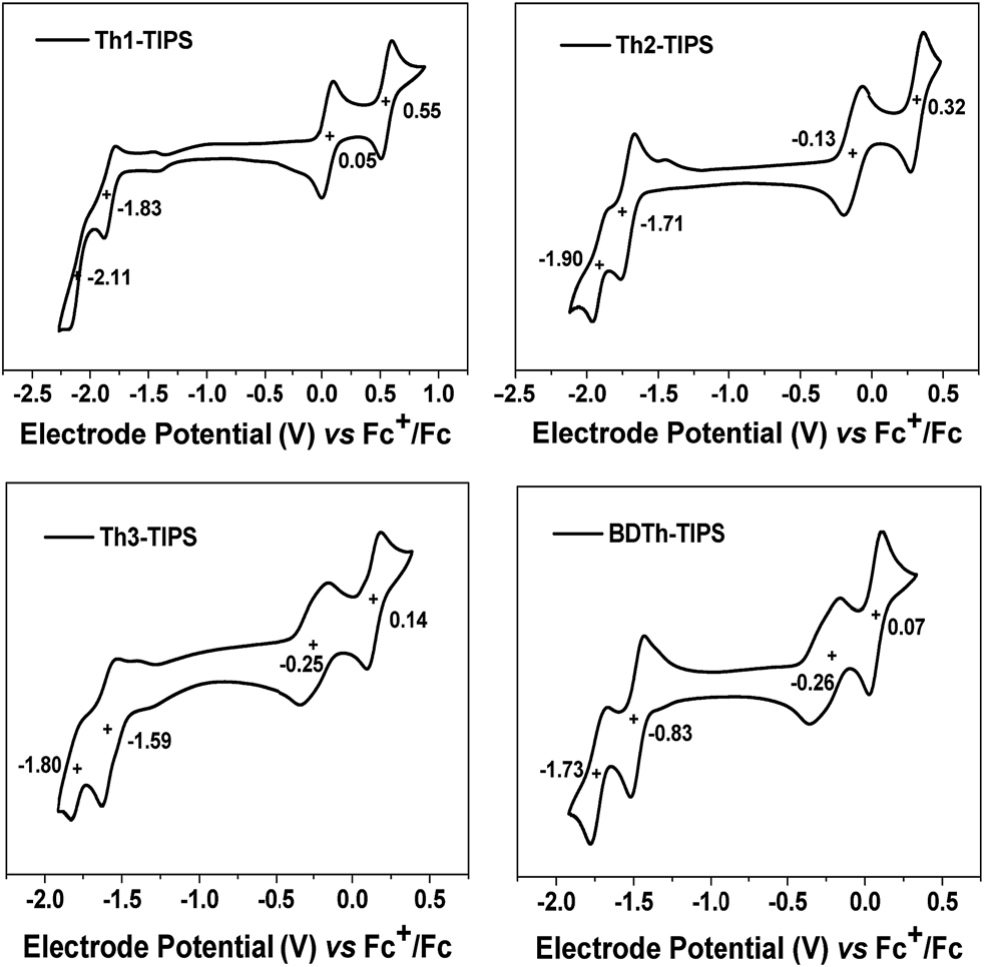
Cyclic voltammograms of **Th1-TIPS**, **Th2-TIPS**, **Th3-TIPS** and **BDTh-TIPS** in dry CH_2_Cl_2_ containing 0.1 M Bu_4_NPF_6_ as the supporting electrolyte, AgCl/Ag as the reference electrode, Au as the working electrode, Pt wire as the counter electrode, and a scan rate of 50 mV s^–1^. The potential was externally calibrated against the ferrocene/ferrocenium redox couple.

The electrochemical properties of all compounds were investigated by cyclic voltammetry (CV) in dry DCM solution (Fig. 9 and Table 1). All compounds display amphoteric redox behaviour with four-stage (quasi-) reversible redox waves. **Th1-TIPS** exhibited two reversible oxidation waves with half-wave potential *E*ox1/2 at 0.05 V, 0.55 V and two quasi-reversible reduction waves at *E*red1/2 = –1.83 V, –2.11 V (*vs.* Fc^+^/Fc, Fc: ferrocene). **Th2-TIPS** and **Th3-TIPS** also displayed two oxidation waves at *E*ox1/2 = –0.13 V, 0.32 V and *E*ox1/2 = –0.25 V, 0.14 V as well as two reduction waves at *E*red1/2 = –1.71 V, –1.90 V and *E*red1/2 = –1.59 V, –1.80 V, respectively. Similarly, **BDTh-TIPS** showed two oxidation waves at *E*ox1/2 = –0.26 V, 0.07 V and two reduction waves at *E*red1/2 = –0.83 V, –1.73 V. The HOMO and LUMO energy levels determined from the onset of the first oxidation and reduction waves, respectively, are summarized in Table 1. It is clear that extension of the molecular size from **Th1-TIPS** to **BDTh-TIPS** leads to a rising of the HOMO energy level and lowering of LUMO energy level, and consequently a decrease of the electrochemical energy band gaps (*E*ECg), which is consistent with the optical energy gap (*E*optg) determined from the lowest-energy absorption band onset. For comparison, the anti-aromatic counterparts **S*n*-TIPS** (*n* = 1–3) are difficult to oxidize due to the local anti-aromatic character of the cyclopentadienyl cation but easy to reduce due to the aromatic character of the cyclopentadienyl anion. The pro-aromatic character of our new series of molecules showed good amphotericity because both oxidation and reduction will lead to recovery of the aromaticity of the central quinoidal thienoacene moiety and there are no other anti-aromatic units.

The multistage reversible redox waves and the large separation between the redox waves allow us to quantitatively attain the singly and doubly charged species by electrochemistry. UV-vis-NIR spectroelectrochemical measurements were thus conducted for **Th*n*-TIPS** (*n* = 1–3) and **BDTh-TIPS** in DCM containing 0.1 M *n*-Bu_4_NPF_6_ as the supporting electrolyte by applying different electrode potentials and the absorption spectra were monitored *in situ* in the UV-vis-NIR range. Fig. 9 shows the electronic absorption spectra of the four compounds in their different redox states according to their electrochemical properties measured by cyclic voltammetry.

**Fig. 10:**
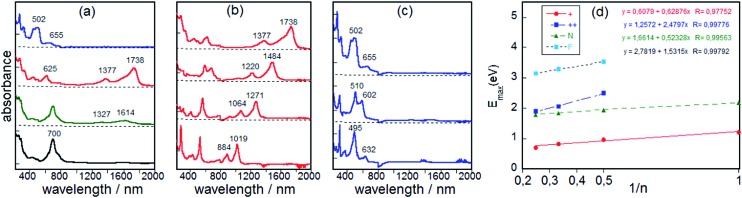
(a) UV-vis-NIR absorption spectra obtained by electrochemical redox treatment of the neutral **BDTh-TIPS** (black line), radical cation-1 (green line), radical cation (red line) and dication (blue line); (b) spectra of the radical cations of **Th1-TIPS**, **Th2-TIPS**, **Th3-TIPS** and **BDTh-TIPS** (from the bottom to the top); (c) spectra of the dications of **Th2-TIPS**, **Th3-TIPS** and **BDTh-TIPS** (from the bottom to the top); (d) representation of the absorption maxima as a function of the reciprocal of the chain length (N: neutral; +: radical cation; ++: dication; F: thienoacenes).

One electron oxidation of **BDTh-TIPS** gives rise to the appearance, at the initial stage of the oxidation, of two weak bands at 1327 and 1614 nm which quickly convert into a spectrum with stronger bands at 625, 1377 and 1738 nm (Fig. 10a). During this first oxidation, the two described spectra coexist. The 625/1377/1738 nm spectrum of the radical cation of **BDTh-TIPS** is surprisingly similar to that described for the radical anions of the **S*n*-TIPS** (*n* = 1–3).9*b* The UV-vis-NIR absorption spectra of the radical cations of **Th1-TIPS**, **Th2-TIPS** and **Th3-TIPS** have also been recorded in Fig. 10 which show quite similar patterns to that of **BDTh-TIPS** (Fig. 10b).

One-electron oxidation of the radical cation produces the clearance of the NIR bands and the growth of intense absorption bands in the visible 500–600 nm region (Fig. 10a and c; a reliable spectrum for the dication of **Th1-TIPS** was not obtained). We assign the characteristic bands of the dication to the bands at 495 nm for **Th2-TIPS**, at 602 nm for **Th3-TIPS**, and at 655 nm for **BDTh-TIPS**. Interestingly, a linear relationship between the energy gap *λ*_max_ (eV) with the reciprocal number of the chain length (1/*n*, herein *n* is the number of thiophene rings in the central quinoidal thienoacene unit and **BDTh-TIPS** was assumed to be an equivalence of **Th4-TIPS** which was not synthesized due to synthetic challenges) was observed for the neutral, radical cationic, and dicationic species (Fig. 10d). The slope for the dications is greater than for the radical cations, likely revealing the strongest pinning of the electron wavefunction. We have also represented the variation *λ*_max_ (eV)–1/*n* for neutral thienoacenes^21^ (Fig. 10d) and obtained a very similar behavior to that found for our dications in consonance with the progressive aromatization of the initial quinoidal structure of **Th*n*-TIPS** (*n* = 1–3) and **BDTh-TIPS** after two-electron extraction.

## Conclusions

In summary, a series of quinoidal benzo-thia-fused [*n*]thienoacenequinodimethanes **Th*n*-TIPS** (*n* = 1–3) and **BDTh-TIPS** were successfully synthesized. Their ground-state geometry and electronic structures were systematically studied by various experimental techniques (electronic absorption spectra, X-ray crystallographic analysis, VT NMR/ESR/SQUID and Raman spectroscopy) and DFT calculations. Our studies revealed that the transformation of the electronic structure from anti-aromatic in the bisindeno-[*n*]thienoacenes **S*n*-TIPS** to pro-aromatic in this new series of quinoidal thienoacenes could be achieved by simply replacing the indene units by fused benzo-thia moieties. Consequently, these pro-aromatic compounds show larger diradical character, much longer singlet excited state lifetime, larger TPA cross section values and better redox amphotericity compared with their anti-aromatic counterparts **S*n*-TIPS**. In addition, extension of molecular length leads to an increase of the diradical character, convergence of optical/electrochemical energy gap, decrease of singlet excited state lifetime and enhancement of TPA cross section. The observed modest singlet excited state lifetime, moderate TPA cross section and amphoteric redox behavior are all related to their pro-aromaticity and small to moderate diradical character. Spectroelectrochemical studies revealed their spectroscopic features of the neutral and charge species which can be correlated to the change of their electronic structures, and a linear *λ* (eV)–1/*n* relationship was obtained. Our detailed studies provided a fundamental understanding on how the pro-aromaticity and anti-aromaticity affect the ground-state electronic structure, diradical character and consequently the physical properties of quinoidal compounds.

## Supplementary Material

Supplementary information

Crystal structure data
