# Supplementary material for: Benzo-thia-fused [n]thienoacenequinodimethanes with small to moderate diradical characters: the role of pro-aromaticity versus anti-aromaticity
Source: Chem Sci. 2016 Jan 19;7(5):3036–46. doi: 10.1039/c5sc04706d (PMC6005137; doi:10.1039/c5sc04706d)
Supplement: Supplementary file 1 [file SC-007-C5SC04706D-s001.pdf]

Electronic Supplementary Material (ESI) for Chemical Science.
This journal is © The Royal Society of Chemistry 2016

Electronic Supplementary Information (ESI)

Benzo-thia-fused [*n*]Thienoacenequinodimethanes with Small to Moderate Diradical Characters: The Role of Pro-aromaticity *versus* Anti-aromaticity

Xueliang Shi,[a] Estefanía Quintero,[b] Sangsu Lee,[c] Linzhi Jing,[a] Tun Seng Herng,[d] Bin Zheng,[e] Kuo-Wei Huang,[e] Juan T. López Navarrete,[b] Jun Ding,*[d] Dongho Kim,*[c] Juan Casado,*[b] Chunyan Chi*[a]

[a]*Department of Chemistry, National University of Singapore, 3 Science Drive 3, 117543, Singapore*

[b]*Department of Physical Chemistry, University of Malaga, Campus de Teatinos s/n, 229071 Malaga, Spain*

[c]*Department of Chemistry, Yonsei University, Seoul 120-749, Korea*

[d] *Department of Materials Science & Engineering, National University of Singapore, 119260, Singapore*

[e]*Division of Physical Science and Engineering and KAUST Catalysis Center, King Abdullah University of Science and Technology (KAUST), Thuwal 23955-6900, Kingdom of Saudi Arabia*

Table of contents

1. Experimental section……………………………………………………………S2

1.1 General……………………...……………………………………………………S2

1.2 Detailed synthetic procedures and characterization data………………...………S4

2. UV-vis absorption and PL spectra of compound **Th1-TIPS**………………...….S12

3. Transient absorption decay curves……………………..…………...………..S13

4. Z-scan curves……………………...…………………………….……………S13

5. DFT calculation details……………………………………….…………………S14

6. Crystallographic data……………………………………………………………….S21

7. ^{1}H and ^{13}C NMR spectra of all new compounds …………………………....S107

8. Appendix: Energies and Cartesian Coordinates………………………………...S122

1. Experimental Section

1.1. General

All reagents were purchased from commercial sources and used without further purification. Anhydrous dichloromethane (DCM) and *N*,*N*-dimethylformaldehyde were distilled from CaH_2. Anhydrous toluene and THF were distilled from sodium benzophenone immediately prior to use. The ^1H NMR and ^{13}C NMR spectra were recorded in solution of $CDCl_3$, benzene-d_6, toluene-d_8 or DMSO-d_6 on Bruker DRX 500 NMR spectrometer with tetramethylsilane (TMS) as the internal standard. Abbreviations for signal coupling are as follows: s, singlet; d, doublet; t, triplet; q, quartet; m, multiplet; br, broad. MALDI-TOF mass spectra were recorded on a Bruker Autoflex instrument. High resolution (HR) EI mass spectra were recorded on Agilent 5975C DIP/MS mass spectrometer. HR APCI mass spectra were recorded on a MicrOTOF-QII instrument. UV-vis-NIR absorption spectra were recorded on a Shimadzu UV-1700 and UV-3600 spectrometer. The electrochemical measurements were carried out in anhydrous DCM with 0.1 M tetrabutylammonium hexafluorophosphate (Bu_4NPF_6) as the supporting electrolyte at a scan rate of 0.05 V/s at room temperature under the protection of nitrogen. A gold disk was used as working electrode, platinum wire was used as counting electrode, and Ag/AgCl (3M KCl solution) was used as reference electrode. The potential was calibrated against the ferrocene/ferrocenium couple. Continuous wave X-band ESR spectra were obtained with a Bruker ELEXSYS E500 spectrometer using a variable temperature Bruker liquid nitrogen cryostat.

A superconducting quantum interference device (SQUID) magnetometer MPMS was used for the magnetic characterization. Powder sample of **BDTh-TIPS** with a weight of 5-10 mg was sealed in a plastic capsule. Magnetic moment was measured in the temperature range of 2 to 380 K. The empty plastic capsule exhibited diamagnetic and its magnetic moment was measured for correction. After correction of diamagnetic signal of plastic capsule and sample holder, magnetic susceptibility was fitted with Bleaney-Bowers equation:

$$\chi = \frac{N\beta^2 g^2}{3kT}\left[1 + \frac{1}{3}exp\left(\frac{J_{s-t}}{kT}\right)\right]^{-1}$$

Raman spectra in solid state with the excitation laser at 785 nm were collected by using the 1×1 camera of a Bruker Senterra Raman microscope by averaging spectra during 50 minutes with a resolution of 3–5 cm^{-1}. A CCD detection camera operating at -50 ºC was used. Variable temperature Raman measurements were performed in KBr pellets (to assure faster thermal equilibration within the sample) in the range between -140 ºC and 140 ºC, by using a Linkam FTIR600 stage cooled by liquid nitrogen and with a temperature stability of < 0.1°C. Raman spectra were recorded after waiting for thermal equilibrium in the sample. The samples in KBr pellets were prepared in an oxygen and water-free bag.

In situ UV-Vis-NIR spectroelectrochemical studies were conducted on a Cary 5000 UV-Vis-NIR spectrophotometer from Varian operating in a maximal 175-3300 nm range. A C3 epsilon potentiostat from BASi was used for the electrolysis using a thin layer cell from a demountable omni cell from Specac. In this cell a three electrodes system was coupled to conduct *in situ* spectroelectrochemistry. A Pt gauze was used as the working electrode, a Pt wire as the counter electrode and a Ag wire as the pseudoreference electrode. The spectra were collected a constant potential electrolysis and the potentials were changed in interval of 100 mV. The electrochemical medium used was 0.1 M $(n\text{-}C_4H_9)_4NPF_6$ in fresh distilled CH_2Cl_2, at room temperature with sample concentrations of 10^{-3} M.

The femtosecond time-resolved transient absorption spectrometer used for this study consisted of a femtosecond optical parametric amplifier (Quantronix, Palitra-FS) pumped by a Ti:sapphire regenerative amplifier system (Quantronix, Integra-C) operating at 1 kHz repetition rate and an accompanying optical detection system. The generated OPA pulses had a pulse width of ~100 fs and an average power of 1 mW in the range 450 to 800 nm, which were used as pump pulses. White light continuum (WLC) probe pulses were generated using a sapphire window (3 mm thick) by focusing of small portion of the fundamental 800 nm pulses, which were picked off by a quartz plate before entering into the OPA. The time delay between pump and probe beams was carefully controlled by making the pump beam travel along a variable optical delay (Newport, ILS250). Intensities of the spectrally dispersed WLC probe pulses were monitored by miniature spectrograph (OceanOptics, USB2000+). To obtain the time-resolved transient absorption difference signal (ΔA) at a specific time, the pump pulses were chopped at 500 Hz and absorption spectra intensities were saved alternately with or without pump pulse. Typically, 4000 pulses were used to excite samples and to obtain the TA spectra at a particular delay time. The polarization angle between pump and probe beam was set at the magic angle (54.7^{o}) using a Glan-laser polarizer with a half-wave retarder to prevent polarization-dependent signals. The cross-correlation fwhm in the pump-probe experiments was less than 200 fs, and chirp of WLC probe pulses was measured to be 800 fs in the 400-800 nm regions. To minimize chirp, all reflection optics were used in the probe beam path, and a quartz cell of 2 mm path length was employed. After completing each set of fluorescence and TA experiments, the absorption spectra of all compounds were carefully checked to rule out the presence of artifacts or spurious signals arising from, for example, degradation or photo-oxidation of the samples in question.

The two-photon absorption spectrum was measured in the NIR region using the open-aperture Z-scan method with 130 fs pulses from an optical parametric amplifier (Light Conversion, TOPAS) operating at a repetition rate of 1 kHz generated from a Ti:sapphire regenerative amplifier system (Spectra-Physics, Hurricane). After passing through a 10 cm focal length lens, the laser beam was focused and passed through a 1 mm quartz cell. Since the position of the sample cell could be controlled along the laser beam direction (*z* axis) using the motorcontrolled delay stage, the local power

density within the sample cell could be simply controlled under constant laser intensity. The transmitted laser beam from the sample cell was then detected by the same photodiode as used for reference monitoring. The on-axis peak intensity of the incident pulses at the focal point, I_0, ranged from 40 to 60 GW cm^{-2}. For a Gaussian beam profile, the nonlinear absorption coefficient can be obtained by curve fitting of the observed open-aperture traces $T(z)$ with the following equation:

$$T(z)=1-\frac{\beta I_0(1-e^{-\alpha_0 l})}{2\alpha_0[1+(z/z_0)^2]}$$

where α_0 is the linear absorption coefficient, l is the sample length, and z_0 is the diffraction length of the incident beam. After the nonlinear absorption coefficient has been obtained, the TPA cross section $\sigma^{(2)}$ of one solute molecule (in units of GM, where 1 GM = 10^{-50}cm^4 s photon^{-1} molecule^{-1}) can be determined by using the following relationship:

$$\beta=\frac{10^{-3}\sigma^{(2)}N_A d}{h\nu}$$

where N_A is the Avogadro constant, d is the concentration of the compound in solution, h is the Planck constant, and ν is the frequency of the incident laser beam.

1.2. Detailed synthetic procedures and characterization data

Typical procedure 1 (TP1): preparation of the key intermediate dibromo-diesters 4-6

A round-bottomed flask was charged with tetrabromo-compound (**1**-**3**, 1 equiv) and freshly-distilled THF under argon atmosphere. Upon cooling to -78 °C, *n*-butyl lithium (2 equiv) was added drop wise to the solution in the course of 30 min under an inert atmosphere. The reaction mixture was maintained at -78 °C with stirring for a further 120 min. Ethyl cyanoformate (2 equiv) was added in one portion and the mixture was warmed slowly to ambient temperature overnight. The reaction was quenched by water at 0 °C. All of the organic solvents were removed and the organic precipitate was collected by filtration. The crude product was washed by hexane and methanol to give pure compound as a solid.

Typical procedure 2 (TP2): palladium-catalyzed cross-coupling reaction with 4-*tert*-butylbenzenethiol for preparation of compounds 7-9

To a solution of $Pd_2(dba)_3$ (46 mg, 0.050 mmol) and dppf (56 mg, 0.100 mmol) in DMF (15 ml) were added intermediate dibromo- diester compound (**4**-**6**, 1.0 mmol), 4-*tert*-butylbenzenethiol (665 mg, 4.0 mmol), and $^{i}Pr_2NEt$ (0.9 mL, 5.2 mmol) at room temperature. The solution was heated to 100 °C and stirred overnight. Upon cooling to room temperature, the reaction was then quenched by addition of water and extracted with ethyl acetate. The organic layer was washed by 10% hydrochloric acid solution and dried over anhydrous Na_2SO_4. The solvent was removed under vacuum and the residue was purified by column chromatography.

Typical procedure 3 (TP3): hydrolysis and acidification reaction for preparation of compounds 10-12

Precursor compound (**7**-**9**, 2 mmol) was dissolved in 60 mL ethanol, followed by the addition of an excess of sodium hydroxide (0.8 g). This mixture was heated to reflux overnight. The solvent was removed by rotary evaporator after the reaction was completed. To the residue then 10% hydrochloric acid solution was added. The precipitate formed was collected by filtration and washed with water and hexane, then dried under vacuum to afford diacid compound (**10**-**12**) as a solid.

Typical procedure 4 (TP4): intramolecular double Friedel–Crafts acylation reaction for preparation of diketone compounds 13-15

Diacid compound (**10**-**12**, 1 mmol) was added in anhydrous DCM (30 mL) in 100 mL two-neck round bottom flask, followed by the addition of excess of thionyl chloride (1 mL). To the mixture anhydrous DMF (1-2 drops) was added at room temperature. The reaction mixture was heated at reflux overnight. After cooling down the solvent was removed by rotary evaporator to afford crude acyl chloride. This intermediate compound was re-dissolved in anhydrous DCM (20 mL) then anhydrous $AlCl_3$ (533 mg, 4.00 mmol) was added carefully at 0 °C. The resultant mixture was allowed to warm up to room temperature and stirred overnight. The reaction mixture was slowly quenched by 10% HCl solution to give a yellow precipitate. After filtration, the crude precipitate was washed with methanol, and hexane until the filtrate was colorless, giving diketone compound (**13**-**15**) as an insoluble solid.

Typical procedure 5 (TP5): nucleophilic addition with lithiated Li-TIPSE and reductive dehydroxylation reaction for preparation of compounds Thn-TIPS

To a solution of triisopropylsilylacetylene (365 mg, 2 mmol) in anhydrous THF (20 mL) at 0 ^{o}C was added dropwise *n*-BuLi (2 M in cyclohexane, 1 mL, 2 mmol). The solution was stirred for 30 min at 0 ^{o}C. Then diketone compound (**13**-**15**, 0.2 mmol) was added in one portion. The mixture was slowly warmed to room temperature and stirred overnight. During this period the insoluble diketone disappeared and the solution became clear. After that, $SnCl_2$ (190 mg, 1 mmol) was added and the solution became deep color immediately. Upon completion of the reaction as monitored by TLC, the resulting solution was filtered over anhydrous Na_2SO_4 and the solvent was subsequently removed by rotary evaporator. The residue was purified by column chromatography (aluminium oxide, hexane: DCM =8:1). The target compound was further purified by recrystallization from MeOH/CH_2Cl_2 as a dark solid.

Compound 4

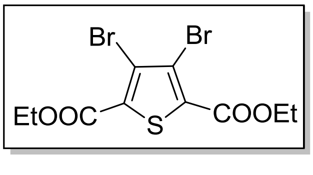

Prepared according to **TP1** from **1** (2 g, 5.0 mmol), white solid; Yield: 1.16 g, 60%; ^{1}H NMR (500 MHz, $CDCl_3$, ppm): δ = 4.41 (q, *J* = 7.1 Hz, 4H), 1.40 (t, *J* = 7.2 Hz, 6H); ^{13}C NMR (125 MHz, $CDCl_3$, ppm): δ = 159.52, 132.15, 121.72, 62.35, 14.12. HR MS (EI): calcd for $C_{10}H_{10}Br_2O_4S$ (M^+), 383.86665; found, 383.86653(error: -0.32 ppm).

Compound 5

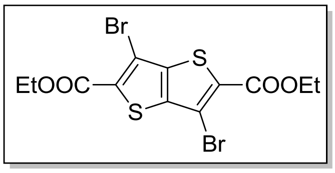

Prepared according to **TP1** from **2** (2.28 g, 5.0 mmol), white solid; Yield: 1.55 g, 70%; ^{1}H NMR (500 MHz, $CDCl_3$, ppm): δ = 4.43 (q, *J* = 7.1 Hz, 2H), 1.42 (t, *J* = 7.2 Hz, 3H); ^{13}C NMR (125 MHz, $CDCl_3$, ppm): δ = 160.48, 142.46, 132.04, 109.55, 62.13, 14.19. HR MS (EI): calcd for $C_{12}H_{10}Br_2O_4S_2$ (M^+), 441.8367; found, 441.8365 (error: -0.45 ppm).

Compound 6

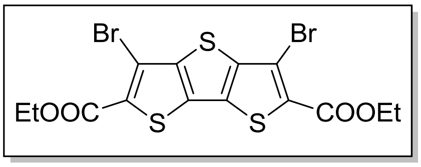

Prepared according to **TP1** from **3** (511 mg, 1.0 mmol), white solid; Yield: 324 mg, 65%; ^{1}H NMR (500 MHz, $CDCl_3$, ppm): δ = 4.43 (q, *J* = 7.1 Hz, 2H), 1.43 (t, *J* = 7.2 Hz, 3H); ^{13}C NMR (125 MHz, $CDCl_3$, ppm): δ = 160.59, 146.06, 132.46, 129.29, 110.45, 62.01, 14.24. HR MS (EI): calcd for $C_{14}H_{10}Br_2O_4S_3$ (M^+), 495.81080; found, 495.81087 (error: 0.14 ppm).

Compound 7

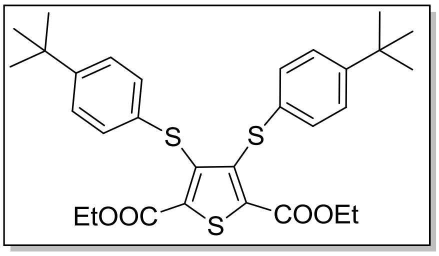

Prepared according to **TP2** from **4** (386 mg, 1.0 mmol), yellowish oil; Yield: 500 mg, 90%; ^{1}H NMR (500 MHz, $CDCl_3$, ppm): δ = 7.19 (d, *J* = 8.4 Hz, 4H), 7.05 (d, *J* = 8.5 Hz, 4H), 4.26 (q, *J* = 7.1, 4H), 1.25 (m, 24H); ^{13}C NMR (125 MHz, $CDCl_3$, ppm): δ = 160.30, 149.41, 141.76, 137.71, 133.06, 128.62, 125.86, 61.95, 34.38, 31.20, 14.01.

HR MS (EI): calcd for $C_{30}H_{36}O_4S_3$ (M^+), 556.17758; found, 556.17889 (error: 2.37 ppm).

Compound 8

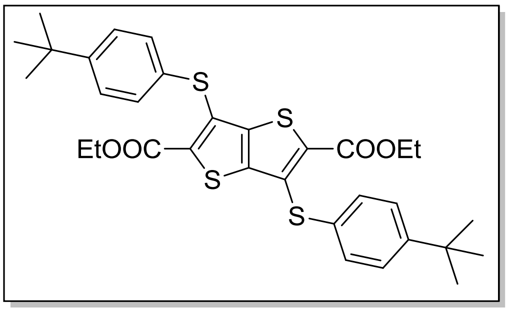

Prepared according to **TP2** from **5** (442 mg, 1.0 mmol), pale yellow solid; Yield: 515mg, 85%; ^{1}H NMR (500 MHz, $CDCl_3$, ppm): δ = 7.54 (d, *J* = 8.4 Hz, 4H), 7.42 (d, *J* = 8.2 Hz, 4H), 4.27 (q, *J* = 7.1, 4H), 1.38 (s, 18H), 1.28 (t, *J* = 7.1 Hz, 6H); ^{13}C NMR (125 MHz, $CDCl_3$, ppm): δ = 162.06, 153.90, 140.65, 135.94, 135.07, 127.37, 126.37, 126.30, 61.31, 34.87, 31.26, 14.25. HR MS (EI): calcd for $C_{32}H_{36}O_4S_4$ (M^+), 612.14965; found, 612.14940 (error: -0.41ppm).

Compound 9

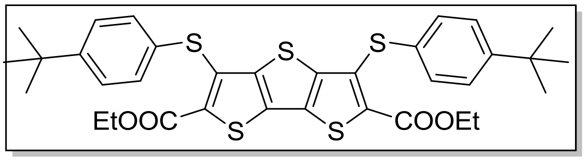

Prepared according to **TP2** from **6** (498 mg, 1.0 mmol), yellowish solid; Yield: 535mg, 80%; ^{1}H NMR (500 MHz, $CDCl_3$, ppm): δ = 7.29 (s, 8H), 4.39 (q, *J* = 7.1, 4H), 1.38 (t, *J* = 7.1 Hz, 24H); ^{13}C NMR (125 MHz, $CDCl_3$, ppm): δ = 161.77, 152.28, 146.25, 134.86, 133.46, 131.95, 127.95, 127.78, 126.14, 61.61, 34.73, 31.28, 14.28. HR MS (EI): calcd for $C_{34}H_{36}O_4S_5$ (M^+), 668.12172; found, 668.12103 (error: -1.03 ppm).

Compound 10

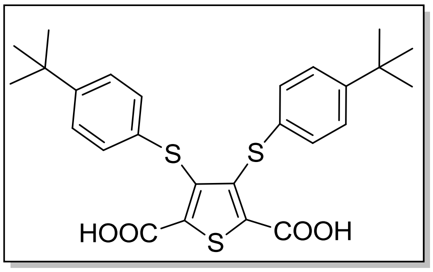

Prepared according to **TP3** from **7** (1.1 g, 2 mmol), yellow solid; Yield: 950 mg, 95%; ^{1}H NMR (500 MHz, DMSO-d_6, ppm): δ = 13.88 (br, 2H), 7.24 (d, *J* = 8.6 Hz, 4H), 6.93 (d, *J* = 8.5 Hz, 4H), 1.21 (s, 18H); ^{13}C NMR (125 MHz, DMSO-d_6, ppm): δ =

160.92, 148.53, 139.65, 139.12, 133.19, 127.35, 125.85, 34.08, 30.95. HR MS (EI): calcd for $C_{26}H_{28}O_4S_3$ (M^+), 500.11497; found, 500.11534 (error: 0.73 ppm).

Compound 11

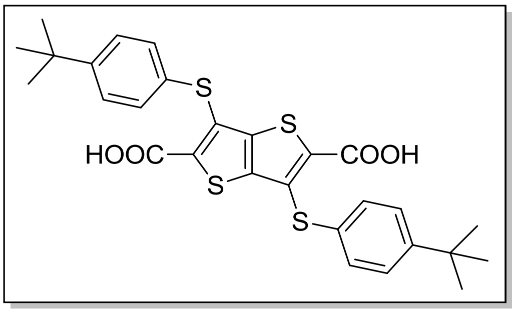

Prepared according to **TP3** from **8** (1.2 g, 2 mmol), yellow solid; Yield: 1.1 g, 95%; ^{1}H NMR (500 MHz, DMSO-d_6, ppm): δ = 13.42 (br, 2H), 7.52 (d, *J* = 8.2 Hz, 4H), 7.47 (d, *J* = 8.2 Hz, 4H), 1.31 (s, 18H); ^{13}C NMR data was not obtained due to its poor solubility. HR MS (EI): calcd for $C_{28}H_{28}O_4S_4$ (M^+), 556.08705; found, 556.08629 (error: -1.36 ppm).

Compound 12

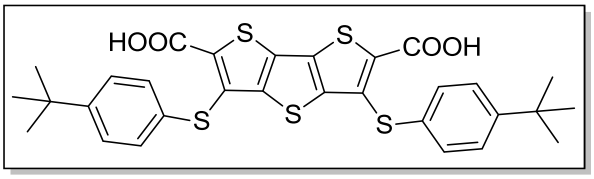

Prepared according to **TP3** from **9** (1.34 g, 2 mmol), yellow solid; Yield: 1.16 g, 95%; ^{1}H NMR (500 MHz, DMSO-d_6, ppm): δ = 13.65 (br, 2H), 7.35 (d, *J* = 8.3 Hz, 4H), 7.24 (d, *J* = 8.3 Hz, 4H), 1.31 (s, 18H); ^{13}C NMR (125 MHz, DMSO-d_6, ppm): δ = 162.41, 151.78, 144.98, 132.73, 132.21, 131.34, 130.08, 127.39, 126.22, 34.43, 30.94. HR MS (APCI): calcd for $C_{30}H_{28}O_4S_5$ ($M+H^+$), 613.0664; found, 613.0656 (error: 1.2 ppm).

Compound 13

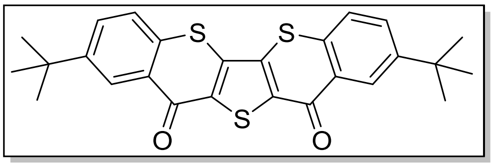

Prepared according to **TP4** from **10** (500 mg, 1 mmol), yellow solid; Yield: 394 mg, 85%; ^{1}H NMR (500 MHz, $CDCl_3$, ppm): δ = 8.69 (s, 2H), 7.77 (d, *J* = 8.5 Hz, 2H), 7.67 (d, *J* = 8.5 Hz, 2H), 1.43 (s, 18H); ^{13}C NMR (125 MHz, $CDCl_3$, ppm): δ = 175.42, 151.55, 137.56, 134.58, 133.18, 130.48, 128.99, 126.82, 125.43, 35.16, 31.18. HR MS (EI): calcd for $C_{26}H_{24}O_2S_3$ (M^+), 464.09385; found, 464.09419 (error: 0.75 ppm).

Compound 14

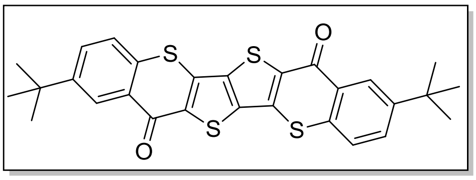

Prepared according to **TP4** from **11** (607 mg, 1 mmol), yellow solid; Yield: 457 mg, 80%; Both ^{1}H NMR and ^{13}C NMR data were not obtained due to its poor solubility. HR MS (EI): calcd for $C_{28}H_{24}O_2S_4$ (M^+), 520.06592; found, 520.06520 (error: -1.38 ppm).

Compound 15

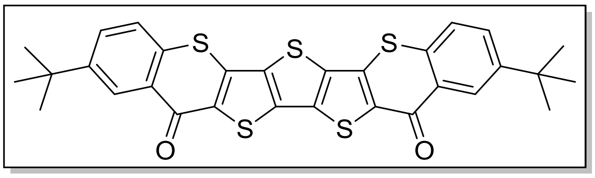

Prepared according to **TP4** from **12** (612 mg, 1 mmol), yellow solid; Yield: 432 mg, 80%; Both ^{1}H NMR and ^{13}C NMR data were not obtained due to its poor solubility. HR MS (APCI): calcd for $C_{30}H_{25}O_2S_5$ ($M+H^+$), 577.0453; found, 577.0451 (error: 0.3ppm).

Compound 17

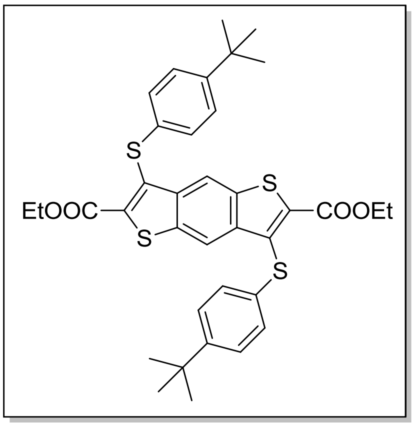

4-*tert*-Butylbenzenethiol (1.33 g, 8 mmol) and K_2CO_3 (1.11 g, 8 mmol) were added to 40 mL dry DMF and the mixture was stirred at room temperature for 30 minutes. After that, compound **16** (1.26 g, 2 mmol) was added as solid in one portion to this reaction mixture and stirred overnight. Then the mixture was poured into cold 10% hydrochloric acid solution and extracted with ethyl acetate. The organic layer was washed by 10% hydrochloric acid solution and dried over anhydrous Na_2SO_4. The solvent was removed under vacuum and the residue was purified by column chromatography (silica, hexane: DCM = 2: 1) to give compound **17** as pare yellow

solid (1.21 g, 91% yield). ^{1}H NMR (500 MHz, $CDCl_3$, ppm): δ = 8.35 (s, 2H), 7.24 (d, *J* = 8.6 Hz, 4H), 7.16 (d, *J* = 8.4 Hz, 4H), 4.39 (q, *J* = 7.2 Hz, 4H), 1.35 (t, *J* = 7.2 Hz, 6H), 1.26 (s, 18H); ^{13}C NMR (125 MHz, $CDCl_3$, ppm): δ = 161.52, 149.64, 140.23, 137.21, 137.06, 132.22, 130.02, 128.22, 126.17, 119.44, 62.01, 34.43, 31.19, 14.13. HR MS (EI): calcd for $C_{36}H_{38}O_4S_4$ (M^+), 662.1653; found, 662.1662 (error: 1.36 ppm).

Compound 18

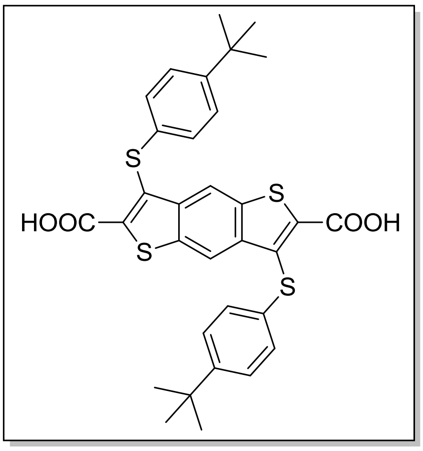

Prepared according to **TP3** from **17** (1.33 g, 2 mmol), yellow solid; Yield: 1.15 g, 95%; ^{1}H NMR (500 MHz, DMSO-d_6, ppm): δ = 13.95 (br, 2H), 8.54 (s, 2H), 7.28 (d, *J* = 8.4 Hz, 4H), 7.13 (d, *J* = 8.6 Hz, 4H), 1.20 (s, 18H); ^{13}C NMR (125 MHz, DMSO-d_6, ppm): δ = 162.23, 149.03, 139.81, 139.72, 136.43, 132.06, 127.78, 127.61, 126.21, 119.28, 34.12, 30.90. HR MS (EI): calcd for $C_{32}H_{30}O_4S_4$ (M^+), 606.1027; found, 606.1005 (error: -3.63 ppm).

Compound 19

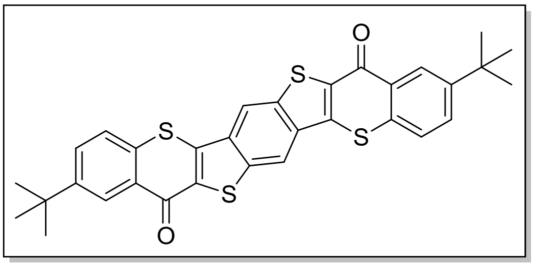

Prepared according to **TP4** from **18** (607 mg, 1 mmol), yellow solid; Yield: 491 mg, 85%; Both ^{1}H NMR and ^{13}C NMR data were not obtained due to its poor solubility. HR MS (APCI): calcd for $C_{32}H_{27}O_2S_4$ $(M + H)^+$, 571.0894; found, 571.0895 (error: 0.18 ppm).

Compound Th1-TIPS

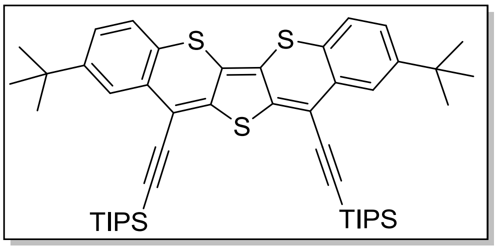

Prepared according to **TP5** from diketone **13** (116 mg, 0.25 mmol), dark red solid; Yield: 160 mg, 60%; ^{1}H NMR (500 MHz, $CDCl_3$, ppm): δ = 7.87 (s, 2H), 7.18 (d, *J* = 8.2 Hz, 2H), 7.11 (d, *J* = 8.3 Hz, 2H), 1.32 (s, 18H), 1.20 (s, 42H); ^{13}C NMR (125 MHz, $CDCl_3$, ppm): δ =150.08, 144.37, 128.90, 125.40, 124.89, 124.65, 124.38, 121.59, 106.19, 103.96, 103.11, 34.67, 31.17, 18.83, 11.29. HR MS (EI): calcd for $C_{48}H_{66}S_3Si_2$ (M^+), 794.38652; found, 794.38554 (error: 0.75 ppm).

Compound Th2-TIPS

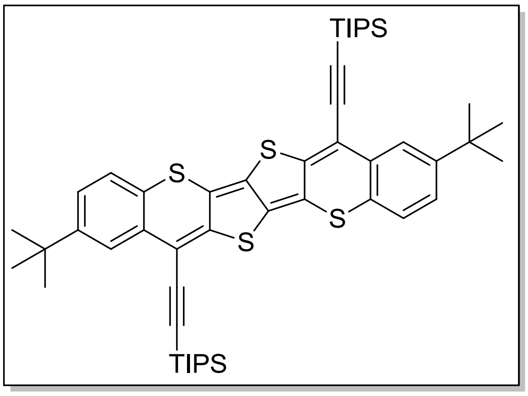

Prepared according to **TP5** from diketone **14** (114 mg, 0.2 mmol), dark blue solid; Yield: 135 mg, 75%; ^{1}H NMR (500 MHz, benzene-d_6, ppm): δ = 8.02 (s, 2H), 6.85 (d, *J* = 8.3 Hz, 2H), 6.75 (d, *J* = 8.3 Hz, 2H), 1.29-1.28 (m, 42H), 1.25 (s, 18H); ^{13}C NMR (125 MHz, benzene-d_6, ppm): δ =150.25, 150.10, 134.71, 129.47, 125.51, 125.32, 125.06, 124.71, 117.65, 108.60, 104.44, 104.35, 34.69, 31.26, 19.12, 11.79. HR MS (EI): calcd for $C_{50}H_{66}S_4Si_2$ (M^+), 850.35860; found, 850.35777 (error: -0.97 ppm).

Compound Th3-TIPS

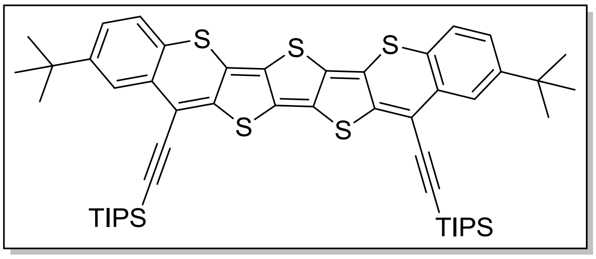

Prepared according to **TP5** from diketone **15** (115 mg, 0.2 mmol), dark green solid; Yield: 108 mg, 60%; ^{1}H NMR (500 MHz, benzene-d_6, ppm): δ = 8.00 (s, 2H), 6.90-6.70 (m, 4H), 1.26-1.23 (m, 42H), 1.12 (s, 18H); ^{13}C NMR data was not obtained due to its poor solubility. HR MS (APCI): calcd for $C_{52}H_{66}S_5$ Si_2 (M+H$^+$), 907.3379; found, 907.3379 (error: 0 ppm).

Compound BDTh-TIPS

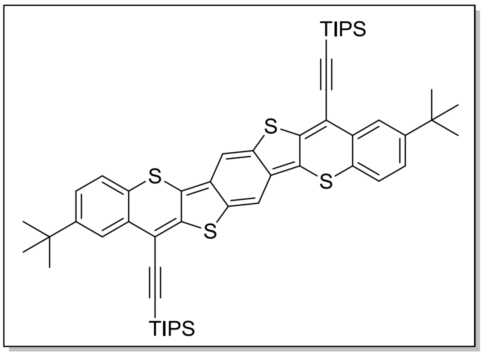

Prepared according to **TP5** from diketone **19** (115 mg, 0.2 mmol), dark green solid; Yield: 100 mg, 55%; ^{1}H NMR (500 MHz, toluene-d_8, ppm): δ = 8.03 (s, 2H), 6.95-6.80 (br, 4H), 6.69 (s, 2H), 1.33-1.30 (m, 42H), 1.27 (s, 18H); ^{13}C NMR data was not obtained due to its poor solubility. HR MS (APCI): calcd for $C_{54}H_{69}S_4Si_2$ (M + H)$^+$, 901.3821; found, 901.3813 (error: -0.89 ppm).

2. UV-vis absorption and PL spectra of compound Th1-TIPS

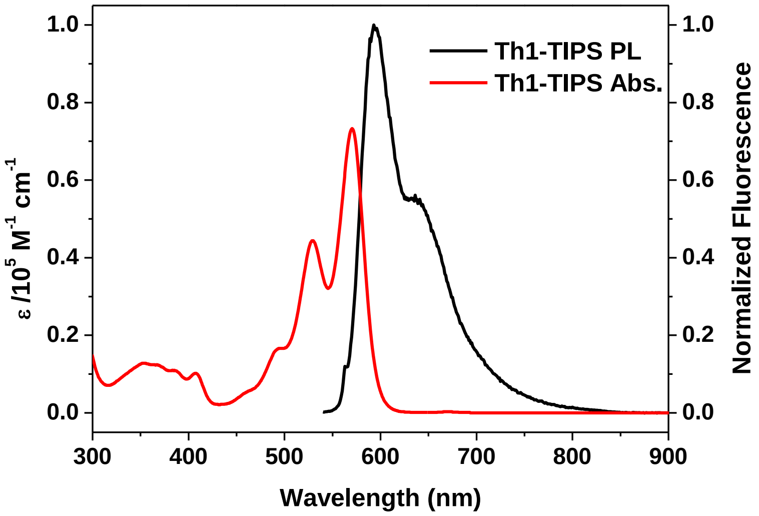

Fig. S1. UV-vis absorption and PL spectra of compound **Th1-TIPS** in DCM solution (10^{-5} M for UV-vis absorption measurements and 10^{-6} M for PL measurements).

3. Transient absorption decay curves

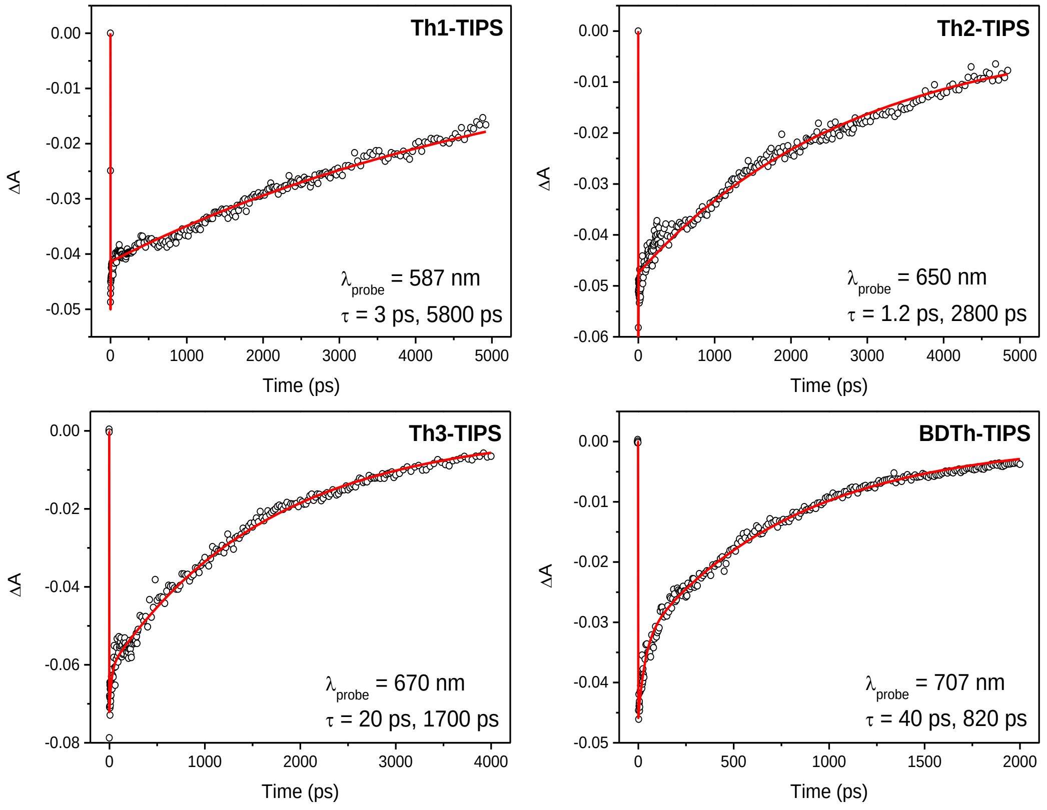

Fig. S2. TA decay curves of all compounds in toluene.

4. Z-scan curves

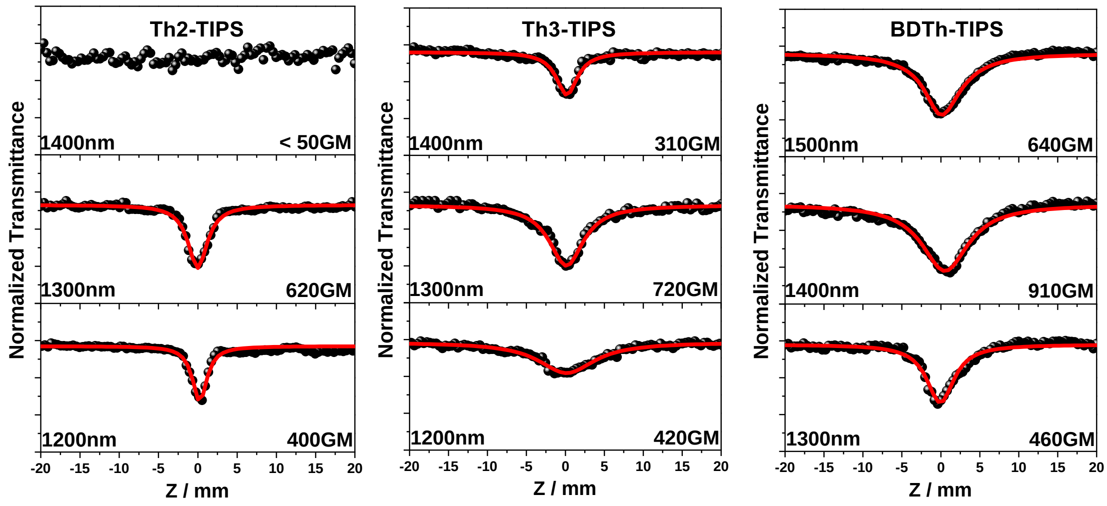

Fig. S3. Z-scan curves by photoexcitation in the range of 1200-1500 nm. **Th1-TIPS** was not recorded due to its short absorption wavelength and it is out of our TPA measuring range (1200-2400 nm).

5. DFT calculation details

Density functional theory calculations were employed with Gaussian 09 package,[1] utilizing the UCAM-B3LYP[2-4] level of theory with Pople basis set 6-31G*[5-7] for all molecules in the gas phase. NICS values at the UCAM-B3LYP/6-31G* level using the standard GIAO procedure (NMR pop=NCSall).[8] The calculated NICS data are shown below. The calculated energies and Cartesian coordinates of the closed-shell singlet, singlet diradical and triplet biradical of all molecules are attached as appendix.

Compound: **Th1-TIPS**

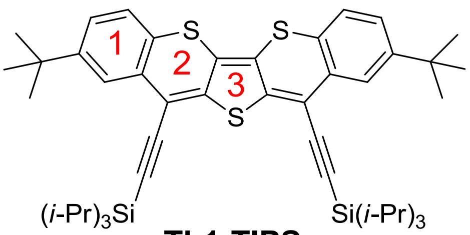

Th1-TIPS

Bq distance (Å)	NICS values (sigma zz)		
	ring 1	ring 2	ring 3
0.000	-4.059	30.810	23.477
0.100	-4.558	30.447	22.841
0.200	-6.010	29.319	20.998
0.300	-8.207	27.516	18.189
0.400	-10.853	25.174	14.754
0.500	-13.628	22.457	11.059
0.600	-16.238	19.535	7.430
0.700	-18.462	16.566	4.114
0.800	-20.165	13.681	1.264
0.900	-21.294	10.978	-1.056
1.000	-21.865	8.521	-2.849
1.100	-21.938	6.345	-4.163
1.200	-21.599	4.458	-5.067
1.300	-20.941	2.850	-5.641
1.400	-20.054	1.503	-5.957
1.500	-19.016	0.388	-6.081
1.600	-17.894	-0.522	-6.068
1.700	-16.738	-1.257	-5.961
1.800	-15.589	-1.842	-5.792
1.900	-14.472	-2.303	-5.587
2.000	-13.408	-2.659	-5.362
2.100	-12.407	-2.930	-5.131
2.200	-11.476	-3.130	-4.901

2.300	-10.617	-3.273	-4.679
2.400	-9.829	-3.370	-4.467
2.500	-9.109	-3.429	-4.267
2.600	-8.453	-3.458	-4.079
2.700	-7.858	-3.463	-3.905
2.800	-7.317	-3.449	-3.744
2.900	-6.827	-3.420	-3.594
3.000	-6.383	-3.379	-3.456
3.100	-5.979	-3.329	-3.329
3.200	-5.613	-3.273	-3.211
3.300	-5.280	-3.212	-3.102
3.400	-4.976	-3.147	-3.001
3.500	-4.699	-3.080	-2.907
3.600	-4.446	-3.012	-2.820
3.700	-4.214	-2.943	-2.740
3.800	-4.001	-2.874	-2.664
3.900	-3.805	-2.806	-2.594
4.000	-3.625	-2.739	-2.528
4.100	-3.458	-2.672	-2.466
4.200	-3.303	-2.608	-2.408
4.300	-3.159	-2.544	-2.353
4.400	-3.026	-2.483	-2.301
4.500	-2.902	-2.423	-2.252
4.600	-2.786	-2.365	-2.205
4.700	-2.677	-2.308	-2.160
4.800	-2.576	-2.253	-2.118
4.900	-2.480	-2.201	-2.077
5.000	-2.390	-2.149	-2.038

Compound: **Th2-TIPS**

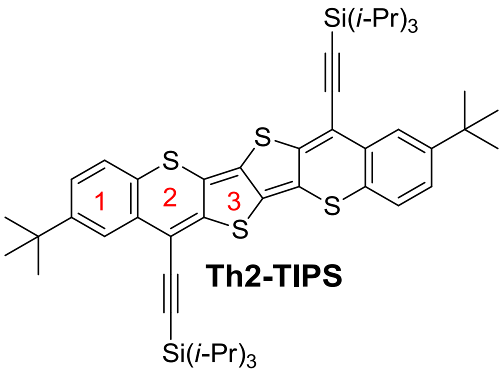

Th2-TIPS

Bq distance (Å)   NICS values (sigma zz)

	ring 1	ring 2	ring 3
0.000	-3.460	31.562	21.790
0.100	-3.966	31.161	21.205
0.200	-5.427	30.011	19.468
0.300	-7.637	28.202	16.798
0.400	-10.298	25.865	13.501
0.500	-13.088	23.158	9.918
0.600	-15.717	20.247	6.359
0.700	-17.961	17.284	3.067
0.800	-19.685	14.397	0.202
0.900	-20.836	11.684	-2.159
1.000	-21.429	9.208	-4.004
1.100	-21.525	7.005	-5.372
1.200	-21.209	5.085	-6.322
1.300	-20.572	3.441	-6.928
1.400	-19.706	2.054	-7.262
1.500	-18.687	0.899	-7.390
1.600	-17.583	-0.051	-7.365
1.700	-16.444	-0.825	-7.234
1.800	-15.310	-1.447	-7.032
1.900	-14.208	-1.942	-6.784
2.000	-13.158	-2.331	-6.511
2.100	-12.170	-2.632	-6.227
2.200	-11.250	-2.859	-5.941
2.300	-10.402	-3.027	-5.661
2.400	-9.624	-3.147	-5.391
2.500	-8.914	-3.226	-5.132
2.600	-8.267	-3.274	-4.888
2.700	-7.681	-3.295	-4.659
2.800	-7.149	-3.296	-4.443
2.900	-6.667	-3.280	-4.242
3.000	-6.231	-3.252	-4.055
3.100	-5.835	-3.213	-3.881
3.200	-5.476	-3.166	-3.718
3.300	-5.150	-3.113	-3.567
3.400	-4.853	-3.056	-3.426
3.500	-4.583	-2.996	-3.295
3.600	-4.336	-2.934	-3.173
3.700	-4.110	-2.870	-3.059
3.800	-3.903	-2.806	-2.952
3.900	-3.713	-2.742	-2.852
4.000	-3.537	-2.678	-2.759
4.100	-3.375	-2.615	-2.671

4.200	-3.225	-2.553	-2.589
4.300	-3.086	-2.492	-2.512
4.400	-2.957	-2.432	-2.439
4.500	-2.837	-2.374	-2.370
4.600	-2.725	-2.317	-2.305
4.700	-2.619	-2.262	-2.244
4.800	-2.521	-2.209	-2.186
4.900	-2.429	-2.157	-2.131
5.000	-2.342	-2.106	-2.078

Compound: **Th3-TIPS**

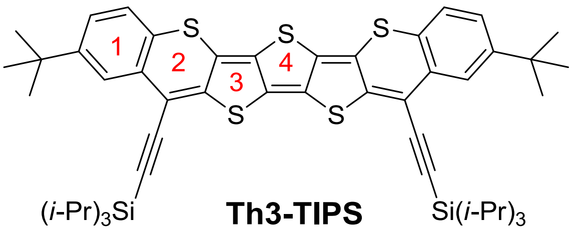

Th3-TIPS

Bq distance (Å)	NICS values (sigma zz)			
	ring 1	ring 2	ring 3	ring 4
0.000	-3.110	31.659	18.745	18.371
0.100	-3.634	31.269	18.120	17.770
0.200	-5.112	30.126	16.349	16.039
0.300	-7.335	28.317	13.655	13.386
0.400	-10.008	25.975	10.350	10.106
0.500	-12.809	23.260	6.778	6.532
0.600	-15.447	20.336	3.254	2.971
0.700	-17.701	17.358	0.024	-0.329
0.800	-19.437	14.454	-2.753	-3.202
0.900	-20.601	11.723	-5.002	-5.562
1.000	-21.209	9.229	-6.715	-7.390
1.100	-21.321	7.008	-7.933	-8.720
1.200	-21.022	5.070	-8.726	-9.611
1.300	-20.404	3.409	-9.170	-10.139
1.400	-19.555	2.007	-9.343	-10.377
1.500	-18.555	0.839	-9.313	-10.395
1.600	-17.467	-0.124	-9.140	-10.252
1.700	-16.345	-0.908	-8.869	-9.994
1.800	-15.226	-1.541	-8.536	-9.661
1.900	-14.138	-2.044	-8.169	-9.281
2.000	-13.100	-2.441	-7.787	-8.876
2.100	-12.123	-2.748	-7.404	-8.461

2.200	-11.215	-2.982	-7.028	-8.048
2.300	-10.376	-3.155	-6.667	-7.645
2.400	-9.606	-3.278	-6.324	-7.255
2.500	-8.903	-3.361	-6.000	-6.883
2.600	-8.263	-3.411	-5.697	-6.531
2.700	-7.682	-3.434	-5.413	-6.198
2.800	-7.155	-3.436	-5.150	-5.885
2.900	-6.678	-3.421	-4.905	-5.591
3.000	-6.245	-3.393	-4.678	-5.317
3.100	-5.853	-3.354	-4.467	-5.060
3.200	-5.497	-3.307	-4.271	-4.820
3.300	-5.174	-3.253	-4.089	-4.596
3.400	-4.879	-3.195	-3.919	-4.386
3.500	-4.611	-3.134	-3.762	-4.191
3.600	-4.366	-3.070	-3.615	-4.008
3.700	-4.142	-3.004	-3.477	-3.837
3.800	-3.937	-2.938	-3.349	-3.677
3.900	-3.748	-2.872	-3.229	-3.527
4.000	-3.574	-2.805	-3.116	-3.387
4.100	-3.413	-2.740	-3.011	-3.256
4.200	-3.264	-2.675	-2.911	-3.132
4.300	-3.126	-2.611	-2.818	-3.016
4.400	-2.998	-2.549	-2.729	-2.907
4.500	-2.879	-2.488	-2.646	-2.804
4.600	-2.767	-2.428	-2.567	-2.708
4.700	-2.663	-2.370	-2.492	-2.616
4.800	-2.566	-2.313	-2.421	-2.530
4.900	-2.474	-2.258	-2.354	-2.449
5.000	-2.388	-2.205	-2.290	-2.372

Compound: **BDTh-TIPS**

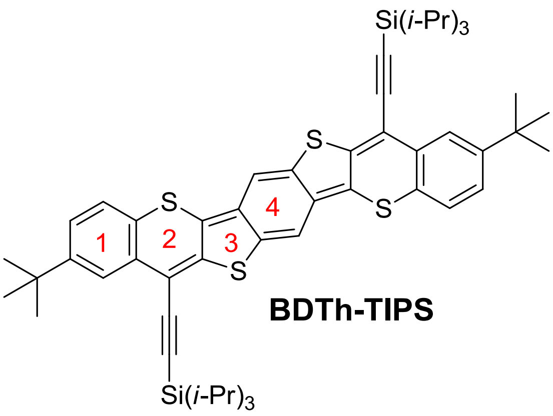

BDTh-TIPS

Bq distance (Å)	NICS values (sigma zz)			
	ring 1	ring 2	ring 3	ring 4
0.0000	-3.1532	30.7479	18.2450	0.4259
0.1000	-3.6792	30.3590	17.5834	-0.0547
0.2000	-5.1604	29.1925	15.7380	-1.4381
0.3000	-7.3870	27.3421	12.9418	-3.5385
0.4000	-10.0630	24.9491	9.5227	-6.0918
0.5000	-12.8665	22.1817	5.8378	-8.8084
0.6000	-15.5067	19.2138	2.2123	-11.4229
0.7000	-17.7624	16.2053	-1.1014	-13.7289
0.8000	-19.4988	13.2887	-3.9416	-15.5947
0.9000	-20.6641	10.5625	-6.2337	-16.9620
1.0000	-21.2732	8.0904	-7.9735	-17.8326
1.1000	-21.3864	5.9047	-9.2046	-18.2505
1.2000	-21.0883	4.0131	-9.9978	-18.2836
1.3000	-20.4713	2.4057	-10.4336	-18.0090
1.4000	-19.6243	1.0609	-10.5907	-17.5029
1.5000	-18.6255	-0.0486	-10.5395	-16.8337
1.6000	-17.5402	-0.9525	-10.3393	-16.0595
1.7000	-16.4195	-1.6800	-10.0376	-15.2269
1.8000	-15.3023	-2.2580	-9.6710	-14.3717
1.9000	-14.2161	-2.7112	-9.2674	-13.5204
2.0000	-13.1797	-3.0606	-8.8467	-12.6916
2.1000	-12.2045	-3.3246	-8.4234	-11.8976
2.2000	-11.2969	-3.5188	-8.0073	-11.1460
2.3000	-10.4589	-3.6559	-7.6049	-10.4408
2.4000	-9.6901	-3.7469	-7.2202	-9.7832
2.5000	-8.9879	-3.8006	-6.8554	-9.1729
2.6000	-8.3486	-3.8244	-6.5117	-8.6084
2.7000	-7.7679	-3.8243	-6.1891	-8.0872
2.8000	-7.2412	-3.8051	-5.8873	-7.6067
2.9000	-6.7636	-3.7709	-5.6053	-7.1639
3.0000	-6.3307	-3.7250	-5.3422	-6.7560
3.1000	-5.9380	-3.6701	-5.0969	-6.3801
3.2000	-5.5815	-3.6083	-4.8681	-6.0335
3.3000	-5.2575	-3.5414	-4.6547	-5.7137
3.4000	-4.9624	-3.4709	-4.4555	-5.4183
3.5000	-4.6934	-3.3980	-4.2695	-5.1451
3.6000	-4.4476	-3.3236	-4.0957	-4.8923
3.7000	-4.2224	-3.2484	-3.9331	-4.6579
3.8000	-4.0159	-3.1731	-3.7807	-4.4405
3.9000	-3.8259	-3.0981	-3.6379	-4.2385
4.0000	-3.6508	-3.0239	-3.5038	-4.0505

4.1000	-3.4890	-2.9508	-3.3778	-3.8755
4.2000	-3.3392	-2.8789	-3.2592	-3.7122
4.3000	-3.2001	-2.8085	-3.1474	-3.5597
4.4000	-3.0708	-2.7396	-3.0421	-3.4172
4.5000	-2.9503	-2.6725	-2.9426	-3.2837
4.6000	-2.8377	-2.6070	-2.8485	-3.1587
4.7000	-2.7323	-2.5433	-2.7594	-3.0413
4.8000	-2.6335	-2.4814	-2.6750	-2.9311
4.9000	-2.5407	-2.4213	-2.5949	-2.8274
5.0000	-2.4533	-2.3630	-2.5189	-2.7297

References:

1. *Gaussian 09; Revision A.2*; Frisch, M. J.; Trucks, G. W.; Schlegel, H. B.; Scuseria, G. E.; Robb, M. A.; Cheeseman, J. R.; Scalmani, G.; Barone, V.; Mennucci, B.; Petersson, G. A.; Nakatsuji, H.; Caricato, M.; Li, X.; Hratchian, H. P.; Izmaylov, A. F.; Bloino, J.; Zheng, G.; Sonnenberg, J. L.; Hada, M.; Ehara, M.; Toyota, K.; Fukuda, R.; Hasegawa, J.; Ishida, M.; Nakajima, T.; Honda, Y.; Kitao, O.; Nakai, H.; Vreven, T.; Montgomery, J., J. A.; Peralta, J. E.; Ogliaro, F.; Bearpark, M.; Heyd, J. J.; Brothers, E.; Kudin, K. N.; Staroverov, V. N.; Kobayashi, R.; Normand, J.; Raghavachari, K.; Rendell, A.; Burant, J. C.; Iyengar, S. S.; Tomasi, J.; Cossi, M.; Rega, N.; Millam, N. J.; Klene, M.; Knox, J. E.; Cross, J. B.; Bakken, V.; Adamo, C.; Jaramillo, J.; Gomperts, R.; Stratmann, R. E.; Yazyev, O.; Austin, A. J.; Cammi, R.; Pomelli, C.; Ochterski, J. W.; Martin, R. L.; Morokuma, K.; Zakrzewski, V. G.; Voth, G. A.; Salvador, P.; Dannenberg, J. J.; Dapprich, S.; Daniels, A. D.; Farkas, Ö.; Foresman, J. B.; Ortiz, J. V.; Cioslowski, J.; Fox, D. J.; Gaussian, Inc., Wallingford CT, 2009.
2. Becke, A. D. *J. Chem. Phys.* **1993**, *98*, 5648.
3. Lee, C.; Yang, W.; Parr, R. G. *Phys. Rev. B: Condens. Matter* **1988**, *37*, 785.
4. Yanai, T.; Tew, D.; and Handy, N. *Chem. Phys. Lett.* **2004**, *393*, 51.
5. Ditchfield, R.; Hehre, W. J.; Pople, J. A. *J. Chem. Phys.* **1971**, *54*, 724.
6. Hehre, W. J.; Ditchfield R.; Pople, J. A. *J. Chem. Phys.* **1972**, *56*, 2257.
7. Hariharan, P. C.; Pople, J. A. *Theor. Chim. Acta* **1973**, *28*, 213.
8. Fallah-Bagher-Shaidaei, H.; Wannere, S. S.; Corminboeuf, C.; Puchta, R.; Schleyer, P. V. R. *Org. Lett.* **2006**, *8*, 863.

6. Crystallographic data

Table S1. Crystal data and structure refinement for **Th1-TIPS**.

Empirical formula	C48 H66 S3 Si2	
Formula weight	795.36	
Temperature	100(2) K	
Wavelength	0.71073 Å	
Crystal system	Triclinic	
Space group	P-1	
Unit cell dimensions	a = 9.8336(6) Å	a= 83.637(2)°.
	b = 14.2701(9) Å	b= 85.505(2)°.
	c = 16.3902(10) Å	g = 83.306(2)°.
Volume	2265.2(2) $Å^3$	
Z	2	
Density (calculated)	1.166 Mg/m^3	
Absorption coefficient	0.248 mm^{-1}	
F(000)	860	
Crystal size	0.530 x 0.390 x 0.060 mm^3	
Theta range for data collection	2.364 to 28.282°.	
Index ranges	-13<=h<=13, -19<=k<=18, -21<=l<=19	
Reflections collected	48687	
Independent reflections	11236 [R(int) = 0.0614]	
Completeness to theta = 25.242°	99.9 %	
Absorption correction	Semi-empirical from equivalents	
Max. and min. transmission	0.7457 and 0.5761	
Refinement method	Full-matrix least-squares on F^2	
Data / restraints / parameters	11236 / 0 / 496	
Goodness-of-fit on F^2	1.040	
Final R indices [I>2sigma(I)]	R1 = 0.0591, wR2 = 0.1306	
R indices (all data)	R1 = 0.0888, wR2 = 0.1444	
Extinction coefficient	n/a	
Largest diff. peak and hole	1.451 and -0.524 e.$Å^{-3}$	

Table S2. Atomic coordinates (x 10^4) and equivalent isotropic displacement parameters ($Å^2$x 10^3) for **Th1-TIPS**. U(eq) is defined as one third of the trace of the orthogonalized U^{ij} tensor.

	x	y	z	U(eq)
Si(1)	7355(1)	9418(1)	6709(1)	23(1)
Si(2)	7956(1)	7291(1)	2498(1)	20(1)
S(1)	5028(1)	7097(1)	5226(1)	21(1)
S(2)	1728(1)	6100(1)	6845(1)	29(1)
S(3)	2083(1)	5123(1)	5134(1)	21(1)
C(1)	4190(2)	6278(2)	4784(1)	20(1)
C(2)	3115(2)	5962(2)	5343(1)	19(1)

C(3)	2970(2)	6368(2)	6066(1)	20(1)
C(4)	3931(2)	7028(2)	6122(1)	21(1)
C(5)	3994(2)	7547(2)	6775(2)	22(1)
C(6)	3032(2)	7448(2)	7510(1)	22(1)
C(7)	2017(2)	6832(2)	7593(2)	24(1)
C(8)	1161(3)	6749(2)	8309(2)	28(1)
C(9)	1312(3)	7262(2)	8956(2)	29(1)
C(10)	2314(2)	7882(2)	8900(2)	24(1)
C(11)	3141(2)	7973(2)	8176(2)	24(1)
C(12)	4514(2)	6014(2)	4004(1)	20(1)
C(13)	3714(2)	5359(2)	3660(1)	19(1)
C(14)	4030(2)	5135(2)	2849(1)	20(1)
C(15)	3269(2)	4571(2)	2465(1)	20(1)
C(16)	2148(2)	4215(2)	2925(2)	24(1)
C(17)	1818(2)	4408(2)	3725(2)	22(1)
C(18)	2601(2)	4974(2)	4101(1)	20(1)
C(19)	2508(3)	8403(2)	9650(2)	28(1)
C(20)	3613(3)	9088(2)	9466(2)	36(1)
C(21)	2937(3)	7657(2)	10355(2)	41(1)
C(22)	1156(3)	8971(2)	9912(2)	39(1)
C(23)	3572(2)	4373(2)	1564(1)	22(1)
C(24)	5004(3)	4605(2)	1235(2)	29(1)
C(25)	2505(3)	4988(2)	1043(2)	31(1)
C(26)	3463(3)	3328(2)	1472(2)	27(1)
C(27)	5024(2)	8172(2)	6751(1)	22(1)
C(28)	5909(2)	8694(2)	6748(2)	23(1)
C(29)	7410(3)	9864(2)	7745(2)	28(1)
C(30)	6083(3)	10495(2)	7954(2)	35(1)
C(31)	7684(3)	9066(2)	8433(2)	36(1)
C(32)	8934(3)	8573(2)	6498(2)	28(1)
C(33)	10273(3)	9025(2)	6518(2)	42(1)
C(34)	8926(3)	8085(2)	5716(2)	35(1)
C(35)	6964(3)	10464(2)	5920(2)	28(1)
C(36)	7912(3)	11248(2)	5909(2)	38(1)
C(37)	6864(3)	10194(2)	5058(2)	36(1)
C(38)	5594(2)	6415(2)	3507(1)	21(1)
C(39)	6505(2)	6765(2)	3085(2)	24(1)
C(40)	9546(2)	6739(2)	3013(2)	23(1)
C(41)	9572(3)	7010(2)	3886(2)	28(1)
C(42)	10881(3)	6932(2)	2509(2)	33(1)
C(43)	7586(2)	8618(2)	2486(2)	23(1)
C(44)	7140(3)	8968(2)	3322(2)	32(1)
C(45)	8754(3)	9151(2)	2067(2)	36(1)
C(46)	8023(3)	6970(2)	1412(2)	33(1)
C(47)	8321(3)	5896(2)	1370(2)	40(1)
C(48)	6695(4)	7351(3)	988(2)	61(1)

Table S3. Bond lengths [Å] and angles [°] for **Th1-TIPS**.

Si(1)-C(28)	1.847(2)
Si(1)-C(29)	1.885(2)
Si(1)-C(32)	1.886(3)
Si(1)-C(35)	1.889(3)
Si(2)-C(39)	1.845(3)
Si(2)-C(46)	1.881(3)
Si(2)-C(40)	1.884(2)
Si(2)-C(43)	1.885(2)
S(1)-C(4)	1.754(3)
S(1)-C(1)	1.755(2)
S(2)-C(3)	1.740(3)
S(2)-C(7)	1.755(2)
S(3)-C(2)	1.735(2)
S(3)-C(18)	1.758(2)
C(1)-C(12)	1.375(3)
C(1)-C(2)	1.424(3)
C(2)-C(3)	1.366(3)
C(3)-C(4)	1.423(3)
C(4)-C(5)	1.376(3)
C(5)-C(27)	1.422(3)
C(5)-C(6)	1.476(3)
C(6)-C(7)	1.394(3)
C(6)-C(11)	1.409(3)
C(7)-C(8)	1.391(4)
C(8)-C(9)	1.380(4)
C(8)-H(8)	0.9500
C(9)-C(10)	1.390(4)
C(9)-H(9)	0.9500
C(10)-C(11)	1.388(4)
C(10)-C(19)	1.540(3)
C(11)-H(11)	0.9500
C(12)-C(38)	1.422(3)
C(12)-C(13)	1.472(3)
C(13)-C(18)	1.396(3)
C(13)-C(14)	1.406(3)
C(14)-C(15)	1.389(3)
C(14)-H(14)	0.9500
C(15)-C(16)	1.399(3)
C(15)-C(23)	1.534(3)
C(16)-C(17)	1.376(3)
C(16)-H(16)	0.9500
C(17)-C(18)	1.398(3)
C(17)-H(17)	0.9500
C(19)-C(22)	1.531(4)
C(19)-C(21)	1.532(4)
C(19)-C(20)	1.535(4)
C(20)-H(20A)	0.9800

C(20)-H(20B)	0.9800
C(20)-H(20C)	0.9800
C(21)-H(21A)	0.9800
C(21)-H(21B)	0.9800
C(21)-H(21C)	0.9800
C(22)-H(22A)	0.9800
C(22)-H(22B)	0.9800
C(22)-H(22C)	0.9800
C(23)-C(24)	1.527(4)
C(23)-C(26)	1.533(3)
C(23)-C(25)	1.536(3)
C(24)-H(24A)	0.9800
C(24)-H(24B)	0.9800
C(24)-H(24C)	0.9800
C(25)-H(25A)	0.9800
C(25)-H(25B)	0.9800
C(25)-H(25C)	0.9800
C(26)-H(26A)	0.9800
C(26)-H(26B)	0.9800
C(26)-H(26C)	0.9800
C(27)-C(28)	1.208(3)
C(29)-C(31)	1.526(4)
C(29)-C(30)	1.535(4)
C(29)-H(29)	1.0000
C(30)-H(30A)	0.9800
C(30)-H(30B)	0.9800
C(30)-H(30C)	0.9800
C(31)-H(31A)	0.9800
C(31)-H(31B)	0.9800
C(31)-H(31C)	0.9800
C(32)-C(34)	1.527(4)
C(32)-C(33)	1.536(4)
C(32)-H(32)	1.0000
C(33)-H(33A)	0.9800
C(33)-H(33B)	0.9800
C(33)-H(33C)	0.9800
C(34)-H(34A)	0.9800
C(34)-H(34B)	0.9800
C(34)-H(34C)	0.9800
C(35)-C(37)	1.518(4)
C(35)-C(36)	1.535(4)
C(35)-H(35)	1.0000
C(36)-H(36A)	0.9800
C(36)-H(36B)	0.9800
C(36)-H(36C)	0.9800
C(37)-H(37A)	0.9800
C(37)-H(37B)	0.9800
C(37)-H(37C)	0.9800
C(38)-C(39)	1.208(3)

C(40)-C(41)	1.526(3)
C(40)-C(42)	1.532(4)
C(40)-H(40)	1.0000
C(41)-H(41A)	0.9800
C(41)-H(41B)	0.9800
C(41)-H(41C)	0.9800
C(42)-H(42A)	0.9800
C(42)-H(42B)	0.9800
C(42)-H(42C)	0.9800
C(43)-C(44)	1.525(3)
C(43)-C(45)	1.529(4)
C(43)-H(43)	1.0000
C(44)-H(44A)	0.9800
C(44)-H(44B)	0.9800
C(44)-H(44C)	0.9800
C(45)-H(45A)	0.9800
C(45)-H(45B)	0.9800
C(45)-H(45C)	0.9800
C(46)-C(47)	1.534(4)
C(46)-C(48)	1.542(4)
C(46)-H(46)	1.0000
C(47)-H(47A)	0.9800
C(47)-H(47B)	0.9800
C(47)-H(47C)	0.9800
C(48)-H(48A)	0.9800
C(48)-H(48B)	0.9800
C(48)-H(48C)	0.9800
C(28)-Si(1)-C(29)	108.71(11)
C(28)-Si(1)-C(32)	104.90(11)
C(29)-Si(1)-C(32)	109.88(11)
C(28)-Si(1)-C(35)	106.30(11)
C(29)-Si(1)-C(35)	108.90(12)
C(32)-Si(1)-C(35)	117.74(12)
C(39)-Si(2)-C(46)	108.33(12)
C(39)-Si(2)-C(40)	106.10(11)
C(46)-Si(2)-C(40)	110.33(12)
C(39)-Si(2)-C(43)	107.11(11)
C(46)-Si(2)-C(43)	108.87(12)
C(40)-Si(2)-C(43)	115.80(11)
C(4)-S(1)-C(1)	92.21(11)
C(3)-S(2)-C(7)	102.55(12)
C(2)-S(3)-C(18)	101.84(11)
C(12)-C(1)-C(2)	126.1(2)
C(12)-C(1)-S(1)	124.24(18)
C(2)-C(1)-S(1)	109.60(16)
C(3)-C(2)-C(1)	114.4(2)
C(3)-C(2)-S(3)	122.51(19)
C(1)-C(2)-S(3)	123.09(17)

C(2)-C(3)-C(4)	113.8(2)
C(2)-C(3)-S(2)	122.96(18)
C(4)-C(3)-S(2)	123.26(18)
C(5)-C(4)-C(3)	125.6(2)
C(5)-C(4)-S(1)	124.40(19)
C(3)-C(4)-S(1)	109.99(17)
C(4)-C(5)-C(27)	119.4(2)
C(4)-C(5)-C(6)	121.2(2)
C(27)-C(5)-C(6)	119.4(2)
C(7)-C(6)-C(11)	117.1(2)
C(7)-C(6)-C(5)	123.1(2)
C(11)-C(6)-C(5)	119.8(2)
C(8)-C(7)-C(6)	120.6(2)
C(8)-C(7)-S(2)	115.05(19)
C(6)-C(7)-S(2)	124.36(19)
C(9)-C(8)-C(7)	120.8(2)
C(9)-C(8)-H(8)	119.6
C(7)-C(8)-H(8)	119.6
C(8)-C(9)-C(10)	120.5(2)
C(8)-C(9)-H(9)	119.8
C(10)-C(9)-H(9)	119.8
C(11)-C(10)-C(9)	118.1(2)
C(11)-C(10)-C(19)	122.6(2)
C(9)-C(10)-C(19)	119.2(2)
C(10)-C(11)-C(6)	122.9(2)
C(10)-C(11)-H(11)	118.6
C(6)-C(11)-H(11)	118.6
C(1)-C(12)-C(38)	119.5(2)
C(1)-C(12)-C(13)	121.0(2)
C(38)-C(12)-C(13)	119.4(2)
C(18)-C(13)-C(14)	117.9(2)
C(18)-C(13)-C(12)	122.3(2)
C(14)-C(13)-C(12)	119.7(2)
C(15)-C(14)-C(13)	123.0(2)
C(15)-C(14)-H(14)	118.5
C(13)-C(14)-H(14)	118.5
C(14)-C(15)-C(16)	117.1(2)
C(14)-C(15)-C(23)	122.8(2)
C(16)-C(15)-C(23)	120.1(2)
C(17)-C(16)-C(15)	121.5(2)
C(17)-C(16)-H(16)	119.2
C(15)-C(16)-H(16)	119.2
C(16)-C(17)-C(18)	120.4(2)
C(16)-C(17)-H(17)	119.8
C(18)-C(17)-H(17)	119.8
C(13)-C(18)-C(17)	120.0(2)
C(13)-C(18)-S(3)	125.03(17)
C(17)-C(18)-S(3)	114.97(18)
C(22)-C(19)-C(21)	109.3(2)

C(22)-C(19)-C(20)	108.5(2)
C(21)-C(19)-C(20)	109.1(2)
C(22)-C(19)-C(10)	109.9(2)
C(21)-C(19)-C(10)	108.0(2)
C(20)-C(19)-C(10)	112.1(2)
C(19)-C(20)-H(20A)	109.5
C(19)-C(20)-H(20B)	109.5
H(20A)-C(20)-H(20B)	109.5
C(19)-C(20)-H(20C)	109.5
H(20A)-C(20)-H(20C)	109.5
H(20B)-C(20)-H(20C)	109.5
C(19)-C(21)-H(21A)	109.5
C(19)-C(21)-H(21B)	109.5
H(21A)-C(21)-H(21B)	109.5
C(19)-C(21)-H(21C)	109.5
H(21A)-C(21)-H(21C)	109.5
H(21B)-C(21)-H(21C)	109.5
C(19)-C(22)-H(22A)	109.5
C(19)-C(22)-H(22B)	109.5
H(22A)-C(22)-H(22B)	109.5
C(19)-C(22)-H(22C)	109.5
H(22A)-C(22)-H(22C)	109.5
H(22B)-C(22)-H(22C)	109.5
C(24)-C(23)-C(26)	108.5(2)
C(24)-C(23)-C(15)	111.93(19)
C(26)-C(23)-C(15)	110.5(2)
C(24)-C(23)-C(25)	108.8(2)
C(26)-C(23)-C(25)	108.56(19)
C(15)-C(23)-C(25)	108.48(19)
C(23)-C(24)-H(24A)	109.5
C(23)-C(24)-H(24B)	109.5
H(24A)-C(24)-H(24B)	109.5
C(23)-C(24)-H(24C)	109.5
H(24A)-C(24)-H(24C)	109.5
H(24B)-C(24)-H(24C)	109.5
C(23)-C(25)-H(25A)	109.5
C(23)-C(25)-H(25B)	109.5
H(25A)-C(25)-H(25B)	109.5
C(23)-C(25)-H(25C)	109.5
H(25A)-C(25)-H(25C)	109.5
H(25B)-C(25)-H(25C)	109.5
C(23)-C(26)-H(26A)	109.5
C(23)-C(26)-H(26B)	109.5
H(26A)-C(26)-H(26B)	109.5
C(23)-C(26)-H(26C)	109.5
H(26A)-C(26)-H(26C)	109.5
H(26B)-C(26)-H(26C)	109.5
C(28)-C(27)-C(5)	178.5(3)
C(27)-C(28)-Si(1)	175.5(2)

C(31)-C(29)-C(30)	110.7(2)
C(31)-C(29)-Si(1)	112.98(17)
C(30)-C(29)-Si(1)	110.57(17)
C(31)-C(29)-H(29)	107.4
C(30)-C(29)-H(29)	107.4
Si(1)-C(29)-H(29)	107.4
C(29)-C(30)-H(30A)	109.5
C(29)-C(30)-H(30B)	109.5
H(30A)-C(30)-H(30B)	109.5
C(29)-C(30)-H(30C)	109.5
H(30A)-C(30)-H(30C)	109.5
H(30B)-C(30)-H(30C)	109.5
C(29)-C(31)-H(31A)	109.5
C(29)-C(31)-H(31B)	109.5
H(31A)-C(31)-H(31B)	109.5
C(29)-C(31)-H(31C)	109.5
H(31A)-C(31)-H(31C)	109.5
H(31B)-C(31)-H(31C)	109.5
C(34)-C(32)-C(33)	110.9(2)
C(34)-C(32)-Si(1)	114.33(18)
C(33)-C(32)-Si(1)	112.87(18)
C(34)-C(32)-H(32)	106.0
C(33)-C(32)-H(32)	106.0
Si(1)-C(32)-H(32)	106.0
C(32)-C(33)-H(33A)	109.5
C(32)-C(33)-H(33B)	109.5
H(33A)-C(33)-H(33B)	109.5
C(32)-C(33)-H(33C)	109.5
H(33A)-C(33)-H(33C)	109.5
H(33B)-C(33)-H(33C)	109.5
C(32)-C(34)-H(34A)	109.5
C(32)-C(34)-H(34B)	109.5
H(34A)-C(34)-H(34B)	109.5
C(32)-C(34)-H(34C)	109.5
H(34A)-C(34)-H(34C)	109.5
H(34B)-C(34)-H(34C)	109.5
C(37)-C(35)-C(36)	110.9(2)
C(37)-C(35)-Si(1)	113.93(18)
C(36)-C(35)-Si(1)	114.23(18)
C(37)-C(35)-H(35)	105.6
C(36)-C(35)-H(35)	105.6
Si(1)-C(35)-H(35)	105.6
C(35)-C(36)-H(36A)	109.5
C(35)-C(36)-H(36B)	109.5
H(36A)-C(36)-H(36B)	109.5
C(35)-C(36)-H(36C)	109.5
H(36A)-C(36)-H(36C)	109.5
H(36B)-C(36)-H(36C)	109.5
C(35)-C(37)-H(37A)	109.5

C(35)-C(37)-H(37B)	109.5
H(37A)-C(37)-H(37B)	109.5
C(35)-C(37)-H(37C)	109.5
H(37A)-C(37)-H(37C)	109.5
H(37B)-C(37)-H(37C)	109.5
C(39)-C(38)-C(12)	179.3(3)
C(38)-C(39)-Si(2)	176.5(2)
C(41)-C(40)-C(42)	110.8(2)
C(41)-C(40)-Si(2)	112.52(17)
C(42)-C(40)-Si(2)	113.51(17)
C(41)-C(40)-H(40)	106.5
C(42)-C(40)-H(40)	106.5
Si(2)-C(40)-H(40)	106.5
C(40)-C(41)-H(41A)	109.5
C(40)-C(41)-H(41B)	109.5
H(41A)-C(41)-H(41B)	109.5
C(40)-C(41)-H(41C)	109.5
H(41A)-C(41)-H(41C)	109.5
H(41B)-C(41)-H(41C)	109.5
C(40)-C(42)-H(42A)	109.5
C(40)-C(42)-H(42B)	109.5
H(42A)-C(42)-H(42B)	109.5
C(40)-C(42)-H(42C)	109.5
H(42A)-C(42)-H(42C)	109.5
H(42B)-C(42)-H(42C)	109.5
C(44)-C(43)-C(45)	110.4(2)
C(44)-C(43)-Si(2)	114.91(17)
C(45)-C(43)-Si(2)	113.04(17)
C(44)-C(43)-H(43)	105.9
C(45)-C(43)-H(43)	105.9
Si(2)-C(43)-H(43)	105.9
C(43)-C(44)-H(44A)	109.5
C(43)-C(44)-H(44B)	109.5
H(44A)-C(44)-H(44B)	109.5
C(43)-C(44)-H(44C)	109.5
H(44A)-C(44)-H(44C)	109.5
H(44B)-C(44)-H(44C)	109.5
C(43)-C(45)-H(45A)	109.5
C(43)-C(45)-H(45B)	109.5
H(45A)-C(45)-H(45B)	109.5
C(43)-C(45)-H(45C)	109.5
H(45A)-C(45)-H(45C)	109.5
H(45B)-C(45)-H(45C)	109.5
C(47)-C(46)-C(48)	110.1(2)
C(47)-C(46)-Si(2)	111.95(19)
C(48)-C(46)-Si(2)	111.9(2)
C(47)-C(46)-H(46)	107.6
C(48)-C(46)-H(46)	107.6
Si(2)-C(46)-H(46)	107.6

C(46)-C(47)-H(47A)	109.5
C(46)-C(47)-H(47B)	109.5
H(47A)-C(47)-H(47B)	109.5
C(46)-C(47)-H(47C)	109.5
H(47A)-C(47)-H(47C)	109.5
H(47B)-C(47)-H(47C)	109.5
C(46)-C(48)-H(48A)	109.5
C(46)-C(48)-H(48B)	109.5
H(48A)-C(48)-H(48B)	109.5
C(46)-C(48)-H(48C)	109.5
H(48A)-C(48)-H(48C)	109.5
H(48B)-C(48)-H(48C)	109.5

Symmetry transformations used to generate equivalent atoms:

Table S4. Anisotropic displacement parameters ($Å^2$x 10^3) for **Th1-TIPS.** The anisotropic displacement factor exponent takes the form: $-2\pi^2[h^2 a^{*2}U^{11} + ... + 2 h k a^* b^* U^{12}]$

	U^{11}	U^{22}	U^{33}	U^{23}	U^{13}	U^{12}
Si(1)	20(1)	24(1)	26(1)	-10(1)	-4(1)	-4(1)
Si(2)	19(1)	22(1)	19(1)	-5(1)	-3(1)	-2(1)
S(1)	19(1)	22(1)	25(1)	-9(1)	-6(1)	-4(1)
S(2)	30(1)	36(1)	24(1)	-10(1)	0(1)	-12(1)
S(3)	22(1)	24(1)	20(1)	-6(1)	-4(1)	-7(1)
C(1)	18(1)	17(1)	26(1)	-5(1)	-10(1)	1(1)
C(2)	18(1)	19(1)	22(1)	-5(1)	-8(1)	0(1)
C(3)	20(1)	23(1)	19(1)	-6(1)	-6(1)	0(1)
C(4)	20(1)	22(1)	23(1)	-4(1)	-9(1)	1(1)
C(5)	20(1)	19(1)	28(1)	-5(1)	-11(1)	0(1)
C(6)	21(1)	24(1)	21(1)	-4(1)	-5(1)	5(1)
C(7)	23(1)	27(1)	25(1)	-8(1)	-6(1)	1(1)
C(8)	25(1)	33(1)	28(1)	-6(1)	1(1)	-7(1)
C(9)	27(1)	33(1)	27(1)	-7(1)	3(1)	-3(1)
C(10)	23(1)	25(1)	22(1)	-4(1)	-1(1)	6(1)
C(11)	19(1)	23(1)	29(1)	-4(1)	-7(1)	0(1)
C(12)	16(1)	20(1)	23(1)	-4(1)	-5(1)	-1(1)
C(13)	18(1)	16(1)	23(1)	-5(1)	-8(1)	2(1)
C(14)	17(1)	19(1)	23(1)	-4(1)	-5(1)	0(1)
C(15)	20(1)	20(1)	22(1)	-7(1)	-5(1)	1(1)
C(16)	24(1)	26(1)	27(1)	-10(1)	-7(1)	-5(1)
C(17)	19(1)	25(1)	25(1)	-8(1)	-3(1)	-5(1)
C(18)	20(1)	19(1)	20(1)	-5(1)	-6(1)	-1(1)
C(19)	32(1)	28(1)	25(1)	-6(1)	2(1)	-6(1)
C(20)	42(2)	42(2)	29(1)	-15(1)	4(1)	-15(1)
C(21)	56(2)	41(2)	26(1)	-3(1)	-6(1)	-3(1)
C(22)	40(2)	37(2)	38(2)	-13(1)	11(1)	-3(1)

C(23)	24(1)	23(1)	22(1)	-8(1)	-7(1)	0(1)
C(24)	29(1)	36(1)	23(1)	-10(1)	-2(1)	-3(1)
C(25)	34(1)	33(1)	26(1)	-5(1)	-9(1)	6(1)
C(26)	31(1)	25(1)	26(1)	-11(1)	-5(1)	2(1)
C(27)	24(1)	23(1)	21(1)	-7(1)	-6(1)	1(1)
C(28)	25(1)	23(1)	24(1)	-8(1)	-6(1)	-2(1)
C(29)	27(1)	29(1)	31(1)	-14(1)	-4(1)	-7(1)
C(30)	36(2)	35(2)	34(1)	-15(1)	-2(1)	1(1)
C(31)	43(2)	37(2)	31(1)	-13(1)	-9(1)	-1(1)
C(32)	26(1)	28(1)	31(1)	-7(1)	-4(1)	-3(1)
C(33)	22(1)	41(2)	65(2)	-15(2)	-1(1)	-2(1)
C(34)	37(2)	34(2)	34(2)	-14(1)	-2(1)	6(1)
C(35)	23(1)	29(1)	34(1)	-9(1)	-3(1)	-4(1)
C(36)	35(2)	33(2)	49(2)	-4(1)	-7(1)	-10(1)
C(37)	42(2)	34(2)	32(1)	-4(1)	-5(1)	-5(1)
C(38)	22(1)	21(1)	23(1)	-8(1)	-9(1)	0(1)
C(39)	22(1)	26(1)	27(1)	-8(1)	-7(1)	-5(1)
C(40)	22(1)	20(1)	26(1)	-4(1)	-5(1)	1(1)
C(41)	27(1)	29(1)	28(1)	-4(1)	-10(1)	-1(1)
C(42)	21(1)	38(2)	38(2)	-8(1)	-1(1)	4(1)
C(43)	22(1)	22(1)	26(1)	-4(1)	-5(1)	0(1)
C(44)	36(2)	27(1)	34(1)	-11(1)	0(1)	3(1)
C(45)	42(2)	24(1)	42(2)	-3(1)	7(1)	-6(1)
C(46)	42(2)	35(2)	24(1)	-11(1)	-5(1)	-2(1)
C(47)	47(2)	42(2)	37(2)	-21(1)	-7(1)	-2(1)
C(48)	86(3)	61(2)	40(2)	-21(2)	-39(2)	14(2)

Table S5. Hydrogen coordinates (x 10^4) and isotropic displacement parameters (Å^{2}x 10 ^{3}) for **Th1-TIPS**.

	x	y	z	U(eq)
H(8)	465	6334	8353	34
H(9)	727	7191	9443	35
H(11)	3810	8409	8127	28
H(14)	4801	5379	2549	24
H(16)	1602	3831	2679	29
H(17)	1054	4155	4024	27
H(20A)	3354	9559	9007	54
H(20B)	4495	8731	9319	54
H(20C)	3694	9411	9954	54
H(21A)	3045	7973	10844	61
H(21B)	3810	7300	10193	61
H(21C)	2230	7222	10480	61
H(22A)	902	9469	9472	58
H(22B)	1267	9260	10414	58

H(22C)	433	8546	10016	58
H(24A)	5050	5289	1220	44
H(24B)	5193	4412	678	44
H(24C)	5687	4264	1595	44
H(25A)	1585	4822	1233	47
H(25B)	2692	4874	464	47
H(25C)	2557	5659	1101	47
H(26A)	4069	2925	1845	41
H(26B)	3735	3200	903	41
H(26C)	2513	3190	1608	41
H(29)	8183	10267	7707	33
H(30A)	5294	10139	7940	52
H(30B)	6000	11057	7551	52
H(30C)	6109	10694	8506	52
H(31A)	7714	9338	8954	54
H(31B)	8566	8698	8306	54
H(31C)	6950	8651	8480	54
H(32)	8942	8057	6964	34
H(33A)	11058	8555	6414	63
H(33B)	10321	9246	7060	63
H(33C)	10294	9565	6093	63
H(34A)	9003	8553	5235	53
H(34B)	8066	7800	5718	53
H(34C)	9702	7590	5693	53
H(35)	6027	10758	6092	34
H(36A)	8854	11000	5748	58
H(36B)	7879	11462	6459	58
H(36C)	7606	11784	5514	58
H(37A)	6473	10747	4710	54
H(37B)	6273	9683	5081	54
H(37C)	7782	9978	4827	54
H(40)	9513	6037	3063	27
H(41A)	10331	6627	4160	42
H(41B)	8703	6894	4195	42
H(41C)	9699	7684	3865	42
H(42A)	10975	7612	2460	49
H(42B)	10861	6726	1959	49
H(42C)	11662	6580	2785	49
H(43)	6787	8805	2139	28
H(44A)	7928	8885	3664	48
H(44B)	6417	8602	3593	48
H(44C)	6788	9641	3247	48
H(45A)	8453	9833	2003	55
H(45B)	9010	8932	1524	55
H(45C)	9549	9030	2404	55
H(46)	8795	7276	1095	40
H(47A)	7582	5576	1678	61
H(47B)	9195	5667	1611	61
H(47C)	8375	5760	795	61

H(48A)	6757	7154	431	92
H(48B)	6573	8046	957	92
H(48C)	5909	7097	1306	92

Table S6. Torsion angles [°] for **Th1-TIPS**.

C(4)-S(1)-C(1)-C(12)	177.0(2)
C(4)-S(1)-C(1)-C(2)	-1.26(17)
C(12)-C(1)-C(2)-C(3)	-177.1(2)
S(1)-C(1)-C(2)-C(3)	1.2(2)
C(12)-C(1)-C(2)-S(3)	3.5(3)
S(1)-C(1)-C(2)-S(3)	-178.22(12)
C(18)-S(3)-C(2)-C(3)	173.46(19)
C(18)-S(3)-C(2)-C(1)	-7.2(2)
C(1)-C(2)-C(3)-C(4)	-0.4(3)
S(3)-C(2)-C(3)-C(4)	179.02(16)
C(1)-C(2)-C(3)-S(2)	179.80(16)
S(3)-C(2)-C(3)-S(2)	-0.8(3)
C(7)-S(2)-C(3)-C(2)	-179.2(2)
C(7)-S(2)-C(3)-C(4)	1.0(2)
C(2)-C(3)-C(4)-C(5)	178.8(2)
S(2)-C(3)-C(4)-C(5)	-1.4(3)
C(2)-C(3)-C(4)-S(1)	-0.6(3)
S(2)-C(3)-C(4)-S(1)	179.23(13)
C(1)-S(1)-C(4)-C(5)	-178.3(2)
C(1)-S(1)-C(4)-C(3)	1.07(17)
C(3)-C(4)-C(5)-C(27)	179.2(2)
S(1)-C(4)-C(5)-C(27)	-1.5(3)
C(3)-C(4)-C(5)-C(6)	0.7(3)
S(1)-C(4)-C(5)-C(6)	-179.97(17)
C(4)-C(5)-C(6)-C(7)	0.2(3)
C(27)-C(5)-C(6)-C(7)	-178.2(2)
C(4)-C(5)-C(6)-C(11)	178.6(2)
C(27)-C(5)-C(6)-C(11)	0.2(3)
C(11)-C(6)-C(7)-C(8)	-0.1(3)
C(5)-C(6)-C(7)-C(8)	178.3(2)
C(11)-C(6)-C(7)-S(2)	-178.90(17)
C(5)-C(6)-C(7)-S(2)	-0.5(3)
C(3)-S(2)-C(7)-C(8)	-178.91(19)
C(3)-S(2)-C(7)-C(6)	-0.1(2)
C(6)-C(7)-C(8)-C(9)	-1.0(4)
S(2)-C(7)-C(8)-C(9)	177.9(2)
C(7)-C(8)-C(9)-C(10)	0.8(4)
C(8)-C(9)-C(10)-C(11)	0.6(4)
C(8)-C(9)-C(10)-C(19)	-176.7(2)
C(9)-C(10)-C(11)-C(6)	-1.8(3)
C(19)-C(10)-C(11)-C(6)	175.3(2)
C(7)-C(6)-C(11)-C(10)	1.6(3)

C(5)-C(6)-C(11)-C(10)	-176.9(2)
C(2)-C(1)-C(12)-C(38)	178.0(2)
S(1)-C(1)-C(12)-C(38)	0.1(3)
C(2)-C(1)-C(12)-C(13)	1.3(3)
S(1)-C(1)-C(12)-C(13)	-176.73(16)
C(1)-C(12)-C(13)-C(18)	-0.3(3)
C(38)-C(12)-C(13)-C(18)	-177.1(2)
C(1)-C(12)-C(13)-C(14)	177.2(2)
C(38)-C(12)-C(13)-C(14)	0.4(3)
C(18)-C(13)-C(14)-C(15)	1.7(3)
C(12)-C(13)-C(14)-C(15)	-175.8(2)
C(13)-C(14)-C(15)-C(16)	-0.6(3)
C(13)-C(14)-C(15)-C(23)	176.6(2)
C(14)-C(15)-C(16)-C(17)	-0.4(3)
C(23)-C(15)-C(16)-C(17)	-177.7(2)
C(15)-C(16)-C(17)-C(18)	0.2(4)
C(14)-C(13)-C(18)-C(17)	-1.9(3)
C(12)-C(13)-C(18)-C(17)	175.6(2)
C(14)-C(13)-C(18)-S(3)	177.02(17)
C(12)-C(13)-C(18)-S(3)	-5.5(3)
C(16)-C(17)-C(18)-C(13)	1.0(3)
C(16)-C(17)-C(18)-S(3)	-178.04(19)
C(2)-S(3)-C(18)-C(13)	8.3(2)
C(2)-S(3)-C(18)-C(17)	-172.74(18)
C(11)-C(10)-C(19)-C(22)	126.0(3)
C(9)-C(10)-C(19)-C(22)	-56.9(3)
C(11)-C(10)-C(19)-C(21)	-114.9(3)
C(9)-C(10)-C(19)-C(21)	62.2(3)
C(11)-C(10)-C(19)-C(20)	5.3(3)
C(9)-C(10)-C(19)-C(20)	-177.6(2)
C(14)-C(15)-C(23)-C(24)	16.8(3)
C(16)-C(15)-C(23)-C(24)	-166.1(2)
C(14)-C(15)-C(23)-C(26)	137.8(2)
C(16)-C(15)-C(23)-C(26)	-45.0(3)
C(14)-C(15)-C(23)-C(25)	-103.3(3)
C(16)-C(15)-C(23)-C(25)	73.9(3)
C(28)-Si(1)-C(29)-C(31)	63.1(2)
C(32)-Si(1)-C(29)-C(31)	-51.2(2)
C(35)-Si(1)-C(29)-C(31)	178.53(19)
C(28)-Si(1)-C(29)-C(30)	-61.6(2)
C(32)-Si(1)-C(29)-C(30)	-175.88(18)
C(35)-Si(1)-C(29)-C(30)	53.8(2)
C(28)-Si(1)-C(32)-C(34)	57.0(2)
C(29)-Si(1)-C(32)-C(34)	173.74(19)
C(35)-Si(1)-C(32)-C(34)	-60.9(2)
C(28)-Si(1)-C(32)-C(33)	-175.0(2)
C(29)-Si(1)-C(32)-C(33)	-58.3(2)
C(35)-Si(1)-C(32)-C(33)	67.1(2)
C(28)-Si(1)-C(35)-C(37)	-61.9(2)

C(29)-Si(1)-C(35)-C(37)	-178.84(19)
C(32)-Si(1)-C(35)-C(37)	55.3(2)
C(28)-Si(1)-C(35)-C(36)	169.3(2)
C(29)-Si(1)-C(35)-C(36)	52.3(2)
C(32)-Si(1)-C(35)-C(36)	-73.6(2)
C(39)-Si(2)-C(40)-C(41)	-65.19(19)
C(46)-Si(2)-C(40)-C(41)	177.68(18)
C(43)-Si(2)-C(40)-C(41)	53.5(2)
C(39)-Si(2)-C(40)-C(42)	168.01(18)
C(46)-Si(2)-C(40)-C(42)	50.9(2)
C(43)-Si(2)-C(40)-C(42)	-73.3(2)
C(39)-Si(2)-C(43)-C(44)	48.1(2)
C(46)-Si(2)-C(43)-C(44)	165.05(19)
C(40)-Si(2)-C(43)-C(44)	-70.0(2)
C(39)-Si(2)-C(43)-C(45)	176.15(18)
C(46)-Si(2)-C(43)-C(45)	-66.9(2)
C(40)-Si(2)-C(43)-C(45)	58.0(2)
C(39)-Si(2)-C(46)-C(47)	-63.8(2)
C(40)-Si(2)-C(46)-C(47)	52.0(2)
C(43)-Si(2)-C(46)-C(47)	-179.9(2)
C(39)-Si(2)-C(46)-C(48)	60.3(3)
C(40)-Si(2)-C(46)-C(48)	176.1(2)
C(43)-Si(2)-C(46)-C(48)	-55.8(3)

Symmetry transformations used to generate equivalent atoms:

Table S7. Crystal data and structure refinement for **Th2-TIPS**.

Empirical formula	$C_{50}H_{66}S_4Si_2$	
Formula weight	851.44	
Temperature	100(2) K	
Wavelength	1.54178 Å	
Crystal system	Triclinic	
Space group	P -1	
Unit cell dimensions	a = 7.4217(4) Å	a = 63.238(2)°
	b = 19.0953(11) Å	b = 89.851(3)°
	c = 19.1877(16) Å	g = 79.162(2)°
Volume	2374.2(3) $Å^3$	
Z	2	
Density (calculated)	1.191 Mg/m^3	
Absorption coefficient	2.557 mm^{-1}	
F(000)	916	
Crystal size	0.240 x 0.123 x 0.098 mm^3	

Theta range for data collection	2.590 to 68.234°
Index ranges	-8<=h<=8, -21<=k<=22, -21<=l<=22
Reflections collected	20701
Independent reflections	7400 [R(int) = 0.2482]
Absorption correction	Semi-empirical from equivalents
Max. and min. transmission	0.788 and 0.579
Refinement method	Full-matrix least-squares on F^2
Data / restraints / parameters	7400 / 54 / 556
Goodness-of-fit on F^2	1.561
Final R indices [I>2sigma(I)]	R1 = 0.0922, wR2 = 0.2629
R indices (all data)	R1 = 0.1101, wR2 = 0.3472
Largest diff. peak and hole	1.123 and -1.223 e.$Å^{-3}$

Table S8. Atomic coordinates (x 10^4) and equivalent isotropic displacement parameters ($Å^2$x 10^3) for **Th2-TIPS**. U(eq) is defined as one third of the trace of the orthogonalized U^{ij} tensor.

	x	y	z	U(eq)
S(1A)	8809(2)	6002(1)	157(1)	18(1)
S(2A)	10050(2)	3480(1)	1804(1)	22(1)
Si(1A)	5063(3)	7374(1)	1760(1)	23(1)
C(1A)	8940(9)	5147(3)	1076(3)	20(1)
C(2A)	9779(8)	4426(3)	1005(3)	14(1)
C(3A)	9171(8)	3703(4)	2558(3)	18(1)
C(4A)	9200(11)	3015(4)	3293(4)	32(2)
C(5A)	8721(11)	3114(4)	3941(4)	33(2)
C(6A)	8207(11)	3871(4)	3906(4)	30(2)
C(7A)	8070(10)	4532(4)	3169(4)	28(2)
C(8A)	8551(10)	4461(4)	2491(3)	26(2)
C(9A)	8353(9)	5186(3)	1720(3)	19(1)
C(10A)	7460(9)	5928(3)	1707(3)	22(1)
C(11A)	6663(10)	6535(4)	1718(3)	23(1)
C(12A)	9786(9)	5418(3)	-271(4)	19(1)
C(13A)	7824(9)	3998(4)	4631(3)	32(2)
C(14A)	9541(15)	3627(8)	5216(7)	55(5)
C(15A)	7330(20)	4886(5)	4437(6)	61(6)
C(16A)	6254(17)	3615(8)	5047(7)	51(5)

C(14C)	9150(30)	4404(12)	4845(15)	80(10)
C(15C)	5869(16)	4480(12)	4521(14)	60(7)
C(16C)	7930(30)	3166(7)	5316(9)	56(7)
C(17A)	2703(12)	7133(4)	1807(4)	33(2)
C(18A)	2616(13)	6345(4)	2545(4)	37(2)
C(19A)	2141(11)	7080(4)	1066(4)	33(2)
C(20A)	5184(11)	8302(3)	820(4)	28(2)
C(21A)	3700(11)	9026(4)	743(4)	36(2)
C(22A)	7103(12)	8515(4)	718(5)	43(2)
C(23A)	5707(13)	7404(5)	2698(4)	40(2)
C(24A)	7721(16)	7483(9)	2780(7)	77(4)
C(25A)	4343(17)	7996(6)	2856(5)	57(3)
S(1B)	6219(2)	3798(1)	305(1)	18(1)
S(2B)	3628(2)	4845(1)	1729(1)	18(1)
Si(1B)	9065(2)	867(1)	2060(1)	18(1)
C(1B)	5353(8)	3816(3)	1150(3)	20(1)
C(2B)	4488(8)	4619(3)	1009(3)	14(1)
C(3B)	3904(9)	3895(3)	2549(3)	20(1)
C(4B)	3234(10)	3888(3)	3215(3)	25(1)
C(5B)	3336(9)	3171(3)	3898(3)	17(1)
C(6B)	4158(9)	2439(3)	3939(3)	19(1)
C(7B)	4835(9)	2458(3)	3252(3)	19(1)
C(8B)	4757(8)	3170(3)	2547(3)	18(1)
C(9B)	5539(9)	3141(3)	1865(3)	21(1)
C(10B)	6591(9)	2393(3)	1919(3)	19(1)
C(11B)	7527(10)	1773(3)	1981(3)	24(1)
C(12B)	5481(9)	4827(3)	-235(3)	17(1)
C(13B)	4291(10)	1628(3)	4665(3)	24(1)
C(14B)	3259(12)	1740(4)	5322(4)	35(2)
C(15B)	3449(12)	1069(4)	4468(4)	35(2)
C(16B)	6316(11)	1265(4)	4978(4)	31(2)
C(17B)	11390(10)	1101(3)	1837(3)	23(1)
C(18B)	11280(11)	1838(4)	1035(4)	30(2)
C(19B)	12401(11)	1233(4)	2445(4)	33(2)
C(20B)	9015(10)	44(3)	3075(4)	30(2)
C(21B)	10350(13)	-734(4)	3266(4)	38(2)
C(22B)	9252(14)	289(4)	3727(4)	43(2)
C(23B)	8127(10)	628(3)	1296(4)	25(1)
C(24B)	6387(12)	307(5)	1521(5)	45(2)
C(25B)	9548(10)	46(4)	1105(4)	30(2)

Table S9. Bond lengths [Å] and angles [°] for **Th2-TIPS**.

S(1A)-C(12A)	1.720(6)	S(1A)-C(1A)	1.769(6)
S(2A)-C(2A)	1.739(5)	S(2A)-C(3A)	1.774(6)
Si(1A)-C(11A)	1.838(7)	Si(1A)-C(17A)	1.884(9)
Si(1A)-C(23A)	1.893(7)	Si(1A)-C(20A)	1.893(6)
C(1A)-C(9A)	1.339(9)	C(1A)-C(2A)	1.463(7)
C(2A)-C(12A)#1	1.355(9)	C(3A)-C(8A)	1.379(9)
C(3A)-C(4A)	1.426(8)	C(4A)-C(5A)	1.376(11)
C(4A)-H(4A)	0.9500	C(5A)-C(6A)	1.394(10)
C(5A)-H(5A)	0.9500	C(6A)-C(7A)	1.396(9)
C(6A)-C(13A)	1.535(10)	C(7A)-C(8A)	1.405(10)
C(7A)-H(7A)	0.9500	C(8A)-C(9A)	1.485(8)
C(9A)-C(10A)	1.436(8)	C(10A)-C(11A)	1.209(9)
C(12A)-C(2A)#1	1.355(9)	C(12A)-C(12A)#1	1.430(10)
C(13A)-C(14C)	1.520(8)	C(13A)-C(15C)	1.523(8)
C(13A)-C(16A)	1.523(7)	C(13A)-C(16C)	1.527(8)
C(13A)-C(14A)	1.528(7)	C(13A)-C(15A)	1.531(7)
C(14A)-H(14A)	0.9800	C(14A)-H(14B)	0.9800
C(14A)-H(14C)	0.9800	C(15A)-H(15A)	0.9800
C(15A)-H(15B)	0.9800	C(15A)-H(15C)	0.9800
C(16A)-H(16A)	0.9800	C(16A)-H(16B)	0.9800
C(16A)-H(16C)	0.9800	C(14C)-H(14D)	0.9800
C(14C)-H(14E)	0.9800	C(14C)-H(14F)	0.9800
C(15C)-H(15D)	0.9800	C(15C)-H(15E)	0.9800
C(15C)-H(15F)	0.9800	C(16C)-H(16D)	0.9800
C(16C)-H(16E)	0.9800	C(16C)-H(16F)	0.9800
C(17A)-C(19A)	1.534(9)	C(17A)-C(18A)	1.547(8)
C(17A)-H(17A)	1.0000	C(18A)-H(18A)	0.9800
C(18A)-H(18B)	0.9800	C(18A)-H(18C)	0.9800
C(19A)-H(19A)	0.9800	C(19A)-H(19B)	0.9800
C(19A)-H(19C)	0.9800	C(20A)-C(22A)	1.542(11)
C(20A)-C(21A)	1.546(9)	C(20A)-H(20A)	1.0000
C(21A)-H(21A)	0.9800	C(21A)-H(21B)	0.9800
C(21A)-H(21C)	0.9800	C(22A)-H(22A)	0.9800
C(22A)-H(22B)	0.9800	C(22A)-H(22C)	0.9800
C(23A)-C(25A)	1.507(12)	C(23A)-C(24A)	1.546(14)
C(23A)-H(23A)	1.0000	C(24A)-H(24A)	0.9800
C(24A)-H(24B)	0.9800	C(24A)-H(24C)	0.9800
C(25A)-H(25A)	0.9800	C(25A)-H(25B)	0.9800
C(25A)-H(25C)	0.9800		
S(1B)-C(12B)	1.733(5)	S(1B)-C(1B)	1.756(7)
S(2B)-C(2B)	1.711(5)	S(2B)-C(3B)	1.760(6)

Si(1B)-C(11B)	1.827(7)	Si(1B)-C(17B)	1.864(7)
Si(1B)-C(20B)	1.879(6)	Si(1B)-C(23B)	1.889(6)
C(1B)-C(9B)	1.379(8)	C(1B)-C(2B)	1.447(7)
C(2B)-C(12B)#2	1.386(8)	C(3B)-C(4B)	1.367(10)
C(3B)-C(8B)	1.411(9)	C(4B)-C(5B)	1.393(8)
C(4B)-H(4B)	0.9500	C(5B)-C(6B)	1.383(8)
C(5B)-H(5B)	0.9500	C(6B)-C(7B)	1.398(9)
C(6B)-C(13B)	1.532(7)	C(7B)-C(8B)	1.413(8)
C(7B)-H(7B)	0.9500	C(8B)-C(9B)	1.451(9)
C(9B)-C(10B)	1.451(8)	C(10B)-C(11B)	1.210(9)
C(12B)-C(2B)#2	1.386(8)	C(12B)-C(12B)#2	1.453(13)
C(13B)-C(15B)	1.513(8)	C(13B)-C(16B)	1.529(10)
C(13B)-C(14B)	1.549(10)	C(14B)-H(14G)	0.9800
C(14B)-H(14H)	0.9800	C(14B)-H(14I)	0.9800
C(15B)-H(15G)	0.9800	C(15B)-H(15H)	0.9800
C(15B)-H(15I)	0.9800	C(16B)-H(16G)	0.9800
C(16B)-H(16H)	0.9800	C(16B)-H(16I)	0.9800
C(17B)-C(19B)	1.526(9)	C(17B)-C(18B)	1.537(8)
C(17B)-H(17B)	1.0000	C(18B)-H(18D)	0.9800
C(18B)-H(18E)	0.9800	C(18B)-H(18F)	0.9800
C(19B)-H(19D)	0.9800	C(19B)-H(19E)	0.9800
C(19B)-H(19F)	0.9800	C(20B)-C(21B)	1.509(9)
C(20B)-C(22B)	1.540(10)	C(20B)-H(20B)	1.0000
C(21B)-H(21D)	0.9800	C(21B)-H(21E)	0.9800
C(21B)-H(21F)	0.9800	C(22B)-H(22D)	0.9800
C(22B)-H(22E)	0.9800	C(22B)-H(22F)	0.9800
C(23B)-C(24B)	1.512(10)	C(23B)-C(25B)	1.552(10)
C(23B)-H(23B)	1.0000	C(24B)-H(24D)	0.9800
C(24B)-H(24E)	0.9800	C(24B)-H(24F)	0.9800
C(25B)-H(25D)	0.9800	C(25B)-H(25E)	0.9800
C(25B)-H(25F)	0.9800		

C(12A)-S(1A)-C(1A)	91.4(3)	C(2A)-S(2A)-C(3A)	102.5(3)
C(11A)-Si(1A)-C(17A)	106.4(3)	C(11A)-Si(1A)-C(23A)	108.4(3)
C(17A)-Si(1A)-C(23A)	108.6(4)	C(11A)-Si(1A)-C(20A)	106.5(3)
C(17A)-Si(1A)-C(20A)	110.9(3)	C(23A)-Si(1A)-C(20A)	115.7(3)
C(9A)-C(1A)-C(2A)	127.3(5)	C(9A)-C(1A)-S(1A)	123.3(4)
C(2A)-C(1A)-S(1A)	109.5(4)	C(12A)#1-C(2A)-C(1A)	113.3(5)
C(12A)#1-C(2A)-S(2A)	125.8(4)	C(1A)-C(2A)-S(2A)	120.8(4)
C(8A)-C(3A)-C(4A)	119.9(6)	C(8A)-C(3A)-S(2A)	125.8(5)
C(4A)-C(3A)-S(2A)	114.3(5)	C(5A)-C(4A)-C(3A)	119.5(6)
C(5A)-C(4A)-H(4A)	120.2	C(3A)-C(4A)-H(4A)	120.2
C(4A)-C(5A)-C(6A)	122.0(6)	C(4A)-C(5A)-H(5A)	119.0

C(6A)-C(5A)-H(5A)	119.0	C(5A)-C(6A)-C(7A)	117.0(6)
C(5A)-C(6A)-C(13A)	123.1(6)	C(7A)-C(6A)-C(13A)	119.9(6)
C(6A)-C(7A)-C(8A)	122.9(6)	C(6A)-C(7A)-H(7A)	118.5
C(8A)-C(7A)-H(7A)	118.5	C(3A)-C(8A)-C(7A)	118.4(5)
C(3A)-C(8A)-C(9A)	121.1(6)	C(7A)-C(8A)-C(9A)	120.5(6)
C(1A)-C(9A)-C(10A)	122.2(5)	C(1A)-C(9A)-C(8A)	122.0(5)
C(10A)-C(9A)-C(8A)	115.8(6)	C(11A)-C(10A)-C(9A)	177.3(7)
C(10A)-C(11A)-Si(1A)	169.0(6)	C(2A)#1-C(12A)-C(12A)#1	113.2(7)
C(2A)#1-C(12A)-S(1A)	134.2(4)	C(12A)#1-C(12A)-S(1A)	112.5(6)
C(14C)-C(13A)-C(15C)	108.9(6)	C(14C)-C(13A)-C(16C)	108.3(6)
C(15C)-C(13A)-C(16C)	107.9(6)	C(16A)-C(13A)-C(14A)	107.9(5)
C(16A)-C(13A)-C(15A)	107.4(5)	C(14A)-C(13A)-C(15A)	106.4(5)
C(14C)-C(13A)-C(6A)	115.8(12)	C(15C)-C(13A)-C(6A)	109.3(10)
C(16A)-C(13A)-C(6A)	111.9(6)	C(16C)-C(13A)-C(6A)	106.2(9)
C(14A)-C(13A)-C(6A)	110.1(7)	C(15A)-C(13A)-C(6A)	112.9(6)
C(13A)-C(14A)-H(14A)	109.5	C(13A)-C(14A)-H(14B)	109.5
H(14A)-C(14A)-H(14B)	109.5	C(13A)-C(14A)-H(14C)	109.5
H(14A)-C(14A)-H(14C)	109.5	H(14B)-C(14A)-H(14C)	109.5
C(13A)-C(15A)-H(15A)	109.5	C(13A)-C(15A)-H(15B)	109.5
H(15A)-C(15A)-H(15B)	109.5	C(13A)-C(15A)-H(15C)	109.5
H(15A)-C(15A)-H(15C)	109.5	H(15B)-C(15A)-H(15C)	109.5
C(13A)-C(16A)-H(16A)	109.5	C(13A)-C(16A)-H(16B)	109.5
H(16A)-C(16A)-H(16B)	109.5	C(13A)-C(16A)-H(16C)	109.5
H(16A)-C(16A)-H(16C)	109.5	H(16B)-C(16A)-H(16C)	109.5
C(13A)-C(14C)-H(14D)	109.5	C(13A)-C(14C)-H(14E)	109.5
H(14D)-C(14C)-H(14E)	109.5	C(13A)-C(14C)-H(14F)	109.5
H(14D)-C(14C)-H(14F)	109.5	H(14E)-C(14C)-H(14F)	109.5
C(13A)-C(15C)-H(15D)	109.5	C(13A)-C(15C)-H(15E)	109.5
H(15D)-C(15C)-H(15E)	109.5	C(13A)-C(15C)-H(15F)	109.5
H(15D)-C(15C)-H(15F)	109.5	H(15E)-C(15C)-H(15F)	109.5
C(13A)-C(16C)-H(16D)	109.5	C(13A)-C(16C)-H(16E)	109.5
H(16D)-C(16C)-H(16E)	109.5	C(13A)-C(16C)-H(16F)	109.5
H(16D)-C(16C)-H(16F)	109.5	H(16E)-C(16C)-H(16F)	109.5
C(19A)-C(17A)-C(18A)	110.1(6)	C(19A)-C(17A)-Si(1A)	110.5(5)
C(18A)-C(17A)-Si(1A)	111.6(6)	C(19A)-C(17A)-H(17A)	108.2
C(18A)-C(17A)-H(17A)	108.2	Si(1A)-C(17A)-H(17A)	108.2
C(17A)-C(18A)-H(18A)	109.5	C(17A)-C(18A)-H(18B)	109.5
H(18A)-C(18A)-H(18B)	109.5	C(17A)-C(18A)-H(18C)	109.5
H(18A)-C(18A)-H(18C)	109.5	H(18B)-C(18A)-H(18C)	109.5
C(17A)-C(19A)-H(19A)	109.5	C(17A)-C(19A)-H(19B)	109.5
H(19A)-C(19A)-H(19B)	109.5	C(17A)-C(19A)-H(19C)	109.5
H(19A)-C(19A)-H(19C)	109.5	H(19B)-C(19A)-H(19C)	109.5
C(22A)-C(20A)-C(21A)	110.7(5)	C(22A)-C(20A)-Si(1A)	114.1(5)

C(21A)-C(20A)-Si(1A)	110.8(5)	C(22A)-C(20A)-H(20A)	107.0
C(21A)-C(20A)-H(20A)	107.0	Si(1A)-C(20A)-H(20A)	107.0
C(20A)-C(21A)-H(21A)	109.5	C(20A)-C(21A)-H(21B)	109.5
H(21A)-C(21A)-H(21B)	109.5	C(20A)-C(21A)-H(21C)	109.5
H(21A)-C(21A)-H(21C)	109.5	H(21B)-C(21A)-H(21C)	109.5
C(20A)-C(22A)-H(22A)	109.5	C(20A)-C(22A)-H(22B)	109.5
H(22A)-C(22A)-H(22B)	109.5	C(20A)-C(22A)-H(22C)	109.5
H(22A)-C(22A)-H(22C)	109.5	H(22B)-C(22A)-H(22C)	109.5
C(25A)-C(23A)-C(24A)	112.8(8)	C(25A)-C(23A)-Si(1A)	113.5(6)
C(24A)-C(23A)-Si(1A)	113.6(6)	C(25A)-C(23A)-H(23A)	105.3
C(24A)-C(23A)-H(23A)	105.3	Si(1A)-C(23A)-H(23A)	105.3
C(23A)-C(24A)-H(24A)	109.5	C(23A)-C(24A)-H(24B)	109.5
H(24A)-C(24A)-H(24B)	109.5	C(23A)-C(24A)-H(24C)	109.5
H(24A)-C(24A)-H(24C)	109.5	H(24B)-C(24A)-H(24C)	109.5
C(23A)-C(25A)-H(25A)	109.5	C(23A)-C(25A)-H(25B)	109.5
H(25A)-C(25A)-H(25B)	109.5	C(23A)-C(25A)-H(25C)	109.5
H(25A)-C(25A)-H(25C)	109.5		

H(25B)-C(25A)-H(25C)	109.5	C(12B)-S(1B)-C(1B)	91.5(3)
C(2B)-S(2B)-C(3B)	102.9(3)	C(11B)-Si(1B)-C(17B)	107.6(3)
C(11B)-Si(1B)-C(20B)	107.4(3)	C(17B)-Si(1B)-C(20B)	113.7(3)
C(11B)-Si(1B)-C(23B)	107.0(3)	C(17B)-Si(1B)-C(23B)	109.9(3)
C(20B)-Si(1B)-C(23B)	110.9(3)	C(9B)-C(1B)-C(2B)	124.9(6)
C(9B)-C(1B)-S(1B)	123.1(5)	C(2B)-C(1B)-S(1B)	112.0(4)
C(12B)#2-C(2B)-C(1B)	111.5(5)	C(12B)#2-C(2B)-S(2B)	125.2(4)
C(1B)-C(2B)-S(2B)	123.3(4)	C(4B)-C(3B)-C(8B)	120.1(5)
C(4B)-C(3B)-S(2B)	115.8(5)	C(8B)-C(3B)-S(2B)	124.1(5)
C(3B)-C(4B)-C(5B)	121.2(6)	C(3B)-C(4B)-H(4B)	119.4
C(5B)-C(4B)-H(4B)	119.4	C(6B)-C(5B)-C(4B)	121.8(6)
C(6B)-C(5B)-H(5B)	119.1	C(4B)-C(5B)-H(5B)	119.1
C(5B)-C(6B)-C(7B)	116.1(5)	C(5B)-C(6B)-C(13B)	124.6(5)
C(7B)-C(6B)-C(13B)	119.2(5)	C(6B)-C(7B)-C(8B)	123.9(6)
C(6B)-C(7B)-H(7B)	118.0	C(8B)-C(7B)-H(7B)	118.0
C(3B)-C(8B)-C(7B)	116.8(5)	C(3B)-C(8B)-C(9B)	122.7(5)
C(7B)-C(8B)-C(9B)	120.6(6)	C(1B)-C(9B)-C(10B)	117.6(6)
C(1B)-C(9B)-C(8B)	121.9(6)	C(10B)-C(9B)-C(8B)	120.5(5)
C(11B)-C(10B)-C(9B)	177.3(6)	C(10B)-C(11B)-Si(1B)	176.4(6)
C(2B)#2-C(12B)-C(12B)#2	113.6(6)	C(2B)#2-C(12B)-S(1B)	135.0(5)
C(12B)#2-C(12B)-S(1B)	111.3(5)	C(15B)-C(13B)-C(6B)	110.7(5)
C(15B)-C(13B)-C(16B)	110.7(5)	C(6B)-C(13B)-C(16B)	109.0(5)
C(15B)-C(13B)-C(14B)	108.4(6)	C(6B)-C(13B)-C(14B)	110.4(5)
C(16B)-C(13B)-C(14B)	107.6(5)	C(13B)-C(14B)-H(14G)	109.5
C(13B)-C(14B)-H(14H)	109.5	H(14G)-C(14B)-H(14H)	109.5

C(13B)-C(14B)-H(14I)	109.5	H(14G)-C(14B)-H(14I)	109.5
H(14H)-C(14B)-H(14I)	109.5	C(13B)-C(15B)-H(15G)	109.5
C(13B)-C(15B)-H(15H)	109.5	H(15G)-C(15B)-H(15H)	109.5
C(13B)-C(15B)-H(15I)	109.5	H(15G)-C(15B)-H(15I)	109.5
H(15H)-C(15B)-H(15I)	109.5	C(13B)-C(16B)-H(16G)	109.5
C(13B)-C(16B)-H(16H)	109.5	H(16G)-C(16B)-H(16H)	109.5
C(13B)-C(16B)-H(16I)	109.5	H(16G)-C(16B)-H(16I)	109.5
H(16H)-C(16B)-H(16I)	109.5	C(19B)-C(17B)-C(18B)	108.5(5)
C(19B)-C(17B)-Si(1B)	115.9(5)	C(18B)-C(17B)-Si(1B)	110.6(5)
C(19B)-C(17B)-H(17B)	107.1	C(18B)-C(17B)-H(17B)	107.1
Si(1B)-C(17B)-H(17B)	107.1	C(17B)-C(18B)-H(18D)	109.5
C(17B)-C(18B)-H(18E)	109.5	H(18D)-C(18B)-H(18E)	109.5
C(17B)-C(18B)-H(18F)	109.5	H(18D)-C(18B)-H(18F)	109.5
H(18E)-C(18B)-H(18F)	109.5	C(17B)-C(19B)-H(19D)	109.5
C(17B)-C(19B)-H(19E)	109.5	H(19D)-C(19B)-H(19E)	109.5
C(17B)-C(19B)-H(19F)	109.5	H(19D)-C(19B)-H(19F)	109.5
H(19E)-C(19B)-H(19F)	109.5	C(21B)-C(20B)-C(22B)	109.6(6)
C(21B)-C(20B)-Si(1B)	114.4(5)	C(22B)-C(20B)-Si(1B)	113.7(4)
C(21B)-C(20B)-H(20B)	106.2	C(22B)-C(20B)-H(20B)	106.2
Si(1B)-C(20B)-H(20B)	106.2	C(20B)-C(21B)-H(21D)	109.5
C(20B)-C(21B)-H(21E)	109.5	H(21D)-C(21B)-H(21E)	109.5
C(20B)-C(21B)-H(21F)	109.5	H(21D)-C(21B)-H(21F)	109.5
H(21E)-C(21B)-H(21F)	109.5	C(20B)-C(22B)-H(22D)	109.5
C(20B)-C(22B)-H(22E)	109.5	H(22D)-C(22B)-H(22E)	109.5
C(20B)-C(22B)-H(22F)	109.5	H(22D)-C(22B)-H(22F)	109.5
H(22E)-C(22B)-H(22F)	109.5	C(24B)-C(23B)-C(25B)	110.0(5)
C(24B)-C(23B)-Si(1B)	111.7(5)	C(25B)-C(23B)-Si(1B)	113.5(4)
C(24B)-C(23B)-H(23B)	107.1	C(25B)-C(23B)-H(23B)	107.1
Si(1B)-C(23B)-H(23B)	107.1	C(23B)-C(24B)-H(24D)	109.5
C(23B)-C(24B)-H(24E)	109.5	H(24D)-C(24B)-H(24E)	109.5
C(23B)-C(24B)-H(24F)	109.5	H(24D)-C(24B)-H(24F)	109.5
H(24E)-C(24B)-H(24F)	109.5	C(23B)-C(25B)-H(25D)	109.5
C(23B)-C(25B)-H(25E)	109.5	H(25D)-C(25B)-H(25E)	109.5
C(23B)-C(25B)-H(25F)	109.5	H(25D)-C(25B)-H(25F)	109.5
H(25E)-C(25B)-H(25F)	109.5		

Symmetry transformations used to generate equivalent atoms: #1 -x+2,-y+1,-z #2 -x+1,-y+1,-z

Table S10. Anisotropic displacement parameters (Å^{2}x 10^3) for **Th2-TIPS**. The anisotropic displacement factor exponent takes the form: $-2\pi^2[h^2a^{*2}U^{11} + ... + 2 h k a^* b^* U^{12}]$

	U^{11}	U^{22}	U^{33}	U^{23}	U^{13}	U^{12}
S(1A)	21(1)	13(1)	21(1)	-7(1)	0(1)	-4(1)
S(2A)	25(1)	14(1)	21(1)	-4(1)	-5(1)	-5(1)
Si(1A)	28(1)	18(1)	25(1)	-12(1)	1(1)	-6(1)
C(1A)	18(3)	18(3)	23(3)	-10(2)	-2(2)	-5(2)
C(2A)	12(3)	14(2)	17(3)	-10(2)	0(2)	-1(2)
C(3A)	9(3)	25(3)	19(3)	-8(2)	0(2)	-6(2)
C(4A)	37(4)	21(3)	28(3)	1(3)	-16(3)	-14(3)
C(5A)	34(4)	34(3)	20(3)	1(3)	-3(3)	-16(3)
C(6A)	27(4)	38(3)	20(3)	-9(3)	-6(3)	-7(3)
C(7A)	24(4)	27(3)	23(3)	-5(3)	-9(3)	5(3)
C(8A)	26(4)	26(3)	17(3)	-2(2)	-12(3)	-9(3)
C(9A)	15(3)	13(2)	24(3)	-5(2)	-5(2)	-5(2)
C(10A)	16(3)	26(3)	22(3)	-12(2)	-1(3)	2(3)
C(11A)	24(4)	25(3)	21(3)	-12(2)	3(2)	-5(3)
C(12A)	17(3)	9(3)	29(3)	-9(2)	2(2)	0(2)
C(13A)	32(4)	44(4)	16(3)	-7(3)	-4(3)	-15(3)
C(14A)	41(9)	75(9)	62(9)	-57(8)	-27(7)	25(7)
C(15A)	124(17)	48(7)	21(5)	-21(5)	13(8)	-28(10)
C(16A)	70(11)	77(10)	47(7)	-53(7)	42(8)	-47(9)
C(14C)	80(13)	76(12)	77(12)	-33(8)	14(9)	-7(8)
C(15C)	47(10)	68(10)	62(10)	-32(8)	12(8)	-1(8)
C(16C)	72(11)	52(9)	47(9)	-28(7)	13(8)	-8(7)
C(17A)	43(5)	22(3)	28(3)	-7(3)	9(3)	-6(3)
C(18A)	48(5)	30(3)	34(4)	-12(3)	7(3)	-17(3)
C(19A)	29(4)	32(3)	38(4)	-19(3)	-6(3)	1(3)
C(20A)	38(4)	17(3)	28(3)	-12(2)	7(3)	1(3)
C(21A)	27(4)	24(3)	48(4)	-14(3)	20(3)	5(3)
C(22A)	40(5)	20(3)	58(5)	-6(3)	5(4)	-9(3)
C(23A)	48(5)	36(3)	34(4)	-21(3)	-8(4)	5(4)
C(24A)	61(7)	132(11)	70(7)	-76(7)	-11(6)	-20(8)
C(25A)	73(7)	65(5)	45(4)	-41(4)	-8(5)	-1(5)
S(1B)	19(1)	12(1)	19(1)	-4(1)	-3(1)	-4(1)
S(2B)	20(1)	14(1)	16(1)	-5(1)	-1(1)	-2(1)
Si(1B)	16(1)	14(1)	22(1)	-6(1)	-1(1)	-3(1)
C(1B)	15(3)	12(2)	25(3)	-3(2)	-10(3)	0(2)

C(2B)	10(2)	18(2)	16(2)	-10(2)	8(2)	-2(2)
C(3B)	21(3)	21(3)	16(3)	-4(2)	-7(2)	-12(2)
C(4B)	36(4)	13(2)	25(3)	-6(2)	-3(3)	-9(3)
C(5B)	17(3)	23(3)	15(2)	-10(2)	5(2)	-7(2)
C(6B)	18(3)	19(3)	21(3)	-10(2)	-3(2)	-3(2)
C(7B)	16(3)	21(3)	17(3)	-5(2)	5(2)	-5(2)
C(8B)	11(3)	21(3)	19(3)	-6(2)	2(2)	-11(2)
C(9B)	19(3)	19(3)	21(3)	-4(2)	-7(2)	-12(2)
C(10B)	22(3)	14(2)	16(2)	-3(2)	0(2)	-5(2)
C(11B)	31(4)	16(3)	15(3)	2(2)	-8(3)	-9(3)
C(12B)	17(3)	14(2)	17(3)	-5(2)	-4(2)	-6(2)
C(13B)	36(4)	18(3)	19(3)	-7(2)	1(3)	-9(3)
C(14B)	47(5)	31(3)	21(3)	-6(3)	16(3)	-13(3)
C(15B)	52(5)	19(3)	27(3)	-1(2)	-5(3)	-13(3)
C(16B)	34(4)	27(3)	22(3)	-4(2)	-5(3)	-3(3)
C(17B)	20(3)	22(3)	24(3)	-9(2)	2(3)	-4(3)
C(18B)	32(4)	23(3)	34(3)	-11(3)	18(3)	-9(3)
C(19B)	24(4)	38(3)	37(3)	-15(3)	-3(3)	-15(3)
C(20B)	26(4)	19(3)	31(3)	1(2)	2(3)	-5(3)
C(21B)	44(5)	21(3)	32(3)	2(3)	-7(3)	-2(3)
C(22B)	55(5)	36(3)	17(3)	-1(3)	7(3)	8(4)
C(23B)	27(4)	21(3)	33(3)	-16(2)	-10(3)	-6(3)
C(24B)	37(5)	55(4)	68(5)	-47(4)	10(4)	-19(4)
C(25B)	16(3)	38(3)	42(4)	-24(3)	2(3)	-7(3)

Table S11. Hydrogen coordinates (x 10^4) and isotropic displacement parameters ($Å^2$x 10^3) for **Th2-TIPS**.

	x	y	z	U(eq)
H(4A)	9547	2492	3334	38
H(5A)	8741	2653	4427	40
H(7A)	7632	5052	3123	34
H(14A)	9288	3717	5674	83
H(14B)	10564	3877	4966	83
H(14C)	9873	3049	5382	83
H(15A)	6159	5139	4105	91
H(15B)	8302	5154	4159	91
H(15C)	7203	4933	4924	91
H(16A)	6621	3031	5262	76
H(16B)	5161	3814	4673	76

H(16C)	5971	3754	5474	76
H(14D)	10372	4048	5015	120
H(14E)	8703	4518	5271	120
H(14F)	9233	4906	4386	120
H(15D)	5603	4542	4993	90
H(15E)	4995	4198	4429	90
H(15F)	5753	5010	4069	90
H(16D)	9016	2796	5296	83
H(16E)	6817	2974	5279	83
H(16F)	8024	3195	5811	83
H(17A)	1802	7579	1833	39
H(18A)	1418	6207	2522	56
H(18B)	2776	6416	3015	56
H(18C)	3600	5912	2566	56
H(19A)	2184	7585	602	49
H(19B)	887	6983	1088	49
H(19C)	2994	6638	1035	49
H(20A)	4885	8190	377	34
H(21A)	2477	8943	651	54
H(21B)	3915	9516	302	54
H(21C)	3769	9077	1227	54
H(22A)	8007	8065	725	65
H(22B)	7455	8621	1147	65
H(22C)	7066	8993	216	65
H(23A)	5636	6865	3129	48
H(24A)	8449	7307	2440	115
H(24B)	8239	7148	3326	115
H(24C)	7750	8044	2627	115
H(25A)	4561	7886	3402	86
H(25B)	3088	7946	2759	86
H(25C)	4492	8542	2507	86
H(4B)	2690	4381	3214	30
H(5B)	2827	3186	4347	20
H(7B)	5381	1963	3259	23
H(14G)	3679	2153	5410	52
H(14H)	3513	1232	5807	52
H(14I)	1931	1906	5165	52
H(15G)	2169	1322	4248	53
H(15H)	3481	566	4945	53
H(15I)	4152	956	4084	53
H(16G)	6404	777	5475	46
H(16H)	6863	1652	5062	46
H(16I)	6980	1132	4596	46

H(17B)	12170	634	1799	27
H(18D)	10615	2317	1063	45
H(18E)	10628	1763	638	45
H(18F)	12529	1903	891	45
H(19D)	13422	1494	2214	49
H(19E)	12884	715	2900	49
H(19F)	11545	1575	2610	49
H(20B)	7755	-76	3098	36
H(21D)	10057	-936	2902	58
H(21E)	10251	-1129	3804	58
H(21F)	11609	-640	3215	58
H(22D)	9177	-161	4237	64
H(22E)	8274	751	3641	64
H(22F)	10455	432	3717	64
H(23B)	7788	1144	802	31
H(24D)	5737	346	1057	67
H(24E)	5590	623	1732	67
H(24F)	6706	-255	1920	67
H(25D)	9839	-483	1565	45
H(25E)	10675	256	965	45
H(25F)	9028	-4	664	45

Table S12. Torsion angles [°] for **Th2-TIPS**.

C(12A)-S(1A)-C(1A)-C(9A)	-179.9(6)
C(12A)-S(1A)-C(1A)-C(2A)	0.5(5)
C(9A)-C(1A)-C(2A)-C(12A)#1	179.4(6)
S(1A)-C(1A)-C(2A)-C(12A)#1	-1.0(7)
C(9A)-C(1A)-C(2A)-S(2A)	2.1(9)
S(1A)-C(1A)-C(2A)-S(2A)	-178.3(3)
C(3A)-S(2A)-C(2A)-C(12A)#1	-178.5(6)
C(3A)-S(2A)-C(2A)-C(1A)	-1.5(5)
C(2A)-S(2A)-C(3A)-C(8A)	-3.3(6)
C(2A)-S(2A)-C(3A)-C(4A)	178.9(5)
C(8A)-C(3A)-C(4A)-C(5A)	-4.2(10)
S(2A)-C(3A)-C(4A)-C(5A)	173.7(5)
C(3A)-C(4A)-C(5A)-C(6A)	-0.1(11)
C(4A)-C(5A)-C(6A)-C(7A)	4.4(12)
C(4A)-C(5A)-C(6A)-C(13A)	-174.7(6)
C(5A)-C(6A)-C(7A)-C(8A)	-4.7(11)
C(13A)-C(6A)-C(7A)-C(8A)	174.4(6)
C(4A)-C(3A)-C(8A)-C(7A)	3.9(10)
S(2A)-C(3A)-C(8A)-C(7A)	-173.7(5)

C(4A)-C(3A)-C(8A)-C(9A)	-174.7(6)
S(2A)-C(3A)-C(8A)-C(9A)	7.7(9)
C(6A)-C(7A)-C(8A)-C(3A)	0.6(11)
C(6A)-C(7A)-C(8A)-C(9A)	179.2(6)
C(2A)-C(1A)-C(9A)-C(10A)	-176.6(6)
S(1A)-C(1A)-C(9A)-C(10A)	3.8(9)
C(2A)-C(1A)-C(9A)-C(8A)	2.1(10)
S(1A)-C(1A)-C(9A)-C(8A)	-177.4(5)
C(3A)-C(8A)-C(9A)-C(1A)	-7.1(10)
C(7A)-C(8A)-C(9A)-C(1A)	174.3(6)
C(3A)-C(8A)-C(9A)-C(10A)	171.7(6)
C(7A)-C(8A)-C(9A)-C(10A)	-6.8(9)
C(17A)-Si(1A)-C(11A)-C(10A)	-5(3)
C(23A)-Si(1A)-C(11A)-C(10A)	112(3)
C(20A)-Si(1A)-C(11A)-C(10A)	-123(3)
C(1A)-S(1A)-C(12A)-C(2A)#1	179.2(7)
C(1A)-S(1A)-C(12A)-C(12A)#1	0.0(7)
C(5A)-C(6A)-C(13A)-C(14C)	114.6(12)
C(7A)-C(6A)-C(13A)-C(14C)	-64.5(12)
C(5A)-C(6A)-C(13A)-C(15C)	-121.9(11)
C(7A)-C(6A)-C(13A)-C(15C)	59.0(11)
C(5A)-C(6A)-C(13A)-C(16A)	-60.0(10)
C(7A)-C(6A)-C(13A)-C(16A)	120.8(8)
C(5A)-C(6A)-C(13A)-C(16C)	-5.7(12)
C(7A)-C(6A)-C(13A)-C(16C)	175.2(10)
C(5A)-C(6A)-C(13A)-C(14A)	60.0(9)
C(7A)-C(6A)-C(13A)-C(14A)	-119.2(8)
C(5A)-C(6A)-C(13A)-C(15A)	178.7(8)
C(7A)-C(6A)-C(13A)-C(15A)	-0.4(10)
C(11A)-Si(1A)-C(17A)-C(19A)	-62.1(5)
C(23A)-Si(1A)-C(17A)-C(19A)	-178.5(4)
C(20A)-Si(1A)-C(17A)-C(19A)	53.4(5)
C(11A)-Si(1A)-C(17A)-C(18A)	60.8(5)
C(23A)-Si(1A)-C(17A)-C(18A)	-55.6(6)
C(20A)-Si(1A)-C(17A)-C(18A)	176.2(5)
C(11A)-Si(1A)-C(20A)-C(22A)	-61.8(6)
C(17A)-Si(1A)-C(20A)-C(22A)	-177.1(5)
C(23A)-Si(1A)-C(20A)-C(22A)	58.7(6)
C(11A)-Si(1A)-C(20A)-C(21A)	172.5(5)
C(17A)-Si(1A)-C(20A)-C(21A)	57.2(6)
C(23A)-Si(1A)-C(20A)-C(21A)	-67.0(6)
C(11A)-Si(1A)-C(23A)-C(25A)	-172.3(7)
C(17A)-Si(1A)-C(23A)-C(25A)	-57.1(8)

C(20A)-Si(1A)-C(23A)-C(25A)	68.3(8)
C(11A)-Si(1A)-C(23A)-C(24A)	57.1(8)
C(17A)-Si(1A)-C(23A)-C(24A)	172.2(8)
C(20A)-Si(1A)-C(23A)-C(24A)	-62.4(9)
C(12B)-S(1B)-C(1B)-C(9B)	-177.9(5)
C(12B)-S(1B)-C(1B)-C(2B)	0.3(5)
C(9B)-C(1B)-C(2B)-C(12B)#2	179.6(6)
S(1B)-C(1B)-C(2B)-C(12B)#2	1.4(6)
C(9B)-C(1B)-C(2B)-S(2B)	2.0(9)
S(1B)-C(1B)-C(2B)-S(2B)	-176.2(3)
C(3B)-S(2B)-C(2B)-C(12B)#2	177.7(5)
C(3B)-S(2B)-C(2B)-C(1B)	-5.1(6)
C(2B)-S(2B)-C(3B)-C(4B)	-176.8(5)
C(2B)-S(2B)-C(3B)-C(8B)	3.4(6)
C(8B)-C(3B)-C(4B)-C(5B)	-1.6(10)
S(2B)-C(3B)-C(4B)-C(5B)	178.6(5)
C(3B)-C(4B)-C(5B)-C(6B)	1.7(10)
C(4B)-C(5B)-C(6B)-C(7B)	-1.6(9)
C(4B)-C(5B)-C(6B)-C(13B)	-179.0(6)
C(5B)-C(6B)-C(7B)-C(8B)	1.5(9)
C(13B)-C(6B)-C(7B)-C(8B)	179.0(6)
C(4B)-C(3B)-C(8B)-C(7B)	1.3(9)
S(2B)-C(3B)-C(8B)-C(7B)	-178.9(4)
C(4B)-C(3B)-C(8B)-C(9B)	-178.2(6)
S(2B)-C(3B)-C(8B)-C(9B)	1.6(8)
C(6B)-C(7B)-C(8B)-C(3B)	-1.3(9)
C(6B)-C(7B)-C(8B)-C(9B)	178.2(6)
C(2B)-C(1B)-C(9B)-C(10B)	-174.6(5)
S(1B)-C(1B)-C(9B)-C(10B)	3.4(8)
C(2B)-C(1B)-C(9B)-C(8B)	4.1(9)
S(1B)-C(1B)-C(9B)-C(8B)	-177.9(4)
C(3B)-C(8B)-C(9B)-C(1B)	-5.9(9)
C(7B)-C(8B)-C(9B)-C(1B)	174.6(6)
C(3B)-C(8B)-C(9B)-C(10B)	172.7(5)
C(7B)-C(8B)-C(9B)-C(10B)	-6.8(8)
C(1B)-S(1B)-C(12B)-C(2B)#2	-177.8(7)
C(1B)-S(1B)-C(12B)-C(12B)#2	-1.9(6)
C(5B)-C(6B)-C(13B)-C(15B)	124.1(7)
C(7B)-C(6B)-C(13B)-C(15B)	-53.2(8)
C(5B)-C(6B)-C(13B)-C(16B)	-114.0(7)
C(7B)-C(6B)-C(13B)-C(16B)	68.7(7)
C(5B)-C(6B)-C(13B)-C(14B)	4.0(9)

C(7B)-C(6B)-C(13B)-C(14B)	-173.3(6)
C(11B)-Si(1B)-C(17B)-C(19B)	69.2(5)
C(20B)-Si(1B)-C(17B)-C(19B)	-49.7(6)
C(23B)-Si(1B)-C(17B)-C(19B)	-174.7(4)
C(11B)-Si(1B)-C(17B)-C(18B)	-54.9(5)
C(20B)-Si(1B)-C(17B)-C(18B)	-173.8(4)
C(23B)-Si(1B)-C(17B)-C(18B)	61.2(5)
C(11B)-Si(1B)-C(20B)-C(21B)	-174.7(5)
C(17B)-Si(1B)-C(20B)-C(21B)	-55.8(6)
C(23B)-Si(1B)-C(20B)-C(21B)	68.7(6)
C(11B)-Si(1B)-C(20B)-C(22B)	-47.7(6)
C(17B)-Si(1B)-C(20B)-C(22B)	71.2(6)
C(23B)-Si(1B)-C(20B)-C(22B)	-164.3(6)
C(11B)-Si(1B)-C(23B)-C(24B)	-72.6(5)
C(17B)-Si(1B)-C(23B)-C(24B)	170.9(5)
C(20B)-Si(1B)-C(23B)-C(24B)	44.3(6)
C(11B)-Si(1B)-C(23B)-C(25B)	162.3(4)
C(17B)-Si(1B)-C(23B)-C(25B)	45.7(5)
C(20B)-Si(1B)-C(23B)-C(25B)	-80.9(5)

Symmetry transformations used to generate equivalent atoms: #1 -x+2,-y+1,-z
#2 -x+1,-y+1,-z

Table S13. Crystal data and structure refinement for **Th3-TIPS**.

Empirical formula	$C_{104}H_{132}S_{10}Si_4$	
Formula weight	1815.05	
Temperature	173(2) K	
Wavelength	1.54178 Å	
Crystal system	Triclinic	
Space group	P -1	
Unit cell dimensions	a = 14.9964(4) Å	a = 96.6629(12)°
	b = 18.3543(5) Å	b = 99.0302(12)°
	c = 19.3746(5) Å	g = 98.7364(11)°
Volume	5151.9(2) Å^3	
Z	2	
Density (calculated)	1.170 Mg/m^3	
Absorption coefficient	2.757 mm^{-1}	
F(000)	1944	
Crystal size	0.314 x 0.070 x 0.056 mm^3	

Theta range for data collection	2.461 to 79.528°
Index ranges	-18<=h<=17, -23<=k<=23, -24<=l<=24
Reflections collected	125655
Independent reflections	22034 [R(int) = 0.0655]
Completeness to theta = 67.679°	99.7 %
Refinement method	Full-matrix least-squares on F^2
Data / restraints / parameters	22034 / 46 / 1227
Goodness-of-fit on F^2	1.027
Final R indices [I>2sigma(I)]	R1 = 0.0442, wR2 = 0.1035
R indices (all data)	R1 = 0.0642, wR2 = 0.1145
Largest diff. peak and hole	0.562 and -0.590 e.Å^{-3}

Table S14. Atomic coordinates (x 10^4) and equivalent isotropic displacement parameters (Å^{2}x 10^3) for **Th3-TIPS**. U(eq) is defined as one third of the trace of the orthogonalized U^{ij} tensor.

	x	y	z	U(eq)
Si(1A)	3362(1)	3625(1)	8642(1)	25(1)
Si(2A)	-231(1)	2891(1)	3444(1)	29(1)
S(1A)	1016(1)	1661(1)	7136(1)	21(1)
S(2A)	-286(1)	1367(1)	5227(1)	21(1)
S(3A)	263(1)	-183(1)	8243(1)	33(1)
S(4A)	-743(1)	-405(1)	6408(1)	21(1)
S(5A)	-1960(1)	-810(1)	4601(1)	20(1)
C(1A)	1039(1)	1143(1)	7847(1)	22(1)
C(2A)	1571(1)	1411(1)	8497(1)	23(1)
C(3A)	1539(1)	982(1)	9091(1)	23(1)
C(4A)	2099(1)	1257(1)	9748(1)	26(1)
C(5A)	2095(2)	884(1)	10333(1)	31(1)
C(6A)	1483(2)	216(1)	10255(1)	36(1)
C(7A)	928(2)	-74(1)	9614(1)	34(1)
C(8A)	962(2)	293(1)	9032(1)	26(1)
C(9A)	445(1)	437(1)	7649(1)	22(1)
C(10A)	35(1)	328(1)	6949(1)	21(1)
C(11A)	-769(1)	119(1)	5708(1)	20(1)
C(12A)	-1243(1)	4(1)	5024(1)	19(1)
C(13A)	-2400(1)	-557(1)	3781(1)	21(1)
C(14A)	-3032(1)	-1111(1)	3330(1)	24(1)
C(15A)	-3425(1)	-984(1)	2670(1)	27(1)

C(16A)	-3206(1)	-298(1)	2436(1)	26(1)
C(17A)	-2564(1)	248(1)	2891(1)	25(1)
C(18A)	-2145(1)	138(1)	3562(1)	21(1)
C(19A)	-1474(1)	730(1)	4012(1)	21(1)
C(20A)	-1078(1)	644(1)	4681(1)	20(1)
C(21A)	-200(1)	831(1)	5905(1)	20(1)
C(22A)	259(1)	939(1)	6588(1)	21(1)
C(23A)	2760(2)	1202(1)	11022(1)	41(1)
C(24A)	2612(2)	1989(2)	11267(2)	48(1)
C(25A)	3742(2)	1239(2)	10875(2)	61(1)
C(26A)	2650(3)	717(2)	11602(2)	80(1)
C(27A)	-3700(2)	-132(2)	1732(1)	32(1)
C(28A)	-4070(2)	-850(2)	1207(1)	42(1)
C(29A)	-4512(2)	242(2)	1878(1)	47(1)
C(30A)	-3060(2)	385(2)	1385(1)	41(1)
C(31A)	2137(1)	2124(1)	8585(1)	24(1)
C(32A)	2611(2)	2729(1)	8621(1)	27(1)
C(33A)	4510(2)	3529(1)	9155(1)	32(1)
C(34A)	5282(2)	4175(2)	9141(2)	45(1)
C(35A)	4445(2)	3406(2)	9915(1)	41(1)
C(36A)	2856(2)	4393(1)	9091(1)	36(1)
C(37A)	1801(2)	4248(2)	8973(2)	50(1)
C(38A)	3208(2)	5161(2)	8892(2)	50(1)
C(39A)	3452(2)	3745(1)	7700(1)	35(1)
C(40A)	3943(2)	3158(2)	7369(2)	50(1)
C(41A)	2510(2)	3725(2)	7242(1)	49(1)
C(42A)	-1185(1)	1426(1)	3783(1)	25(1)
C(43A)	-847(2)	2031(1)	3673(1)	29(1)
C(44A)	869(2)	3185(1)	4115(1)	37(1)
C(45A)	1495(2)	3842(2)	3923(2)	52(1)
C(46A)	1407(2)	2560(2)	4280(2)	44(1)
C(47A)	-990(2)	3621(2)	3493(1)	40(1)
C(48A)	-928(3)	3999(2)	4248(2)	74(1)
C(49A)	-845(2)	4199(2)	2986(2)	57(1)
C(50A)	-73(2)	2629(2)	2504(1)	40(1)
C(51A)	671(2)	2144(2)	2445(2)	48(1)
C(52A)	-987(2)	2230(2)	2055(1)	51(1)
Si(2B)	5716(1)	13093(1)	3473(1)	37(1)
S(1B)	4088(1)	8638(1)	2814(1)	21(1)
S(2B)	5476(1)	10515(1)	3850(1)	21(1)
S(3B)	4383(1)	6813(1)	4001(1)	29(1)
S(4B)	5678(1)	8576(1)	4804(1)	21(1)
S(5B)	6961(1)	10235(1)	5870(1)	19(1)

C(1B)	3935(1)	7710(1)	2982(1)	22(1)
C(2B)	3409(1)	7131(1)	2499(1)	22(1)
C(3B)	3366(1)	6356(1)	2637(1)	24(1)
C(4B)	2890(2)	5775(1)	2111(1)	27(1)
C(6B)	3247(2)	4860(1)	2842(1)	33(1)
C(7B)	3725(2)	5420(1)	3368(1)	32(1)
C(8B)	3790(1)	6163(1)	3272(1)	26(1)
C(9B)	4442(1)	7644(1)	3651(1)	22(1)
C(10B)	4929(1)	8316(1)	3994(1)	21(1)
C(11B)	5816(1)	9503(1)	4649(1)	20(1)
C(12B)	6321(1)	10156(1)	5029(1)	19(1)
C(13B)	7507(1)	11177(1)	6012(1)	19(1)
C(14B)	8136(1)	11387(1)	6640(1)	21(1)
C(15B)	8615(1)	12108(1)	6810(1)	23(1)
C(16B)	8477(1)	12644(1)	6368(1)	23(1)
C(17B)	7830(1)	12428(1)	5751(1)	23(1)
C(18B)	7330(1)	11699(1)	5556(1)	20(1)
C(19B)	6661(1)	11506(1)	4894(1)	21(1)
C(20B)	6231(1)	10779(1)	4667(1)	20(1)
C(21B)	5310(1)	9589(1)	3991(1)	20(1)
C(22B)	4815(1)	8929(1)	3626(1)	21(1)
C(5B)	2826(2)	5026(1)	2197(1)	31(1)
C(23B)	2380(4)	4407(2)	1574(2)	33(1)
C(24B)	1819(4)	4696(2)	969(2)	66(2)
C(25B)	3126(3)	4053(2)	1279(3)	56(1)
C(26B)	1745(3)	3813(3)	1835(2)	51(1)
C(23')	2164(4)	4416(4)	1662(4)	35(4)
C(24')	1170(4)	4521(4)	1626(5)	39(2)
C(25')	2452(6)	4498(5)	950(4)	45(3)
C(26')	2260(7)	3649(3)	1852(5)	46(3)
C(27B)	9075(1)	13423(1)	6538(1)	28(1)
C(28B)	9936(2)	13394(1)	6208(2)	39(1)
C(29B)	9365(2)	13660(2)	7339(1)	45(1)
C(30B)	8563(2)	14013(1)	6242(1)	35(1)
C(31B)	2931(1)	7281(1)	1852(1)	24(1)
C(32B)	2514(2)	7391(1)	1298(1)	28(1)
C(33B)	1448(2)	6499(1)	-14(1)	43(1)
C(34B)	1138(3)	6412(2)	-815(2)	65(1)
C(35B)	704(2)	6109(2)	343(2)	69(1)
C(36B)	2749(2)	7997(2)	-33(1)	37(1)
C(37B)	3494(2)	7539(2)	-166(2)	62(1)
C(38B)	3199(2)	8760(2)	389(2)	51(1)
Si(1B)	1884(1)	7490(1)	430(1)	26(1)

C(39B)	995(2)	8112(2)	554(2)	25(1)
C(40B)	154(4)	7936(5)	-42(3)	48(2)
C(41B)	658(4)	8057(4)	1264(2)	67(2)
C(39')	820(5)	7841(6)	645(6)	69(5)
C(40')	203(8)	8037(9)	13(7)	61(7)
C(41')	1044(10)	8480(6)	1263(7)	69(4)
C(42B)	6436(1)	12060(1)	4471(1)	24(1)
C(43B)	6172(2)	12479(1)	4082(1)	31(1)
C(44B)	5746(2)	12608(2)	2525(2)	31(1)
C(45B)	6563(5)	12400(5)	2446(5)	106(3)
C(46B)	5302(3)	13006(4)	1938(3)	45(1)
C(44')	6150(8)	13006(7)	2650(5)	51(3)
C(45')	6747(5)	12577(5)	2425(5)	16(2)
C(46')	5506(9)	13257(7)	2055(7)	57(5)
C(47B)	4458(2)	13049(2)	3492(1)	40(1)
C(48B)	3944(2)	12243(2)	3308(2)	60(1)
C(49B)	4239(2)	13405(2)	4179(2)	54(1)
C(50B)	6404(5)	14006(3)	3699(3)	35(1)
C(51B)	5990(6)	14621(4)	3343(4)	60(2)
C(52B)	6659(5)	14261(4)	4494(3)	44(1)
C(50')	6427(12)	14187(8)	3952(7)	39(4)
C(51')	6024(19)	14803(11)	3587(9)	83(7)
C(52')	6482(14)	14380(9)	4747(7)	58(4)

Table S15. Bond lengths [Å] and angles [°] for **Th3-TIPS.**

Si(1A)-C(32A)	1.840(2)	Si(1A)-C(39A)	1.886(2)
Si(1A)-C(36A)	1.889(2)	Si(1A)-C(33A)	1.892(2)
Si(2A)-C(43A)	1.841(2)	Si(2A)-C(47A)	1.889(3)
Si(2A)-C(50A)	1.890(3)	Si(2A)-C(44A)	1.895(2)
S(1A)-C(22A)	1.735(2)	S(1A)-C(1A)	1.761(2)
S(2A)-C(21A)	1.730(2)	S(2A)-C(20A)	1.758(2)
S(3A)-C(9A)	1.733(2)	S(3A)-C(8A)	1.763(2)
S(4A)-C(11A)	1.751(2)	S(4A)-C(10A)	1.757(2)
S(5A)-C(12A)	1.736(2)	S(5A)-C(13A)	1.763(2)
C(1A)-C(2A)	1.375(3)	C(1A)-C(9A)	1.430(3)
C(2A)-C(31A)	1.424(3)	C(2A)-C(3A)	1.470(3)
C(3A)-C(8A)	1.402(3)	C(3A)-C(4A)	1.402(3)
C(4A)-C(5A)	1.392(3)	C(4A)-H(4A)	0.9500
C(5A)-C(6A)	1.392(3)	C(5A)-C(23A)	1.530(3)
C(6A)-C(7A)	1.382(3)	C(6A)-H(6A)	0.9500
C(7A)-C(8A)	1.382(3)	C(7A)-H(7A)	0.9500
C(9A)-C(10A)	1.376(3)	C(10A)-C(22A)	1.414(3)

C(11A)-C(12A)	1.377(3)	C(11A)-C(21A)	1.422(3)
C(12A)-C(20A)	1.425(3)	C(13A)-C(14A)	1.392(3)
C(13A)-C(18A)	1.408(3)	C(14A)-C(15A)	1.382(3)
C(14A)-H(14A)	0.9500	C(15A)-C(16A)	1.396(3)
C(15A)-H(15A)	0.9500	C(16A)-C(17A)	1.394(3)
C(16A)-C(27A)	1.535(3)	C(17A)-C(18A)	1.402(3)
C(17A)-H(17A)	0.9500	C(18A)-C(19A)	1.459(3)
C(19A)-C(20A)	1.376(3)	C(19A)-C(42A)	1.428(3)
C(21A)-C(22A)	1.370(3)	C(23A)-C(26A)	1.525(4)
C(23A)-C(24A)	1.526(4)	C(23A)-C(25A)	1.537(4)
C(24A)-H(24A)	0.9800	C(24A)-H(24B)	0.9800
C(24A)-H(24C)	0.9800	C(25A)-H(25A)	0.9800
C(25A)-H(25B)	0.9800	C(25A)-H(25C)	0.9800
C(26A)-H(26A)	0.9800	C(26A)-H(26B)	0.9800
C(26A)-H(26C)	0.9800	C(27A)-C(30A)	1.531(4)
C(27A)-C(29A)	1.532(3)	C(27A)-C(28A)	1.539(3)
C(28A)-H(28A)	0.9800	C(28A)-H(28B)	0.9800
C(28A)-H(28C)	0.9800	C(29A)-H(29A)	0.9800
C(29A)-H(29B)	0.9800	C(29A)-H(29C)	0.9800
C(30A)-H(30A)	0.9800	C(30A)-H(30B)	0.9800
C(30A)-H(30C)	0.9800	C(31A)-C(32A)	1.212(3)
C(33A)-C(35A)	1.532(4)	C(33A)-C(34A)	1.532(3)
C(33A)-H(33A)	1.0000	C(34A)-H(34A)	0.9800
C(34A)-H(34B)	0.9800	C(34A)-H(34C)	0.9800
C(35A)-H(35A)	0.9800	C(35A)-H(35B)	0.9800
C(35A)-H(35C)	0.9800	C(36A)-C(37A)	1.539(4)
C(36A)-C(38A)	1.541(4)	C(36A)-H(36A)	1.0000
C(37A)-H(37A)	0.9800	C(37A)-H(37B)	0.9800
C(37A)-H(37C)	0.9800	C(38A)-H(38A)	0.9800
C(38A)-H(38B)	0.9800	C(38A)-H(38C)	0.9800
C(39A)-C(40A)	1.531(4)	C(39A)-C(41A)	1.539(4)
C(39A)-H(39A)	1.0000	C(40A)-H(40A)	0.9800
C(40A)-H(40B)	0.9800	C(40A)-H(40C)	0.9800
C(41A)-H(41A)	0.9800	C(41A)-H(41B)	0.9800
C(41A)-H(41C)	0.9800	C(42A)-C(43A)	1.205(3)
C(44A)-C(45A)	1.532(4)	C(44A)-C(46A)	1.534(3)
C(44A)-H(44A)	1.0000	C(45A)-H(45A)	0.9800
C(45A)-H(45B)	0.9800	C(45A)-H(45C)	0.9800
C(46A)-H(46A)	0.9800	C(46A)-H(46B)	0.9800
C(46A)-H(46C)	0.9800	C(47A)-C(48A)	1.527(4)
C(47A)-C(49A)	1.542(4)	C(47A)-H(47A)	1.0000
C(48A)-H(48A)	0.9800	C(48A)-H(48B)	0.9800
C(48A)-H(48C)	0.9800	C(49A)-H(49A)	0.9800

C(49A)-H(49B)	0.9800	C(49A)-H(49C)	0.9800
C(50A)-C(52A)	1.531(4)	C(50A)-C(51A)	1.539(4)
C(50A)-H(50A)	1.0000	C(51A)-H(51A)	0.9800
C(51A)-H(51B)	0.9800	C(51A)-H(51C)	0.9800
C(52A)-H(52A)	0.9800	C(52A)-H(52B)	0.9800
C(52A)-H(52C)	0.9800		
Si(2B)-C(50B)	1.796(7)	Si(2B)-C(44')	1.815(9)
Si(2B)-C(43B)	1.849(2)	Si(2B)-C(47B)	1.883(3)
Si(2B)-C(44B)	1.958(4)	Si(2B)-C(50')	2.153(16)
S(1B)-C(22B)	1.743(2)	S(1B)-C(1B)	1.759(2)
S(2B)-C(21B)	1.739(2)	S(2B)-C(20B)	1.7651(19)
S(3B)-C(9B)	1.735(2)	S(3B)-C(8B)	1.761(2)
S(4B)-C(11B)	1.750(2)	S(4B)-C(10B)	1.750(2)
S(5B)-C(12B)	1.7337(19)	S(5B)-C(13B)	1.766(2)
C(1B)-C(2B)	1.382(3)	C(1B)-C(9B)	1.425(3)
C(2B)-C(31B)	1.421(3)	C(2B)-C(3B)	1.471(3)
C(3B)-C(8B)	1.402(3)	C(3B)-C(4B)	1.406(3)
C(4B)-C(5B)	1.394(3)	C(4B)-H(4B)	0.9500
C(6B)-C(7B)	1.383(3)	C(6B)-C(5B)	1.396(3)
C(6B)-H(6B)	0.9500	C(7B)-C(8B)	1.389(3)
C(7B)-H(7B)	0.9500	C(9B)-C(10B)	1.371(3)
C(10B)-C(22B)	1.417(3)	C(11B)-C(12B)	1.376(3)
C(11B)-C(21B)	1.417(3)	C(12B)-C(20B)	1.423(3)
C(13B)-C(14B)	1.393(3)	C(13B)-C(18B)	1.404(3)
C(14B)-C(15B)	1.384(3)	C(14B)-H(14B)	0.9500
C(15B)-C(16B)	1.394(3)	C(15B)-H(15B)	0.9500
C(16B)-C(17B)	1.391(3)	C(16B)-C(27B)	1.539(3)
C(17B)-C(18B)	1.409(3)	C(17B)-H(17B)	0.9500
C(18B)-C(19B)	1.469(3)	C(19B)-C(20B)	1.381(3)
C(19B)-C(42B)	1.425(3)	C(21B)-C(22B)	1.371(3)
C(5B)-C(23')	1.544(5)	C(5B)-C(23B)	1.548(4)
C(23B)-C(26B)	1.522(6)	C(23B)-C(24B)	1.527(6)
C(23B)-C(25B)	1.529(7)	C(24B)-H(24D)	0.9800
C(24B)-H(24E)	0.9800	C(24B)-H(24F)	0.9800
C(25B)-H(25D)	0.9800	C(25B)-H(25E)	0.9800
C(25B)-H(25F)	0.9800	C(26B)-H(26D)	0.9800
C(26B)-H(26E)	0.9800	C(26B)-H(26F)	0.9800
C(23')-C(26')	1.518(7)	C(23')-C(24')	1.524(7)
C(23')-C(25')	1.526(8)	C(24')-H(24G)	0.9800
C(24')-H(24H)	0.9800	C(24')-H(24I)	0.9800
C(25')-H(25G)	0.9800	C(25')-H(25H)	0.9800
C(25')-H(25I)	0.9800	C(26')-H(26G)	0.9800

C(26')-H(26H)	0.9800	C(26')-H(26I)	0.9800
C(27B)-C(30B)	1.534(3)	C(27B)-C(28B)	1.535(3)
C(27B)-C(29B)	1.536(3)	C(28B)-H(28D)	0.9800
C(28B)-H(28E)	0.9800	C(28B)-H(28F)	0.9800
C(29B)-H(29D)	0.9800	C(29B)-H(29E)	0.9800
C(29B)-H(29F)	0.9800	C(30B)-H(30D)	0.9800
C(30B)-H(30E)	0.9800	C(30B)-H(30F)	0.9800
C(31B)-C(32B)	1.208(3)	C(32B)-Si(1B)	1.839(2)
C(33B)-C(34B)	1.531(4)	C(33B)-C(35B)	1.532(4)
C(33B)-Si(1B)	1.890(3)	C(33B)-H(33B)	1.0000
C(34B)-H(34D)	0.9800	C(34B)-H(34E)	0.9800
C(34B)-H(34F)	0.9800	C(35B)-H(35D)	0.9800
C(35B)-H(35E)	0.9800	C(35B)-H(35F)	0.9800
C(36B)-C(37B)	1.532(4)	C(36B)-C(38B)	1.534(4)
C(36B)-Si(1B)	1.883(3)	C(36B)-H(36B)	1.0000
C(37B)-H(37D)	0.9800	C(37B)-H(37E)	0.9800
C(37B)-H(37F)	0.9800	C(38B)-H(38D)	0.9800
C(38B)-H(38E)	0.9800	C(38B)-H(38F)	0.9800
Si(1B)-C(39')	1.896(5)	Si(1B)-C(39B)	1.908(3)
C(39B)-C(40B)	1.536(5)	C(39B)-C(41B)	1.546(5)
C(39B)-H(39B)	1.0000	C(40B)-H(40D)	0.9800
C(40B)-H(40E)	0.9800	C(40B)-H(40F)	0.9800
C(41B)-H(41D)	0.9800	C(41B)-H(41E)	0.9800
C(41B)-H(41F)	0.9800	C(39')-C(40')	1.527(6)
C(39')-C(41')	1.534(6)	C(39')-H(39')	1.0000
C(40')-H(40G)	0.9800	C(40')-H(40H)	0.9800
C(40')-H(40I)	0.9800	C(41')-H(41G)	0.9800
C(41')-H(41H)	0.9800	C(41')-H(41I)	0.9800
C(42B)-C(43B)	1.204(3)	C(44B)-C(45B)	1.364(6)
C(44B)-C(46B)	1.540(4)	C(44B)-H(44B)	1.0000
C(45B)-H(45D)	0.9800	C(45B)-H(45E)	0.9800
C(45B)-H(45F)	0.9800	C(46B)-H(46D)	0.9800
C(46B)-H(46E)	0.9800	C(46B)-H(46F)	0.9800
C(44')-C(45')	1.366(7)	C(44')-C(46')	1.545(6)
C(44')-H(44')	1.0000	C(45')-H(45G)	0.9800
C(45')-H(45H)	0.9800	C(45')-H(45I)	0.9800
C(46')-H(46G)	0.9800	C(46')-H(46H)	0.9800
C(46')-H(46I)	0.9800	C(47B)-C(49B)	1.518(4)
C(47B)-C(48B)	1.535(4)	C(47B)-H(47B)	1.0000
C(48B)-H(48D)	0.9800	C(48B)-H(48E)	0.9800
C(48B)-H(48F)	0.9800	C(49B)-H(49G)	0.9800
C(49B)-H(49H)	0.9800	C(49B)-H(49I)	0.9800
C(50B)-C(52B)	1.528(6)	C(50B)-C(51B)	1.548(5)

C(50B)-H(50B)	1.0000	C(51B)-H(51D)	0.9800
C(51B)-H(51E)	0.9800	C(51B)-H(51F)	0.9800
C(52B)-H(52D)	0.9800	C(52B)-H(52E)	0.9800
C(52B)-H(52F)	0.9800	C(50')-C(52')	1.528(7)
C(50')-C(51')	1.549(6)	C(50')-H(50')	1.0000
C(51')-H(51G)	0.9800	C(51')-H(51H)	0.9800
C(51')-H(51I)	0.9800	C(52')-H(52G)	0.9800
C(52')-H(52H)	0.9800	C(52')-H(52I)	0.9800

C(32A)-Si(1A)-C(39A)	107.37(10)	C(32A)-Si(1A)-C(36A)	108.91(11)
C(39A)-Si(1A)-C(36A)	112.16(12)	C(32A)-Si(1A)-C(33A)	105.23(10)
C(39A)-Si(1A)-C(33A)	110.75(11)	C(36A)-Si(1A)-C(33A)	112.05(11)
C(43A)-Si(2A)-C(47A)	107.80(12)	C(43A)-Si(2A)-C(50A)	104.41(11)
C(47A)-Si(2A)-C(50A)	110.62(12)	C(43A)-Si(2A)-C(44A)	107.43(11)
C(47A)-Si(2A)-C(44A)	110.85(12)	C(50A)-Si(2A)-C(44A)	115.22(12)
C(22A)-S(1A)-C(1A)	90.95(10)	C(21A)-S(2A)-C(20A)	90.81(10)
C(9A)-S(3A)-C(8A)	102.44(10)	C(11A)-S(4A)-C(10A)	89.70(10)
C(12A)-S(5A)-C(13A)	102.45(10)	C(2A)-C(1A)-C(9A)	126.6(2)
C(2A)-C(1A)-S(1A)	122.19(16)	C(9A)-C(1A)-S(1A)	111.21(15)
C(1A)-C(2A)-C(31A)	118.09(19)	C(1A)-C(2A)-C(3A)	120.95(19)
C(31A)-C(2A)-C(3A)	120.94(19)	C(8A)-C(3A)-C(4A)	117.3(2)
C(8A)-C(3A)-C(2A)	122.48(19)	C(4A)-C(3A)-C(2A)	120.2(2)
C(5A)-C(4A)-C(3A)	123.1(2)	C(5A)-C(4A)-H(4A)	118.4
C(3A)-C(4A)-H(4A)	118.4	C(6A)-C(5A)-C(4A)	117.3(2)
C(6A)-C(5A)-C(23A)	122.7(2)	C(4A)-C(5A)-C(23A)	120.0(2)
C(7A)-C(6A)-C(5A)	121.0(2)	C(7A)-C(6A)-H(6A)	119.5
C(5A)-C(6A)-H(6A)	119.5	C(6A)-C(7A)-C(8A)	120.8(2)
C(6A)-C(7A)-H(7A)	119.6	C(8A)-C(7A)-H(7A)	119.6
C(7A)-C(8A)-C(3A)	120.3(2)	C(7A)-C(8A)-S(3A)	114.93(17)
C(3A)-C(8A)-S(3A)	124.74(17)	C(10A)-C(9A)-C(1A)	112.06(19)
C(10A)-C(9A)-S(3A)	125.32(16)	C(1A)-C(9A)-S(3A)	122.60(16)
C(9A)-C(10A)-C(22A)	114.16(19)	C(9A)-C(10A)-S(4A)	133.42(17)
C(22A)-C(10A)-S(4A)	112.39(15)	C(12A)-C(11A)-C(21A)	113.75(18)
C(12A)-C(11A)-S(4A)	134.10(16)	C(21A)-C(11A)-S(4A)	112.10(15)
C(11A)-C(12A)-C(20A)	111.97(18)	C(11A)-C(12A)-S(5A)	126.02(16)
C(20A)-C(12A)-S(5A)	122.01(15)	C(14A)-C(13A)-C(18A)	119.90(19)
C(14A)-C(13A)-S(5A)	115.01(16)	C(18A)-C(13A)-S(5A)	125.08(15)
C(15A)-C(14A)-C(13A)	120.7(2)	C(15A)-C(14A)-H(14A)	119.7
C(13A)-C(14A)-H(14A)	119.7	C(14A)-C(15A)-C(16A)	121.4(2)
C(14A)-C(15A)-H(15A)	119.3	C(16A)-C(15A)-H(15A)	119.3
C(17A)-C(16A)-C(15A)	117.3(2)	C(17A)-C(16A)-C(27A)	121.1(2)
C(15A)-C(16A)-C(27A)	121.5(2)	C(16A)-C(17A)-C(18A)	123.1(2)
C(16A)-C(17A)-H(17A)	118.5	C(18A)-C(17A)-H(17A)	118.5

C(17A)-C(18A)-C(13A)	117.71(19)	C(17A)-C(18A)-C(19A)	120.54(19)
C(13A)-C(18A)-C(19A)	121.75(18)	C(20A)-C(19A)-C(42A)	116.32(18)
C(20A)-C(19A)-C(18A)	121.54(19)	C(42A)-C(19A)-C(18A)	122.14(18)
C(19A)-C(20A)-C(12A)	126.99(19)	C(19A)-C(20A)-S(2A)	121.31(16)
C(12A)-C(20A)-S(2A)	111.69(15)	C(22A)-C(21A)-C(11A)	113.00(19)
C(22A)-C(21A)-S(2A)	135.23(17)	C(11A)-C(21A)-S(2A)	111.73(15)
C(21A)-C(22A)-C(10A)	112.77(18)	C(21A)-C(22A)-S(1A)	135.59(17)
C(10A)-C(22A)-S(1A)	111.57(15)	C(26A)-C(23A)-C(24A)	110.0(3)
C(26A)-C(23A)-C(5A)	112.2(2)	C(24A)-C(23A)-C(5A)	109.3(2)
C(26A)-C(23A)-C(25A)	108.1(3)	C(24A)-C(23A)-C(25A)	108.9(2)
C(5A)-C(23A)-C(25A)	108.1(2)	C(23A)-C(24A)-H(24A)	109.5
C(23A)-C(24A)-H(24B)	109.5	H(24A)-C(24A)-H(24B)	109.5
C(23A)-C(24A)-H(24C)	109.5	H(24A)-C(24A)-H(24C)	109.5
H(24B)-C(24A)-H(24C)	109.5	C(23A)-C(25A)-H(25A)	109.5
C(23A)-C(25A)-H(25B)	109.5	H(25A)-C(25A)-H(25B)	109.5
C(23A)-C(25A)-H(25C)	109.5	H(25A)-C(25A)-H(25C)	109.5
H(25B)-C(25A)-H(25C)	109.5	C(23A)-C(26A)-H(26A)	109.5
C(23A)-C(26A)-H(26B)	109.5	H(26A)-C(26A)-H(26B)	109.5
C(23A)-C(26A)-H(26C)	109.5	H(26A)-C(26A)-H(26C)	109.5
H(26B)-C(26A)-H(26C)	109.5	C(30A)-C(27A)-C(29A)	109.5(2)
C(30A)-C(27A)-C(16A)	111.41(19)	C(29A)-C(27A)-C(16A)	108.08(19)
C(30A)-C(27A)-C(28A)	107.9(2)	C(29A)-C(27A)-C(28A)	108.5(2)
C(16A)-C(27A)-C(28A)	111.4(2)	C(27A)-C(28A)-H(28A)	109.5
C(27A)-C(28A)-H(28B)	109.5	H(28A)-C(28A)-H(28B)	109.5
C(27A)-C(28A)-H(28C)	109.5	H(28A)-C(28A)-H(28C)	109.5
H(28B)-C(28A)-H(28C)	109.5	C(27A)-C(29A)-H(29A)	109.5
C(27A)-C(29A)-H(29B)	109.5	H(29A)-C(29A)-H(29B)	109.5
C(27A)-C(29A)-H(29C)	109.5	H(29A)-C(29A)-H(29C)	109.5
H(29B)-C(29A)-H(29C)	109.5	C(27A)-C(30A)-H(30A)	109.5
C(27A)-C(30A)-H(30B)	109.5	H(30A)-C(30A)-H(30B)	109.5
C(27A)-C(30A)-H(30C)	109.5	H(30A)-C(30A)-H(30C)	109.5
H(30B)-C(30A)-H(30C)	109.5	C(32A)-C(31A)-C(2A)	176.5(2)
C(31A)-C(32A)-Si(1A)	177.1(2)	C(35A)-C(33A)-C(34A)	110.8(2)
C(35A)-C(33A)-Si(1A)	111.58(16)	C(34A)-C(33A)-Si(1A)	113.75(18)
C(35A)-C(33A)-H(33A)	106.7	C(34A)-C(33A)-H(33A)	106.7
Si(1A)-C(33A)-H(33A)	106.7	C(33A)-C(34A)-H(34A)	109.5
C(33A)-C(34A)-H(34B)	109.5	H(34A)-C(34A)-H(34B)	109.5
C(33A)-C(34A)-H(34C)	109.5	H(34A)-C(34A)-H(34C)	109.5
H(34B)-C(34A)-H(34C)	109.5	C(33A)-C(35A)-H(35A)	109.5
C(33A)-C(35A)-H(35B)	109.5	H(35A)-C(35A)-H(35B)	109.5
C(33A)-C(35A)-H(35C)	109.5	H(35A)-C(35A)-H(35C)	109.5
H(35B)-C(35A)-H(35C)	109.5	C(37A)-C(36A)-C(38A)	110.0(2)
C(37A)-C(36A)-Si(1A)	114.09(18)	C(38A)-C(36A)-Si(1A)	112.25(19)

C(37A)-C(36A)-H(36A)	106.7	C(38A)-C(36A)-H(36A)	106.7
Si(1A)-C(36A)-H(36A)	106.7	C(36A)-C(37A)-H(37A)	109.5
C(36A)-C(37A)-H(37B)	109.5	H(37A)-C(37A)-H(37B)	109.5
C(36A)-C(37A)-H(37C)	109.5	H(37A)-C(37A)-H(37C)	109.5
H(37B)-C(37A)-H(37C)	109.5	C(36A)-C(38A)-H(38A)	109.5
C(36A)-C(38A)-H(38B)	109.5	H(38A)-C(38A)-H(38B)	109.5
C(36A)-C(38A)-H(38C)	109.5	H(38A)-C(38A)-H(38C)	109.5
H(38B)-C(38A)-H(38C)	109.5	C(40A)-C(39A)-C(41A)	110.1(2)
C(40A)-C(39A)-Si(1A)	111.23(18)	C(41A)-C(39A)-Si(1A)	112.20(18)
C(40A)-C(39A)-H(39A)	107.7	C(41A)-C(39A)-H(39A)	107.7
Si(1A)-C(39A)-H(39A)	107.7	C(39A)-C(40A)-H(40A)	109.5
C(39A)-C(40A)-H(40B)	109.5	H(40A)-C(40A)-H(40B)	109.5
C(39A)-C(40A)-H(40C)	109.5	H(40A)-C(40A)-H(40C)	109.5
H(40B)-C(40A)-H(40C)	109.5	C(39A)-C(41A)-H(41A)	109.5
C(39A)-C(41A)-H(41B)	109.5	H(41A)-C(41A)-H(41B)	109.5
C(39A)-C(41A)-H(41C)	109.5	H(41A)-C(41A)-H(41C)	109.5
H(41B)-C(41A)-H(41C)	109.5	C(43A)-C(42A)-C(19A)	170.9(2)
C(42A)-C(43A)-Si(2A)	172.5(2)	C(45A)-C(44A)-C(46A)	110.6(2)
C(45A)-C(44A)-Si(2A)	111.90(18)	C(46A)-C(44A)-Si(2A)	115.56(18)
C(45A)-C(44A)-H(44A)	106.0	C(46A)-C(44A)-H(44A)	106.0
Si(2A)-C(44A)-H(44A)	106.0	C(44A)-C(45A)-H(45A)	109.5
C(44A)-C(45A)-H(45B)	109.5	H(45A)-C(45A)-H(45B)	109.5
C(44A)-C(45A)-H(45C)	109.5	H(45A)-C(45A)-H(45C)	109.5
H(45B)-C(45A)-H(45C)	109.5	C(44A)-C(46A)-H(46A)	109.5
C(44A)-C(46A)-H(46B)	109.5	H(46A)-C(46A)-H(46B)	109.5
C(44A)-C(46A)-H(46C)	109.5	H(46A)-C(46A)-H(46C)	109.5
H(46B)-C(46A)-H(46C)	109.5	C(48A)-C(47A)-C(49A)	111.3(3)
C(48A)-C(47A)-Si(2A)	112.12(19)	C(49A)-C(47A)-Si(2A)	114.0(2)
C(48A)-C(47A)-H(47A)	106.3	C(49A)-C(47A)-H(47A)	106.3
Si(2A)-C(47A)-H(47A)	106.3	C(47A)-C(48A)-H(48A)	109.5
C(47A)-C(48A)-H(48B)	109.5	H(48A)-C(48A)-H(48B)	109.5
C(47A)-C(48A)-H(48C)	109.5	H(48A)-C(48A)-H(48C)	109.5
H(48B)-C(48A)-H(48C)	109.5	C(47A)-C(49A)-H(49A)	109.5
C(47A)-C(49A)-H(49B)	109.5	H(49A)-C(49A)-H(49B)	109.5
C(47A)-C(49A)-H(49C)	109.5	H(49A)-C(49A)-H(49C)	109.5
H(49B)-C(49A)-H(49C)	109.5	C(52A)-C(50A)-C(51A)	110.2(2)
C(52A)-C(50A)-Si(2A)	109.70(19)	C(51A)-C(50A)-Si(2A)	113.09(19)
C(52A)-C(50A)-H(50A)	107.9	C(51A)-C(50A)-H(50A)	107.9
Si(2A)-C(50A)-H(50A)	107.9	C(50A)-C(51A)-H(51A)	109.5
C(50A)-C(51A)-H(51B)	109.5	H(51A)-C(51A)-H(51B)	109.5
C(50A)-C(51A)-H(51C)	109.5	H(51A)-C(51A)-H(51C)	109.5
H(51B)-C(51A)-H(51C)	109.5	C(50A)-C(52A)-H(52A)	109.5
C(50A)-C(52A)-H(52B)	109.5	H(52A)-C(52A)-H(52B)	109.5

C(50A)-C(52A)-H(52C)	109.5	H(52A)-C(52A)-H(52C)	109.5
H(52B)-C(52A)-H(52C)	109.5		

C(50B)-Si(2B)-C(43B)	108.9(2)	C(44')-Si(2B)-C(43B)	112.1(3)
C(50B)-Si(2B)-C(47B)	115.5(2)	C(44')-Si(2B)-C(47B)	121.9(4)
C(43B)-Si(2B)-C(47B)	108.61(12)	C(50B)-Si(2B)-C(44B)	113.9(2)
C(43B)-Si(2B)-C(44B)	105.41(13)	C(47B)-Si(2B)-C(44B)	104.00(14)
C(44')-Si(2B)-C(50')	100.6(6)	C(43B)-Si(2B)-C(50')	103.3(4)
C(47B)-Si(2B)-C(50')	108.5(5)	C(22B)-S(1B)-C(1B)	91.03(10)
C(21B)-S(2B)-C(20B)	90.99(10)	C(9B)-S(3B)-C(8B)	102.04(10)
C(11B)-S(4B)-C(10B)	89.56(10)	C(12B)-S(5B)-C(13B)	101.98(10)
C(2B)-C(1B)-C(9B)	126.0(2)	C(2B)-C(1B)-S(1B)	122.54(16)
C(9B)-C(1B)-S(1B)	111.42(15)	C(1B)-C(2B)-C(31B)	119.87(19)
C(1B)-C(2B)-C(3B)	120.53(19)	C(31B)-C(2B)-C(3B)	119.57(18)
C(8B)-C(3B)-C(4B)	117.6(2)	C(8B)-C(3B)-C(2B)	123.12(19)
C(4B)-C(3B)-C(2B)	119.29(19)	C(5B)-C(4B)-C(3B)	122.8(2)
C(5B)-C(4B)-H(4B)	118.6	C(3B)-C(4B)-H(4B)	118.6
C(7B)-C(6B)-C(5B)	121.0(2)	C(7B)-C(6B)-H(6B)	119.5
C(5B)-C(6B)-H(6B)	119.5	C(6B)-C(7B)-C(8B)	120.8(2)
C(6B)-C(7B)-H(7B)	119.6	C(8B)-C(7B)-H(7B)	119.6
C(7B)-C(8B)-C(3B)	120.2(2)	C(7B)-C(8B)-S(3B)	115.56(17)
C(3B)-C(8B)-S(3B)	124.16(17)	C(10B)-C(9B)-C(1B)	112.04(19)
C(10B)-C(9B)-S(3B)	124.83(16)	C(1B)-C(9B)-S(3B)	123.03(16)
C(9B)-C(10B)-C(22B)	114.72(18)	C(9B)-C(10B)-S(4B)	132.74(17)
C(22B)-C(10B)-S(4B)	112.55(15)	C(12B)-C(11B)-C(21B)	114.32(18)
C(12B)-C(11B)-S(4B)	133.18(16)	C(21B)-C(11B)-S(4B)	112.49(15)
C(11B)-C(12B)-C(20B)	112.26(17)	C(11B)-C(12B)-S(5B)	124.97(16)
C(20B)-C(12B)-S(5B)	122.72(15)	C(14B)-C(13B)-C(18B)	120.49(19)
C(14B)-C(13B)-S(5B)	114.61(16)	C(18B)-C(13B)-S(5B)	124.89(15)
C(15B)-C(14B)-C(13B)	120.21(19)	C(15B)-C(14B)-H(14B)	119.9
C(13B)-C(14B)-H(14B)	119.9	C(14B)-C(15B)-C(16B)	121.45(19)
C(14B)-C(15B)-H(15B)	119.3	C(16B)-C(15B)-H(15B)	119.3
C(17B)-C(16B)-C(15B)	117.52(19)	C(17B)-C(16B)-C(27B)	121.94(19)
C(15B)-C(16B)-C(27B)	120.39(18)	C(16B)-C(17B)-C(18B)	122.9(2)
C(16B)-C(17B)-H(17B)	118.5	C(18B)-C(17B)-H(17B)	118.5
C(13B)-C(18B)-C(17B)	117.37(18)	C(13B)-C(18B)-C(19B)	122.40(18)
C(17B)-C(18B)-C(19B)	120.22(18)	C(20B)-C(19B)-C(42B)	118.16(18)
C(20B)-C(19B)-C(18B)	120.55(18)	C(42B)-C(19B)-C(18B)	121.29(19)
C(19B)-C(20B)-C(12B)	126.80(18)	C(19B)-C(20B)-S(2B)	121.95(16)
C(12B)-C(20B)-S(2B)	111.25(14)	C(22B)-C(21B)-C(11B)	112.77(19)
C(22B)-C(21B)-S(2B)	136.06(16)	C(11B)-C(21B)-S(2B)	111.17(15)
C(21B)-C(22B)-C(10B)	112.64(18)	C(21B)-C(22B)-S(1B)	136.58(17)
C(10B)-C(22B)-S(1B)	110.77(15)	C(4B)-C(5B)-C(6B)	117.6(2)

C(4B)-C(5B)-C(23')	120.8(4)	C(6B)-C(5B)-C(23')	120.6(4)
C(4B)-C(5B)-C(23B)	120.5(3)	C(6B)-C(5B)-C(23B)	121.7(3)
C(26B)-C(23B)-C(24B)	108.1(5)	C(26B)-C(23B)-C(25B)	109.2(4)
C(24B)-C(23B)-C(25B)	107.8(4)	C(26B)-C(23B)-C(5B)	109.2(3)
C(24B)-C(23B)-C(5B)	113.0(3)	C(25B)-C(23B)-C(5B)	109.6(4)
C(23B)-C(24B)-H(24D)	109.5	C(23B)-C(24B)-H(24E)	109.5
H(24D)-C(24B)-H(24E)	109.5	C(23B)-C(24B)-H(24F)	109.5
H(24D)-C(24B)-H(24F)	109.5	H(24E)-C(24B)-H(24F)	109.5
C(23B)-C(25B)-H(25D)	109.5	C(23B)-C(25B)-H(25E)	109.5
H(25D)-C(25B)-H(25E)	109.5	C(23B)-C(25B)-H(25F)	109.5
H(25D)-C(25B)-H(25F)	109.5	H(25E)-C(25B)-H(25F)	109.5
C(23B)-C(26B)-H(26D)	109.5	C(23B)-C(26B)-H(26E)	109.5
H(26D)-C(26B)-H(26E)	109.5	C(23B)-C(26B)-H(26F)	109.5
H(26D)-C(26B)-H(26F)	109.5	H(26E)-C(26B)-H(26F)	109.5
C(26')-C(23')-C(24')	109.4(6)	C(26')-C(23')-C(25')	110.0(6)
C(24')-C(23')-C(25')	109.6(6)	C(26')-C(23')-C(5B)	110.6(5)
C(24')-C(23')-C(5B)	111.8(5)	C(25')-C(23')-C(5B)	105.4(5)
C(23')-C(24')-H(24G)	109.5	C(23')-C(24')-H(24H)	109.5
H(24G)-C(24')-H(24H)	109.5	C(23')-C(24')-H(24I)	109.5
H(24G)-C(24')-H(24I)	109.5	H(24H)-C(24')-H(24I)	109.5
C(23')-C(25')-H(25G)	109.5	C(23')-C(25')-H(25H)	109.5
H(25G)-C(25')-H(25H)	109.5	C(23')-C(25')-H(25I)	109.5
H(25G)-C(25')-H(25I)	109.5	H(25H)-C(25')-H(25I)	109.5
C(23')-C(26')-H(26G)	109.5	C(23')-C(26')-H(26H)	109.5
H(26G)-C(26')-H(26H)	109.5	C(23')-C(26')-H(26I)	109.5
H(26G)-C(26')-H(26I)	109.5	H(26H)-C(26')-H(26I)	109.5
C(30B)-C(27B)-C(28B)	109.5(2)	C(30B)-C(27B)-C(29B)	107.7(2)
C(28B)-C(27B)-C(29B)	108.9(2)	C(30B)-C(27B)-C(16B)	111.52(17)
C(28B)-C(27B)-C(16B)	107.96(18)	C(29B)-C(27B)-C(16B)	111.2(2)
C(27B)-C(28B)-H(28D)	109.5	C(27B)-C(28B)-H(28E)	109.5
H(28D)-C(28B)-H(28E)	109.5	C(27B)-C(28B)-H(28F)	109.5
H(28D)-C(28B)-H(28F)	109.5	H(28E)-C(28B)-H(28F)	109.5
C(27B)-C(29B)-H(29D)	109.5	C(27B)-C(29B)-H(29E)	109.5
H(29D)-C(29B)-H(29E)	109.5	C(27B)-C(29B)-H(29F)	109.5
H(29D)-C(29B)-H(29F)	109.5	H(29E)-C(29B)-H(29F)	109.5
C(27B)-C(30B)-H(30D)	109.5	C(27B)-C(30B)-H(30E)	109.5
H(30D)-C(30B)-H(30E)	109.5	C(27B)-C(30B)-H(30F)	109.5
H(30D)-C(30B)-H(30F)	109.5	H(30E)-C(30B)-H(30F)	109.5
C(32B)-C(31B)-C(2B)	178.4(2)	C(31B)-C(32B)-Si(1B)	175.9(2)
C(34B)-C(33B)-C(35B)	111.4(3)	C(34B)-C(33B)-Si(1B)	114.9(2)
C(35B)-C(33B)-Si(1B)	111.3(2)	C(34B)-C(33B)-H(33B)	106.2
C(35B)-C(33B)-H(33B)	106.2	Si(1B)-C(33B)-H(33B)	106.2
C(33B)-C(34B)-H(34D)	109.5	C(33B)-C(34B)-H(34E)	109.5

H(34D)-C(34B)-H(34E)	109.5	C(33B)-C(34B)-H(34F)	109.5
H(34D)-C(34B)-H(34F)	109.5	H(34E)-C(34B)-H(34F)	109.5
C(33B)-C(35B)-H(35D)	109.5	C(33B)-C(35B)-H(35E)	109.5
H(35D)-C(35B)-H(35E)	109.5	C(33B)-C(35B)-H(35F)	109.5
H(35D)-C(35B)-H(35F)	109.5	H(35E)-C(35B)-H(35F)	109.5
C(37B)-C(36B)-C(38B)	109.4(2)	C(37B)-C(36B)-Si(1B)	111.4(2)
C(38B)-C(36B)-Si(1B)	111.57(19)	C(37B)-C(36B)-H(36B)	108.1
C(38B)-C(36B)-H(36B)	108.1	Si(1B)-C(36B)-H(36B)	108.1
C(36B)-C(37B)-H(37D)	109.5	C(36B)-C(37B)-H(37E)	109.5
H(37D)-C(37B)-H(37E)	109.5	C(36B)-C(37B)-H(37F)	109.5
H(37D)-C(37B)-H(37F)	109.5	H(37E)-C(37B)-H(37F)	109.5
C(36B)-C(38B)-H(38D)	109.5	C(36B)-C(38B)-H(38E)	109.5
H(38D)-C(38B)-H(38E)	109.5	C(36B)-C(38B)-H(38F)	109.5
H(38D)-C(38B)-H(38F)	109.5	H(38E)-C(38B)-H(38F)	109.5
C(32B)-Si(1B)-C(36B)	106.04(11)	C(32B)-Si(1B)-C(33B)	104.23(11)
C(36B)-Si(1B)-C(33B)	112.06(13)	C(32B)-Si(1B)-C(39')	103.5(3)
C(36B)-Si(1B)-C(39')	124.7(4)	C(33B)-Si(1B)-C(39')	104.3(4)
C(32B)-Si(1B)-C(39B)	109.36(15)	C(36B)-Si(1B)-C(39B)	107.20(15)
C(33B)-Si(1B)-C(39B)	117.37(15)	C(40B)-C(39B)-C(41B)	108.3(4)
C(40B)-C(39B)-Si(1B)	113.2(3)	C(41B)-C(39B)-Si(1B)	111.6(3)
C(40B)-C(39B)-H(39B)	107.9	C(41B)-C(39B)-H(39B)	107.9
Si(1B)-C(39B)-H(39B)	107.9	C(39B)-C(40B)-H(40D)	109.5
C(39B)-C(40B)-H(40E)	109.5	H(40D)-C(40B)-H(40E)	109.5
C(39B)-C(40B)-H(40F)	109.5	H(40D)-C(40B)-H(40F)	109.5
H(40E)-C(40B)-H(40F)	109.5	C(39B)-C(41B)-H(41D)	109.5
C(39B)-C(41B)-H(41E)	109.5	H(41D)-C(41B)-H(41E)	109.5
C(39B)-C(41B)-H(41F)	109.5	H(41D)-C(41B)-H(41F)	109.5
H(41E)-C(41B)-H(41F)	109.5	C(40')-C(39')-C(41')	111.4(7)
C(40')-C(39')-Si(1B)	114.5(6)	C(41')-C(39')-Si(1B)	112.3(6)
C(40')-C(39')-H(39')	105.9	C(41')-C(39')-H(39')	105.9
Si(1B)-C(39')-H(39')	105.9	C(39')-C(40')-H(40G)	109.5
C(39')-C(40')-H(40H)	109.5	H(40G)-C(40')-H(40H)	109.5
C(39')-C(40')-H(40I)	109.5	H(40G)-C(40')-H(40I)	109.5
H(40H)-C(40')-H(40I)	109.5	C(39')-C(41')-H(41G)	109.5
C(39')-C(41')-H(41H)	109.5	H(41G)-C(41')-H(41H)	109.5
C(39')-C(41')-H(41I)	109.5	H(41G)-C(41')-H(41I)	109.5
H(41H)-C(41')-H(41I)	109.5	C(43B)-C(42B)-C(19B)	173.7(2)
C(42B)-C(43B)-Si(2B)	177.4(2)	C(45B)-C(44B)-C(46B)	116.3(4)
C(45B)-C(44B)-Si(2B)	114.3(4)	C(46B)-C(44B)-Si(2B)	112.8(3)
C(45B)-C(44B)-H(44B)	103.8	C(46B)-C(44B)-H(44B)	103.8
Si(2B)-C(44B)-H(44B)	103.8	C(44B)-C(45B)-H(45D)	109.5
C(44B)-C(45B)-H(45E)	109.5	H(45D)-C(45B)-H(45E)	109.5
C(44B)-C(45B)-H(45F)	109.5	H(45D)-C(45B)-H(45F)	109.5

H(45E)-C(45B)-H(45F)	109.5	C(44B)-C(46B)-H(46D)	109.5
C(44B)-C(46B)-H(46E)	109.5	H(46D)-C(46B)-H(46E)	109.5
C(44B)-C(46B)-H(46F)	109.5	H(46D)-C(46B)-H(46F)	109.5
H(46E)-C(46B)-H(46F)	109.5	C(45')-C(44')-C(46')	114.9(7)
C(45')-C(44')-Si(2B)	130.8(7)	C(46')-C(44')-Si(2B)	111.0(8)
C(45')-C(44')-H(44')	95.9	C(46')-C(44')-H(44')	95.9
Si(2B)-C(44')-H(44')	95.9	C(44')-C(45')-H(45G)	109.5
C(44')-C(45')-H(45H)	109.5	H(45G)-C(45')-H(45H)	109.5
C(44')-C(45')-H(45I)	109.5	H(45G)-C(45')-H(45I)	109.5
H(45H)-C(45')-H(45I)	109.5	C(44')-C(46')-H(46G)	109.5
C(44')-C(46')-H(46H)	109.5	H(46G)-C(46')-H(46H)	109.5
C(44')-C(46')-H(46I)	109.5	H(46G)-C(46')-H(46I)	109.5
H(46H)-C(46')-H(46I)	109.5	C(49B)-C(47B)-C(48B)	109.7(3)
C(49B)-C(47B)-Si(2B)	114.90(19)	C(48B)-C(47B)-Si(2B)	110.8(2)
C(49B)-C(47B)-H(47B)	107.0	C(48B)-C(47B)-H(47B)	107.0
Si(2B)-C(47B)-H(47B)	107.0	C(47B)-C(48B)-H(48D)	109.5
C(47B)-C(48B)-H(48E)	109.5	H(48D)-C(48B)-H(48E)	109.5
C(47B)-C(48B)-H(48F)	109.5	H(48D)-C(48B)-H(48F)	109.5
H(48E)-C(48B)-H(48F)	109.5	C(47B)-C(49B)-H(49G)	109.5
C(47B)-C(49B)-H(49H)	109.5	H(49G)-C(49B)-H(49H)	109.5
C(47B)-C(49B)-H(49I)	109.5	H(49G)-C(49B)-H(49I)	109.5
H(49H)-C(49B)-H(49I)	109.5	C(52B)-C(50B)-C(51B)	108.9(4)
C(52B)-C(50B)-Si(2B)	113.2(5)	C(51B)-C(50B)-Si(2B)	114.7(5)
C(52B)-C(50B)-H(50B)	106.5	C(51B)-C(50B)-H(50B)	106.5
Si(2B)-C(50B)-H(50B)	106.5	C(50B)-C(51B)-H(51D)	109.5
C(50B)-C(51B)-H(51E)	109.5	H(51D)-C(51B)-H(51E)	109.5
C(50B)-C(51B)-H(51F)	109.5	H(51D)-C(51B)-H(51F)	109.5
H(51E)-C(51B)-H(51F)	109.5	C(50B)-C(52B)-H(52D)	109.5
C(50B)-C(52B)-H(52E)	109.5	H(52D)-C(52B)-H(52E)	109.5
C(50B)-C(52B)-H(52F)	109.5	H(52D)-C(52B)-H(52F)	109.5
H(52E)-C(52B)-H(52F)	109.5	C(52')-C(50')-C(51')	108.2(7)
C(52')-C(50')-Si(2B)	116.7(12)	C(51')-C(50')-Si(2B)	111.4(12)
C(52')-C(50')-H(50')	106.7	C(51')-C(50')-H(50')	106.7
Si(2B)-C(50')-H(50')	106.7	C(50')-C(51')-H(51G)	109.5
C(50')-C(51')-H(51H)	109.5	H(51G)-C(51')-H(51H)	109.5
C(50')-C(51')-H(51I)	109.5	H(51G)-C(51')-H(51I)	109.5
H(51H)-C(51')-H(51I)	109.5	C(50')-C(52')-H(52G)	109.5
C(50')-C(52')-H(52H)	109.5	H(52G)-C(52')-H(52H)	109.5
C(50')-C(52')-H(52I)	109.5	H(52G)-C(52')-H(52I)	109.5
H(52H)-C(52')-H(52I)	109.5		

Table S16. Anisotropic displacement parameters ($Å^2$x 10^3) for **Th3-TIPS**. The anisotropic displacement factor exponent takes the form: $-2\pi^2[h^2a^{*2}U^{11} + ... + 2 h k a^* b^* U^{12}]$

	U^{11}	U^{22}	U^{33}	U^{23}	U^{13}	U^{12}
Si(1A)	24(1)	23(1)	26(1)	1(1)	2(1)	-1(1)
Si(2A)	34(1)	24(1)	29(1)	7(1)	4(1)	2(1)
S(1A)	18(1)	21(1)	21(1)	2(1)	1(1)	0(1)
S(2A)	18(1)	20(1)	23(1)	4(1)	1(1)	2(1)
S(3A)	40(1)	26(1)	26(1)	7(1)	-3(1)	-7(1)
S(4A)	19(1)	20(1)	22(1)	2(1)	1(1)	1(1)
S(5A)	16(1)	21(1)	22(1)	2(1)	1(1)	2(1)
C(1A)	17(1)	22(1)	25(1)	4(1)	3(1)	4(1)
C(2A)	19(1)	25(1)	24(1)	4(1)	3(1)	5(1)
C(3A)	21(1)	25(1)	23(1)	2(1)	2(1)	7(1)
C(4A)	25(1)	24(1)	25(1)	0(1)	0(1)	6(1)
C(5A)	40(1)	26(1)	24(1)	1(1)	-2(1)	9(1)
C(6A)	53(2)	30(1)	25(1)	8(1)	3(1)	6(1)
C(7A)	43(1)	27(1)	29(1)	6(1)	3(1)	1(1)
C(8A)	28(1)	26(1)	21(1)	2(1)	2(1)	3(1)
C(9A)	19(1)	21(1)	25(1)	3(1)	4(1)	4(1)
C(10A)	17(1)	23(1)	23(1)	2(1)	4(1)	5(1)
C(11A)	16(1)	20(1)	24(1)	2(1)	5(1)	4(1)
C(12A)	14(1)	22(1)	22(1)	1(1)	3(1)	5(1)
C(13A)	14(1)	27(1)	22(1)	2(1)	4(1)	7(1)
C(14A)	20(1)	27(1)	25(1)	0(1)	6(1)	4(1)
C(15A)	18(1)	35(1)	24(1)	-5(1)	3(1)	4(1)
C(16A)	19(1)	38(1)	22(1)	1(1)	4(1)	11(1)
C(17A)	20(1)	30(1)	26(1)	5(1)	4(1)	8(1)
C(18A)	17(1)	27(1)	21(1)	3(1)	4(1)	7(1)
C(19A)	17(1)	24(1)	23(1)	5(1)	3(1)	6(1)
C(20A)	14(1)	20(1)	25(1)	0(1)	5(1)	3(1)
C(21A)	16(1)	21(1)	23(1)	3(1)	4(1)	4(1)
C(22A)	16(1)	22(1)	24(1)	1(1)	4(1)	3(1)
C(23A)	59(2)	28(1)	28(1)	3(1)	-10(1)	6(1)
C(24A)	67(2)	41(2)	33(1)	-3(1)	-2(1)	13(1)
C(25A)	58(2)	64(2)	49(2)	-10(2)	-26(2)	24(2)
C(26A)	145(4)	43(2)	28(2)	10(1)	-31(2)	-14(2)
C(27A)	25(1)	49(2)	22(1)	5(1)	1(1)	13(1)
C(28A)	38(1)	58(2)	24(1)	0(1)	-6(1)	5(1)
C(29A)	38(1)	80(2)	31(1)	15(1)	2(1)	33(1)

C(30A)	42(1)	57(2)	26(1)	11(1)	3(1)	11(1)
C(31A)	21(1)	30(1)	20(1)	2(1)	0(1)	5(1)
C(32A)	27(1)	27(1)	23(1)	3(1)	0(1)	0(1)
C(33A)	25(1)	30(1)	36(1)	-1(1)	0(1)	2(1)
C(34A)	27(1)	48(2)	54(2)	0(1)	2(1)	-6(1)
C(35A)	32(1)	51(2)	38(1)	6(1)	-3(1)	6(1)
C(36A)	36(1)	33(1)	36(1)	0(1)	5(1)	9(1)
C(37A)	40(2)	49(2)	60(2)	0(1)	10(1)	15(1)
C(38A)	53(2)	30(1)	63(2)	1(1)	4(1)	8(1)
C(39A)	41(1)	31(1)	30(1)	5(1)	6(1)	-6(1)
C(40A)	67(2)	49(2)	35(2)	-4(1)	20(1)	3(1)
C(41A)	54(2)	55(2)	31(1)	12(1)	-4(1)	-8(1)
C(42A)	21(1)	31(1)	23(1)	4(1)	1(1)	6(1)
C(43A)	30(1)	30(1)	27(1)	8(1)	3(1)	4(1)
C(44A)	34(1)	36(1)	38(1)	6(1)	3(1)	4(1)
C(45A)	44(2)	35(2)	69(2)	10(1)	-6(1)	-4(1)
C(46A)	31(1)	46(2)	57(2)	19(1)	6(1)	5(1)
C(47A)	41(1)	35(1)	41(2)	4(1)	-6(1)	11(1)
C(48A)	74(2)	95(3)	55(2)	-15(2)	-3(2)	55(2)
C(49A)	63(2)	32(2)	73(2)	16(2)	-9(2)	10(1)
C(50A)	53(2)	31(1)	33(1)	8(1)	10(1)	-4(1)
C(51A)	63(2)	35(2)	46(2)	0(1)	24(1)	-1(1)
C(52A)	66(2)	50(2)	30(1)	6(1)	5(1)	-7(1)
Si(2B)	34(1)	47(1)	39(1)	23(1)	9(1)	20(1)
S(1B)	19(1)	22(1)	19(1)	0(1)	0(1)	2(1)
S(2B)	18(1)	24(1)	19(1)	1(1)	0(1)	4(1)
S(3B)	35(1)	25(1)	25(1)	4(1)	-2(1)	1(1)
S(4B)	17(1)	23(1)	21(1)	2(1)	1(1)	3(1)
S(5B)	16(1)	21(1)	19(1)	1(1)	0(1)	3(1)
C(1B)	19(1)	22(1)	23(1)	3(1)	5(1)	3(1)
C(2B)	19(1)	25(1)	21(1)	-1(1)	2(1)	2(1)
C(3B)	20(1)	24(1)	26(1)	2(1)	4(1)	2(1)
C(4B)	24(1)	26(1)	29(1)	0(1)	3(1)	2(1)
C(6B)	32(1)	22(1)	45(1)	5(1)	3(1)	5(1)
C(7B)	30(1)	30(1)	35(1)	6(1)	2(1)	8(1)
C(8B)	23(1)	24(1)	29(1)	1(1)	3(1)	2(1)
C(9B)	19(1)	23(1)	22(1)	1(1)	3(1)	3(1)
C(10B)	16(1)	25(1)	21(1)	1(1)	3(1)	4(1)
C(11B)	15(1)	24(1)	21(1)	2(1)	5(1)	6(1)
C(12B)	14(1)	24(1)	18(1)	1(1)	3(1)	5(1)
C(13B)	14(1)	20(1)	22(1)	0(1)	4(1)	2(1)
C(14B)	18(1)	24(1)	22(1)	4(1)	2(1)	5(1)
C(15B)	17(1)	26(1)	22(1)	-1(1)	0(1)	4(1)

C(16B)	16(1)	24(1)	26(1)	-2(1)	3(1)	3(1)
C(17B)	19(1)	21(1)	27(1)	4(1)	3(1)	5(1)
C(18B)	15(1)	24(1)	21(1)	1(1)	2(1)	4(1)
C(19B)	17(1)	24(1)	23(1)	4(1)	2(1)	5(1)
C(20B)	13(1)	26(1)	18(1)	1(1)	1(1)	5(1)
C(21B)	15(1)	24(1)	21(1)	1(1)	4(1)	5(1)
C(22B)	14(1)	26(1)	20(1)	0(1)	3(1)	3(1)
C(5B)	27(1)	27(1)	38(1)	-1(1)	5(1)	4(1)
C(23B)	34(2)	22(2)	39(3)	0(2)	1(2)	-2(2)
C(24B)	98(4)	32(2)	48(3)	-10(2)	-30(3)	4(2)
C(25B)	61(3)	36(2)	65(3)	-17(2)	28(2)	-7(2)
C(26B)	52(3)	46(3)	46(2)	-2(2)	9(2)	-19(2)
C(23')	31(6)	36(7)	38(6)	0(4)	10(5)	3(4)
C(24')	34(4)	26(4)	48(5)	-9(4)	-2(4)	-4(3)
C(25')	52(6)	48(6)	29(5)	-5(4)	11(4)	-8(5)
C(26')	47(6)	24(5)	52(6)	-9(4)	-13(5)	-1(4)
C(27B)	23(1)	23(1)	33(1)	1(1)	-1(1)	0(1)
C(28B)	24(1)	29(1)	65(2)	9(1)	7(1)	1(1)
C(29B)	54(2)	29(1)	39(2)	-3(1)	-9(1)	-6(1)
C(30B)	28(1)	23(1)	50(2)	3(1)	-1(1)	3(1)
C(31B)	23(1)	20(1)	25(1)	-3(1)	4(1)	-1(1)
C(32B)	29(1)	26(1)	26(1)	-1(1)	2(1)	0(1)
C(33B)	58(2)	29(1)	33(1)	0(1)	-14(1)	5(1)
C(34B)	106(3)	38(2)	37(2)	-12(1)	-28(2)	19(2)
C(35B)	65(2)	48(2)	75(2)	25(2)	-26(2)	-20(2)
C(36B)	35(1)	47(2)	30(1)	15(1)	3(1)	11(1)
C(37B)	52(2)	89(3)	59(2)	25(2)	29(2)	28(2)
C(38B)	42(2)	51(2)	53(2)	21(1)	-4(1)	-10(1)
Si(1B)	26(1)	28(1)	22(1)	-1(1)	0(1)	6(1)
C(39B)	18(2)	28(2)	30(2)	3(2)	6(1)	7(1)
C(40B)	31(3)	64(4)	43(4)	-11(3)	-12(2)	29(3)
C(41B)	59(3)	128(6)	33(2)	19(3)	19(2)	61(4)
C(39')	54(8)	71(11)	70(11)	-25(8)	-6(7)	16(8)
C(40')	94(15)	36(8)	68(13)	33(8)	47(10)	3(7)
C(41')	93(12)	60(8)	72(9)	17(7)	33(8)	42(8)
C(42B)	21(1)	25(1)	24(1)	1(1)	1(1)	3(1)
C(43B)	30(1)	33(1)	33(1)	9(1)	1(1)	9(1)
C(44B)	26(2)	35(2)	30(2)	7(2)	-1(1)	2(2)
C(45B)	135(6)	121(6)	63(4)	-9(4)	-7(4)	73(5)
C(46B)	43(2)	64(4)	32(2)	20(2)	2(2)	16(3)
C(44')	65(7)	46(7)	46(6)	21(5)	4(5)	19(6)
C(45')	14(2)	17(2)	16(2)	2(1)	3(1)	1(1)
C(46')	57(5)	57(5)	57(5)	9(1)	10(1)	10(1)

C(47B)	32(1)	53(2)	37(1)	16(1)	1(1)	13(1)
C(48B)	50(2)	72(2)	48(2)	15(2)	-11(1)	-3(2)
C(49B)	34(1)	74(2)	58(2)	12(2)	12(1)	17(1)
C(50B)	37(2)	29(3)	39(3)	7(2)	5(3)	5(2)
C(51B)	77(4)	30(3)	74(4)	28(3)	5(4)	9(3)
C(52B)	50(3)	30(3)	48(4)	1(3)	6(3)	1(2)
C(50')	42(6)	33(8)	40(10)	9(6)	-6(7)	7(5)
C(51')	88(10)	76(11)	86(10)	31(8)	3(8)	16(8)
C(52')	84(12)	41(8)	41(9)	7(7)	-2(7)	-9(7)

Table S17. Hydrogen coordinates (x 10^4) and isotropic displacement parameters (Å^{2}x 10^3) for **Th3-TIPS**.

	x	y	z	U(eq)
H(4A)	2499	1721	9796	31
H(6A)	1446	-43	10649	44
H(7A)	518	-532	9572	40
H(14A)	-3195	-1582	3477	29
H(15A)	-3853	-1370	2370	32
H(17A)	-2401	716	2738	30
H(24A)	2693	2298	10895	72
H(24B)	3058	2203	11695	72
H(24C)	1990	1970	11369	72
H(25A)	3841	1577	10527	91
H(25B)	3830	740	10689	91
H(25C)	4180	1424	11314	91
H(26A)	3095	936	12029	120
H(26B)	2756	216	11443	120
H(26C)	2027	688	11706	120
H(28A)	-3572	-1131	1160	63
H(28B)	-4314	-721	746	63
H(28C)	-4559	-1154	1381	63
H(29A)	-4941	-101	2073	71
H(29B)	-4826	369	1437	71
H(29C)	-4288	696	2218	71
H(30A)	-2875	870	1684	62
H(30B)	-3380	450	921	62
H(30C)	-2515	166	1329	62
H(33A)	4684	3069	8921	38
H(34A)	5155	4634	9389	68
H(34B)	5320	4238	8650	68
H(34C)	5866	4067	9375	68

H(35A)	5036	3313	10152	62
H(35B)	3968	2976	9911	62
H(35C)	4291	3851	10168	62
H(36A)	3074	4422	9610	43
H(37A)	1591	4644	9256	74
H(37B)	1585	3767	9115	74
H(37C)	1557	4242	8472	74
H(38A)	2986	5166	8389	75
H(38B)	3879	5257	8986	75
H(38C)	2980	5548	9173	75
H(39A)	3829	4245	7706	42
H(40A)	3602	2659	7375	76
H(40B)	4564	3207	7639	76
H(40C)	3977	3231	6880	76
H(41A)	2587	3782	6757	74
H(41B)	2226	4132	7435	74
H(41C)	2116	3247	7243	74
H(44A)	683	3372	4567	44
H(45A)	2008	4026	4314	79
H(45B)	1146	4242	3834	79
H(45C)	1731	3679	3498	79
H(46A)	1683	2404	3874	66
H(46B)	990	2135	4379	66
H(46C)	1890	2741	4694	66
H(47A)	-1635	3350	3339	48
H(48A)	-302	4265	4428	111
H(48B)	-1086	3621	4548	111
H(48C)	-1357	4352	4253	111
H(49A)	-1270	4551	3030	86
H(49B)	-960	3944	2499	86
H(49C)	-213	4470	3105	86
H(50A)	116	3099	2310	48
H(51A)	540	1708	2684	72
H(51B)	1271	2435	2668	72
H(51C)	676	1980	1946	72
H(52A)	-913	2126	1561	76
H(52B)	-1452	2548	2088	76
H(52C)	-1181	1760	2228	76
H(4B)	2600	5897	1678	32
H(6B)	3205	4355	2920	40
H(7B)	4012	5294	3801	38
H(14B)	8237	11033	6953	26
H(15B)	9047	12242	7238	27

H(17B)	7721	12789	5448	27
H(24D)	2226	5049	765	99
H(24E)	1353	4948	1149	99
H(24F)	1520	4278	604	99
H(25D)	3468	3828	1648	84
H(25E)	3547	4436	1121	84
H(25F)	2844	3667	879	84
H(26D)	1441	3431	1438	77
H(26E)	1282	4042	2044	77
H(26F)	2103	3584	2191	77
H(24G)	1096	4999	1465	59
H(24H)	1005	4516	2095	59
H(24I)	769	4116	1293	59
H(25G)	3100	4457	982	68
H(25H)	2360	4986	821	68
H(25I)	2080	4104	589	68
H(26G)	1907	3268	1470	68
H(26H)	2027	3586	2290	68
H(26I)	2908	3597	1917	68
H(28D)	10294	13051	6430	59
H(28E)	10309	13893	6282	59
H(28F)	9756	13218	5700	59
H(29D)	8820	13621	7561	67
H(29E)	9691	14176	7433	67
H(29F)	9770	13333	7533	67
H(30D)	8389	13886	5728	52
H(30E)	8962	14502	6358	52
H(30F)	8010	14029	6450	52
H(33B)	1977	6228	66	52
H(34D)	986	5881	-1007	98
H(34E)	1633	6654	-1029	98
H(34F)	595	6645	-921	98
H(35D)	146	6324	242	103
H(35E)	920	6177	854	103
H(35F)	570	5576	160	103
H(36B)	2422	8081	-500	44
H(37D)	3928	7814	-412	93
H(37E)	3211	7063	-457	93
H(37F)	3820	7446	286	93
H(38D)	3451	8697	872	76
H(38E)	2739	9085	400	76
H(38F)	3693	8983	163	76
H(39B)	1294	8639	561	30

H(40D)	-196	7445	-22	71
H(40E)	357	7936	-499	71
H(40F)	-234	8315	14	71
H(41D)	255	8422	1334	100
H(41E)	1187	8160	1650	100
H(41F)	320	7555	1256	100
H(39')	448	7419	811	83
H(40G)	-339	8197	165	92
H(40H)	11	7599	-350	92
H(40I)	541	8442	-181	92
H(41G)	1520	8868	1173	103
H(41H)	1263	8291	1700	103
H(41I)	490	8691	1312	103
H(44B)	5316	12124	2477	37
H(45D)	6509	12126	1972	159
H(45E)	6731	12081	2800	159
H(45F)	7038	12845	2510	159
H(46D)	5630	13519	1990	68
H(46E)	4659	13011	1976	68
H(46F)	5336	12742	1476	68
H(44')	6600	13483	2764	61
H(45G)	6436	12230	2003	23
H(45H)	6962	12296	2799	23
H(45I)	7272	12899	2311	23
H(46G)	5295	13708	2241	86
H(46H)	4976	12861	1876	86
H(46I)	5837	13363	1670	86
H(47B)	4210	13329	3113	48
H(48D)	4168	11950	3669	90
H(48E)	4049	12028	2847	90
H(48F)	3286	12236	3290	90
H(49G)	3573	13330	4153	81
H(49H)	4485	13939	4255	81
H(49I)	4517	13174	4571	81
H(50B)	6992	13973	3524	42
H(51D)	5398	14661	3485	90
H(51E)	5902	14494	2829	90
H(51F)	6408	15098	3492	90
H(52D)	7082	14738	4585	66
H(52E)	6957	13888	4721	66
H(52F)	6104	14320	4687	66
H(50')	7073	14220	3868	47
H(51G)	6106	15254	3930	124

H(51H)	5369	14631	3404	124
H(51I)	6342	14912	3196	124
H(52G)	6937	14833	4924	88
H(52H)	6664	13967	4983	88
H(52I)	5881	14463	4845	88

Table S18. Torsion angles [°] for **Th3-TIPS**.

C(22A)-S(1A)-C(1A)-C(2A)	178.54(18)
C(22A)-S(1A)-C(1A)-C(9A)	-1.58(16)
C(9A)-C(1A)-C(2A)-C(31A)	178.5(2)
S(1A)-C(1A)-C(2A)-C(31A)	-1.6(3)
C(9A)-C(1A)-C(2A)-C(3A)	-3.1(3)
S(1A)-C(1A)-C(2A)-C(3A)	176.72(15)
C(1A)-C(2A)-C(3A)-C(8A)	-1.1(3)
C(31A)-C(2A)-C(3A)-C(8A)	177.2(2)
C(1A)-C(2A)-C(3A)-C(4A)	178.9(2)
C(31A)-C(2A)-C(3A)-C(4A)	-2.8(3)
C(8A)-C(3A)-C(4A)-C(5A)	-0.7(3)
C(2A)-C(3A)-C(4A)-C(5A)	179.3(2)
C(3A)-C(4A)-C(5A)-C(6A)	-1.8(3)
C(3A)-C(4A)-C(5A)-C(23A)	177.0(2)
C(4A)-C(5A)-C(6A)-C(7A)	2.3(4)
C(23A)-C(5A)-C(6A)-C(7A)	-176.4(2)
C(5A)-C(6A)-C(7A)-C(8A)	-0.4(4)
C(6A)-C(7A)-C(8A)-C(3A)	-2.2(4)
C(6A)-C(7A)-C(8A)-S(3A)	176.2(2)
C(4A)-C(3A)-C(8A)-C(7A)	2.7(3)
C(2A)-C(3A)-C(8A)-C(7A)	-177.3(2)
C(4A)-C(3A)-C(8A)-S(3A)	-175.51(16)
C(2A)-C(3A)-C(8A)-S(3A)	4.5(3)
C(9A)-S(3A)-C(8A)-C(7A)	178.24(18)
C(9A)-S(3A)-C(8A)-C(3A)	-3.5(2)
C(2A)-C(1A)-C(9A)-C(10A)	-177.7(2)
S(1A)-C(1A)-C(9A)-C(10A)	2.5(2)
C(2A)-C(1A)-C(9A)-S(3A)	3.6(3)
S(1A)-C(1A)-C(9A)-S(3A)	-176.25(11)
C(8A)-S(3A)-C(9A)-C(10A)	-178.88(18)

C(8A)-S(3A)-C(9A)-C(1A)	-0.3(2)
C(1A)-C(9A)-C(10A)-C(22A)	-2.3(3)
S(3A)-C(9A)-C(10A)-C(22A)	176.42(15)
C(1A)-C(9A)-C(10A)-S(4A)	-179.91(16)
S(3A)-C(9A)-C(10A)-S(4A)	-1.2(3)
C(11A)-S(4A)-C(10A)-C(9A)	177.1(2)
C(11A)-S(4A)-C(10A)-C(22A)	-0.60(16)
C(10A)-S(4A)-C(11A)-C(12A)	-177.7(2)
C(10A)-S(4A)-C(11A)-C(21A)	-0.54(15)
C(21A)-C(11A)-C(12A)-C(20A)	-2.5(2)
S(4A)-C(11A)-C(12A)-C(20A)	174.58(16)
C(21A)-C(11A)-C(12A)-S(5A)	177.21(15)
S(4A)-C(11A)-C(12A)-S(5A)	-5.7(3)
C(13A)-S(5A)-C(12A)-C(11A)	175.66(17)
C(13A)-S(5A)-C(12A)-C(20A)	-4.63(18)
C(12A)-S(5A)-C(13A)-C(14A)	-178.12(15)
C(12A)-S(5A)-C(13A)-C(18A)	3.19(19)
C(18A)-C(13A)-C(14A)-C(15A)	-0.9(3)
S(5A)-C(13A)-C(14A)-C(15A)	-179.69(16)
C(13A)-C(14A)-C(15A)-C(16A)	-0.3(3)
C(14A)-C(15A)-C(16A)-C(17A)	1.1(3)
C(14A)-C(15A)-C(16A)-C(27A)	-174.67(19)
C(15A)-C(16A)-C(17A)-C(18A)	-0.7(3)
C(27A)-C(16A)-C(17A)-C(18A)	175.12(19)
C(16A)-C(17A)-C(18A)-C(13A)	-0.5(3)
C(16A)-C(17A)-C(18A)-C(19A)	179.71(19)
C(14A)-C(13A)-C(18A)-C(17A)	1.3(3)
S(5A)-C(13A)-C(18A)-C(17A)	179.94(15)
C(14A)-C(13A)-C(18A)-C(19A)	-178.92(18)
S(5A)-C(13A)-C(18A)-C(19A)	-0.3(3)
C(17A)-C(18A)-C(19A)-C(20A)	177.82(19)
C(13A)-C(18A)-C(19A)-C(20A)	-1.9(3)
C(17A)-C(18A)-C(19A)-C(42A)	-2.8(3)
C(13A)-C(18A)-C(19A)-C(42A)	177.45(19)
C(42A)-C(19A)-C(20A)-C(12A)	-179.21(19)
C(18A)-C(19A)-C(20A)-C(12A)	0.2(3)
C(42A)-C(19A)-C(20A)-S(2A)	1.8(3)

C(18A)-C(19A)-C(20A)-S(2A)	-178.73(15)
C(11A)-C(12A)-C(20A)-C(19A)	-176.65(19)
S(5A)-C(12A)-C(20A)-C(19A)	3.6(3)
C(11A)-C(12A)-C(20A)-S(2A)	2.4(2)
S(5A)-C(12A)-C(20A)-S(2A)	-177.36(10)
C(21A)-S(2A)-C(20A)-C(19A)	177.83(17)
C(21A)-S(2A)-C(20A)-C(12A)	-1.26(15)
C(12A)-C(11A)-C(21A)-C(22A)	179.37(17)
S(4A)-C(11A)-C(21A)-C(22A)	1.6(2)
C(12A)-C(11A)-C(21A)-S(2A)	1.6(2)
S(4A)-C(11A)-C(21A)-S(2A)	-176.20(10)
C(20A)-S(2A)-C(21A)-C(22A)	-177.3(2)
C(20A)-S(2A)-C(21A)-C(11A)	-0.13(15)
C(11A)-C(21A)-C(22A)-C(10A)	-2.1(3)
S(2A)-C(21A)-C(22A)-C(10A)	175.03(17)
C(11A)-C(21A)-C(22A)-S(1A)	-178.81(17)
S(2A)-C(21A)-C(22A)-S(1A)	-1.7(4)
C(9A)-C(10A)-C(22A)-C(21A)	-176.49(18)
S(4A)-C(10A)-C(22A)-C(21A)	1.7(2)
C(9A)-C(10A)-C(22A)-S(1A)	1.0(2)
S(4A)-C(10A)-C(22A)-S(1A)	179.19(10)
C(1A)-S(1A)-C(22A)-C(21A)	177.1(2)
C(1A)-S(1A)-C(22A)-C(10A)	0.35(16)
C(6A)-C(5A)-C(23A)-C(26A)	-1.5(4)
C(4A)-C(5A)-C(23A)-C(26A)	179.8(3)
C(6A)-C(5A)-C(23A)-C(24A)	-123.9(3)
C(4A)-C(5A)-C(23A)-C(24A)	57.4(3)
C(6A)-C(5A)-C(23A)-C(25A)	117.6(3)
C(4A)-C(5A)-C(23A)-C(25A)	-61.1(3)
C(17A)-C(16A)-C(27A)-C(30A)	37.3(3)
C(15A)-C(16A)-C(27A)-C(30A)	-147.1(2)
C(17A)-C(16A)-C(27A)-C(29A)	-83.0(3)
C(15A)-C(16A)-C(27A)-C(29A)	92.6(3)
C(17A)-C(16A)-C(27A)-C(28A)	157.9(2)
C(15A)-C(16A)-C(27A)-C(28A)	-26.5(3)
C(32A)-Si(1A)-C(33A)-C(35A)	-62.5(2)
C(39A)-Si(1A)-C(33A)-C(35A)	-178.18(17)

C(36A)-Si(1A)-C(33A)-C(35A)	55.8(2)
C(32A)-Si(1A)-C(33A)-C(34A)	171.28(18)
C(39A)-Si(1A)-C(33A)-C(34A)	55.6(2)
C(36A)-Si(1A)-C(33A)-C(34A)	-70.5(2)
C(32A)-Si(1A)-C(36A)-C(37A)	-32.3(2)
C(39A)-Si(1A)-C(36A)-C(37A)	86.4(2)
C(33A)-Si(1A)-C(36A)-C(37A)	-148.3(2)
C(32A)-Si(1A)-C(36A)-C(38A)	-158.30(18)
C(39A)-Si(1A)-C(36A)-C(38A)	-39.6(2)
C(33A)-Si(1A)-C(36A)-C(38A)	85.7(2)
C(32A)-Si(1A)-C(39A)-C(40A)	-65.6(2)
C(36A)-Si(1A)-C(39A)-C(40A)	174.83(18)
C(33A)-Si(1A)-C(39A)-C(40A)	48.8(2)
C(32A)-Si(1A)-C(39A)-C(41A)	58.3(2)
C(36A)-Si(1A)-C(39A)-C(41A)	-61.3(2)
C(33A)-Si(1A)-C(39A)-C(41A)	172.72(18)
C(43A)-Si(2A)-C(44A)-C(45A)	173.0(2)
C(47A)-Si(2A)-C(44A)-C(45A)	-69.5(2)
C(50A)-Si(2A)-C(44A)-C(45A)	57.1(2)
C(43A)-Si(2A)-C(44A)-C(46A)	45.3(2)
C(47A)-Si(2A)-C(44A)-C(46A)	162.8(2)
C(50A)-Si(2A)-C(44A)-C(46A)	-70.6(2)
C(43A)-Si(2A)-C(47A)-C(48A)	81.9(3)
C(50A)-Si(2A)-C(47A)-C(48A)	-164.5(2)
C(44A)-Si(2A)-C(47A)-C(48A)	-35.4(3)
C(43A)-Si(2A)-C(47A)-C(49A)	-150.49(19)
C(50A)-Si(2A)-C(47A)-C(49A)	-36.9(2)
C(44A)-Si(2A)-C(47A)-C(49A)	92.2(2)
C(43A)-Si(2A)-C(50A)-C(52A)	48.9(2)
C(47A)-Si(2A)-C(50A)-C(52A)	-66.9(2)
C(44A)-Si(2A)-C(50A)-C(52A)	166.45(19)
C(43A)-Si(2A)-C(50A)-C(51A)	-74.6(2)
C(47A)-Si(2A)-C(50A)-C(51A)	169.73(18)
C(44A)-Si(2A)-C(50A)-C(51A)	43.0(2)

C(22B)-S(1B)-C(1B)-C(2B)	-177.96(18)

C(22B)-S(1B)-C(1B)-C(9B)	-0.09(16)
C(9B)-C(1B)-C(2B)-C(31B)	179.3(2)
S(1B)-C(1B)-C(2B)-C(31B)	-3.1(3)
C(9B)-C(1B)-C(2B)-C(3B)	-2.9(3)
S(1B)-C(1B)-C(2B)-C(3B)	174.67(15)
C(1B)-C(2B)-C(3B)-C(8B)	4.6(3)
C(31B)-C(2B)-C(3B)-C(8B)	-177.6(2)
C(1B)-C(2B)-C(3B)-C(4B)	-174.5(2)
C(31B)-C(2B)-C(3B)-C(4B)	3.3(3)
C(8B)-C(3B)-C(4B)-C(5B)	0.1(3)
C(2B)-C(3B)-C(4B)-C(5B)	179.3(2)
C(5B)-C(6B)-C(7B)-C(8B)	0.7(4)
C(6B)-C(7B)-C(8B)-C(3B)	0.3(4)
C(6B)-C(7B)-C(8B)-S(3B)	177.72(19)
C(4B)-C(3B)-C(8B)-C(7B)	-0.7(3)
C(2B)-C(3B)-C(8B)-C(7B)	-179.8(2)
C(4B)-C(3B)-C(8B)-S(3B)	-177.85(16)
C(2B)-C(3B)-C(8B)-S(3B)	3.0(3)
C(9B)-S(3B)-C(8B)-C(7B)	173.06(18)
C(9B)-S(3B)-C(8B)-C(3B)	-9.6(2)
C(2B)-C(1B)-C(9B)-C(10B)	177.1(2)
S(1B)-C(1B)-C(9B)-C(10B)	-0.6(2)
C(2B)-C(1B)-C(9B)-S(3B)	-6.4(3)
S(1B)-C(1B)-C(9B)-S(3B)	175.85(12)
C(8B)-S(3B)-C(9B)-C(10B)	-172.85(18)
C(8B)-S(3B)-C(9B)-C(1B)	11.1(2)
C(1B)-C(9B)-C(10B)-C(22B)	1.3(3)
S(3B)-C(9B)-C(10B)-C(22B)	-175.15(15)
C(1B)-C(9B)-C(10B)-S(4B)	-178.40(16)
S(3B)-C(9B)-C(10B)-S(4B)	5.2(3)
C(11B)-S(4B)-C(10B)-C(9B)	-179.4(2)
C(11B)-S(4B)-C(10B)-C(22B)	0.90(16)
C(10B)-S(4B)-C(11B)-C(12B)	180.0(2)
C(10B)-S(4B)-C(11B)-C(21B)	-0.94(15)
C(21B)-C(11B)-C(12B)-C(20B)	-0.9(2)
S(4B)-C(11B)-C(12B)-C(20B)	178.15(16)
C(21B)-C(11B)-C(12B)-S(5B)	176.52(14)

S(4B)-C(11B)-C(12B)-S(5B)	-4.4(3)
C(13B)-S(5B)-C(12B)-C(11B)	174.85(17)
C(13B)-S(5B)-C(12B)-C(20B)	-7.95(18)
C(12B)-S(5B)-C(13B)-C(14B)	-175.12(15)
C(12B)-S(5B)-C(13B)-C(18B)	6.43(19)
C(18B)-C(13B)-C(14B)-C(15B)	-2.0(3)
S(5B)-C(13B)-C(14B)-C(15B)	179.47(16)
C(13B)-C(14B)-C(15B)-C(16B)	0.7(3)
C(14B)-C(15B)-C(16B)-C(17B)	0.8(3)
C(14B)-C(15B)-C(16B)-C(27B)	-174.86(19)
C(15B)-C(16B)-C(17B)-C(18B)	-1.0(3)
C(27B)-C(16B)-C(17B)-C(18B)	174.54(19)
C(14B)-C(13B)-C(18B)-C(17B)	1.7(3)
S(5B)-C(13B)-C(18B)-C(17B)	-179.91(15)
C(14B)-C(13B)-C(18B)-C(19B)	-178.97(19)
S(5B)-C(13B)-C(18B)-C(19B)	-0.6(3)
C(16B)-C(17B)-C(18B)-C(13B)	-0.2(3)
C(16B)-C(17B)-C(18B)-C(19B)	-179.53(19)
C(13B)-C(18B)-C(19B)-C(20B)	-5.4(3)
C(17B)-C(18B)-C(19B)-C(20B)	173.92(19)
C(13B)-C(18B)-C(19B)-C(42B)	174.46(19)
C(17B)-C(18B)-C(19B)-C(42B)	-6.3(3)
C(42B)-C(19B)-C(20B)-C(12B)	-176.16(19)
C(18B)-C(19B)-C(20B)-C(12B)	3.7(3)
C(42B)-C(19B)-C(20B)-S(2B)	4.0(3)
C(18B)-C(19B)-C(20B)-S(2B)	-176.15(15)
C(11B)-C(12B)-C(20B)-C(19B)	-178.48(19)
S(5B)-C(12B)-C(20B)-C(19B)	4.0(3)
C(11B)-C(12B)-C(20B)-S(2B)	1.4(2)
S(5B)-C(12B)-C(20B)-S(2B)	-176.15(11)
C(21B)-S(2B)-C(20B)-C(19B)	178.73(18)
C(21B)-S(2B)-C(20B)-C(12B)	-1.12(15)
C(12B)-C(11B)-C(21B)-C(22B)	-179.97(17)
S(4B)-C(11B)-C(21B)-C(22B)	0.8(2)
C(12B)-C(11B)-C(21B)-S(2B)	0.1(2)
S(4B)-C(11B)-C(21B)-S(2B)	-179.20(10)
C(20B)-S(2B)-C(21B)-C(22B)	-179.3(2)

C(20B)-S(2B)-C(21B)-C(11B)	0.61(15)
C(11B)-C(21B)-C(22B)-C(10B)	-0.1(2)
S(2B)-C(21B)-C(22B)-C(10B)	179.88(17)
C(11B)-C(21B)-C(22B)-S(1B)	-178.79(17)
S(2B)-C(21B)-C(22B)-S(1B)	1.2(4)
C(9B)-C(10B)-C(22B)-C(21B)	179.63(18)
S(4B)-C(10B)-C(22B)-C(21B)	-0.6(2)
C(9B)-C(10B)-C(22B)-S(1B)	-1.3(2)
S(4B)-C(10B)-C(22B)-S(1B)	178.41(10)
C(1B)-S(1B)-C(22B)-C(21B)	179.5(2)
C(1B)-S(1B)-C(22B)-C(10B)	0.77(15)
C(3B)-C(4B)-C(5B)-C(6B)	0.9(3)
C(3B)-C(4B)-C(5B)-C(23')	169.4(4)
C(3B)-C(4B)-C(5B)-C(23B)	-173.7(3)
C(7B)-C(6B)-C(5B)-C(4B)	-1.2(4)
C(7B)-C(6B)-C(5B)-C(23')	-169.8(4)
C(7B)-C(6B)-C(5B)-C(23B)	173.2(3)
C(4B)-C(5B)-C(23B)-C(26B)	-135.9(4)
C(6B)-C(5B)-C(23B)-C(26B)	49.8(6)
C(4B)-C(5B)-C(23B)-C(24B)	-15.6(6)
C(6B)-C(5B)-C(23B)-C(24B)	170.1(4)
C(4B)-C(5B)-C(23B)-C(25B)	104.6(4)
C(6B)-C(5B)-C(23B)-C(25B)	-69.7(4)
C(4B)-C(5B)-C(23')-C(26')	176.9(5)
C(6B)-C(5B)-C(23')-C(26')	-14.9(8)
C(4B)-C(5B)-C(23')-C(24')	-60.9(7)
C(6B)-C(5B)-C(23')-C(24')	107.3(6)
C(4B)-C(5B)-C(23')-C(25')	58.1(6)
C(6B)-C(5B)-C(23')-C(25')	-133.7(5)
C(17B)-C(16B)-C(27B)-C(30B)	32.3(3)
C(15B)-C(16B)-C(27B)-C(30B)	-152.3(2)
C(17B)-C(16B)-C(27B)-C(28B)	-88.0(2)
C(15B)-C(16B)-C(27B)-C(28B)	87.4(2)
C(17B)-C(16B)-C(27B)-C(29B)	152.5(2)
C(15B)-C(16B)-C(27B)-C(29B)	-32.0(3)
C(37B)-C(36B)-Si(1B)-C(32B)	63.8(2)
C(38B)-C(36B)-Si(1B)-C(32B)	-58.8(2)

C(37B)-C(36B)-Si(1B)-C(33B)	-49.3(2)
C(38B)-C(36B)-Si(1B)-C(33B)	-171.87(18)
C(37B)-C(36B)-Si(1B)-C(39')	-176.5(4)
C(38B)-C(36B)-Si(1B)-C(39')	60.9(4)
C(37B)-C(36B)-Si(1B)-C(39B)	-179.4(2)
C(38B)-C(36B)-Si(1B)-C(39B)	58.0(2)
C(34B)-C(33B)-Si(1B)-C(32B)	-163.2(2)
C(35B)-C(33B)-Si(1B)-C(32B)	69.1(2)
C(34B)-C(33B)-Si(1B)-C(36B)	-48.9(3)
C(35B)-C(33B)-Si(1B)-C(36B)	-176.69(18)
C(34B)-C(33B)-Si(1B)-C(39')	88.6(4)
C(35B)-C(33B)-Si(1B)-C(39')	-39.2(4)
C(34B)-C(33B)-Si(1B)-C(39B)	75.7(3)
C(35B)-C(33B)-Si(1B)-C(39B)	-52.0(3)
C(32B)-Si(1B)-C(39')-C(40')	175.0(8)
C(36B)-Si(1B)-C(39')-C(40')	54.2(10)
C(33B)-Si(1B)-C(39')-C(40')	-76.2(9)
C(32B)-Si(1B)-C(39')-C(41')	46.6(8)
C(36B)-Si(1B)-C(39')-C(41')	-74.2(8)
C(33B)-Si(1B)-C(39')-C(41')	155.4(7)
C(43B)-Si(2B)-C(44')-C(45')	-0.7(14)
C(47B)-Si(2B)-C(44')-C(45')	-131.9(11)
C(50')-Si(2B)-C(44')-C(45')	108.4(13)
C(43B)-Si(2B)-C(44')-C(46')	157.4(8)
C(47B)-Si(2B)-C(44')-C(46')	26.3(11)
C(50')-Si(2B)-C(44')-C(46')	-93.4(10)
C(50B)-Si(2B)-C(47B)-C(49B)	-53.6(3)
C(44')-Si(2B)-C(47B)-C(49B)	-158.5(5)
C(43B)-Si(2B)-C(47B)-C(49B)	69.0(2)
C(44B)-Si(2B)-C(47B)-C(49B)	-179.1(2)
C(50')-Si(2B)-C(47B)-C(49B)	-42.6(4)
C(50B)-Si(2B)-C(47B)-C(48B)	-178.7(3)
C(44')-Si(2B)-C(47B)-C(48B)	76.4(5)
C(43B)-Si(2B)-C(47B)-C(48B)	-56.1(2)
C(44B)-Si(2B)-C(47B)-C(48B)	55.8(2)
C(50')-Si(2B)-C(47B)-C(48B)	-167.8(4)
C(43B)-Si(2B)-C(50B)-C(52B)	-41.8(5)

C(47B)-Si(2B)-C(50B)-C(52B)	80.6(4)
C(44B)-Si(2B)-C(50B)-C(52B)	-159.1(4)
C(43B)-Si(2B)-C(50B)-C(51B)	-167.6(5)
C(47B)-Si(2B)-C(50B)-C(51B)	-45.2(6)
C(44B)-Si(2B)-C(50B)-C(51B)	75.1(5)

Table S19. Crystal data and structure refinement for **BDTh-TIPS**.

Empirical formula	C54 H68 S4 Si2	
Formula weight	901.50	
Temperature	100(2) K	
Wavelength	1.54178 Å	
Crystal system	Monoclinic	
Space group	$P2_1/n$	
Unit cell dimensions	a = 6.9436(2) Å	a = 90°.
	b = 21.6735(6) Å	b = 94.3960(10)°.
	c = 33.9795(8) Å	g = 90°.
Volume	5098.6(2) $Å^3$	
Z	4	
Density (calculated)	1.174 Mg/m^3	
Absorption coefficient	2.410 mm^{-1}	
F(000)	1936	
Crystal size	0.291 x 0.069 x 0.023 mm^3	
Theta range for data collection	2.420 to 66.593°.	
Index ranges	-4<=h<=8, -25<=k<=25, -40<=l<=36	
Reflections collected	25307	
Independent reflections	8924 [R(int) = 0.0495]	
Completeness to theta = 66.500°	99.1 %	
Absorption correction	Semi-empirical from equivalents	
Max. and min. transmission	0.7531 and 0.6226	
Refinement method	Full-matrix least-squares on F^2	
Data / restraints / parameters	8924 / 372 / 802	
Goodness-of-fit on F^2	1.018	
Final R indices [I>2sigma(I)]	R1 = 0.0495, wR2 = 0.1206	
R indices (all data)	R1 = 0.0680, wR2 = 0.1327	
Extinction coefficient	n/a	
Largest diff. peak and hole	0.547 and -0.495 e.$Å^{-3}$	

Table S20. Atomic coordinates (x 10^4) and equivalent isotropic displacement parameters ($Å^2$x 10^3) for **BDTh-TIPS**. U(eq) is defined as one third of the trace of the orthogonalized U^{ij} tensor.

	x	y	z	U(eq)
Si(1)	7201(1)	5385(1)	7790(1)	38(1)
S(1)	4101(1)	6800(1)	9659(1)	36(1)
S(2)	5202(1)	5082(1)	9089(1)	32(1)
S(3)	4305(1)	8518(1)	4254(1)	23(1)
S(4)	4186(1)	8836(1)	5500(1)	23(1)
C(1)	3910(4)	7191(2)	9204(1)	36(1)
C(2)	3517(4)	7817(2)	9218(1)	42(1)
C(3)	3323(4)	8167(2)	8878(1)	43(1)
C(4)	3501(4)	7905(2)	8508(1)	39(1)
C(5)	3930(4)	7281(1)	8499(1)	34(1)
C(6)	4160(4)	6912(1)	8838(1)	32(1)
C(7)	4635(4)	6261(1)	8802(1)	31(1)
C(8)	4688(3)	5869(1)	9125(1)	30(1)
C(9)	4450(3)	6041(1)	9520(1)	31(1)
C(10)	4684(3)	5552(1)	9786(1)	31(1)
C(11)	4566(3)	5558(2)	10202(1)	31(1)
C(12)	4877(3)	5016(2)	10401(1)	32(1)
C(13)	3140(5)	8269(2)	8122(1)	45(1)
C(17)	5217(4)	6016(1)	8441(1)	31(1)
C(18)	5848(4)	5775(1)	8157(1)	32(1)
C(55)	3892(3)	7721(1)	4317(1)	23(1)
C(56)	3742(4)	7371(1)	3972(1)	28(1)
C(57)	3459(4)	6738(1)	3988(1)	30(1)
C(58)	3335(4)	6436(1)	4348(1)	30(1)
C(59)	3426(4)	6796(1)	4686(1)	29(1)
C(60)	3686(3)	7438(1)	4681(1)	24(1)
C(61)	3678(3)	7785(1)	5048(1)	24(1)
C(62)	4095(3)	8408(1)	5062(1)	21(1)
C(63)	4436(3)	8789(1)	4736(1)	21(1)
C(64)	4750(3)	9409(1)	4836(1)	20(1)
C(65)	5115(3)	9911(1)	4583(1)	21(1)
C(66)	5346(3)	10481(1)	4750(1)	21(1)
C(67)	3181(4)	5729(1)	4376(1)	34(1)
C(71)	3037(4)	7501(1)	5393(1)	28(1)
C(72)	2321(4)	7294(2)	5676(1)	39(1)
C(14)	1480(13)	7972(4)	7920(3)	47(2)

C(15)	2765(14)	8926(4)	8250(2)	53(2)
C(16)	5014(12)	8216(4)	7929(2)	55(2)
C(14A)	908(13)	8258(5)	7958(3)	58(2)
C(15A)	3661(15)	8969(4)	8122(3)	52(2)
C(16A)	4123(15)	8005(4)	7749(2)	63(2)
C(19)	8832(9)	4790(3)	7998(2)	44(1)
C(20)	9812(12)	4938(4)	8396(2)	46(1)
C(21)	7647(10)	4193(3)	8038(2)	49(1)
C(22)	5611(9)	5114(3)	7356(2)	41(1)
C(23)	6723(10)	4848(3)	7024(2)	46(1)
C(24)	4088(10)	5575(3)	7189(2)	46(1)
C(25)	8740(9)	6091(3)	7571(2)	47(1)
C(26)	10297(10)	6136(3)	7981(2)	44(1)
C(27)	7614(11)	6701(4)	7533(2)	56(1)
C(19A)	9727(9)	5431(3)	7969(2)	42(1)
C(20A)	10630(10)	6208(4)	7778(2)	50(1)
C(21A)	10251(11)	5167(4)	8380(2)	43(1)
C(22A)	6300(9)	4511(3)	7901(2)	45(1)
C(23A)	7318(10)	4037(3)	7663(2)	51(1)
C(24A)	4131(9)	4444(3)	7860(2)	45(1)
C(25A)	6445(10)	5597(3)	7290(2)	43(1)
C(26A)	7960(10)	5411(3)	7010(2)	47(1)
C(27A)	6068(10)	6296(3)	7258(2)	47(1)
C(68)	5163(11)	5501(3)	4551(2)	39(2)
C(69)	2836(10)	5437(3)	3953(2)	37(1)
C(70)	1565(10)	5570(3)	4618(2)	37(1)
C(68A)	5099(11)	5442(3)	4303(3)	51(2)
C(69A)	1607(13)	5475(3)	4085(2)	52(2)
C(70A)	2673(13)	5486(3)	4796(2)	52(2)
Si(2)	839(3)	6903(1)	6015(1)	28(1)
C(73)	-1175(12)	6447(4)	5738(2)	42(1)
C(74)	-1732(10)	6685(3)	5321(2)	45(1)
C(75)	-2972(9)	6384(3)	5962(2)	44(1)
C(76)	2381(10)	6361(3)	6329(2)	44(1)
C(77)	3428(10)	5888(3)	6100(2)	49(1)
C(78)	1263(10)	6047(3)	6648(2)	50(1)
C(79)	-138(10)	7560(3)	6304(2)	42(1)
C(80)	-1157(11)	8031(4)	6026(2)	46(1)
C(81)	1432(10)	7855(3)	6572(2)	50(1)
Si(2A)	796(3)	7172(1)	6116(1)	34(1)
C(73A)	-274(9)	6383(3)	6002(2)	40(1)
C(74A)	1302(10)	5883(3)	5992(2)	46(1)
C(75A)	-1538(12)	6397(4)	5606(2)	43(1)

C(76A)	2461(10)	7154(3)	6576(2)	43(1)
C(77A)	3810(10)	7707(3)	6625(2)	47(1)
C(78A)	1396(10)	7049(3)	6949(2)	50(1)
C(79A)	-1249(11)	7765(3)	6094(2)	41(1)
C(80A)	-529(10)	8427(3)	6173(2)	45(1)
C(81A)	-2882(10)	7603(3)	6349(2)	46(1)

Table S21. Bond lengths [Å] and angles [°] for **BDTh-TIPS.**

Si(1)-C(25A)	1.798(6)
Si(1)-C(19A)	1.815(6)
Si(1)-C(19)	1.824(7)
Si(1)-C(18)	1.825(3)
Si(1)-C(22)	1.868(6)
Si(1)-C(25)	2.039(7)
Si(1)-C(22A)	2.040(7)
S(1)-C(9)	1.734(3)
S(1)-C(1)	1.759(3)
S(2)-C(8)	1.748(3)
S(2)-C(12)#1	1.753(3)
S(3)-C(63)	1.735(2)
S(3)-C(55)	1.765(3)
S(4)-C(66)#2	1.750(2)
S(4)-C(62)	1.751(2)
C(1)-C(2)	1.386(5)
C(1)-C(6)	1.406(4)
C(2)-C(3)	1.381(5)
C(2)-H(2)	0.9500
C(3)-C(4)	1.394(4)
C(3)-H(3)	0.9500
C(4)-C(5)	1.386(4)
C(4)-C(13)	1.534(5)
C(5)-C(6)	1.401(4)
C(5)-H(5)	0.9500
C(6)-C(7)	1.455(4)
C(7)-C(8)	1.386(4)
C(7)-C(17)	1.424(4)
C(8)-C(9)	1.415(4)
C(9)-C(10)	1.393(4)
C(10)-C(11)	1.422(4)
C(10)-C(12)#1	1.429(4)
C(11)-C(12)	1.363(4)
C(11)-H(11)	0.9500

C(12)-C(10)#1	1.430(4)
C(12)-S(2)#1	1.753(3)
C(13)-C(14)	1.445(9)
C(13)-C(16)	1.504(9)
C(13)-C(15)	1.518(10)
C(13)-C(15A)	1.560(8)
C(13)-C(16A)	1.589(8)
C(13)-C(14A)	1.607(9)
C(17)-C(18)	1.209(4)
C(55)-C(56)	1.394(4)
C(55)-C(60)	1.401(4)
C(56)-C(57)	1.387(4)
C(56)-H(56)	0.9500
C(57)-C(58)	1.396(4)
C(57)-H(57)	0.9500
C(58)-C(59)	1.385(4)
C(58)-C(67)	1.539(4)
C(59)-C(60)	1.404(4)
C(59)-H(59)	0.9500
C(60)-C(61)	1.455(4)
C(61)-C(62)	1.381(3)
C(61)-C(71)	1.426(4)
C(62)-C(63)	1.416(3)
C(63)-C(64)	1.400(3)
C(64)-C(65)	1.420(3)
C(64)-C(66)#2	1.432(3)
C(65)-C(66)	1.365(4)
C(65)-H(65)	0.9500
C(66)-C(64)#2	1.432(3)
C(66)-S(4)#2	1.750(2)
C(67)-C(70)	1.483(7)
C(67)-C(68A)	1.507(8)
C(67)-C(69A)	1.519(7)
C(67)-C(68)	1.539(7)
C(67)-C(69)	1.570(7)
C(67)-C(70A)	1.587(8)
C(71)-C(72)	1.202(4)
C(72)-Si(2)	1.812(3)
C(72)-Si(2A)	1.917(3)
C(14)-H(14A)	0.9800
C(14)-H(14B)	0.9800
C(14)-H(14C)	0.9800
C(15)-H(15A)	0.9800

C(15)-H(15B)	0.9800
C(15)-H(15C)	0.9800
C(16)-H(16A)	0.9800
C(16)-H(16B)	0.9800
C(16)-H(16C)	0.9800
C(14A)-H(14D)	0.9800
C(14A)-H(14E)	0.9800
C(14A)-H(14F)	0.9800
C(15A)-H(15D)	0.9800
C(15A)-H(15E)	0.9800
C(15A)-H(15F)	0.9800
C(16A)-H(16D)	0.9800
C(16A)-H(16E)	0.9800
C(16A)-H(16F)	0.9800
C(19)-C(20)	1.501(10)
C(19)-C(21)	1.544(10)
C(19)-H(19)	1.0000
C(20)-H(20A)	0.9800
C(20)-H(20B)	0.9800
C(20)-H(20C)	0.9800
C(21)-H(21A)	0.9800
C(21)-H(21B)	0.9800
C(21)-H(21C)	0.9800
C(22)-C(23)	1.527(8)
C(22)-C(24)	1.532(9)
C(22)-H(22)	1.0000
C(23)-H(23A)	0.9800
C(23)-H(23B)	0.9800
C(23)-H(23C)	0.9800
C(24)-H(24A)	0.9800
C(24)-H(24B)	0.9800
C(24)-H(24C)	0.9800
C(25)-C(27)	1.536(10)
C(25)-C(26)	1.698(9)
C(25)-H(25)	1.0000
C(26)-H(26A)	0.9800
C(26)-H(26B)	0.9800
C(26)-H(26C)	0.9800
C(27)-H(27A)	0.9800
C(27)-H(27B)	0.9800
C(27)-H(27C)	0.9800
C(19A)-C(21A)	1.529(10)
C(19A)-C(20A)	1.927(10)

C(19A)-H(19A)	1.0000
C(20A)-H(20D)	0.9800
C(20A)-H(20E)	0.9800
C(20A)-H(20F)	0.9800
C(21A)-H(21D)	0.9800
C(21A)-H(21E)	0.9800
C(21A)-H(21F)	0.9800
C(22A)-C(24A)	1.509(9)
C(22A)-C(23A)	1.517(10)
C(22A)-H(22A)	1.0000
C(23A)-H(23D)	0.9800
C(23A)-H(23E)	0.9800
C(23A)-H(23F)	0.9800
C(24A)-H(24D)	0.9800
C(24A)-H(24E)	0.9800
C(24A)-H(24F)	0.9800
C(25A)-C(26A)	1.527(9)
C(25A)-C(27A)	1.540(9)
C(25A)-H(25A)	1.0000
C(26A)-H(26D)	0.9800
C(26A)-H(26E)	0.9800
C(26A)-H(26F)	0.9800
C(27A)-H(27D)	0.9800
C(27A)-H(27E)	0.9800
C(27A)-H(27F)	0.9800
C(68)-H(68A)	0.9800
C(68)-H(68B)	0.9800
C(68)-H(68C)	0.9800
C(69)-H(69A)	0.9800
C(69)-H(69B)	0.9800
C(69)-H(69C)	0.9800
C(70)-H(70A)	0.9800
C(70)-H(70B)	0.9800
C(70)-H(70C)	0.9800
C(68A)-H(68D)	0.9800
C(68A)-H(68E)	0.9800
C(68A)-H(68F)	0.9800
C(69A)-H(69D)	0.9800
C(69A)-H(69E)	0.9800
C(69A)-H(69F)	0.9800
C(70A)-H(70D)	0.9800
C(70A)-H(70E)	0.9800
C(70A)-H(70F)	0.9800

Si(2)-C(76)	1.868(7)
Si(2)-C(79)	1.886(7)
Si(2)-C(73)	1.903(8)
C(73)-C(75)	1.518(10)
C(73)-C(74)	1.528(10)
C(73)-H(73)	1.0000
C(74)-H(74A)	0.9800
C(74)-H(74B)	0.9800
C(74)-H(74C)	0.9800
C(75)-H(75A)	0.9800
C(75)-H(75B)	0.9800
C(75)-H(75C)	0.9800
C(76)-C(77)	1.505(10)
C(76)-C(78)	1.540(8)
C(76)-H(76)	1.0000
C(77)-H(77A)	0.9800
C(77)-H(77B)	0.9800
C(77)-H(77C)	0.9800
C(78)-H(78A)	0.9800
C(78)-H(78B)	0.9800
C(78)-H(78C)	0.9800
C(79)-C(81)	1.507(10)
C(79)-C(80)	1.526(10)
C(79)-H(79)	1.0000
C(80)-H(80A)	0.9800
C(80)-H(80B)	0.9800
C(80)-H(80C)	0.9800
C(81)-H(81A)	0.9800
C(81)-H(81B)	0.9800
C(81)-H(81C)	0.9800
Si(2A)-C(76A)	1.872(7)
Si(2A)-C(73A)	1.892(7)
Si(2A)-C(79A)	1.912(8)
C(73A)-C(74A)	1.542(9)
C(73A)-C(75A)	1.549(9)
C(73A)-H(73A)	1.0000
C(74A)-H(74D)	0.9800
C(74A)-H(74E)	0.9800
C(74A)-H(74F)	0.9800
C(75A)-H(75D)	0.9800
C(75A)-H(75E)	0.9800
C(75A)-H(75F)	0.9800
C(76A)-C(77A)	1.524(9)

C(76A)-C(78A)	1.531(9)
C(76A)-H(76A)	1.0000
C(77A)-H(77D)	0.9800
C(77A)-H(77E)	0.9800
C(77A)-H(77F)	0.9800
C(78A)-H(78D)	0.9800
C(78A)-H(78E)	0.9800
C(78A)-H(78F)	0.9800
C(79A)-C(81A)	1.520(9)
C(79A)-C(80A)	1.538(9)
C(79A)-H(79A)	1.0000
C(80A)-H(80D)	0.9800
C(80A)-H(80E)	0.9800
C(80A)-H(80F)	0.9800
C(81A)-H(81D)	0.9800
C(81A)-H(81E)	0.9800
C(81A)-H(81F)	0.9800

C(25A)-Si(1)-C(19A)	120.5(3)
C(25A)-Si(1)-C(18)	113.5(2)
C(19A)-Si(1)-C(18)	106.3(2)
C(19)-Si(1)-C(18)	113.5(2)
C(19)-Si(1)-C(22)	113.2(3)
C(18)-Si(1)-C(22)	112.4(2)
C(19)-Si(1)-C(25)	110.2(3)
C(18)-Si(1)-C(25)	102.3(2)
C(22)-Si(1)-C(25)	104.1(3)
C(25A)-Si(1)-C(22A)	109.9(3)
C(19A)-Si(1)-C(22A)	106.9(3)
C(18)-Si(1)-C(22A)	97.0(2)
C(9)-S(1)-C(1)	102.67(14)
C(8)-S(2)-C(12)#1	91.57(14)
C(63)-S(3)-C(55)	102.41(12)
C(66)#2-S(4)-C(62)	91.66(11)
C(2)-C(1)-C(6)	119.3(3)
C(2)-C(1)-S(1)	116.2(2)
C(6)-C(1)-S(1)	124.5(3)
C(3)-C(2)-C(1)	121.0(3)
C(3)-C(2)-H(2)	119.5
C(1)-C(2)-H(2)	119.5
C(2)-C(3)-C(4)	121.5(3)
C(2)-C(3)-H(3)	119.3
C(4)-C(3)-H(3)	119.3

C(5)-C(4)-C(3)	116.8(3)
C(5)-C(4)-C(13)	120.2(3)
C(3)-C(4)-C(13)	122.9(3)
C(4)-C(5)-C(6)	123.4(3)
C(4)-C(5)-H(5)	118.3
C(6)-C(5)-H(5)	118.3
C(5)-C(6)-C(1)	117.9(3)
C(5)-C(6)-C(7)	119.9(2)
C(1)-C(6)-C(7)	122.2(3)
C(8)-C(7)-C(17)	117.3(3)
C(8)-C(7)-C(6)	121.5(2)
C(17)-C(7)-C(6)	121.0(3)
C(7)-C(8)-C(9)	126.2(3)
C(7)-C(8)-S(2)	122.4(2)
C(9)-C(8)-S(2)	111.3(2)
C(10)-C(9)-C(8)	113.4(3)
C(10)-C(9)-S(1)	123.8(2)
C(8)-C(9)-S(1)	122.5(2)
C(9)-C(10)-C(11)	128.7(3)
C(9)-C(10)-C(12)#1	112.8(2)
C(11)-C(10)-C(12)#1	118.5(3)
C(12)-C(11)-C(10)	117.9(3)
C(12)-C(11)-H(11)	121.1
C(10)-C(11)-H(11)	121.1
C(11)-C(12)-C(10)#1	123.6(2)
C(11)-C(12)-S(2)#1	125.5(2)
C(10)#1-C(12)-S(2)#1	110.9(2)
C(14)-C(13)-C(16)	116.4(6)
C(14)-C(13)-C(15)	113.9(6)
C(16)-C(13)-C(15)	111.6(6)
C(14)-C(13)-C(4)	104.5(4)
C(16)-C(13)-C(4)	104.1(4)
C(15)-C(13)-C(4)	104.8(4)
C(4)-C(13)-C(15A)	118.4(4)
C(4)-C(13)-C(16A)	116.4(4)
C(15A)-C(13)-C(16A)	103.7(5)
C(4)-C(13)-C(14A)	112.3(4)
C(15A)-C(13)-C(14A)	103.4(6)
C(16A)-C(13)-C(14A)	100.4(6)
C(18)-C(17)-C(7)	173.6(3)
C(17)-C(18)-Si(1)	169.3(3)
C(56)-C(55)-C(60)	119.9(2)
C(56)-C(55)-S(3)	115.73(19)

C(60)-C(55)-S(3)	124.35(19)
C(57)-C(56)-C(55)	120.4(2)
C(57)-C(56)-H(56)	119.8
C(55)-C(56)-H(56)	119.8
C(56)-C(57)-C(58)	121.2(2)
C(56)-C(57)-H(57)	119.4
C(58)-C(57)-H(57)	119.4
C(59)-C(58)-C(57)	117.4(2)
C(59)-C(58)-C(67)	120.6(2)
C(57)-C(58)-C(67)	121.9(2)
C(58)-C(59)-C(60)	123.1(3)
C(58)-C(59)-H(59)	118.4
C(60)-C(59)-H(59)	118.4
C(55)-C(60)-C(59)	117.9(2)
C(55)-C(60)-C(61)	122.5(2)
C(59)-C(60)-C(61)	119.6(2)
C(62)-C(61)-C(71)	118.1(2)
C(62)-C(61)-C(60)	121.4(2)
C(71)-C(61)-C(60)	120.2(2)
C(61)-C(62)-C(63)	126.1(2)
C(61)-C(62)-S(4)	122.79(19)
C(63)-C(62)-S(4)	111.09(18)
C(64)-C(63)-C(62)	113.7(2)
C(64)-C(63)-S(3)	123.33(18)
C(62)-C(63)-S(3)	122.79(19)
C(63)-C(64)-C(65)	128.4(2)
C(63)-C(64)-C(66)#2	112.2(2)
C(65)-C(64)-C(66)#2	119.4(2)
C(66)-C(65)-C(64)	117.6(2)
C(66)-C(65)-H(65)	121.2
C(64)-C(65)-H(65)	121.2
C(65)-C(66)-C(64)#2	123.1(2)
C(65)-C(66)-S(4)#2	125.65(19)
C(64)#2-C(66)-S(4)#2	111.29(18)
C(68A)-C(67)-C(69A)	110.2(5)
C(70)-C(67)-C(68)	113.8(4)
C(70)-C(67)-C(58)	108.9(3)
C(68A)-C(67)-C(58)	109.5(3)
C(69A)-C(67)-C(58)	111.8(3)
C(68)-C(67)-C(58)	106.2(3)
C(70)-C(67)-C(69)	110.2(4)
C(68)-C(67)-C(69)	107.1(4)
C(58)-C(67)-C(69)	110.6(3)

C(68A)-C(67)-C(70A)	105.7(5)
C(69A)-C(67)-C(70A)	105.4(5)
C(58)-C(67)-C(70A)	114.0(3)
C(72)-C(71)-C(61)	173.3(3)
C(71)-C(72)-Si(2)	166.5(3)
C(71)-C(72)-Si(2A)	164.3(3)
C(13)-C(14)-H(14A)	109.5
C(13)-C(14)-H(14B)	109.5
H(14A)-C(14)-H(14B)	109.5
C(13)-C(14)-H(14C)	109.5
H(14A)-C(14)-H(14C)	109.5
H(14B)-C(14)-H(14C)	109.5
C(13)-C(15)-H(15A)	109.5
C(13)-C(15)-H(15B)	109.5
H(15A)-C(15)-H(15B)	109.5
C(13)-C(15)-H(15C)	109.5
H(15A)-C(15)-H(15C)	109.5
H(15B)-C(15)-H(15C)	109.5
C(13)-C(16)-H(16A)	109.5
C(13)-C(16)-H(16B)	109.5
H(16A)-C(16)-H(16B)	109.5
C(13)-C(16)-H(16C)	109.5
H(16A)-C(16)-H(16C)	109.5
H(16B)-C(16)-H(16C)	109.5
C(13)-C(14A)-H(14D)	109.5
C(13)-C(14A)-H(14E)	109.5
H(14D)-C(14A)-H(14E)	109.5
C(13)-C(14A)-H(14F)	109.5
H(14D)-C(14A)-H(14F)	109.5
H(14E)-C(14A)-H(14F)	109.5
C(13)-C(15A)-H(15D)	109.5
C(13)-C(15A)-H(15E)	109.5
H(15D)-C(15A)-H(15E)	109.5
C(13)-C(15A)-H(15F)	109.5
H(15D)-C(15A)-H(15F)	109.5
H(15E)-C(15A)-H(15F)	109.5
C(13)-C(16A)-H(16D)	109.5
C(13)-C(16A)-H(16E)	109.5
H(16D)-C(16A)-H(16E)	109.5
C(13)-C(16A)-H(16F)	109.5
H(16D)-C(16A)-H(16F)	109.5
H(16E)-C(16A)-H(16F)	109.5
C(20)-C(19)-C(21)	108.0(6)

C(20)-C(19)-Si(1)	115.0(5)
C(21)-C(19)-Si(1)	107.9(4)
C(20)-C(19)-H(19)	108.6
C(21)-C(19)-H(19)	108.6
Si(1)-C(19)-H(19)	108.6
C(19)-C(20)-H(20A)	109.5
C(19)-C(20)-H(20B)	109.5
H(20A)-C(20)-H(20B)	109.5
C(19)-C(20)-H(20C)	109.5
H(20A)-C(20)-H(20C)	109.5
H(20B)-C(20)-H(20C)	109.5
C(19)-C(21)-H(21A)	109.5
C(19)-C(21)-H(21B)	109.5
H(21A)-C(21)-H(21B)	109.5
C(19)-C(21)-H(21C)	109.5
H(21A)-C(21)-H(21C)	109.5
H(21B)-C(21)-H(21C)	109.5
C(23)-C(22)-C(24)	110.2(5)
C(23)-C(22)-Si(1)	113.5(5)
C(24)-C(22)-Si(1)	115.9(4)
C(23)-C(22)-H(22)	105.4
C(24)-C(22)-H(22)	105.4
Si(1)-C(22)-H(22)	105.4
C(22)-C(23)-H(23A)	109.5
C(22)-C(23)-H(23B)	109.5
H(23A)-C(23)-H(23B)	109.5
C(22)-C(23)-H(23C)	109.5
H(23A)-C(23)-H(23C)	109.5
H(23B)-C(23)-H(23C)	109.5
C(22)-C(24)-H(24A)	109.5
C(22)-C(24)-H(24B)	109.5
H(24A)-C(24)-H(24B)	109.5
C(22)-C(24)-H(24C)	109.5
H(24A)-C(24)-H(24C)	109.5
H(24B)-C(24)-H(24C)	109.5
C(27)-C(25)-C(26)	107.9(6)
C(27)-C(25)-Si(1)	113.5(5)
C(26)-C(25)-Si(1)	93.6(4)
C(27)-C(25)-H(25)	113.4
C(26)-C(25)-H(25)	113.4
Si(1)-C(25)-H(25)	113.4
C(25)-C(26)-H(26A)	109.5
C(25)-C(26)-H(26B)	109.5

H(26A)-C(26)-H(26B)	109.5
C(25)-C(26)-H(26C)	109.5
H(26A)-C(26)-H(26C)	109.5
H(26B)-C(26)-H(26C)	109.5
C(25)-C(27)-H(27A)	109.5
C(25)-C(27)-H(27B)	109.5
H(27A)-C(27)-H(27B)	109.5
C(25)-C(27)-H(27C)	109.5
H(27A)-C(27)-H(27C)	109.5
H(27B)-C(27)-H(27C)	109.5
C(21A)-C(19A)-Si(1)	116.2(5)
C(21A)-C(19A)-C(20A)	125.1(5)
Si(1)-C(19A)-C(20A)	105.4(4)
C(21A)-C(19A)-H(19A)	102.2
Si(1)-C(19A)-H(19A)	102.2
C(20A)-C(19A)-H(19A)	102.2
C(19A)-C(20A)-H(20D)	109.5
C(19A)-C(20A)-H(20E)	109.5
H(20D)-C(20A)-H(20E)	109.5
C(19A)-C(20A)-H(20F)	109.5
H(20D)-C(20A)-H(20F)	109.5
H(20E)-C(20A)-H(20F)	109.5
C(19A)-C(21A)-H(21D)	109.5
C(19A)-C(21A)-H(21E)	109.5
H(21D)-C(21A)-H(21E)	109.5
C(19A)-C(21A)-H(21F)	109.5
H(21D)-C(21A)-H(21F)	109.5
H(21E)-C(21A)-H(21F)	109.5
C(24A)-C(22A)-C(23A)	112.8(6)
C(24A)-C(22A)-Si(1)	113.0(5)
C(23A)-C(22A)-Si(1)	111.6(5)
C(24A)-C(22A)-H(22A)	106.3
C(23A)-C(22A)-H(22A)	106.3
Si(1)-C(22A)-H(22A)	106.3
C(22A)-C(23A)-H(23D)	109.5
C(22A)-C(23A)-H(23E)	109.5
H(23D)-C(23A)-H(23E)	109.5
C(22A)-C(23A)-H(23F)	109.5
H(23D)-C(23A)-H(23F)	109.5
H(23E)-C(23A)-H(23F)	109.5
C(22A)-C(24A)-H(24D)	109.5
C(22A)-C(24A)-H(24E)	109.5
H(24D)-C(24A)-H(24E)	109.5

C(22A)-C(24A)-H(24F)	109.5
H(24D)-C(24A)-H(24F)	109.5
H(24E)-C(24A)-H(24F)	109.5
C(26A)-C(25A)-C(27A)	109.7(5)
C(26A)-C(25A)-Si(1)	110.9(5)
C(27A)-C(25A)-Si(1)	110.7(4)
C(26A)-C(25A)-H(25A)	108.5
C(27A)-C(25A)-H(25A)	108.5
Si(1)-C(25A)-H(25A)	108.5
C(25A)-C(26A)-H(26D)	109.5
C(25A)-C(26A)-H(26E)	109.5
H(26D)-C(26A)-H(26E)	109.5
C(25A)-C(26A)-H(26F)	109.5
H(26D)-C(26A)-H(26F)	109.5
H(26E)-C(26A)-H(26F)	109.5
C(25A)-C(27A)-H(27D)	109.5
C(25A)-C(27A)-H(27E)	109.5
H(27D)-C(27A)-H(27E)	109.5
C(25A)-C(27A)-H(27F)	109.5
H(27D)-C(27A)-H(27F)	109.5
H(27E)-C(27A)-H(27F)	109.5
C(67)-C(68)-H(68A)	109.5
C(67)-C(68)-H(68B)	109.5
H(68A)-C(68)-H(68B)	109.5
C(67)-C(68)-H(68C)	109.5
H(68A)-C(68)-H(68C)	109.5
H(68B)-C(68)-H(68C)	109.5
C(67)-C(69)-H(69A)	109.5
C(67)-C(69)-H(69B)	109.5
H(69A)-C(69)-H(69B)	109.5
C(67)-C(69)-H(69C)	109.5
H(69A)-C(69)-H(69C)	109.5
H(69B)-C(69)-H(69C)	109.5
C(67)-C(70)-H(70A)	109.5
C(67)-C(70)-H(70B)	109.5
H(70A)-C(70)-H(70B)	109.5
C(67)-C(70)-H(70C)	109.5
H(70A)-C(70)-H(70C)	109.5
H(70B)-C(70)-H(70C)	109.5
C(67)-C(68A)-H(68D)	109.5
C(67)-C(68A)-H(68E)	109.5
H(68D)-C(68A)-H(68E)	109.5
C(67)-C(68A)-H(68F)	109.5

H(68D)-C(68A)-H(68F)	109.5
H(68E)-C(68A)-H(68F)	109.5
C(67)-C(69A)-H(69D)	109.5
C(67)-C(69A)-H(69E)	109.5
H(69D)-C(69A)-H(69E)	109.5
C(67)-C(69A)-H(69F)	109.5
H(69D)-C(69A)-H(69F)	109.5
H(69E)-C(69A)-H(69F)	109.5
C(67)-C(70A)-H(70D)	109.5
C(67)-C(70A)-H(70E)	109.5
H(70D)-C(70A)-H(70E)	109.5
C(67)-C(70A)-H(70F)	109.5
H(70D)-C(70A)-H(70F)	109.5
H(70E)-C(70A)-H(70F)	109.5
C(72)-Si(2)-C(76)	109.2(2)
C(72)-Si(2)-C(79)	102.9(2)
C(76)-Si(2)-C(79)	113.0(3)
C(72)-Si(2)-C(73)	111.1(3)
C(76)-Si(2)-C(73)	108.8(4)
C(79)-Si(2)-C(73)	111.8(4)
C(75)-C(73)-C(74)	109.7(6)
C(75)-C(73)-Si(2)	113.5(5)
C(74)-C(73)-Si(2)	114.0(5)
C(75)-C(73)-H(73)	106.4
C(74)-C(73)-H(73)	106.4
Si(2)-C(73)-H(73)	106.4
C(73)-C(74)-H(74A)	109.5
C(73)-C(74)-H(74B)	109.5
H(74A)-C(74)-H(74B)	109.5
C(73)-C(74)-H(74C)	109.5
H(74A)-C(74)-H(74C)	109.5
H(74B)-C(74)-H(74C)	109.5
C(73)-C(75)-H(75A)	109.5
C(73)-C(75)-H(75B)	109.5
H(75A)-C(75)-H(75B)	109.5
C(73)-C(75)-H(75C)	109.5
H(75A)-C(75)-H(75C)	109.5
H(75B)-C(75)-H(75C)	109.5
C(77)-C(76)-C(78)	110.6(6)
C(77)-C(76)-Si(2)	114.4(5)
C(78)-C(76)-Si(2)	112.5(5)
C(77)-C(76)-H(76)	106.3
C(78)-C(76)-H(76)	106.3

Si(2)-C(76)-H(76)	106.3
C(76)-C(77)-H(77A)	109.5
C(76)-C(77)-H(77B)	109.5
H(77A)-C(77)-H(77B)	109.5
C(76)-C(77)-H(77C)	109.5
H(77A)-C(77)-H(77C)	109.5
H(77B)-C(77)-H(77C)	109.5
C(76)-C(78)-H(78A)	109.5
C(76)-C(78)-H(78B)	109.5
H(78A)-C(78)-H(78B)	109.5
C(76)-C(78)-H(78C)	109.5
H(78A)-C(78)-H(78C)	109.5
H(78B)-C(78)-H(78C)	109.5
C(81)-C(79)-C(80)	111.7(6)
C(81)-C(79)-Si(2)	111.2(5)
C(80)-C(79)-Si(2)	110.5(5)
C(81)-C(79)-H(79)	107.7
C(80)-C(79)-H(79)	107.7
Si(2)-C(79)-H(79)	107.7
C(79)-C(80)-H(80A)	109.5
C(79)-C(80)-H(80B)	109.5
H(80A)-C(80)-H(80B)	109.5
C(79)-C(80)-H(80C)	109.5
H(80A)-C(80)-H(80C)	109.5
H(80B)-C(80)-H(80C)	109.5
C(79)-C(81)-H(81A)	109.5
C(79)-C(81)-H(81B)	109.5
H(81A)-C(81)-H(81B)	109.5
C(79)-C(81)-H(81C)	109.5
H(81A)-C(81)-H(81C)	109.5
H(81B)-C(81)-H(81C)	109.5
C(76A)-Si(2A)-C(73A)	111.1(3)
C(76A)-Si(2A)-C(79A)	117.2(3)
C(73A)-Si(2A)-C(79A)	108.7(3)
C(76A)-Si(2A)-C(72)	108.2(2)
C(73A)-Si(2A)-C(72)	101.3(2)
C(79A)-Si(2A)-C(72)	109.2(2)
C(74A)-C(73A)-C(75A)	110.5(6)
C(74A)-C(73A)-Si(2A)	111.8(4)
C(75A)-C(73A)-Si(2A)	110.4(5)
C(74A)-C(73A)-H(73A)	108.0
C(75A)-C(73A)-H(73A)	108.0
Si(2A)-C(73A)-H(73A)	108.0

C(73A)-C(74A)-H(74D)	109.5
C(73A)-C(74A)-H(74E)	109.5
H(74D)-C(74A)-H(74E)	109.5
C(73A)-C(74A)-H(74F)	109.5
H(74D)-C(74A)-H(74F)	109.5
H(74E)-C(74A)-H(74F)	109.5
C(73A)-C(75A)-H(75D)	109.5
C(73A)-C(75A)-H(75E)	109.5
H(75D)-C(75A)-H(75E)	109.5
C(73A)-C(75A)-H(75F)	109.5
H(75D)-C(75A)-H(75F)	109.5
H(75E)-C(75A)-H(75F)	109.5
C(77A)-C(76A)-C(78A)	111.0(6)
C(77A)-C(76A)-Si(2A)	114.2(5)
C(78A)-C(76A)-Si(2A)	112.7(5)
C(77A)-C(76A)-H(76A)	106.1
C(78A)-C(76A)-H(76A)	106.1
Si(2A)-C(76A)-H(76A)	106.1
C(76A)-C(77A)-H(77D)	109.5
C(76A)-C(77A)-H(77E)	109.5
H(77D)-C(77A)-H(77E)	109.5
C(76A)-C(77A)-H(77F)	109.5
H(77D)-C(77A)-H(77F)	109.5
H(77E)-C(77A)-H(77F)	109.5
C(76A)-C(78A)-H(78D)	109.5
C(76A)-C(78A)-H(78E)	109.5
H(78D)-C(78A)-H(78E)	109.5
C(76A)-C(78A)-H(78F)	109.5
H(78D)-C(78A)-H(78F)	109.5
H(78E)-C(78A)-H(78F)	109.5
C(81A)-C(79A)-C(80A)	111.2(6)
C(81A)-C(79A)-Si(2A)	114.0(5)
C(80A)-C(79A)-Si(2A)	113.0(5)
C(81A)-C(79A)-H(79A)	106.0
C(80A)-C(79A)-H(79A)	106.0
Si(2A)-C(79A)-H(79A)	106.0
C(79A)-C(80A)-H(80D)	109.5
C(79A)-C(80A)-H(80E)	109.5
H(80D)-C(80A)-H(80E)	109.5
C(79A)-C(80A)-H(80F)	109.5
H(80D)-C(80A)-H(80F)	109.5
H(80E)-C(80A)-H(80F)	109.5
C(79A)-C(81A)-H(81D)	109.5

C(79A)-C(81A)-H(81E)	109.5
H(81D)-C(81A)-H(81E)	109.5
C(79A)-C(81A)-H(81F)	109.5
H(81D)-C(81A)-H(81F)	109.5
H(81E)-C(81A)-H(81F)	109.5

Symmetry transformations used to generate equivalent atoms:
#1 -x+1,-y+1,-z+2   #2 -x+1,-y+2,-z+1

Table S22. Anisotropic displacement parameters ($Å^2$x 10^3) for **BDTh-TIPS**. The anisotropic displacement factor exponent takes the form: $-2\pi^2[h^2a^{*2}U^{11} + ... + 2 h k a^* b^* U^{12}]$

	U^{11}	U^{22}	U^{33}	U^{23}	U^{13}	U^{12}
Si(1)	34(1)	47(1)	30(1)	-12(1)	-2(1)	7(1)
S(1)	24(1)	57(1)	28(1)	-14(1)	3(1)	-8(1)
S(2)	27(1)	50(1)	20(1)	-5(1)	3(1)	-4(1)
S(3)	24(1)	22(1)	22(1)	2(1)	1(1)	1(1)
S(4)	26(1)	20(1)	22(1)	3(1)	3(1)	-1(1)
C(1)	20(1)	52(2)	35(2)	-11(1)	3(1)	-9(1)
C(2)	24(1)	57(2)	46(2)	-21(2)	6(1)	-7(1)
C(3)	31(2)	40(2)	57(2)	-13(2)	2(1)	-4(1)
C(4)	23(1)	44(2)	48(2)	-6(1)	2(1)	-8(1)
C(5)	26(1)	42(2)	35(2)	-7(1)	4(1)	-10(1)
C(6)	20(1)	44(2)	31(1)	-8(1)	3(1)	-9(1)
C(7)	21(1)	49(2)	25(1)	-6(1)	3(1)	-8(1)
C(8)	18(1)	49(2)	25(1)	-6(1)	4(1)	-6(1)
C(9)	16(1)	51(2)	24(1)	-6(1)	2(1)	-8(1)
C(10)	12(1)	56(2)	25(1)	-9(1)	2(1)	-9(1)
C(11)	18(1)	52(2)	23(1)	-6(1)	2(1)	-6(1)
C(12)	15(1)	59(2)	21(1)	-5(1)	1(1)	-8(1)
C(13)	45(2)	34(2)	58(2)	-3(2)	7(2)	-7(1)
C(17)	29(1)	37(2)	26(1)	1(1)	2(1)	-7(1)
C(18)	34(1)	37(2)	24(1)	1(1)	1(1)	-4(1)
C(55)	19(1)	21(1)	29(1)	0(1)	2(1)	3(1)
C(56)	25(1)	30(1)	28(1)	0(1)	2(1)	3(1)
C(57)	28(1)	27(1)	34(1)	-7(1)	2(1)	1(1)
C(58)	26(1)	23(1)	40(2)	-3(1)	-1(1)	2(1)
C(59)	29(1)	24(1)	33(1)	3(1)	-3(1)	1(1)
C(60)	20(1)	22(1)	30(1)	1(1)	-1(1)	2(1)
C(61)	22(1)	22(1)	26(1)	4(1)	1(1)	2(1)

C(62)	18(1)	23(1)	23(1)	0(1)	0(1)	1(1)
C(63)	16(1)	23(1)	23(1)	1(1)	2(1)	1(1)
C(64)	13(1)	22(1)	25(1)	2(1)	2(1)	2(1)
C(65)	17(1)	24(1)	21(1)	3(1)	0(1)	1(1)
C(66)	16(1)	23(1)	24(1)	5(1)	2(1)	2(1)
C(67)	42(2)	24(1)	36(2)	-3(1)	-1(1)	0(1)
C(71)	30(1)	22(1)	31(1)	2(1)	-2(1)	-2(1)
C(72)	40(2)	39(2)	38(2)	13(1)	2(1)	-8(1)
C(14)	56(5)	41(5)	41(4)	13(4)	-24(4)	2(4)
C(15)	73(6)	45(4)	39(5)	13(4)	-11(4)	1(4)
C(16)	68(5)	60(5)	37(4)	22(4)	3(4)	-1(4)
C(14A)	46(5)	73(7)	50(5)	32(5)	-16(4)	-5(5)
C(15A)	81(7)	33(4)	42(5)	3(3)	-5(4)	-16(4)
C(16A)	101(7)	43(5)	47(5)	19(4)	31(5)	14(5)
C(19)	39(2)	48(2)	45(2)	2(2)	3(1)	1(1)
C(20)	40(2)	53(2)	45(2)	5(2)	-2(2)	2(2)
C(21)	46(2)	48(2)	54(2)	5(2)	3(2)	3(2)
C(22)	47(2)	46(2)	30(2)	-5(2)	0(2)	-5(2)
C(23)	53(2)	51(2)	34(2)	-7(2)	2(2)	-5(2)
C(24)	52(2)	50(2)	34(2)	-4(2)	-5(2)	-4(2)
C(25)	44(2)	51(2)	46(2)	2(2)	6(2)	-11(2)
C(26)	41(2)	50(2)	41(2)	5(2)	5(2)	-12(2)
C(27)	54(2)	57(2)	57(2)	9(2)	0(2)	-7(2)
C(19A)	37(2)	48(2)	42(2)	2(2)	2(1)	-4(1)
C(20A)	45(2)	54(2)	51(2)	1(2)	1(2)	-10(2)
C(21A)	37(2)	47(2)	43(2)	4(2)	-3(2)	-3(2)
C(22A)	41(2)	46(2)	50(2)	0(2)	5(2)	1(2)
C(23A)	46(2)	48(2)	59(2)	-2(2)	7(2)	1(2)
C(24A)	42(2)	44(3)	49(3)	-5(2)	6(2)	-6(2)
C(25A)	49(2)	48(2)	32(2)	-3(2)	0(2)	-4(2)
C(26A)	56(2)	53(2)	32(2)	-4(2)	3(2)	-3(2)
C(27A)	49(2)	50(2)	40(2)	6(2)	1(2)	0(2)
C(68)	62(4)	22(3)	34(3)	-1(3)	-1(3)	9(3)
C(69)	49(4)	25(3)	40(3)	-8(2)	8(3)	0(3)
C(70)	54(4)	17(3)	40(3)	-3(2)	3(3)	-7(3)
C(68A)	50(4)	27(4)	75(6)	5(4)	7(4)	5(3)
C(69A)	76(5)	18(3)	59(5)	3(3)	-21(4)	-4(3)
C(70A)	76(5)	29(4)	53(4)	3(3)	16(4)	-2(4)
Si(2)	29(1)	29(1)	26(1)	10(1)	6(1)	2(1)
C(73)	44(2)	36(2)	47(2)	3(2)	3(2)	-5(2)
C(74)	45(2)	40(2)	49(2)	2(2)	-2(2)	-4(2)
C(75)	43(2)	39(2)	51(2)	4(2)	3(2)	-5(2)
C(76)	47(2)	40(2)	45(2)	10(2)	6(2)	1(2)

C(77)	49(2)	41(2)	55(2)	11(2)	5(2)	5(2)
C(78)	51(3)	51(3)	48(3)	21(2)	5(2)	4(2)
C(79)	48(1)	35(1)	43(1)	-2(1)	10(1)	-1(1)
C(80)	52(2)	38(2)	49(2)	-2(2)	10(2)	2(2)
C(81)	56(2)	46(2)	49(2)	-7(2)	5(2)	-1(2)
Si(2A)	45(1)	26(1)	33(1)	0(1)	10(1)	-7(1)
C(73A)	44(1)	33(1)	43(2)	2(1)	5(1)	-7(1)
C(74A)	49(2)	38(2)	51(2)	6(2)	2(2)	-2(2)
C(75A)	45(2)	37(2)	48(2)	2(2)	3(2)	-5(2)
C(76A)	50(1)	39(1)	40(1)	1(1)	6(1)	-3(1)
C(77A)	53(2)	43(2)	46(2)	-5(2)	2(2)	-5(2)
C(78A)	59(3)	51(3)	41(2)	1(2)	6(2)	-6(2)
C(79A)	49(1)	34(2)	42(1)	1(1)	11(1)	-4(1)
C(80A)	50(2)	37(2)	48(2)	-1(2)	9(2)	0(2)
C(81A)	50(2)	39(2)	50(2)	0(2)	12(2)	0(2)

Table S23. Hydrogen coordinates (x 10^4) and isotropic displacement parameters ($Å^2$x 10^3) for **BDTh-TIPS**.

	x	y	z	U(eq)
H(2)	3380	8008	9466	51
H(3)	3062	8596	8897	51
H(5)	4076	7094	8250	41
H(11)	4280	5926	10337	37
H(56)	3835	7567	3724	33
H(57)	3347	6506	3750	36
H(59)	3307	6598	4933	35
H(65)	5195	9852	4308	25
H(14A)	1086	8202	7679	71
H(14B)	414	7966	8093	71
H(14C)	1816	7549	7852	71
H(15A)	2422	9181	8017	80
H(15B)	3931	9092	8393	80
H(15C)	1699	8929	8423	80
H(16A)	5257	7783	7868	82
H(16B)	6070	8374	8109	82
H(16C)	4934	8458	7685	82
H(14D)	751	8485	7708	86
H(14E)	121	8451	8151	86
H(14F)	490	7829	7914	86
H(15D)	3304	9144	7861	79
H(15E)	5053	9019	8186	79

H(15F)	2953	9183	8320	79
H(16D)	3587	7597	7682	94
H(16E)	5520	7970	7812	94
H(16F)	3868	8285	7525	94
H(19)	9845	4711	7810	53
H(20A)	8835	5025	8582	70
H(20B)	10642	5300	8374	70
H(20C)	10598	4585	8491	70
H(21A)	8485	3869	8157	74
H(21B)	7118	4059	7776	74
H(21C)	6586	4272	8205	74
H(22)	4864	4759	7456	49
H(23A)	5820	4644	6830	69
H(23B)	7672	4547	7134	69
H(23C)	7391	5182	6896	69
H(24A)	4701	5882	7028	68
H(24B)	3504	5783	7407	68
H(24C)	3085	5355	7026	68
H(25)	9381	5978	7327	56
H(26A)	11562	5977	7920	66
H(26B)	9801	5890	8192	66
H(26C)	10425	6567	8065	66
H(27A)	7467	6871	7796	85
H(27B)	6336	6626	7399	85
H(27C)	8321	6996	7379	85
H(19A)	10341	5128	7795	51
H(20D)	11607	6380	7971	75
H(20E)	9543	6496	7740	75
H(20F)	11199	6144	7526	75
H(21D)	11629	5232	8452	64
H(21E)	9968	4724	8381	64
H(21F)	9491	5376	8572	64
H(22A)	6709	4427	8184	55
H(23D)	6955	3621	7744	76
H(23E)	8719	4089	7709	76
H(23F)	6937	4094	7381	76
H(24D)	3643	4562	7592	68
H(24E)	3559	4713	8051	68
H(24F)	3782	4015	7909	68
H(25A)	5215	5375	7210	52
H(26D)	7507	5523	6739	71
H(26E)	8171	4964	7025	71
H(26F)	9175	5626	7084	71

H(27D)	7287	6519	7312	70
H(27E)	5159	6418	7451	70
H(27F)	5519	6395	6991	70
H(68A)	5472	5697	4808	59
H(68B)	6153	5609	4372	59
H(68C)	5127	5052	4584	59
H(69A)	2718	4989	3977	56
H(69B)	3928	5536	3798	56
H(69C)	1645	5605	3821	56
H(70A)	1493	5121	4647	56
H(70B)	349	5724	4489	56
H(70C)	1785	5761	4879	56
H(68D)	6104	5611	4491	76
H(68E)	5417	5536	4033	76
H(68F)	5024	4994	4337	76
H(69D)	1610	5023	4098	79
H(69E)	1846	5606	3817	79
H(69F)	349	5631	4152	79
H(70D)	1448	5667	4863	78
H(70E)	3702	5604	4995	78
H(70F)	2554	5035	4788	78
H(73)	-666	6019	5707	51
H(74A)	-2690	6408	5189	68
H(74B)	-581	6700	5172	68
H(74C)	-2282	7100	5337	68
H(75A)	-3615	6786	5973	67
H(75B)	-2606	6241	6232	67
H(75C)	-3854	6085	5828	67
H(76)	3401	6619	6472	53
H(77A)	4222	5628	6285	73
H(77B)	4257	6098	5922	73
H(77C)	2486	5631	5947	73
H(78A)	257	5780	6522	75
H(78B)	665	6363	6805	75
H(78C)	2156	5799	6820	75
H(79)	-1120	7386	6474	50
H(80A)	-1821	8335	6181	69
H(80B)	-2101	7819	5844	69
H(80C)	-202	8240	5875	69
H(81A)	2349	8065	6412	75
H(81B)	2109	7536	6734	75
H(81C)	858	8155	6744	75
H(73A)	-1124	6274	6216	48

H(74D)	694	5480	5939	69
H(74E)	2061	5871	6247	69
H(74F)	2152	5980	5783	69
H(75D)	-732	6501	5392	65
H(75E)	-2556	6707	5621	65
H(75F)	-2127	5990	5557	65
H(76A)	3311	6785	6552	51
H(77D)	3054	8080	6670	71
H(77E)	4497	7759	6386	71
H(77F)	4746	7640	6852	71
H(78D)	2338	7003	7177	75
H(78E)	611	6674	6918	75
H(78F)	558	7403	6990	75
H(79A)	-1827	7761	5815	50
H(80D)	-1614	8715	6132	67
H(80E)	456	8529	5992	67
H(80F)	30	8460	6446	67
H(81D)	-2378	7579	6626	69
H(81E)	-3439	7204	6266	69
H(81F)	-3882	7923	6320	69

Table S24. Torsion angles [°] for **BDTh-TIPS**.

C(9)-S(1)-C(1)-C(2)	176.8(2)
C(9)-S(1)-C(1)-C(6)	-4.4(3)
C(6)-C(1)-C(2)-C(3)	1.6(4)
S(1)-C(1)-C(2)-C(3)	-179.5(2)
C(1)-C(2)-C(3)-C(4)	0.4(4)
C(2)-C(3)-C(4)-C(5)	-1.6(4)
C(2)-C(3)-C(4)-C(13)	175.1(3)
C(3)-C(4)-C(5)-C(6)	0.9(4)
C(13)-C(4)-C(5)-C(6)	-175.9(3)
C(4)-C(5)-C(6)-C(1)	1.0(4)
C(4)-C(5)-C(6)-C(7)	-179.2(2)
C(2)-C(1)-C(6)-C(5)	-2.2(4)
S(1)-C(1)-C(6)-C(5)	178.9(2)
C(2)-C(1)-C(6)-C(7)	178.0(2)
S(1)-C(1)-C(6)-C(7)	-0.8(4)
C(5)-C(6)-C(7)-C(8)	-173.6(2)
C(1)-C(6)-C(7)-C(8)	6.1(4)
C(5)-C(6)-C(7)-C(17)	11.6(4)
C(1)-C(6)-C(7)-C(17)	-168.7(2)
C(17)-C(7)-C(8)-C(9)	170.0(2)

C(6)-C(7)-C(8)-C(9)	-5.0(4)
C(17)-C(7)-C(8)-S(2)	-6.4(3)
C(6)-C(7)-C(8)-S(2)	178.57(19)
C(12)#1-S(2)-C(8)-C(7)	176.4(2)
C(12)#1-S(2)-C(8)-C(9)	-0.51(19)
C(7)-C(8)-C(9)-C(10)	-176.6(2)
S(2)-C(8)-C(9)-C(10)	0.2(3)
C(7)-C(8)-C(9)-S(1)	-1.6(4)
S(2)-C(8)-C(9)-S(1)	175.21(14)
C(1)-S(1)-C(9)-C(10)	179.9(2)
C(1)-S(1)-C(9)-C(8)	5.5(2)
C(8)-C(9)-C(10)-C(11)	179.4(2)
S(1)-C(9)-C(10)-C(11)	4.5(4)
C(8)-C(9)-C(10)-C(12)#1	0.2(3)
S(1)-C(9)-C(10)-C(12)#1	-174.65(18)
C(9)-C(10)-C(11)-C(12)	-179.2(2)
C(12)#1-C(10)-C(11)-C(12)	-0.1(4)
C(10)-C(11)-C(12)-C(10)#1	0.1(4)
C(10)-C(11)-C(12)-S(2)#1	-179.83(18)
C(5)-C(4)-C(13)-C(14)	61.1(5)
C(3)-C(4)-C(13)-C(14)	-115.5(5)
C(5)-C(4)-C(13)-C(16)	-61.5(5)
C(3)-C(4)-C(13)-C(16)	121.9(5)
C(5)-C(4)-C(13)-C(15)	-178.9(5)
C(3)-C(4)-C(13)-C(15)	4.5(5)
C(5)-C(4)-C(13)-C(15A)	-150.2(5)
C(3)-C(4)-C(13)-C(15A)	33.2(6)
C(5)-C(4)-C(13)-C(16A)	-25.5(6)
C(3)-C(4)-C(13)-C(16A)	157.9(5)
C(5)-C(4)-C(13)-C(14A)	89.3(5)
C(3)-C(4)-C(13)-C(14A)	-87.3(6)
C(25A)-Si(1)-C(18)-C(17)	-155.4(14)
C(19A)-Si(1)-C(18)-C(17)	-20.6(14)
C(19)-Si(1)-C(18)-C(17)	31.9(14)
C(22)-Si(1)-C(18)-C(17)	162.1(14)
C(25)-Si(1)-C(18)-C(17)	-86.8(14)
C(22A)-Si(1)-C(18)-C(17)	89.3(14)
C(63)-S(3)-C(55)-C(56)	-179.22(19)
C(63)-S(3)-C(55)-C(60)	1.8(2)
C(60)-C(55)-C(56)-C(57)	-2.3(4)
S(3)-C(55)-C(56)-C(57)	178.6(2)
C(55)-C(56)-C(57)-C(58)	-0.6(4)
C(56)-C(57)-C(58)-C(59)	2.6(4)

C(56)-C(57)-C(58)-C(67)	-175.1(2)
C(57)-C(58)-C(59)-C(60)	-1.8(4)
C(67)-C(58)-C(59)-C(60)	176.0(2)
C(56)-C(55)-C(60)-C(59)	3.1(3)
S(3)-C(55)-C(60)-C(59)	-177.94(19)
C(56)-C(55)-C(60)-C(61)	-175.1(2)
S(3)-C(55)-C(60)-C(61)	3.8(3)
C(58)-C(59)-C(60)-C(55)	-1.1(4)
C(58)-C(59)-C(60)-C(61)	177.2(2)
C(55)-C(60)-C(61)-C(62)	-7.5(4)
C(59)-C(60)-C(61)-C(62)	174.3(2)
C(55)-C(60)-C(61)-C(71)	165.8(2)
C(59)-C(60)-C(61)-C(71)	-12.4(4)
C(71)-C(61)-C(62)-C(63)	-168.9(2)
C(60)-C(61)-C(62)-C(63)	4.6(4)
C(71)-C(61)-C(62)-S(4)	8.9(3)
C(60)-C(61)-C(62)-S(4)	-177.63(18)
C(66)#2-S(4)-C(62)-C(61)	-177.6(2)
C(66)#2-S(4)-C(62)-C(63)	0.50(18)
C(61)-C(62)-C(63)-C(64)	177.6(2)
S(4)-C(62)-C(63)-C(64)	-0.4(3)
C(61)-C(62)-C(63)-S(3)	1.9(3)
S(4)-C(62)-C(63)-S(3)	-176.07(13)
C(55)-S(3)-C(63)-C(64)	-179.8(2)
C(55)-S(3)-C(63)-C(62)	-4.5(2)
C(62)-C(63)-C(64)-C(65)	-179.4(2)
S(3)-C(63)-C(64)-C(65)	-3.8(4)
C(62)-C(63)-C(64)-C(66)#2	0.0(3)
S(3)-C(63)-C(64)-C(66)#2	175.67(17)
C(63)-C(64)-C(65)-C(66)	179.0(2)
C(66)#2-C(64)-C(65)-C(66)	-0.4(4)
C(64)-C(65)-C(66)-C(64)#2	0.4(4)
C(64)-C(65)-C(66)-S(4)#2	179.76(17)
C(59)-C(58)-C(67)-C(70)	51.5(4)
C(57)-C(58)-C(67)-C(70)	-130.8(4)
C(59)-C(58)-C(67)-C(68A)	-105.2(5)
C(57)-C(58)-C(67)-C(68A)	72.4(5)
C(59)-C(58)-C(67)-C(69A)	132.4(5)
C(57)-C(58)-C(67)-C(69A)	-50.0(5)
C(59)-C(58)-C(67)-C(68)	-71.3(4)
C(57)-C(58)-C(67)-C(68)	106.3(4)
C(59)-C(58)-C(67)-C(69)	172.8(4)
C(57)-C(58)-C(67)-C(69)	-9.6(4)

C(59)-C(58)-C(67)-C(70A)	13.0(5)
C(57)-C(58)-C(67)-C(70A)	-169.4(4)
C(18)-Si(1)-C(19)-C(20)	-37.2(6)
C(22)-Si(1)-C(19)-C(20)	-166.9(5)
C(25)-Si(1)-C(19)-C(20)	76.9(6)
C(18)-Si(1)-C(19)-C(21)	83.4(5)
C(22)-Si(1)-C(19)-C(21)	-46.4(5)
C(25)-Si(1)-C(19)-C(21)	-162.5(4)
C(19)-Si(1)-C(22)-C(23)	-55.9(6)
C(18)-Si(1)-C(22)-C(23)	173.8(4)
C(25)-Si(1)-C(22)-C(23)	63.8(5)
C(19)-Si(1)-C(22)-C(24)	175.0(5)
C(18)-Si(1)-C(22)-C(24)	44.7(5)
C(25)-Si(1)-C(22)-C(24)	-65.3(5)
C(25A)-Si(1)-C(19A)-C(21A)	-172.8(5)
C(18)-Si(1)-C(19A)-C(21A)	56.3(6)
C(22A)-Si(1)-C(19A)-C(21A)	-46.5(6)
C(25A)-Si(1)-C(19A)-C(20A)	44.1(5)
C(18)-Si(1)-C(19A)-C(20A)	-86.8(4)
C(22A)-Si(1)-C(19A)-C(20A)	170.4(4)
C(19A)-Si(1)-C(25A)-C(26A)	35.9(6)
C(18)-Si(1)-C(25A)-C(26A)	163.6(4)
C(22A)-Si(1)-C(25A)-C(26A)	-89.0(5)
C(19A)-Si(1)-C(25A)-C(27A)	-86.1(5)
C(18)-Si(1)-C(25A)-C(27A)	41.6(5)
C(22A)-Si(1)-C(25A)-C(27A)	149.0(4)
C(71)-C(72)-Si(2)-C(76)	122.3(13)
C(71)-C(72)-Si(2)-C(79)	-117.4(13)
C(71)-C(72)-Si(2)-C(73)	2.3(13)
C(72)-Si(2)-C(76)-C(77)	-57.3(6)
C(79)-Si(2)-C(76)-C(77)	-171.1(5)
C(73)-Si(2)-C(76)-C(77)	64.1(6)
C(72)-Si(2)-C(76)-C(78)	175.4(5)
C(79)-Si(2)-C(76)-C(78)	61.6(6)
C(73)-Si(2)-C(76)-C(78)	-63.2(6)
C(72)-Si(2)-C(79)-C(81)	-69.7(5)
C(76)-Si(2)-C(79)-C(81)	47.9(6)
C(73)-Si(2)-C(79)-C(81)	171.0(5)
C(72)-Si(2)-C(79)-C(80)	54.9(5)
C(76)-Si(2)-C(79)-C(80)	172.5(5)
C(73)-Si(2)-C(79)-C(80)	-64.3(6)
C(76A)-Si(2A)-C(73A)-C(74A)	-52.8(5)
C(79A)-Si(2A)-C(73A)-C(74A)	176.8(4)

C(72)-Si(2A)-C(73A)-C(74A)	61.9(5)
C(76A)-Si(2A)-C(73A)-C(75A)	-176.2(5)
C(79A)-Si(2A)-C(73A)-C(75A)	53.4(6)
C(72)-Si(2A)-C(73A)-C(75A)	-61.5(5)
C(73A)-Si(2A)-C(76A)-C(77A)	163.3(5)
C(79A)-Si(2A)-C(76A)-C(77A)	-71.0(6)
C(72)-Si(2A)-C(76A)-C(77A)	52.9(5)
C(73A)-Si(2A)-C(76A)-C(78A)	-68.8(6)
C(79A)-Si(2A)-C(76A)-C(78A)	56.9(6)
C(72)-Si(2A)-C(76A)-C(78A)	-179.2(4)

Symmetry transformations used to generate equivalent atoms:
#1 -x+1,-y+1,-z+2 #2 -x+1,-y+2,-z+1

7. ^{1}H and ^{13}C NMR spectra of all new compounds

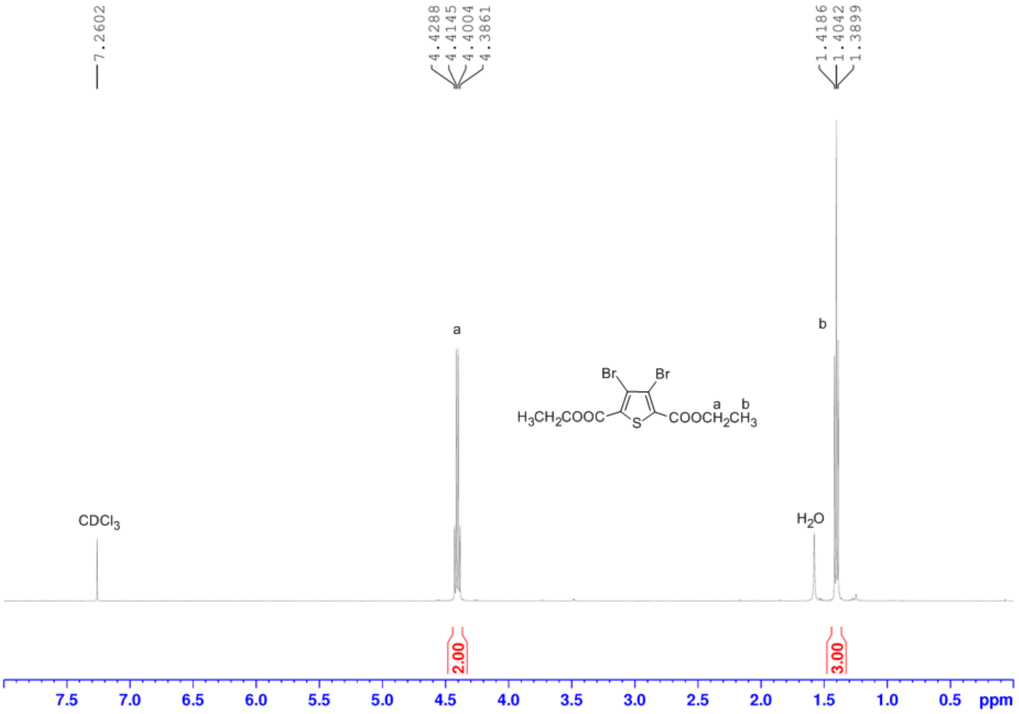

Fig. S4. ^{1}H NMR spectrum of compound **4** (500 MHz, $CDCl_3$, rt).

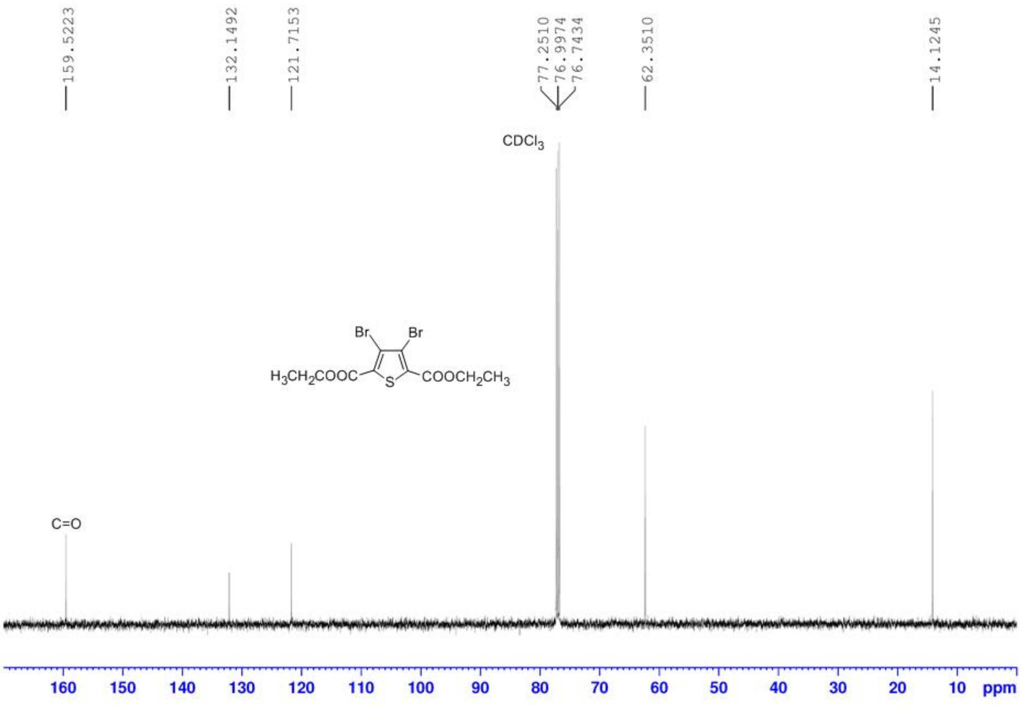

Fig. S5. ^{13}C NMR spectrum of compound **4** (125 MHz, $CDCl_3$, rt).

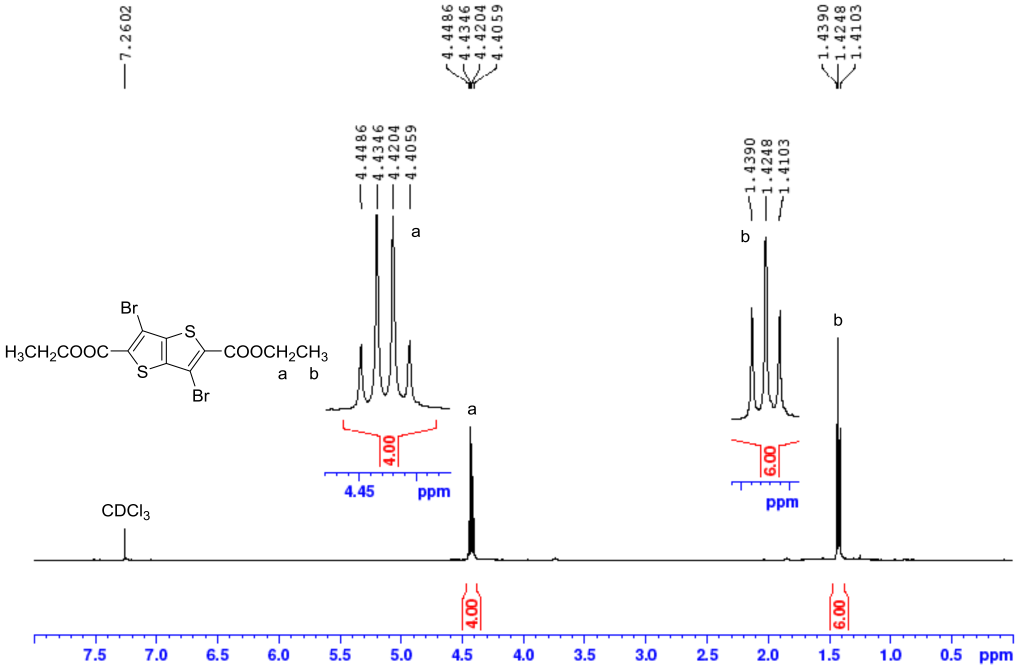

Fig. S6. ^{1}H NMR spectrum of compound **5** (500 MHz, $CDCl_3$, rt).

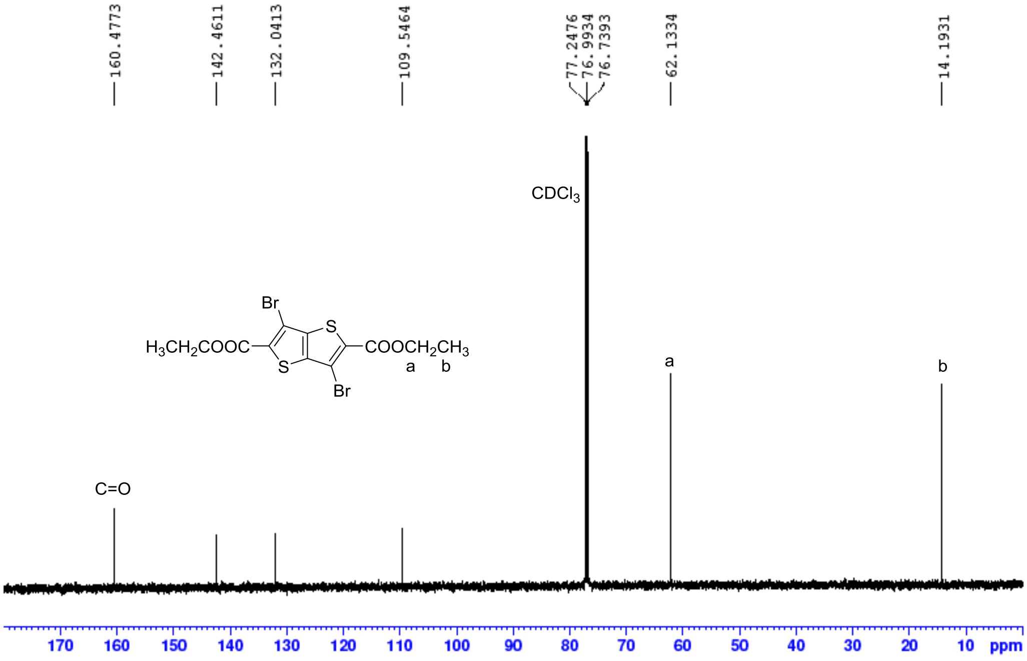

Fig. S7. ^{13}C NMR spectrum of compound **5** (125 MHz, $CDCl_3$, rt).

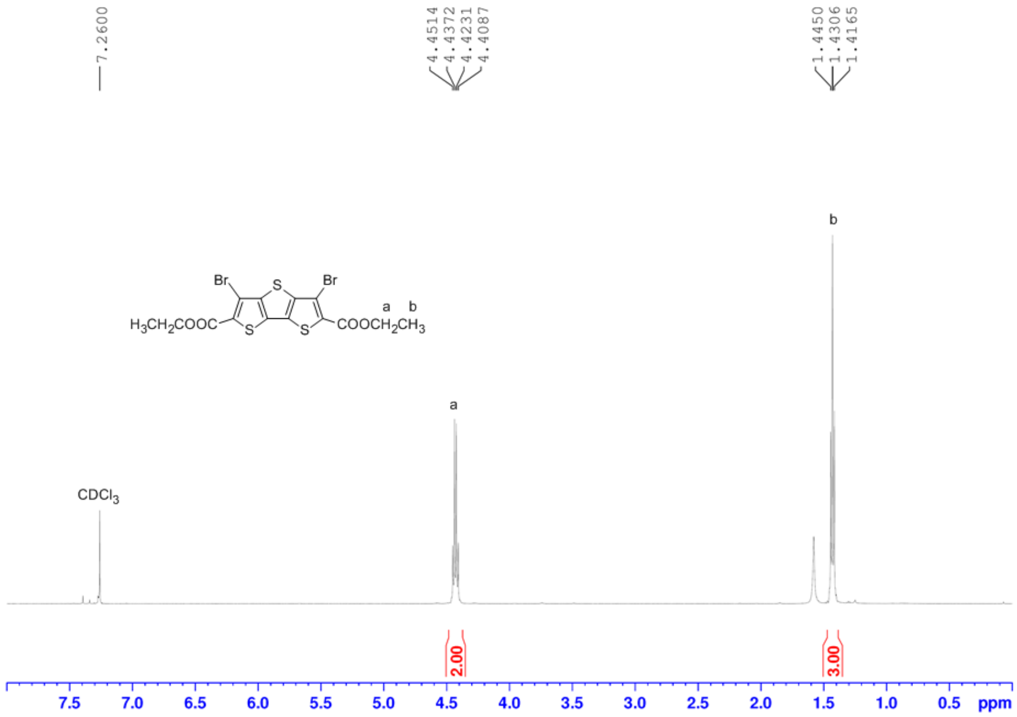

Fig. S8. ^{1}H NMR spectrum of compound **6** (500 MHz, $CDCl_3$, rt).

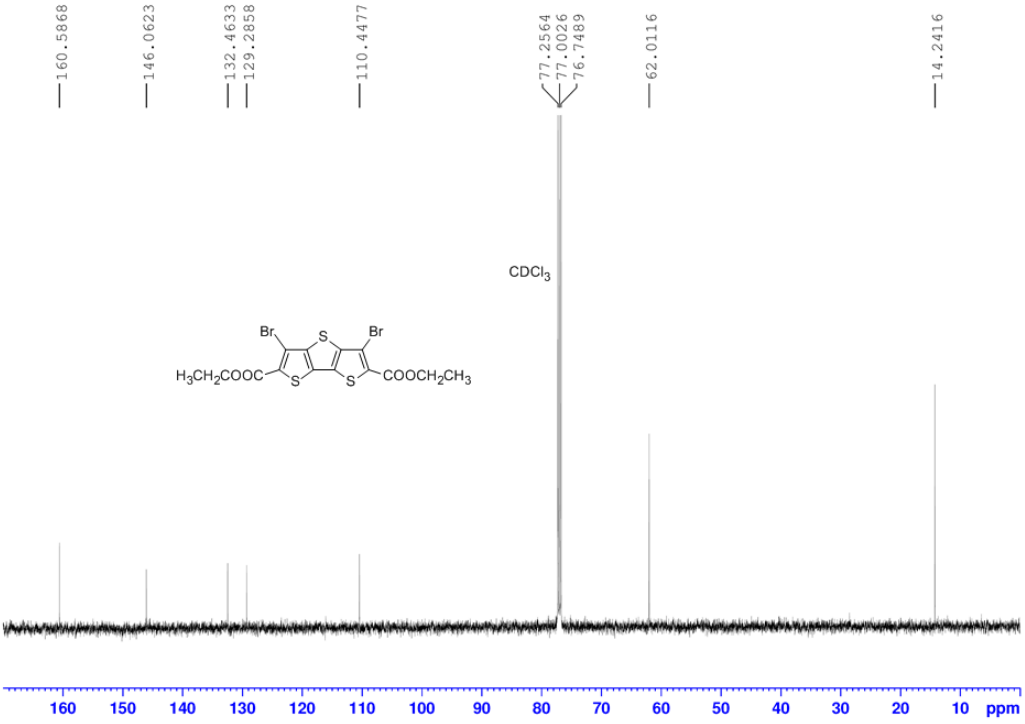

Fig. S9. ^{13}C NMR spectrum of compound **6** (125 MHz, $CDCl_3$, rt).

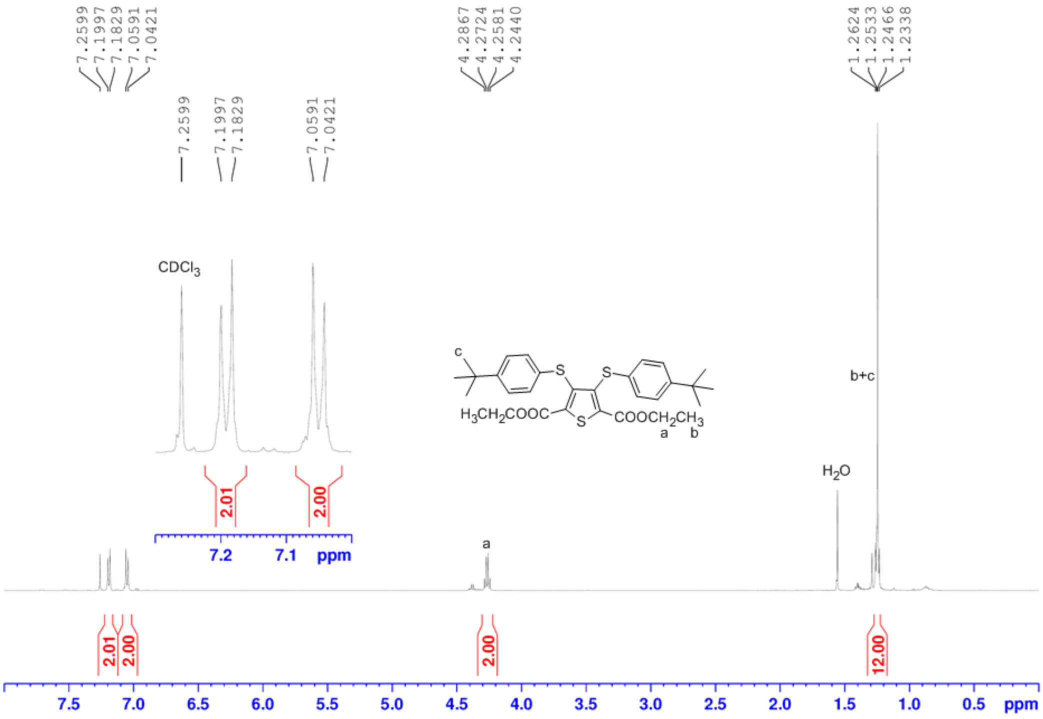

Fig. S10. ^{1}H NMR spectrum of compound **7** (500 MHz, $CDCl_3$, rt).

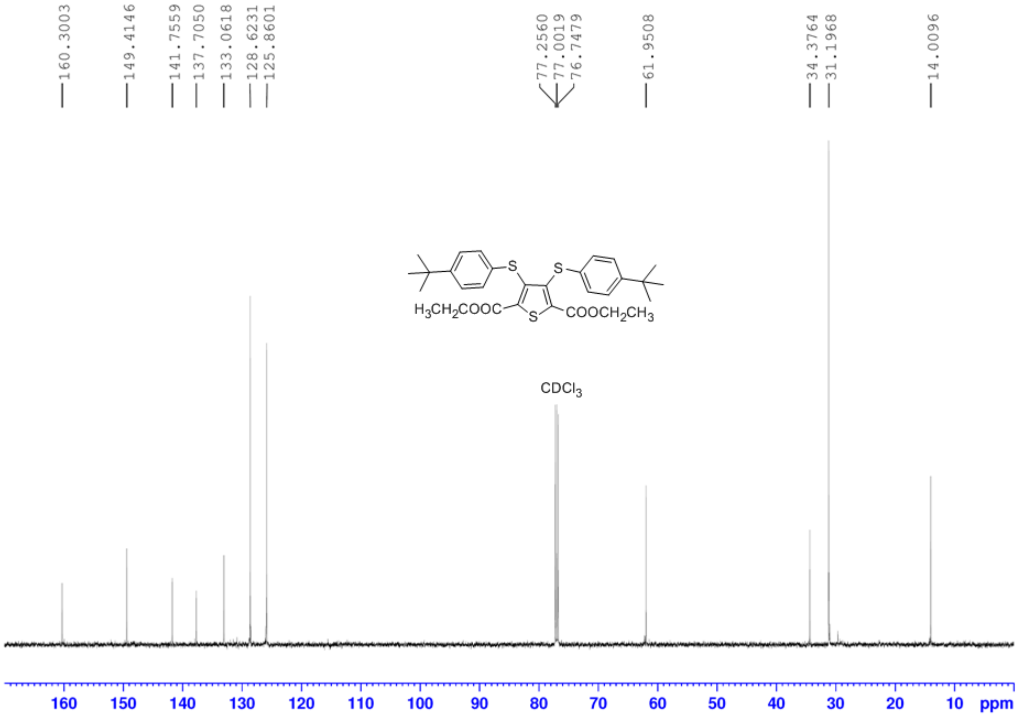

Fig. S11. ^{13}C NMR spectrum of compound **7** (125 MHz, $CDCl_3$, rt).

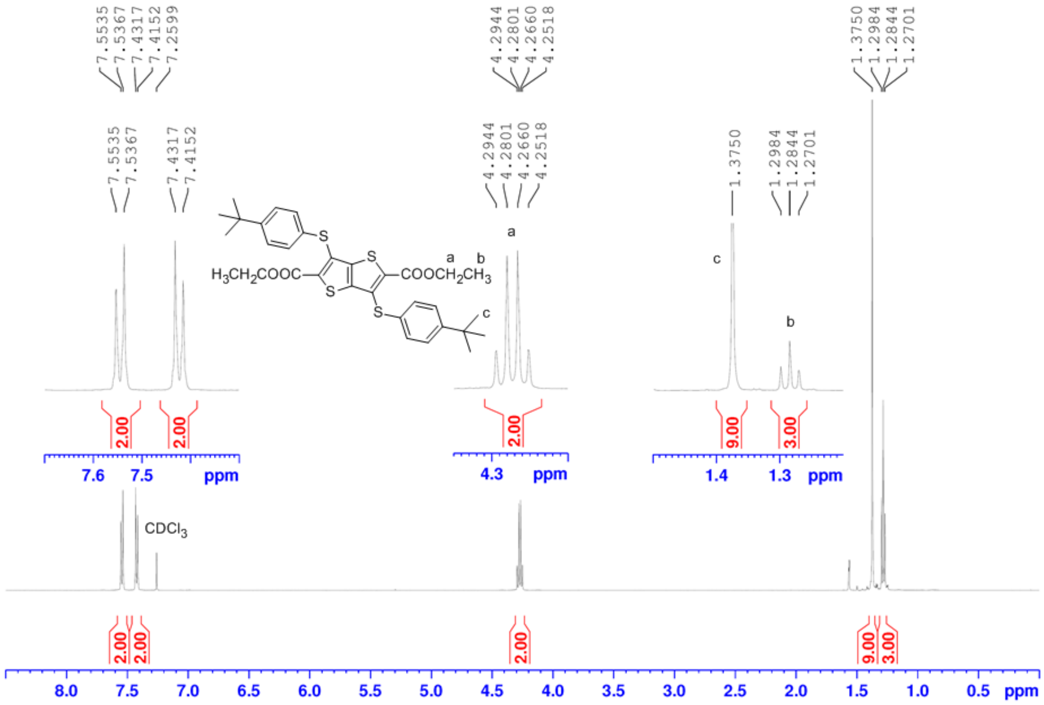

Fig. S12. ^{1}H NMR spectrum of compound **8** (500 MHz, $CDCl_3$, rt).

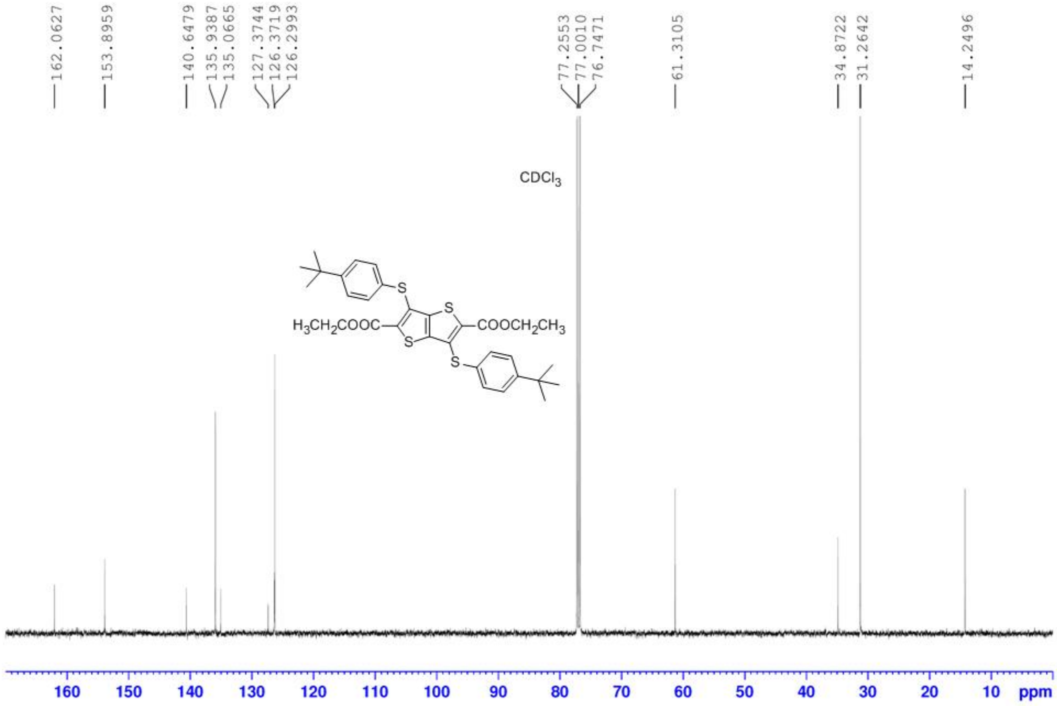

Fig. S13. ^{13}C NMR spectrum of compound **8** (125 MHz, $CDCl_3$, rt).

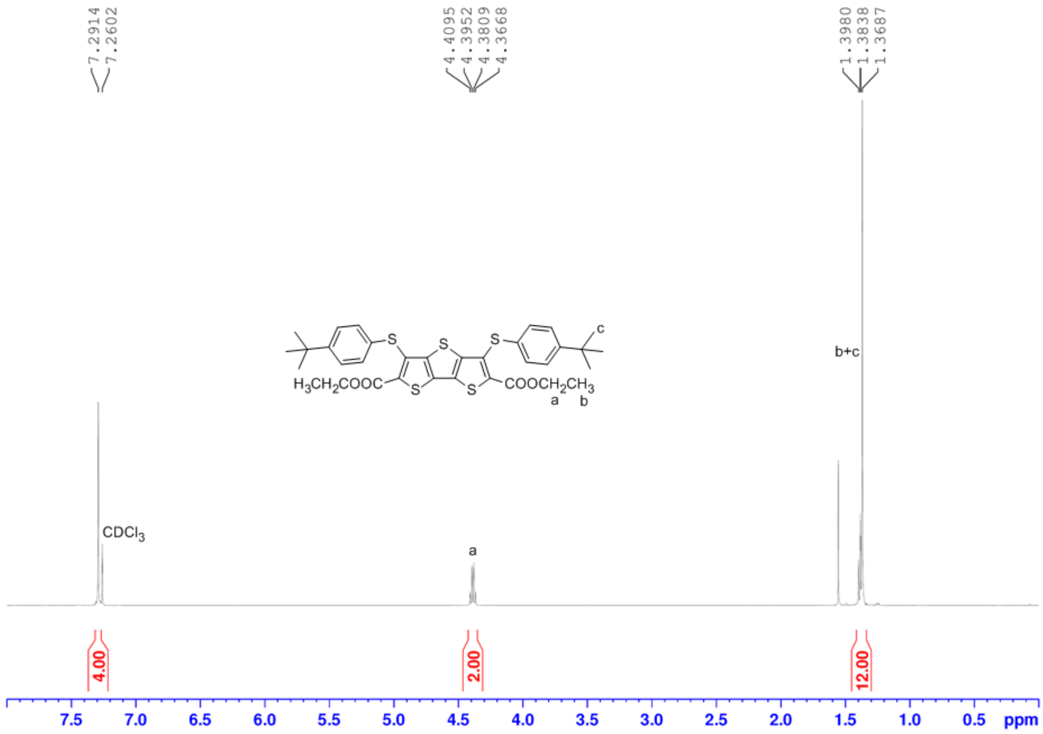

Fig. S14. ^{1}H NMR spectrum of compound **9** (500 MHz, $CDCl_3$, rt).

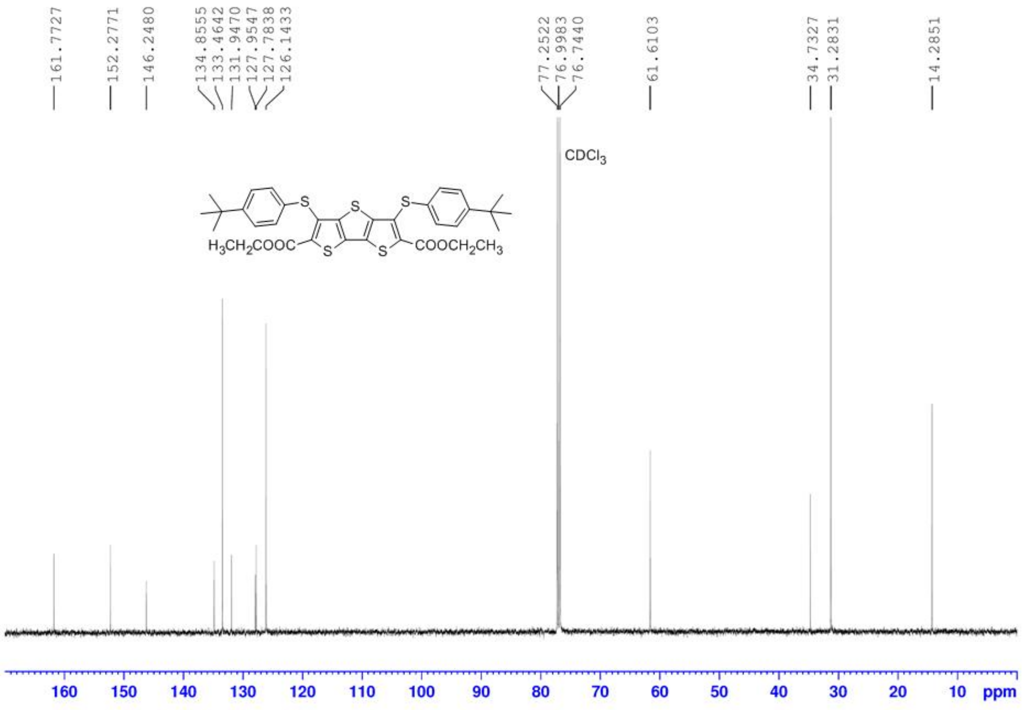

Fig. S15. ^{13}C NMR spectrum of compound **9** (125 MHz, $CDCl_3$, rt).

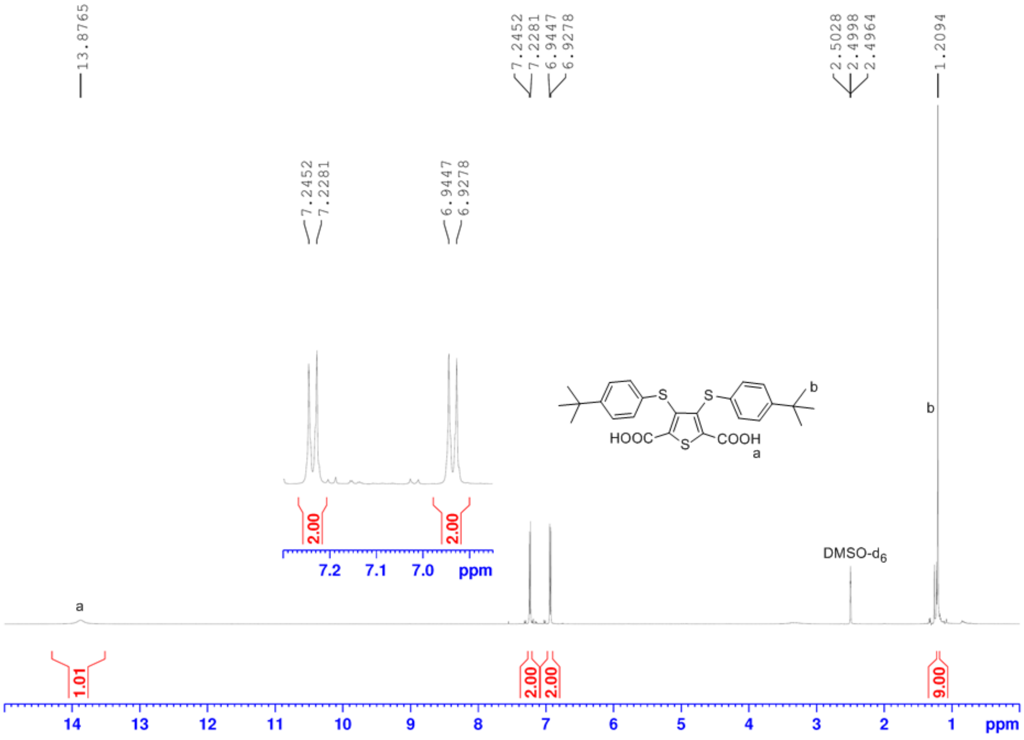

Fig. S16. ^{1}H NMR spectrum of compound **10** (500 MHz, DMSO-d_6, rt).

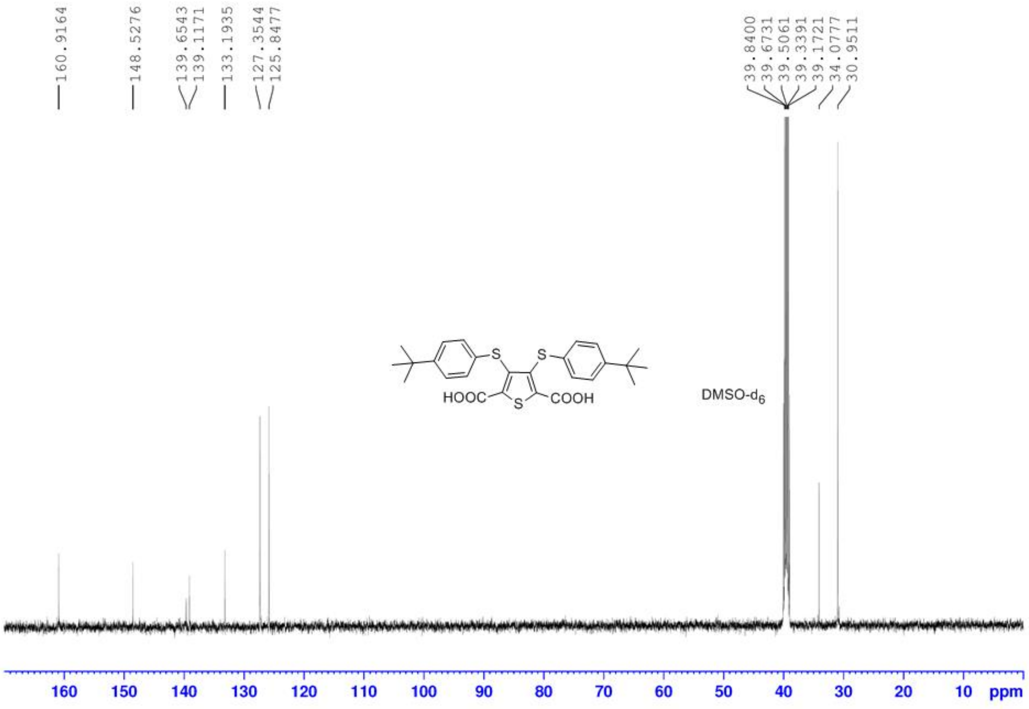

Fig. S17. ^{13}C NMR spectrum of compound **10** (125 MHz, DMSO-d_6, rt).

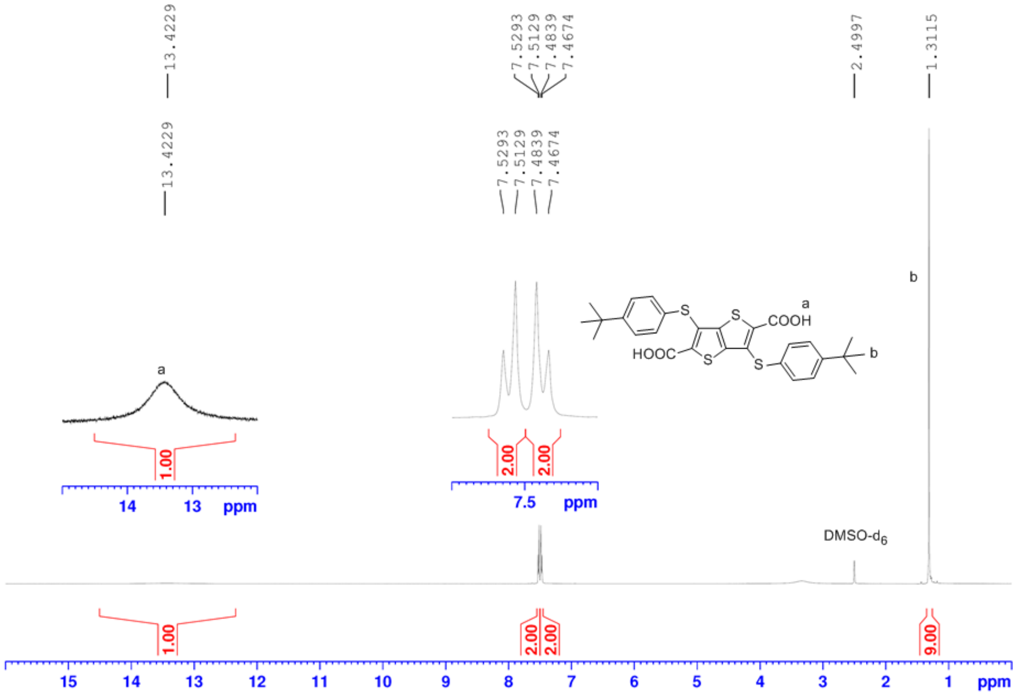

Fig. S18. ^{1}H NMR spectrum of compound **11** (500 MHz, DMSO-d_6, rt).

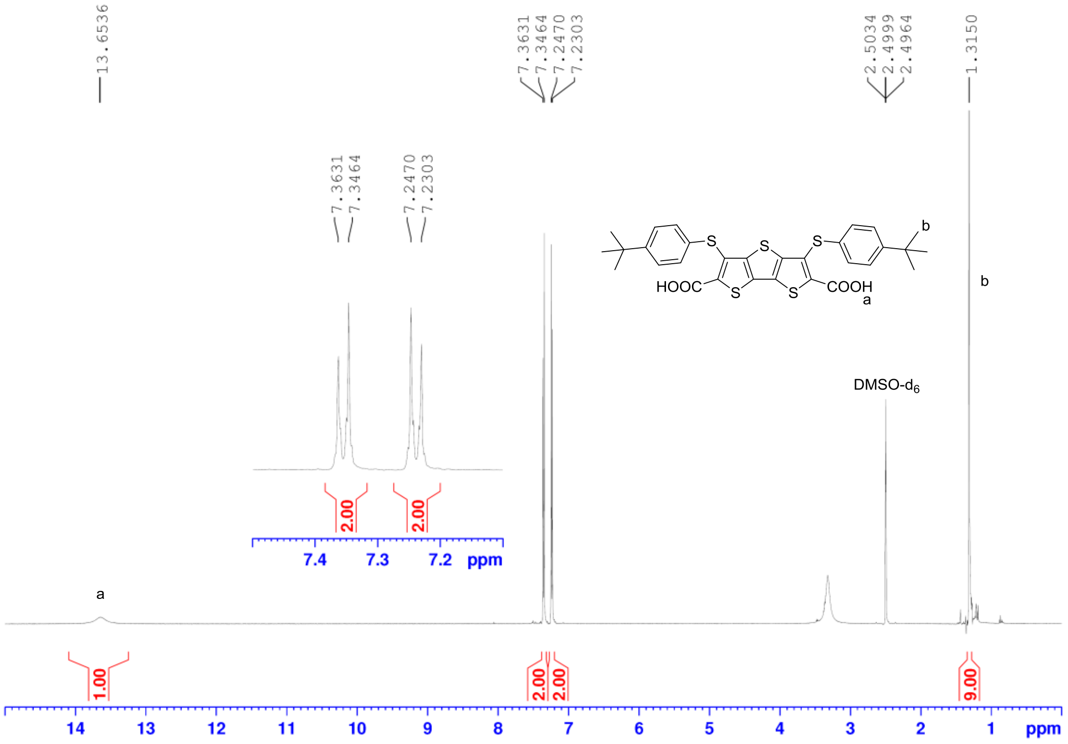

Fig. S19. ^{1}H NMR spectrum of compound **12** (500 MHz, DMSO-d_6, rt).

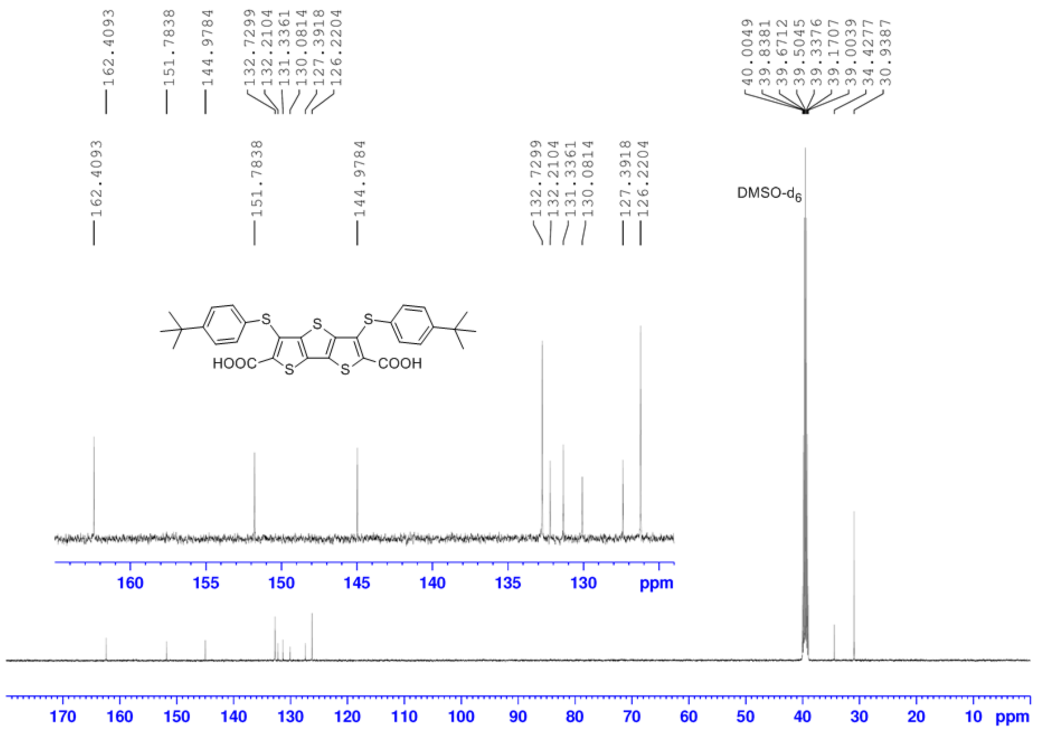

Fig. S20. ^{13}C NMR spectrum of compound **12** (125 MHz, DMSO-d_6, rt).

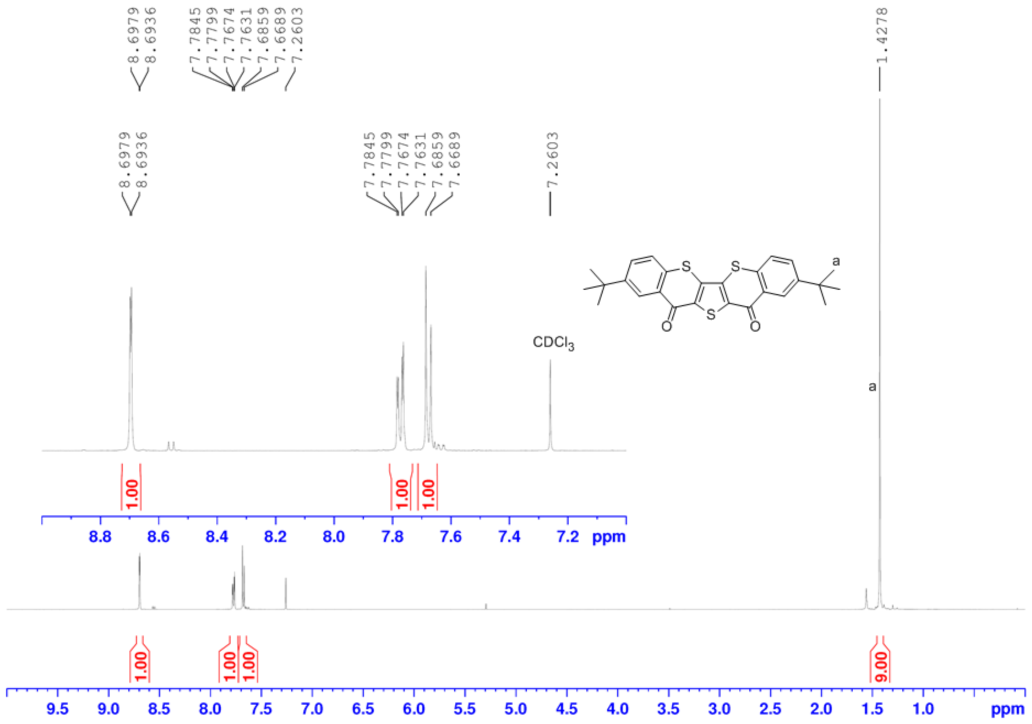

Fig. S21. ^{1}H NMR spectrum of compound **13** (500 MHz, $CDCl_3$, rt).

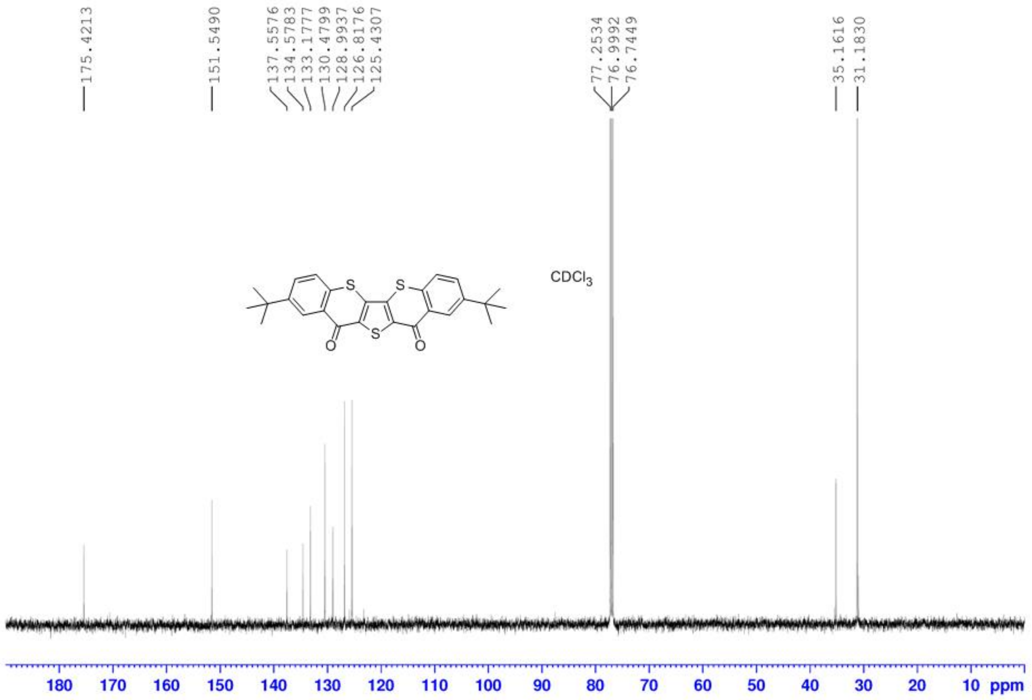

Fig. S22. ^{13}C NMR spectrum of compound **13** (125 MHz, $CDCl_3$, rt).

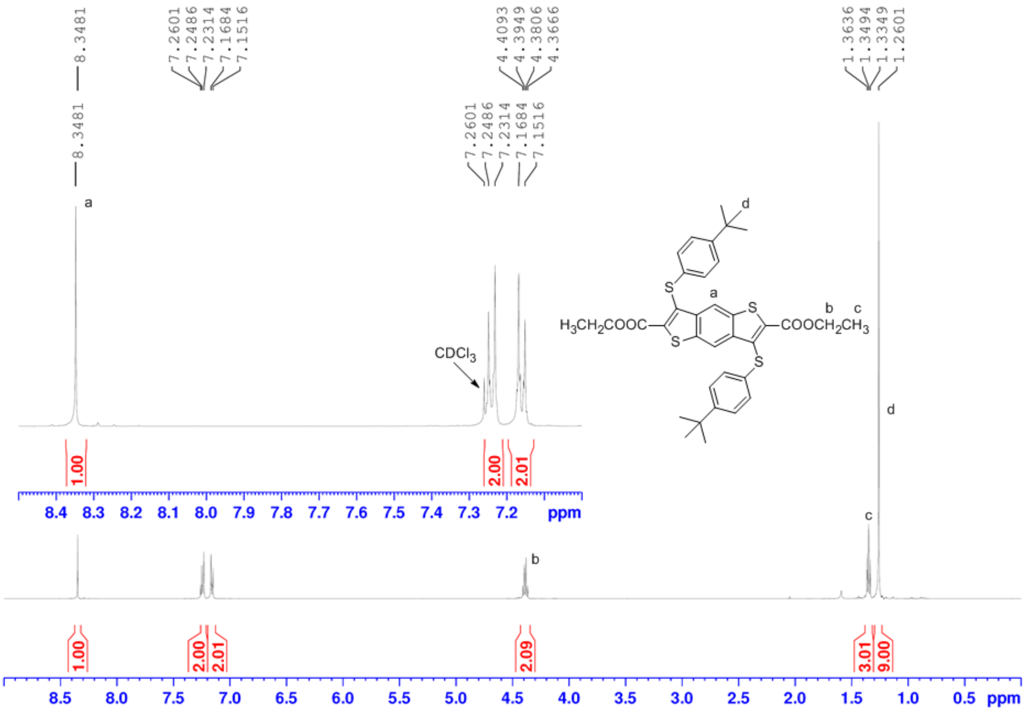

Fig. S23. ^{1}H NMR spectrum of compound **17** (500 MHz, $CDCl_3$, rt).

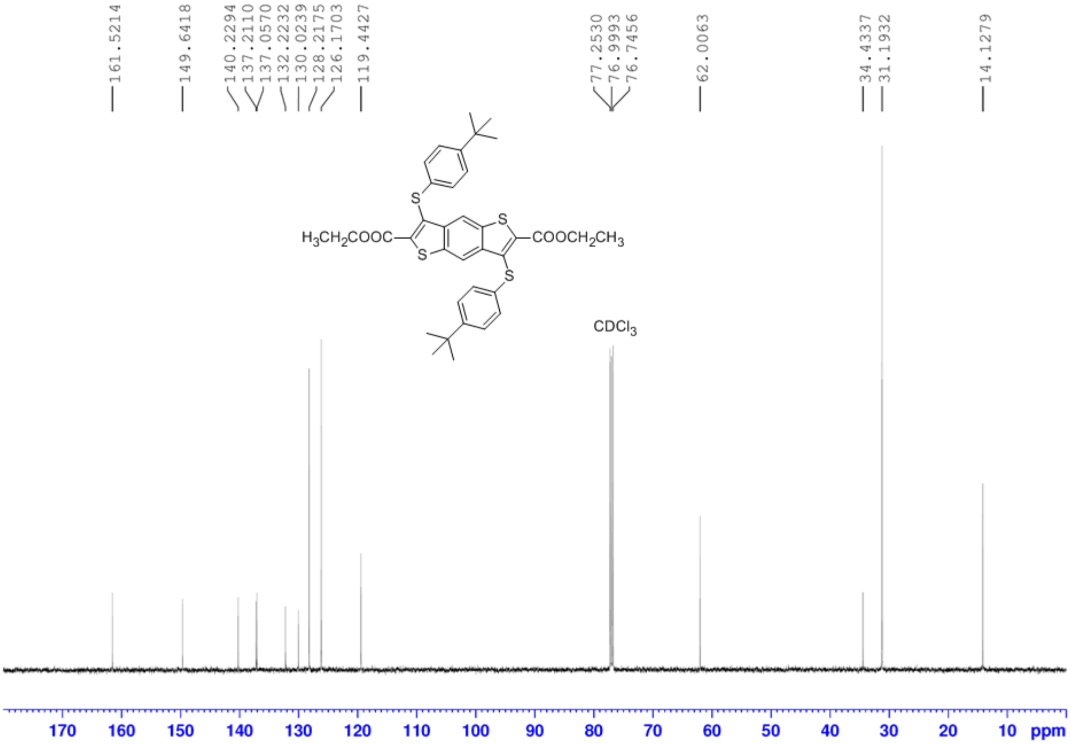

Fig. S24. ^{13}C NMR spectrum of compound **17** (125 MHz, $CDCl_3$, rt).

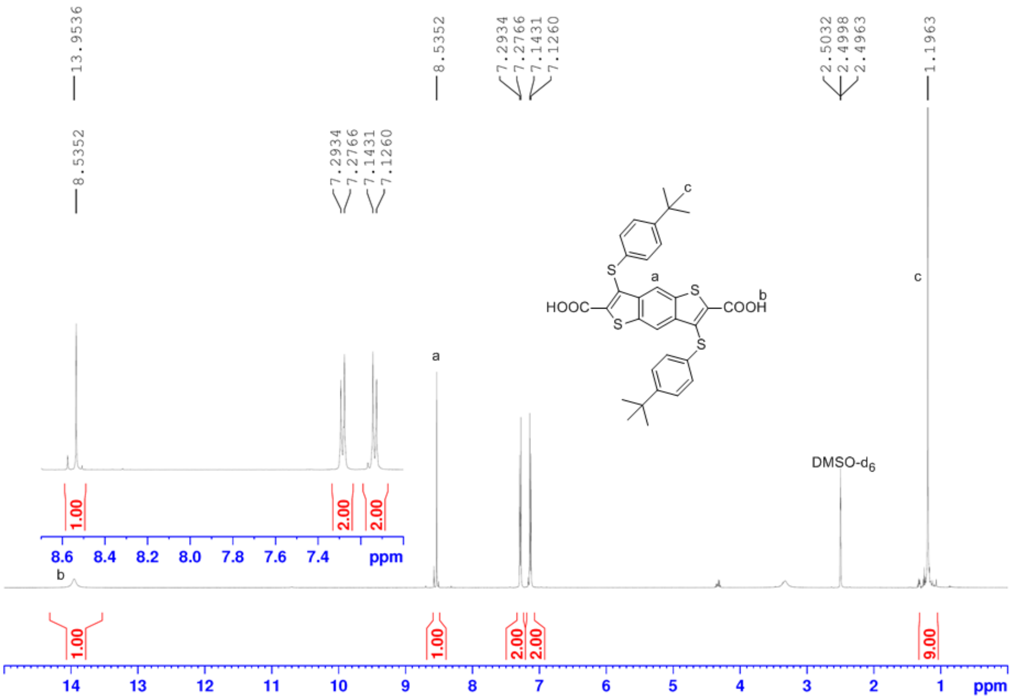

Fig. S25. ^{1}H NMR spectrum of compound **18** (500 MHz, DMSO-d_6, rt).

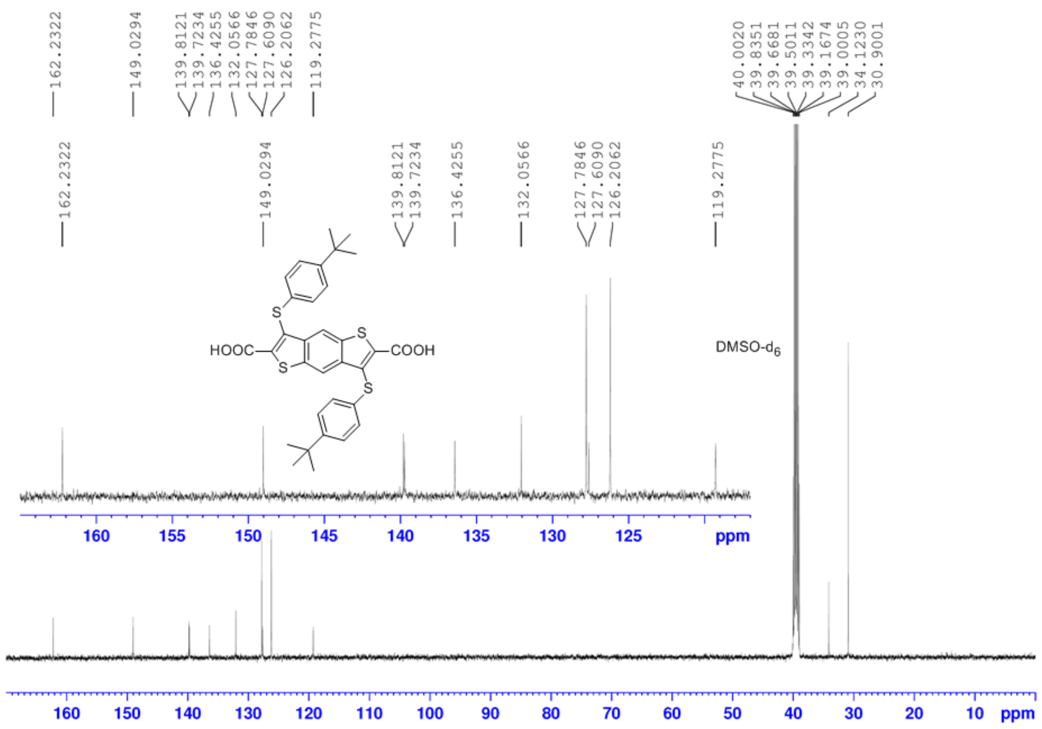

Fig. S26. ^{13}C NMR spectrum of compound **18** (125 MHz, DMSO-d$_6$, rt).

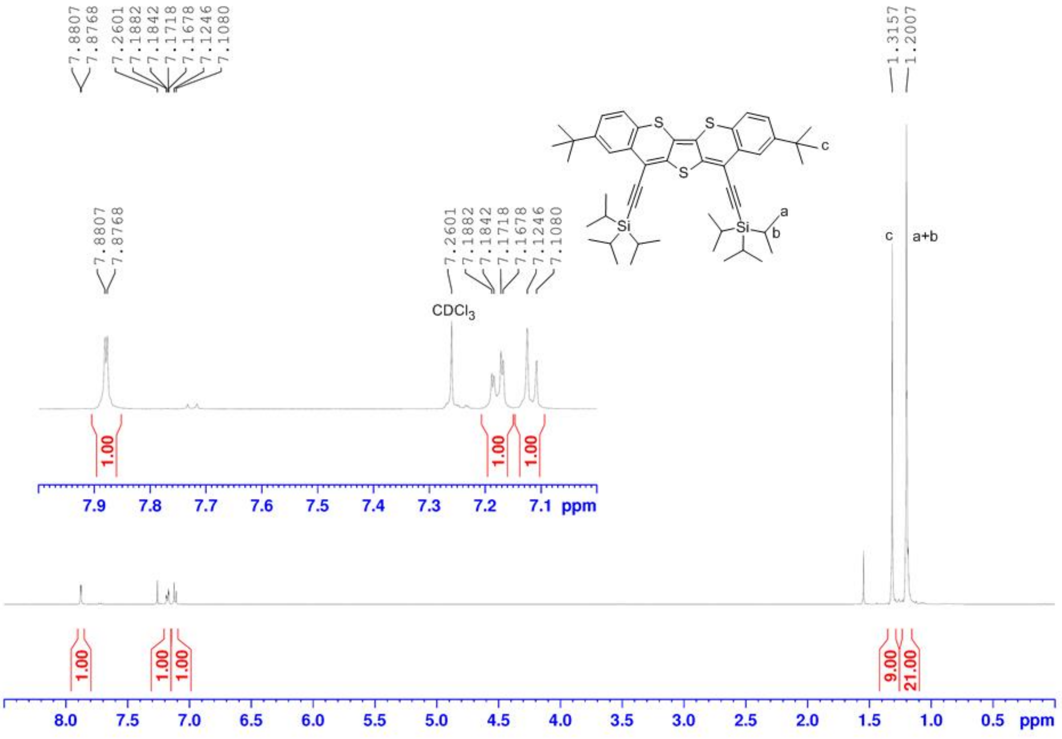

Fig. S27. ^{1}H NMR spectrum of compound **Th1-TIPS** (500 MHz, $CDCl_3$, rt).

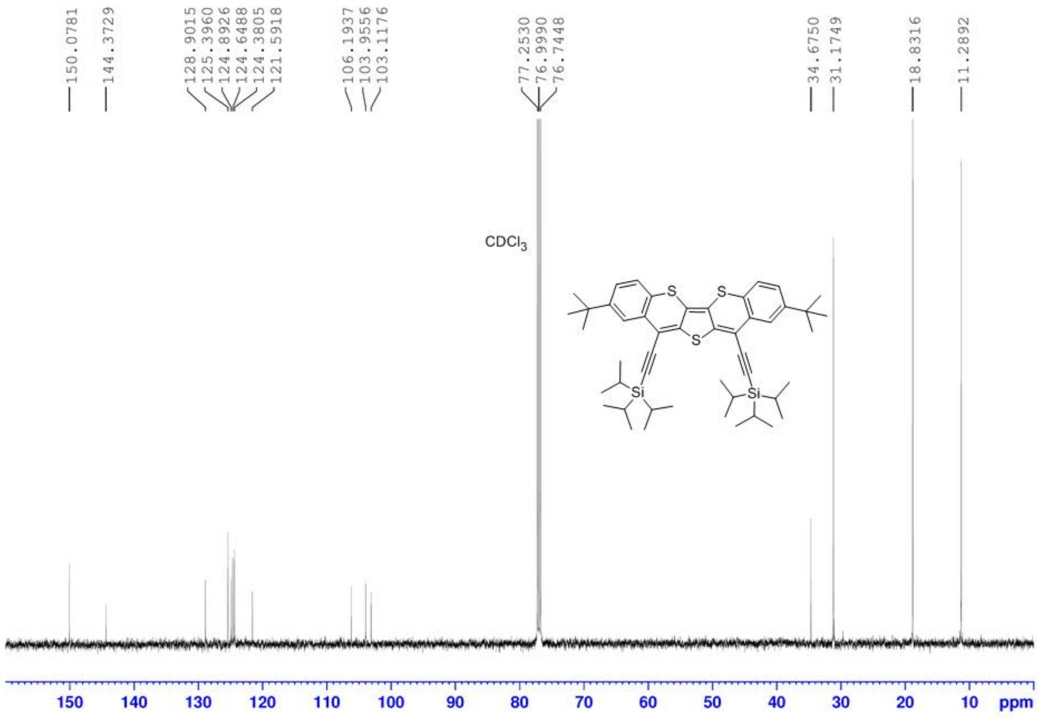

Fig. S28. ^{13}C NMR spectrum of compound **Th1-TIPS** (125 MHz, $CDCl_3$, rt).

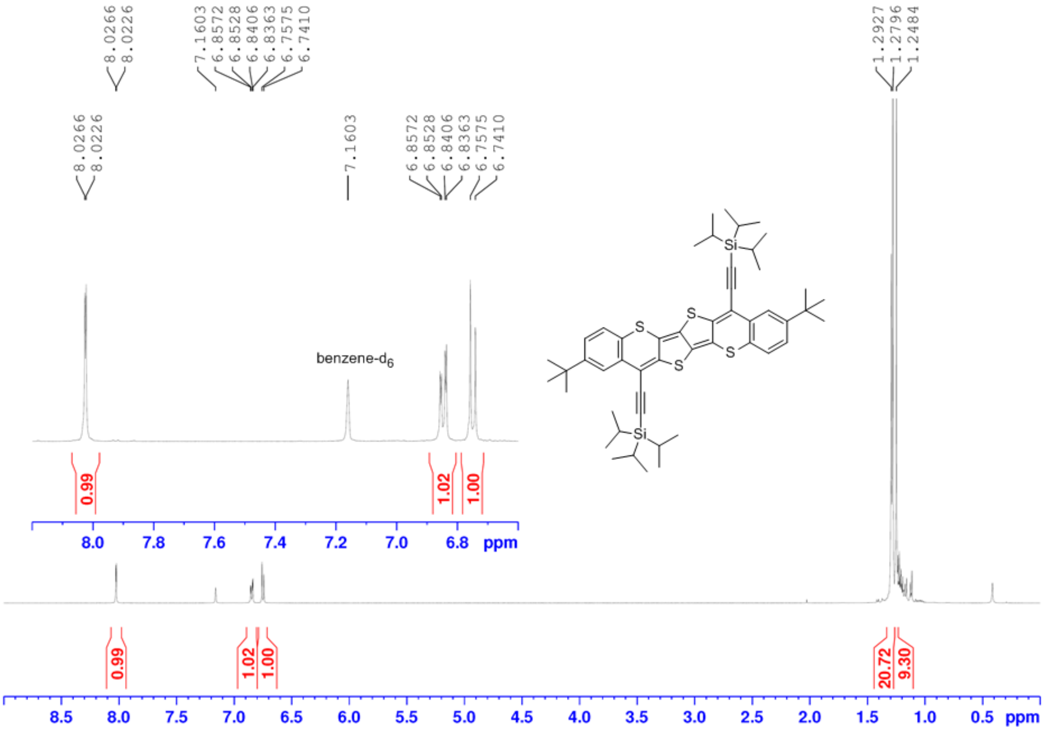

Fig. S29. ^{1}H NMR spectrum of compound **Th2-TIPS** (500 MHz, benzene-d_6, rt).

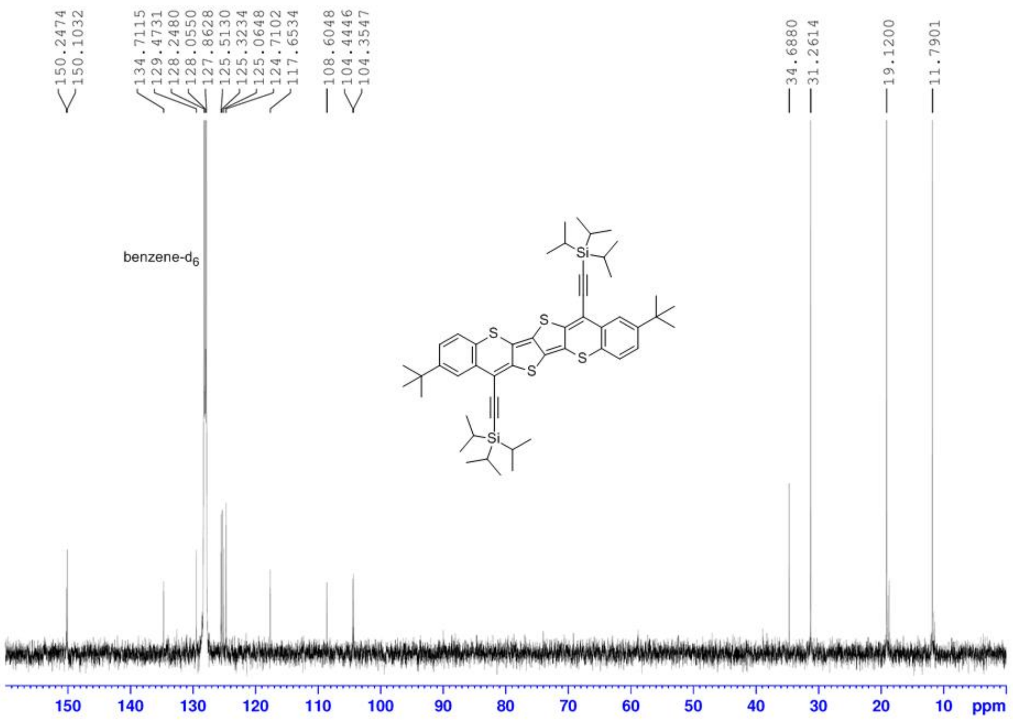

Fig. S30. ^{13}C NMR spectrum of compound **Th2-TIPS** (125 MHz, benzene-d$_6$, rt).

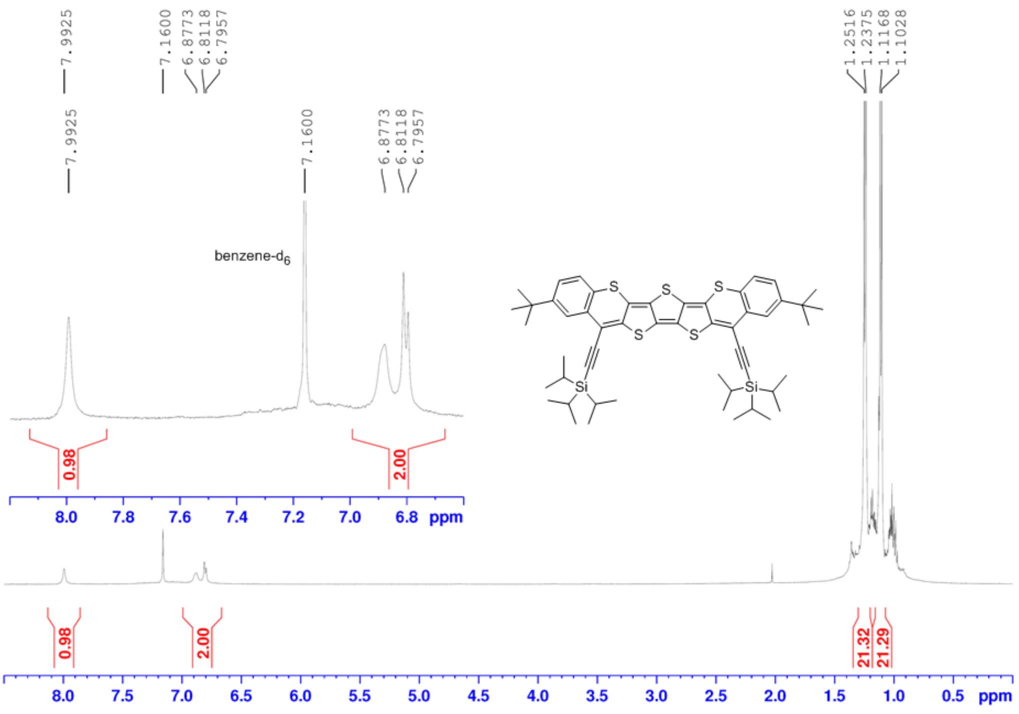

Fig. S31. ^{1}H NMR spectrum of compound **Th3-TIPS** (500 MHz, benzene-d$_6$, rt).

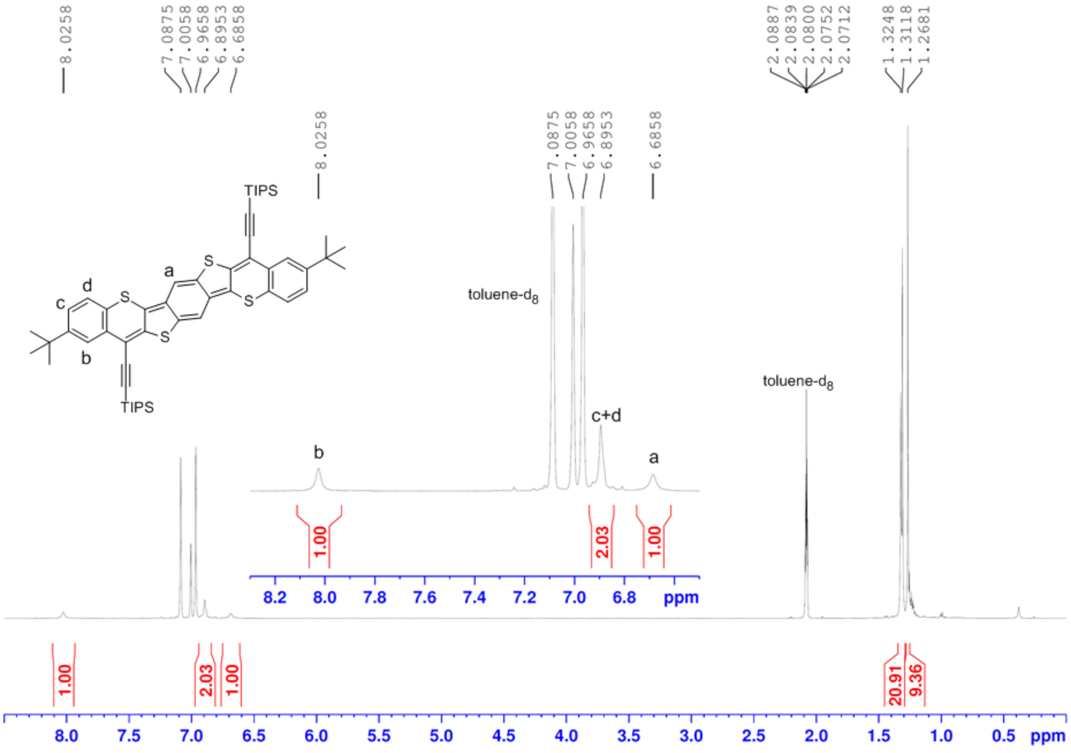

Fig. S32. ^{1}H NMR spectrum of compound **BDTh-TIPS** (500 MHz, toluene-d_8, rt).

8. Appendix: Energies and Cartesian Coordinates

8.1 Compound BDTh-TIPS

Natural Orbital Coefficient
HOMO: 1.34585 LUMO: 0.65415
singlet biradical character y_0 = 0.382

Singlet Biradical Open Shell:

Sum of electronic and zero-point Energies = -4268.041914
Sum of electronic and thermal Energies = -4267.974086
Sum of electronic and thermal Enthalpies = -4267.973141
Sum of electronic and thermal Free Energies = -4268.151390

S	-2.88466000	1.16558200	-0.06412600
S	2.95054500	-1.08542100	0.01838200
C	-1.20947200	0.64155400	-0.04129900
C	2.44757900	1.47957600	-0.03239000
C	3.51719300	0.58337700	-0.00934700
C	-1.10293300	-0.77678800	-0.01499700
C	-2.38033400	-1.39906400	-0.01392100
C	-3.45051000	-0.50370000	-0.03855900
C	-4.81013900	-0.83314500	-0.04325500
C	-4.33955400	-3.30296000	0.00106300
C	-5.24576700	-2.22588700	-0.02789000
S	-2.58031200	-3.12863300	0.01998000
C	-4.81832400	-4.60913300	0.01800800
C	-6.61558300	-2.53077300	-0.04129400
C	-6.17905400	-4.87094800	0.00666600
C	-7.11101400	-3.82904900	-0.02394800
H	-4.11347100	-5.43530400	0.04087700
H	-6.50521100	-5.90343500	0.02161600
H	-7.30386100	-1.69375400	-0.06694300
C	-5.76374600	0.20778500	-0.05951600
C	-8.62553800	-4.06633900	-0.04246800
C	-9.26828100	-3.38240000	1.17740400
C	-9.21937100	-3.47188100	-1.33203700
C	-8.97789600	-5.55787700	0.00497400
H	-9.09503400	-2.30237700	1.17783300
H	-10.35143000	-3.54555400	1.17566800
H	-8.86497800	-3.78628300	2.11144100
H	-8.78500100	-3.94597500	-2.21797000
H	-10.30297700	-3.63021800	-1.35850300

H	-9.03742100	-2.39593000	-1.40506100
H	-8.60205100	-6.03687300	0.91483100
H	-10.06550000	-5.67831200	-0.00587700
H	-8.57838500	-6.09918300	-0.85859100
C	-6.56697200	1.12541300	-0.07138800
Si	-7.76818400	2.52690000	-0.05868900
C	-9.00615900	2.23417600	-1.48255800
C	-10.32565200	3.00557700	-1.32872700
C	-9.29370700	0.75127800	-1.75542800
H	-8.49159800	2.63369800	-2.36936000
H	-10.17513200	4.07558700	-1.15642400
H	-10.93908300	2.90129600	-2.23186000
H	-10.91733000	2.61656700	-0.49313000
H	-8.37758500	0.18961300	-1.95754900
H	-9.79372300	0.27660700	-0.90342500
H	-9.95612500	0.63936600	-2.62260000
C	-6.72586400	4.08055400	-0.40984500
C	-5.78281200	4.41655800	0.75303900
C	-7.52737800	5.31495100	-0.84373800
H	-6.09924800	3.78423200	-1.26362300
H	-5.15784600	3.56217300	1.03224600
H	-5.11343400	5.24292600	0.48500500
H	-6.33901800	4.72796100	1.64459500
H	-8.13354400	5.12005000	-1.73386400
H	-8.20046600	5.66656100	-0.05450600
H	-6.85261100	6.14564200	-1.08375700
C	-8.52215200	2.52765700	1.69147400
C	-9.31421400	1.24680700	1.98853100
C	-9.34682900	3.77148100	2.05072800
H	-7.63996000	2.52142100	2.34769800
H	-8.72214400	0.34697600	1.79177700
H	-9.62343900	1.21621400	3.04034400
H	-10.22568600	1.18733500	1.38327700
H	-8.76918900	4.69491200	1.94733400
H	-10.23868100	3.86678600	1.42380700
H	-9.68851500	3.71604500	3.09160900
C	4.87738000	0.91090500	-0.00839600
C	5.31442500	2.30257900	-0.03225100
C	4.40954400	3.38085500	-0.05954200
C	6.68456400	2.60527000	-0.02950800
C	4.89038800	4.68615600	-0.08416900
C	7.18212700	3.90249300	-0.05372600
C	6.25162900	4.94580100	-0.08177400

H	7.37090800	1.76654700	-0.00776500
H	6.57933700	5.97771300	-0.10203200
H	4.18680100	5.51343400	-0.10581600
C	8.69713000	4.13712700	-0.04352400
C	9.29457600	3.55063000	1.24798600
C	9.33401100	3.44289500	-1.26058900
C	9.05209000	5.62758700	-0.10265100
H	8.86406700	4.03090900	2.13242700
H	9.11027000	2.47553200	1.32839500
H	10.37863400	3.70623200	1.26964000
H	8.92833700	3.84111100	-2.19603800
H	10.41751300	3.60376800	-1.26364300
H	9.15817500	2.36348000	-1.25230700
H	10.13996000	5.74607800	-0.09843200
H	8.67218400	6.10108200	-1.01368700
H	8.65820200	6.17544200	0.75934700
C	5.82577800	-0.13504200	0.01282800
C	6.61026500	-1.06869300	0.02922000
S	2.64981700	3.20904100	-0.06566300
Si	7.76410100	-2.51170400	0.04084800
C	6.96221500	-3.79430300	-1.11369100
C	7.54174100	-5.21287100	-1.02833200
C	6.93877800	-3.29896000	-2.56641000
H	5.91905300	-3.84615400	-0.77079300
H	7.46231700	-5.63086600	-0.02052400
H	7.00278300	-5.88747100	-1.70481100
H	8.59750800	-5.24551000	-1.31726100
H	7.94956500	-3.23176700	-2.98488800
H	6.37105000	-3.98813500	-3.20301600
H	6.47587000	-2.31097500	-2.65222600
C	9.42001000	-1.83667400	-0.61482500
C	10.44572700	-2.90003200	-1.03030100
C	10.04310900	-0.83439100	0.36647900
H	9.14268600	-1.27901300	-1.52063200
H	10.06241800	-3.55604400	-1.81715100
H	11.35622600	-2.42507700	-1.41589900
H	10.74562400	-3.53404300	-0.18941900
H	9.33840800	-0.04430300	0.64723700
H	10.37015800	-1.32591900	1.28958500
H	10.92487900	-0.35256600	-0.07307900
C	7.85279100	-3.08189400	1.85499500
C	8.98552500	-4.06398300	2.18415600
C	6.49945400	-3.62770300	2.33215800

H	8.04808200	-2.15787500	2.41760200
H	9.97220400	-3.64672700	1.96192500
H	8.97257500	-4.31916700	3.25089500
H	8.89172200	-5.00271800	1.62855000
H	5.68583100	-2.91846900	2.15000900
H	6.24288600	-4.56527900	1.82583500
H	6.52245200	-3.83812300	3.40817600
C	0.16821200	-1.37767500	0.00551800
C	-0.10166400	1.45906100	-0.04828300
C	1.27593400	-0.56026700	-0.00139900
C	1.16964200	0.85814100	-0.02845400
H	0.26861500	-2.45810100	0.02552300
H	-0.20211500	2.53947600	-0.06878000

Singlet Closed Shell:

Sum of electronic and zero-point Energies = -4268.028580
Sum of electronic and thermal Energies = -4267.960772
Sum of electronic and thermal Enthalpies = -4267.959828
Sum of electronic and thermal Free Energies = -4268.138221

S	-2.89106600	1.17775400	-0.05702100
S	2.95745900	-1.09688300	0.02083800
C	-1.20894900	0.65814500	-0.03565000
C	2.41199600	1.47792300	-0.02882400
C	3.52339800	0.57396300	-0.00664400
C	-1.11201800	-0.78450200	-0.00969200
C	-2.34418900	-1.39664900	-0.00797100
C	-3.45617600	-0.49364500	-0.03271000
C	-4.78093500	-0.83162200	-0.03798600
C	-4.32053000	-3.30447400	0.00719200
C	-5.22564300	-2.23111100	-0.02249200
S	-2.56106200	-3.12781900	0.02727200
C	-4.79848800	-4.61007800	0.02408800
C	-6.59262100	-2.53019300	-0.03689200
C	-6.16046100	-4.87045700	0.01186100
C	-7.09080000	-3.82988700	-0.01963900
H	-4.09429200	-5.43675700	0.04756700
H	-6.48690500	-5.90293500	0.02685700
H	-7.28008800	-1.69253400	-0.06316200
C	-5.74904200	0.20848100	-0.05626600
C	-8.60542400	-4.06477700	-0.03944500
C	-9.24861100	-3.37881700	1.17906900
C	-9.19713900	-3.47069700	-1.33017500

C	-8.95978900	-5.55575200	0.00903400
H	-9.07381400	-2.29901000	1.17885900
H	-10.33203000	-3.54021500	1.17623400
H	-8.84698900	-3.78253000	2.11389800
H	-8.76301100	-3.94679000	-2.21515000
H	-10.28107300	-3.62673500	-1.35734800
H	-9.01291900	-2.39519400	-1.40443900
H	-8.58522800	-6.03430200	0.91965400
H	-10.04753100	-5.67493800	-0.00270600
H	-8.56006000	-6.09827300	-0.85366500
C	-6.55398100	1.12076600	-0.06972400
Si	-7.75861400	2.52109400	-0.06324600
C	-8.98786500	2.22481800	-1.49352600
C	-10.30995000	2.99314300	-1.34611900
C	-9.27066900	0.74125300	-1.76769400
H	-8.46982600	2.62558200	-2.37772100
H	-10.16292600	4.06371900	-1.17425100
H	-10.91910400	2.88639000	-2.25182400
H	-10.90418200	2.60340100	-0.51267900
H	-8.35240600	0.18157200	-1.96546900
H	-9.77381500	0.26547800	-0.91817000
H	-9.92861900	0.62797700	-2.63805600
C	-6.71692100	4.07607200	-0.40904100
C	-5.78114500	4.41443400	0.75907500
C	-7.51863600	5.30865100	-0.84782400
H	-6.08489600	3.78072900	-1.25914900
H	-5.15590700	3.56156900	1.04220700
H	-5.11200100	5.24200500	0.49430800
H	-6.34301300	4.72520800	1.64729200
H	-8.11936100	5.11231700	-1.74131900
H	-8.19684800	5.65908000	-0.06245600
H	-6.84420700	6.14061700	-1.08424000
C	-8.51981800	2.51871300	1.68345100
C	-9.31048600	1.23612000	1.97658900
C	-9.34870300	3.76081000	2.03905300
H	-7.64052200	2.51431900	2.34356200
H	-8.71547600	0.33757900	1.78287300
H	-9.62473600	1.20494500	3.02687200
H	-10.21884700	1.17461500	1.36688700
H	-8.77261000	4.68545600	1.93805500
H	-10.23810100	3.85414700	1.40834700
H	-9.69464900	3.70462600	3.07846000
C	4.84871900	0.91002600	-0.00682400

C	5.29487400	2.30837600	-0.02986400
C	4.39115100	3.38301400	-0.05621000
C	6.66220900	2.60508100	-0.02684000
C	4.87131100	4.68774900	-0.07999200
C	7.16263900	3.90364600	-0.05018500
C	6.23382300	4.94576300	-0.07750200
H	7.34764600	1.76561100	-0.00566000
H	6.56194300	5.97764200	-0.09709400
H	4.16846800	5.51563900	-0.10099600
C	8.67779000	4.13556400	-0.03961600
C	9.27406600	3.54771700	1.25184200
C	9.31390300	3.44064200	-1.25671500
C	9.03501600	5.62544500	-0.09822700
H	8.84467100	4.02900800	2.13626800
H	9.08731600	2.47301700	1.33237100
H	10.35850200	3.70072300	1.27347800
H	8.90930300	3.84003200	-2.19212400
H	10.39772700	3.59939200	-1.25932000
H	9.13611200	2.36150500	-1.24903600
H	10.12305200	5.74247000	-0.09381500
H	8.65578300	6.09969100	-1.00915300
H	8.64168000	6.17353100	0.76387700
C	5.81175200	-0.13507300	0.01284900
C	6.59785100	-1.06365900	0.02740300
S	2.63124400	3.20887800	-0.06233900
Si	7.75448500	-2.50614800	0.03560900
C	6.95215500	-3.78603900	-1.12119800
C	7.53279600	-5.20438100	-1.03920400
C	6.92748400	-3.28750600	-2.57281500
H	5.90930500	-3.83922400	-0.77762700
H	7.45452200	-5.62451100	-0.03219500
H	6.99364800	-5.87788400	-1.71658500
H	8.58831000	-5.23569600	-1.32918100
H	7.93793000	-3.21878000	-2.99184700
H	6.35974800	-3.97572500	-3.21039700
H	6.46370700	-2.29973300	-2.65625100
C	9.40877500	-1.82760900	-0.61973300
C	10.43536500	-2.88916700	-1.03772400
C	10.03132000	-0.82645500	0.36310000
H	9.13029700	-1.26864100	-1.52439500
H	10.05238900	-3.54402400	-1.82569300
H	11.34517200	-2.41249200	-1.42277200
H	10.73626200	-3.52446700	-0.19817700

H	9.32599700	-0.03765600	0.64589400
H	10.35965100	-1.31936700	1.28500000
H	10.91214100	-0.34275000	-0.07622900
C	7.84282200	-3.07765500	1.84906600
C	8.97676100	-4.05895900	2.17661300
C	6.49015600	-3.62584300	2.32538800
H	8.03714900	-2.15418000	2.41289000
H	9.96295800	-3.64054600	1.95442900
H	8.96443200	-4.31527700	3.24306000
H	8.88351000	-4.99718600	1.62004700
H	5.67545700	-2.91766500	2.14401400
H	6.23503200	-4.56326300	1.81806700
H	6.51347400	-3.83735900	3.40115900
C	0.18876300	-1.38484800	0.01019100
C	-0.12172500	1.46704400	-0.04265900
C	1.27594100	-0.57603700	0.00285800
C	1.17926500	0.86665100	-0.02386300
H	0.28798600	-2.46518200	0.02954100
H	-0.22101500	2.54737500	-0.06220600

Triplet:

Sum of electronic and zero-point Energies = -4268.035480
Sum of electronic and thermal Energies = -4267.967646
Sum of electronic and thermal Enthalpies = -4267.966702
Sum of electronic and thermal Free Energies = -4268.145995

S	-2.88268300	1.16156700	-0.06916100
S	2.94831700	-1.08170200	0.01634500
C	-1.20967200	0.63602300	-0.04566400
C	2.46346200	1.47982500	-0.03574800
C	3.51457900	0.58719300	-0.01186200
C	-1.09886000	-0.77389100	-0.01879900
C	-2.39652200	-1.39964900	-0.01785600
C	-3.44818300	-0.50777100	-0.04268500
C	-4.82505200	-0.83389000	-0.04719500
C	-4.34789000	-3.30152100	-0.00354000
C	-5.25541700	-2.22263600	-0.03227900
S	-2.58955800	-3.12799500	0.01579000
C	-4.82668000	-4.60831500	0.01309100
C	-6.62676400	-2.53139300	-0.04558100
C	-6.18651800	-4.87137100	0.00181300
C	-7.11995800	-3.82901100	-0.02842900
H	-4.12115100	-5.43393200	0.03573800

H	-6.51212900	-5.90398100	0.01652000
H	-7.31581800	-1.69501600	-0.07099900
C	-5.77167300	0.20717400	-0.06200300
C	-8.63428400	-4.06844800	-0.04661600
C	-9.27744400	-3.38587100	1.17379800
C	-9.22924900	-3.47415900	-1.33573600
C	-8.98476500	-5.56048000	0.00026400
H	-9.10560600	-2.30564200	1.17456100
H	-10.36035400	-3.55056400	1.17238300
H	-8.87325400	-3.78958400	2.10752800
H	-8.79421200	-3.94690800	-2.22206300
H	-10.31256600	-3.63440600	-1.36211800
H	-9.04916200	-2.39786000	-1.40801600
H	-8.60816300	-6.03947800	0.90980700
H	-10.07222600	-5.68215700	-0.01031300
H	-8.58494200	-6.10096200	-0.86367500
C	-6.57395500	1.12762300	-0.07260400
Si	-7.77338400	2.52948900	-0.05474300
C	-9.01937000	2.23771200	-1.47209100
C	-10.33738600	3.01030000	-1.31209500
C	-9.30945100	0.75496900	-1.74308600
H	-8.50887100	2.63649500	-2.36157600
H	-10.18500400	4.08002900	-1.13974600
H	-10.95485200	2.90725500	-2.21263900
H	-10.92595400	2.62139500	-0.47426000
H	-8.39469700	0.19269900	-1.94973500
H	-9.80529200	0.28087000	-0.88831300
H	-9.97644100	0.64318900	-2.60677900
C	-6.73191400	4.08245200	-0.41213400
C	-5.78202000	4.41782200	0.74529200
C	-7.53469200	5.31744400	-0.84192600
H	-6.11042000	3.78530400	-1.26936800
H	-5.15646600	3.56278900	1.02121800
H	-5.11325000	5.24344200	0.47338800
H	-6.33290400	4.72998700	1.63988600
H	-8.14620200	5.12262900	-1.72840500
H	-8.20286800	5.67006600	-0.04898600
H	-6.86056700	6.14741000	-1.08627700
C	-8.51946300	2.53266200	1.69904700
C	-9.31127900	1.25268600	2.00057500
C	-9.34139200	3.77737900	2.06148300
H	-7.63425600	2.52596700	2.35117500
H	-8.72108200	0.35221800	1.80113000

H	-9.61530500	1.22274400	3.05393600
H	-10.22581900	1.19393100	1.39990700
H	-8.76332900	4.70023400	1.95524800
H	-10.23587900	3.87331100	1.43843400
H	-9.67860300	3.72267700	3.10388000
C	4.89200700	0.91144500	-0.01018600
C	5.32374700	2.29914700	-0.03488700
C	4.41748800	3.37916500	-0.06319000
C	6.69540100	2.60584400	-0.03216500
C	4.89825400	4.68509100	-0.08861800
C	7.19062700	3.90244900	-0.05717500
C	6.25858300	4.94611000	-0.08612600
H	7.38259200	1.76784700	-0.00972700
H	6.58565800	5.97815400	-0.10703400
H	4.19392300	5.51173500	-0.11099700
C	8.70540400	4.13944600	-0.04692600
C	9.30346700	3.55448700	1.24498800
C	9.34337600	3.44547800	-1.26355700
C	9.05833800	5.63039600	-0.10682300
H	8.87184200	4.03428600	2.12915100
H	9.12106000	2.47910200	1.32565300
H	10.38721900	3.71212300	1.26689100
H	8.93715000	3.84250600	-2.19927400
H	10.42661100	3.60809300	-1.26662300
H	9.16918000	2.36582600	-1.25460300
H	10.14605200	5.75024300	-0.10259900
H	8.67796200	6.10294900	-1.01815200
H	8.66376500	6.17823600	0.75487100
C	5.83333400	-0.13464000	0.01251200
C	6.61680700	-1.07105600	0.03047700
S	2.65865500	3.20810600	-0.06953300
Si	7.76912500	-2.51418000	0.04525000
C	6.96840600	-3.79880000	-1.10811300
C	7.54738200	-5.21739500	-1.01970600
C	6.94673200	-3.30597500	-2.56169600
H	5.92482000	-3.84981000	-0.76634800
H	7.46653100	-5.63371100	-0.01131800
H	7.00913500	-5.89299300	-1.69578300
H	8.60352200	-5.25081300	-1.30718200
H	7.95802900	-3.23966200	-2.97910800
H	6.37962500	-3.99610200	-3.19785000
H	6.48420400	-2.31799800	-2.64966000
C	9.42632600	-1.84119000	-0.60967900

C	10.45196200	-2.90557600	-1.02267900
C	10.04894800	-0.83781300	0.37080100
H	9.15019400	-1.28464800	-1.51652900
H	10.06911100	-3.56242300	-1.80905700
H	11.36310300	-2.43159200	-1.40800000
H	10.75071600	-3.53865200	-0.18068900
H	9.34430500	-0.04697600	0.64960700
H	10.37468800	-1.32813600	1.29501800
H	10.93150400	-0.35712800	-0.06847000
C	7.85677800	-3.08319400	1.86003700
C	8.98884700	-4.06535900	2.19114400
C	6.50283300	-3.62784300	2.33678400
H	8.05198700	-2.15869500	2.42188300
H	9.97584900	-3.64853200	1.96954100
H	8.97481000	-4.31968200	3.25809300
H	8.89540100	-5.00452500	1.63621500
H	5.68980700	-2.91822300	2.15343300
H	6.24600400	-4.56567800	1.83106500
H	6.52492100	-3.83738100	3.41300800
C	0.16090700	-1.37510500	0.00225300
C	-0.09462400	1.45612300	-0.05276400
C	1.27586700	-0.55510500	-0.00457500
C	1.16528900	0.85487400	-0.03222700
H	0.26140200	-2.45561000	0.02280700
H	-0.19516700	2.53661300	-0.07390700

8.2 Compound Th1-TIPS

Singlet Closed Shell:

Sum of electronic and zero-point Energies = -3640.193518
Sum of electronic and thermal Energies = -3640.130899
Sum of electronic and thermal Enthalpies = -3640.129954
Sum of electronic and thermal Free Energies = -3640.295710

S	-0.01601300	-0.45904300	-0.04386800
C	-0.72462900	-2.99067700	-0.09018700
C	0.63766000	-3.00570000	-0.09997300
C	1.23681000	-1.69951400	-0.06887400
C	-1.29533900	-1.67192200	-0.05951200
C	-2.62297500	-1.36528400	-0.03825500
C	2.57049500	-1.42138700	-0.05133400
C	-3.34974100	-3.77330100	-0.03354000
C	-3.65992500	-2.40854800	-0.00310500

C	3.24673100	-3.84703500	-0.13248700
C	3.58636300	-2.48581300	-0.08472400
C	-4.37374900	-4.71808800	0.01411700
C	-5.01285800	-2.04232300	0.07878600
C	4.25013200	-4.80822000	-0.17169000
C	4.94475900	-2.15338200	-0.07416700
H	-4.12645000	-5.77550700	-0.00882100
H	3.98125700	-5.85995000	-0.21021400
C	-6.04607700	-2.96764200	0.12977700
C	-5.69541800	-4.32315500	0.09344900
C	5.58803600	-4.44327200	-0.16292500
C	5.96509500	-3.10055800	-0.11257500
H	-5.23077500	-0.98278800	0.10605600
H	-6.46418500	-5.08802100	0.13040900
H	5.19269500	-1.09886500	-0.03534800
H	6.33514600	-5.22661600	-0.19623000
C	-7.52070200	-2.56374600	0.23065600
C	-8.11498600	-3.13355800	1.53131500
C	-7.70856600	-1.04281500	0.24339100
C	-8.29112900	-3.13236800	-0.97434700
H	-8.04806400	-4.22484900	1.56609900
H	-7.59021400	-2.73818500	2.40672300
H	-9.17269200	-2.86193800	1.61680400
H	-7.33344500	-0.58019600	-0.67481000
H	-8.77409100	-0.80511300	0.32075100
H	-7.20294700	-0.57520300	1.09425600
H	-9.34921000	-2.85547000	-0.91327700
H	-7.89082100	-2.74083800	-1.91484000
H	-8.23581900	-4.22396700	-1.01618000
C	7.42879800	-2.64582400	-0.09081000
C	7.70872600	-1.89612700	1.22386900
C	8.40172500	-3.82689200	-0.18676400
C	7.69623600	-1.70677800	-1.28040400
H	7.06779000	-1.01670800	1.33261800
H	7.53629300	-2.54454000	2.08876700
H	8.74993700	-1.55691400	1.25431200
H	8.26318600	-4.39346600	-1.11318000
H	9.43129900	-3.45615000	-0.17678600
H	8.29218300	-4.51574400	0.65693200
H	8.74081300	-1.37693400	-1.27478700
H	7.50498800	-2.21444200	-2.23114100
H	7.06615300	-0.81355000	-1.24533500
C	-3.00311300	0.00613400	-0.03926600

C	2.98233500	-0.06037100	0.00480600
C	-3.28540500	1.18933100	-0.04949600
C	3.29759000	1.11306300	0.06377200
Si	-3.66241000	2.99780400	-0.07975200
Si	3.74792800	2.90338200	0.13832700
C	-4.87454100	3.24873800	-1.52762600
C	-5.10561700	4.70977700	-1.93796400
C	-6.21267200	2.53225400	-1.30477300
H	-4.37489900	2.74869000	-2.36964500
H	-4.17324800	5.21910100	-2.19883400
H	-5.76136100	4.76147900	-2.81580900
H	-5.58693400	5.28734400	-1.14256400
H	-6.07238100	1.47602000	-1.05489500
H	-6.78558300	2.99316200	-0.49254300
H	-6.83527400	2.58056900	-2.20650600
C	-1.98125000	3.83700300	-0.39374300
C	-1.92009700	5.33125500	-0.05025100
C	-1.47840500	3.57648900	-1.82004000
H	-1.29777300	3.31718400	0.29372000
H	-2.18551000	5.52442400	0.99387300
H	-0.90616000	5.71948000	-0.20695200
H	-2.59169500	5.92611200	-0.67872300
H	-1.45606600	2.50758900	-2.05490500
H	-2.11090200	4.06977200	-2.56662400
H	-0.46106900	3.96445500	-1.95213500
C	-4.33858300	3.46527700	1.64226700
C	-5.18254600	2.36040400	2.29350300
C	-5.10211000	4.79787900	1.67254600
H	-3.43421000	3.58834200	2.25718500
H	-4.63929200	1.41374700	2.35261900
H	-5.47386200	2.64789500	3.31117300
H	-6.10610000	2.17773200	1.73234000
H	-4.53374900	5.62431000	1.23534600
H	-6.04989100	4.72466800	1.12817800
H	-5.34683300	5.07697000	2.70458100
C	2.50278900	3.76752400	-1.01308100
C	2.73021700	3.39736200	-2.48460900
C	2.39072800	5.28761600	-0.83682900
H	1.53688500	3.33430600	-0.71403300
H	2.75707100	2.31299400	-2.63103800
H	1.92716000	3.79644600	-3.11615400
H	3.67281800	3.80920600	-2.86278600
H	2.12164700	5.56478200	0.18735200

H	3.32576800	5.80138100	-1.08462100
H	1.61662800	5.69502900	-1.49918500
C	5.53613200	2.99752300	-0.51062100
C	6.53186200	2.27285400	0.40544500
C	6.03990200	4.40868600	-0.84393400
H	5.49408200	2.43548300	-1.45486400
H	6.20415800	1.25597800	0.64527600
H	7.51831700	2.20178400	-0.06886800
H	6.66759300	2.80669100	1.35239400
H	5.39910900	4.91555600	-1.57133300
H	6.09788400	5.04391500	0.04574400
H	7.04825500	4.36356700	-1.27345700
C	3.49903500	3.48625200	1.93781000
C	4.23635700	4.79142200	2.27394700
C	3.82464900	2.41258200	2.98534500
H	2.41948900	3.68949900	2.00475600
H	4.01889600	5.59829500	1.56758500
H	3.95537200	5.14514400	3.27330700
H	5.32144100	4.64230100	2.28121500
H	3.26288000	1.49101900	2.81389000
H	4.88957800	2.15541500	2.97852200
H	3.58517100	2.77341400	3.99309100
S	-1.71023700	-4.43275500	-0.14204500
S	1.58912200	-4.47086800	-0.14532900

Triplet:

Sum of electronic and zero-point Energies = -3640.167930
Sum of electronic and thermal Energies = -3640.105187
Sum of electronic and thermal Enthalpies = -3640.104243
Sum of electronic and thermal Free Energies = -3640.271068

S	-0.00373100	-0.49486300	-0.01151900
C	-0.74384400	-2.97107600	-0.06052400
C	0.69274700	-2.98417500	-0.06245600
C	1.23869100	-1.72432100	-0.03428200
C	-1.26722200	-1.70210200	-0.03656700
C	-2.65912500	-1.35957700	-0.02250100
C	2.63551000	-1.40637700	-0.02560800
C	-3.34135300	-3.77214000	0.03353000
C	-3.66160600	-2.39962500	0.04519900
C	3.27781300	-3.83108000	-0.15315300
C	3.62265600	-2.46206600	-0.10686600
C	-4.36024400	-4.72362800	0.11015200

C	-5.02867000	-2.05036200	0.13672700
C	4.27903400	-4.79411600	-0.24224000
C	4.99311800	-2.14052800	-0.14992900
H	-4.10378800	-5.77911900	0.10191600
H	4.00493100	-5.84454100	-0.28000500
C	-6.04609700	-2.98226700	0.21422500
C	-5.68281700	-4.34089200	0.19808600
C	5.61579200	-4.43538100	-0.28482700
C	6.00094200	-3.08935500	-0.23830400
H	-5.25619500	-0.99262900	0.14906700
H	-6.44467200	-5.11092700	0.25729900
H	5.24713100	-1.08728600	-0.11378800
H	6.35823100	-5.22028600	-0.35638900
C	-7.52374400	-2.59337000	0.32285800
C	-8.09870100	-3.14916700	1.63822800
C	-7.72912600	-1.07462800	0.31302500
C	-8.29781900	-3.19098500	-0.86573400
H	-8.01900600	-4.23890400	1.69043300
H	-7.57066900	-2.73314100	2.50201100
H	-9.15862200	-2.88836900	1.72905200
H	-7.36706900	-0.62196500	-0.61539500
H	-8.79668100	-0.84829000	0.39596500
H	-7.22174900	-0.58805500	1.15206000
H	-9.35851500	-2.92594900	-0.79894600
H	-7.91116100	-2.80952300	-1.81600200
H	-8.22967200	-4.28238100	-0.89082900
C	7.46766900	-2.64382700	-0.27841500
C	7.80788600	-1.89330600	1.02133200
C	8.42884000	-3.83099300	-0.41332500
C	7.68911900	-1.70837900	-1.48017700
H	7.18178200	-1.00625300	1.15195500
H	7.66363200	-2.53682100	1.89498600
H	8.85314700	-1.56576200	1.00832800
H	8.25017300	-4.39712200	-1.33314900
H	9.46007100	-3.46619900	-0.44544400
H	8.35001100	-4.51909000	0.43447200
H	8.73500300	-1.38526500	-1.52059600
H	7.45326100	-2.21588700	-2.42090500
H	7.06619400	-0.81159300	-1.41853600
C	-3.02000900	-0.00471700	-0.06312700
C	3.01947400	-0.05990500	0.05602000
C	-3.29625500	1.18559000	-0.10819700
C	3.31417400	1.12396600	0.13859400

Si	-3.67237600	2.98873300	-0.20367900
Si	3.72620300	2.91782200	0.25877000
C	-4.85605800	3.19163900	-1.68372800
C	-5.08119600	4.63714900	-2.14810900
C	-6.19689500	2.47932100	-1.46323900
H	-4.33907700	2.66454000	-2.49829200
H	-4.14404100	5.13994200	-2.40451900
H	-5.71678500	4.65761800	-3.04197800
H	-5.58177100	5.23970000	-1.38380700
H	-6.05884900	1.43218400	-1.17583600
H	-6.78621100	2.96538600	-0.67786200
H	-6.80214800	2.49588900	-2.37786200
C	-1.98575300	3.82205200	-0.50578500
C	-1.93701000	5.32815800	-0.21662100
C	-1.44359900	3.51008100	-1.90705200
H	-1.31976200	3.33052700	0.21872100
H	-2.23042500	5.55905400	0.81223800
H	-0.92012400	5.71355500	-0.36091800
H	-2.59314900	5.89708900	-0.88420600
H	-1.41230400	2.43313000	-2.10053000
H	-2.05705200	3.97224000	-2.68870300
H	-0.42396000	3.89666500	-2.02651000
C	-4.38719400	3.51656500	1.48595800
C	-5.23977500	2.43196600	2.15959100
C	-5.15721100	4.84527000	1.45117200
H	-3.49672700	3.66674700	2.11496900
H	-4.69261200	1.49167500	2.26649600
H	-5.55557300	2.75569300	3.15897100
H	-6.14943200	2.22215500	1.58530200
H	-4.58252600	5.65872500	0.99813700
H	-6.09110700	4.74728300	0.88732400
H	-5.42774000	5.15965000	2.46654200
C	2.55649800	3.77547100	-0.97506900
C	2.89995900	3.42706100	-2.42920400
C	2.40379400	5.29112500	-0.79205100
H	1.57874900	3.32218800	-0.75444700
H	2.95982400	2.34522800	-2.58453900
H	2.13820800	3.81813000	-3.11472100
H	3.85955700	3.86171200	-2.73141300
H	2.05072600	5.55086200	0.21093000
H	3.34573400	5.82434100	-0.96045200
H	1.67606100	5.69300500	-1.50824200
C	5.55829600	3.04551600	-0.24924000

C	6.48899600	2.33150700	0.74066300
C	6.06257500	4.46676800	-0.53536900
H	5.60083900	2.48839300	-1.19636900
H	6.15921300	1.30836900	0.94971900
H	7.51159900	2.27908300	0.34728100
H	6.53873800	2.86262900	1.69753800
H	5.47344400	4.96592300	-1.31035900
H	6.03654300	5.09786000	0.35856300
H	7.10305500	4.44174800	-0.88201500
C	3.33625000	3.48824900	2.03808100
C	4.03216900	4.79897800	2.43460200
C	3.59324500	2.41185700	3.10172400
H	2.25266500	3.67998000	2.02514400
H	3.86319900	5.60592300	1.71522600
H	3.67035400	5.14710100	3.40971700
H	5.11470300	4.66022900	2.52673200
H	3.05768400	1.48483200	2.88215600
H	4.65850000	2.16692800	3.17540500
H	3.27252400	2.76314800	4.09013400
S	-1.70543500	-4.42521200	-0.09638100
S	1.62645300	-4.45637200	-0.08653200

8.3 Compound Th2-TIPS

Natural Orbital Coefficient
HOMO: 1.80024 LUMO: 0.19976
singlet biradical character y_0 = 0.024

Singlet Biradical Open Shell:

Sum of electronic and zero-point Energies = -4114.527387
Sum of electronic and thermal Energies = -4114.462141
Sum of electronic and thermal Enthalpies = -4114.461197
Sum of electronic and thermal Free Energies = -4114.633563

S	-1.71273000	1.22953200	-0.05067800
S	1.78245100	-1.14006200	0.00614600
C	-0.03206100	0.75108700	-0.03270600
C	1.15856800	1.43350500	-0.03751000
C	2.28916000	0.55803800	-0.01749500
C	0.10238500	-0.66053500	-0.00977100
C	-1.08773600	-1.34355700	-0.00577900
C	-2.21885900	-0.46899900	-0.02695700
C	-3.53987800	-0.84766900	-0.03028400

C	-2.99611100	-3.30784100	0.00997100
C	-3.93448000	-2.26216900	-0.01563900
S	-1.23894600	-3.08256300	0.02676900
C	-3.43343500	-4.62712300	0.02531500
C	-5.29142200	-2.60526400	-0.02709900
C	-4.78684900	-4.93082900	0.01569200
C	-5.74899500	-3.91992500	-0.01134300
H	-2.70376800	-5.43152700	0.04551200
H	-5.08045100	-5.97316900	0.02933900
H	-6.00517300	-1.78970900	-0.04945400
C	-4.53808000	0.16022500	-0.04740900
C	-7.25570500	-4.20103500	-0.02771200
C	-7.91596800	-3.53816400	1.19442800
C	-7.86885400	-3.62216800	-1.31533500
C	-7.56398500	-5.70232400	0.01787100
H	-7.77214900	-2.45380400	1.19780400
H	-8.99429200	-3.73057800	1.19315300
H	-7.50091200	-3.93341300	2.12700600
H	-7.42321100	-4.08309400	-2.20261700
H	-8.94770300	-3.81050100	-1.33979300
H	-7.71711500	-2.54147500	-1.38781700
H	-7.17202900	-6.17148800	0.92604500
H	-8.64756200	-5.85491900	0.00918200
H	-7.15053200	-6.23001600	-0.84755200
C	-5.37011700	1.04886100	-0.06166500
Si	-6.61686300	2.41173300	-0.06584500
C	-7.82501100	2.07505700	-1.50496800
C	-9.17079600	2.80375700	-1.37019800
C	-8.06172500	0.58313700	-1.77774300
H	-7.31208800	2.48887100	-2.38610500
H	-9.05796500	3.87899300	-1.20144900
H	-9.76979200	2.67515900	-2.27983500
H	-9.75877000	2.39907900	-0.53946600
H	-7.12597500	0.05054400	-1.96799000
H	-8.55665600	0.09419600	-0.93086200
H	-8.70995000	0.44900800	-2.65242500
C	-5.61816500	3.99613200	-0.40613900
C	-4.70548200	4.36516800	0.77089700
C	-6.45131100	5.20313400	-0.85720500
H	-4.96867100	3.71724700	-1.24857300
H	-4.06161200	3.53053400	1.06635600
H	-4.05470600	5.20827400	0.50963700
H	-5.28580200	4.66566800	1.65072200

H	-7.03689500	4.98621500	-1.75597900
H	-7.14749900	5.53557100	-0.07981300
H	-5.79946800	6.05398700	-1.08961300
C	-7.39230300	2.39082800	1.67435400
C	-8.14899000	1.08701900	1.96361900
C	-8.25883100	3.60967300	2.02033000
H	-6.51884900	2.41241200	2.34185200
H	-7.52772000	0.20507700	1.77607400
H	-8.47044600	1.04862900	3.01148000
H	-9.05042400	0.99945300	1.34696300
H	-7.70789100	4.54984900	1.92281500
H	-9.14495900	3.67751300	1.38169000
H	-8.61230600	3.54541900	3.05673300
C	3.61077800	0.93475500	-0.01591000
C	4.00702400	2.34813000	-0.03366500
C	3.07011600	3.39517000	-0.05726800
C	5.36437100	2.68873000	-0.02747600
C	3.50983900	4.71366500	-0.07495000
C	5.82438100	4.00235000	-0.04483800
C	4.86388100	5.01494400	-0.06929200
H	6.07602000	1.87122700	-0.00880600
H	5.15931300	6.05675900	-0.08418400
H	2.78163100	5.51942000	-0.09358600
C	7.33168600	4.28028400	-0.03170300
C	7.94485900	3.70388800	1.25699300
C	7.98906700	3.61163300	-1.25220600
C	7.64328300	5.78069400	-0.08201500
H	7.50077700	4.16742000	2.14365200
H	7.79041500	2.62365400	1.33194500
H	9.02420000	3.88922400	1.28008400
H	7.57431500	4.00525300	-2.18561300
H	9.06790900	3.80120400	-1.25227900
H	7.84199100	2.52785600	-1.25170100
H	8.72721300	5.93095400	-0.07611700
H	7.25032900	6.24804800	-0.99067600
H	7.23283600	6.31179500	0.78274200
C	4.60372600	-0.07861900	0.00229800
C	5.41679700	-0.98466200	0.01646100
S	1.31243000	3.17245900	-0.06789900
Si	6.61425500	-2.39324300	0.02942400
C	5.84250100	-3.70571000	-1.11174200
C	6.46317800	-5.10632600	-1.01922400
C	5.79705200	-3.22159700	-2.56776600

H	4.80309800	-3.78523700	-0.76275200
H	6.39980800	-5.51949200	-0.00826800
H	5.94107600	-5.80073100	-1.68878000
H	7.51822600	-5.11039900	-1.31236200
H	6.80312700	-3.12853600	-2.99252900
H	5.24563900	-3.93117800	-3.19616100
H	5.30555400	-2.24790000	-2.65795500
C	8.24587600	-1.67496600	-0.64035400
C	9.29819900	-2.71232700	-1.05528400
C	8.84678700	-0.64985500	0.33121100
H	7.94823400	-1.13081100	-1.54793200
H	8.92972200	-3.38221700	-1.83751200
H	10.19387200	-2.21465000	-1.44688400
H	9.61852300	-3.33393900	-0.21272900
H	8.12282600	0.12308900	0.61075900
H	9.19183800	-1.12648600	1.25555800
H	9.71287500	-0.14745400	-0.11633700
C	6.72899500	-2.94741300	1.84692900
C	7.89180500	-3.89392100	2.17563700
C	5.39494300	-3.52926900	2.33545900
H	6.90057700	-2.01438500	2.40235800
H	8.86469000	-3.44917000	1.94602700
H	7.89164600	-4.14318200	3.24382300
H	7.82283400	-4.83816500	1.62577900
H	4.56006400	-2.84489600	2.15460400
H	5.16223800	-4.47665200	1.83594700
H	5.43052400	-3.73270300	3.41244800

Singlet Closed Shell:

Sum of electronic and zero-point Energies = -4114.525095
Sum of electronic and thermal Energies = -4114.459917
Sum of electronic and thermal Enthalpies = -4114.458973
Sum of electronic and thermal Free Energies = -4114.631399

S	-1.71532300	1.23499300	-0.04989600
S	1.78523600	-1.14534800	0.00609100
C	-0.03069200	0.75828300	-0.03258100
C	1.14572400	1.43597200	-0.03783100
C	2.29110900	0.55654700	-0.01777000
C	0.10122100	-0.66752500	-0.00935000
C	-1.07469000	-1.34583400	-0.00478300
C	-2.22061400	-0.46734300	-0.02604400
C	-3.52845800	-0.84794100	-0.02930500

C	-2.98982900	-3.31030600	0.01154400
C	-3.92699400	-2.26536300	-0.01439200
S	-1.23219800	-3.08577300	0.02868300
C	-3.42800400	-4.62900800	0.02709600
C	-5.28315800	-2.60515000	-0.02604700
C	-4.78211300	-4.93114600	0.01731300
C	-5.74285500	-3.91999300	-0.01011600
H	-2.69922000	-5.43419300	0.04758900
H	-5.07663700	-5.97325600	0.03115700
H	-5.99597000	-1.78877600	-0.04866100
C	-4.53174300	0.16011000	-0.04688700
C	-7.24985800	-4.19896800	-0.02673600
C	-7.90960900	-3.53450100	1.19482000
C	-7.86189700	-3.62001200	-1.31485000
C	-7.56013200	-5.69980500	0.01962400
H	-7.76429300	-2.45032400	1.19767200
H	-8.98821400	-3.72535200	1.19330800
H	-7.49540400	-3.92982600	2.12774100
H	-7.41679500	-4.08223500	-2.20172600
H	-8.94104600	-3.80663500	-1.33940300
H	-7.70846500	-2.53959800	-1.38805300
H	-7.16897700	-6.16893100	0.92816300
H	-8.64390400	-5.85103000	0.01073100
H	-7.14709000	-6.22850200	-0.84538100
C	-5.36382000	1.04719000	-0.06134900
Si	-6.61075600	2.41063800	-0.06624500
C	-7.81797300	2.07351500	-1.50591700
C	-9.16390900	2.80206000	-1.37162200
C	-8.05434900	0.58155700	-1.77874300
H	-7.30477800	2.48742100	-2.38684200
H	-9.05127800	3.87731900	-1.20289000
H	-9.76254500	2.67333700	-2.28146800
H	-9.75211100	2.39733200	-0.54107900
H	-7.11846600	0.04909300	-1.96868600
H	-8.54950200	0.09253900	-0.93204700
H	-8.70223500	0.44733400	-2.65365200
C	-5.61157700	3.99467300	-0.40628000
C	-4.69941000	4.36382500	0.77114000
C	-6.44445800	5.20165900	-0.85792100
H	-4.96170700	3.71557600	-1.24834900
H	-4.05560000	3.52929400	1.06696600
H	-4.04855700	5.20689700	0.51000200
H	-5.28011600	4.66447800	1.65065800

H	-7.02961400	4.98471700	-1.75697000
H	-7.14098300	5.53424300	-0.08088900
H	-5.79241500	6.05240800	-1.09009400
C	-7.38618100	2.38910000	1.67381400
C	-8.14276000	1.08517300	1.96282300
C	-8.25289200	3.60785700	2.01968700
H	-6.51281400	2.41075700	2.34141900
H	-7.52134000	0.20331100	1.77542600
H	-8.46442600	1.04673000	3.01061000
H	-9.04404500	0.99750400	1.34597100
H	-7.70205100	4.54809400	1.92221800
H	-9.13897000	3.67556600	1.38096300
H	-8.60644300	3.54354300	3.05605000
C	3.59955600	0.93520900	-0.01629300
C	3.99974300	2.35149000	-0.03408500
C	3.06405900	3.39781300	-0.05788100
C	5.35631400	2.68874500	-0.02758200
C	3.50466400	4.71572700	-0.07555500
C	5.81847900	4.00252700	-0.04491800
C	4.85940400	5.01539600	-0.06963300
H	6.06699300	1.87040400	-0.00867100
H	5.15578800	6.05697600	-0.08452300
H	2.77736400	5.52228600	-0.09437400
C	7.32608200	4.27825000	-0.03132300
C	7.93805600	3.70104800	1.25759000
C	7.98297500	3.60863200	-1.25156800
C	7.63972100	5.77821400	-0.08158600
H	7.49459700	4.16552100	2.14406700
H	7.78173400	2.62108500	1.33275400
H	9.01772200	3.88447600	1.28086400
H	7.56910900	4.00282500	-2.18512500
H	9.06209500	3.79663000	-1.25127000
H	7.83438900	2.52504600	-1.25112200
H	8.72384700	5.92707000	-0.07547000
H	7.24751200	6.24604800	-0.99032300
H	7.22976000	6.30984300	0.78307800
C	4.59761500	-0.07829800	0.00195100
C	5.41061000	-0.98292900	0.01605300
S	1.30592000	3.17583700	-0.06887000
Si	6.60805200	-2.39223700	0.02870500
C	5.83572100	-3.70337000	-1.11341200
C	6.45585700	-5.10428700	-1.02166000
C	5.79069100	-3.21829300	-2.56913000

H	4.79625300	-3.78263000	-0.76459000
H	6.39237900	-5.51797700	-0.01092500
H	5.93341100	-5.79809200	-1.69155400
H	7.51088000	-5.10862800	-1.31489200
H	6.79685700	-3.12530100	-2.99369100
H	5.23912700	-3.92731500	-3.19800500
H	5.29943800	-2.24442900	-2.65879700
C	8.23987400	-1.67396700	-0.64028200
C	9.29203200	-2.71148600	-1.05525400
C	8.84073800	-0.64937600	0.33186400
H	7.94262500	-1.12942200	-1.54776200
H	8.92360300	-3.38103200	-1.83779900
H	10.18793300	-2.21390700	-1.44643500
H	9.61195100	-3.33343400	-0.21279100
H	8.11691700	0.12365400	0.61152500
H	9.18545100	-1.12644800	1.25610600
H	9.70703700	-0.14699900	-0.11528000
C	6.72124000	-2.94646800	1.84615500
C	7.88381600	-3.89314600	2.17527000
C	5.38684900	-3.52842400	2.33366300
H	6.89257200	-2.01358200	2.40190000
H	8.85687400	-3.44847700	1.94622800
H	7.88306000	-4.14257900	3.24340600
H	7.81497900	-4.83729100	1.62522200
H	4.55198500	-2.84417400	2.15229700
H	5.15461300	-4.47581200	1.83394900
H	5.42175300	-3.73191000	3.41065700

Triplet:

Sum of electronic and zero-point Energies = -4114.511586
Sum of electronic and thermal Energies = -4114.446305
Sum of electronic and thermal Enthalpies = -4114.445361
Sum of electronic and thermal Free Energies = -4114.618795

S	-1.70627800	1.21310400	-0.05419800
S	1.77565300	-1.12410300	0.00443500
C	-0.03804000	0.72874000	-0.03471100
C	1.20468300	1.42554000	-0.03868700
C	2.28413900	0.56205100	-0.01886200
C	0.10796900	-0.63879600	-0.01232200
C	-1.13422800	-1.33614400	-0.00979900
C	-2.21421100	-0.47343900	-0.03076800
C	-3.58665400	-0.84792300	-0.03452400

C	-3.01924100	-3.29825900	0.00308500
C	-3.96389700	-2.24986300	-0.02133600
S	-1.26545800	-3.07092400	0.01908300
C	-3.45308300	-4.62048100	0.01749300
C	-5.32435300	-2.60761500	-0.03224500
C	-4.80305900	-4.93096500	0.00821800
C	-5.77212300	-3.92119200	-0.01741100
H	-2.71949300	-5.42138000	0.03671300
H	-5.09248600	-5.97432900	0.02098000
H	-6.04227000	-1.79572300	-0.05349000
C	-4.56619300	0.15885700	-0.04932900
C	-7.27736000	-4.21234300	-0.03278800
C	-7.94010900	-3.55612600	1.19157700
C	-7.89552700	-3.63464400	-1.31851300
C	-7.57635500	-5.71568800	0.00998900
H	-7.80294200	-2.47096800	1.19679000
H	-9.01714700	-3.75552700	1.19127900
H	-7.52136500	-3.95057400	2.12285100
H	-7.44726800	-4.08998100	-2.20736900
H	-8.97293700	-3.83096400	-1.34256300
H	-7.75171600	-2.55272000	-1.38822600
H	-7.18120800	-6.18444300	0.91698800
H	-8.65899700	-5.87466100	0.00175200
H	-7.16053900	-6.23925500	-0.85681200
C	-5.39732100	1.05438600	-0.06214200
Si	-6.64146100	2.41648200	-0.06116500
C	-7.85679900	2.08318100	-1.49566700
C	-9.20109400	2.81355000	-1.35595000
C	-8.09656300	0.59173800	-1.76846200
H	-7.34647600	2.49660300	-2.37852100
H	-9.08620400	3.88842500	-1.18634400
H	-9.80320900	2.68682800	-2.26383400
H	-9.78712100	2.40885800	-0.52384900
H	-7.16209900	0.05809700	-1.96218700
H	-8.58906100	0.10303000	-0.92000200
H	-8.74810400	0.45880500	-2.64090300
C	-5.64338200	4.00110000	-0.40454100
C	-4.72560500	4.36835200	0.76899700
C	-6.47691000	5.20929000	-0.85153900
H	-4.99758300	3.72218000	-1.24981400
H	-4.08167500	3.53256100	1.06119200
H	-4.07488400	5.21104600	0.50608100
H	-5.30207700	4.66853100	1.65146400

H	-7.06632700	4.99335200	-1.74803600
H	-7.16969100	5.54203500	-0.07125700
H	-5.82519300	6.05967600	-1.08617800
C	-7.41369200	2.39876300	1.68116900
C	-8.17179100	1.09625200	1.97257800
C	-8.27736400	3.61898100	2.02931200
H	-6.53866300	2.41887200	2.34663600
H	-7.55248200	0.21333800	1.78302600
H	-8.49041600	1.05819800	3.02135100
H	-9.07510600	1.01025500	1.35843800
H	-7.72492400	4.55820400	1.93098600
H	-9.16464200	3.68869100	1.39249800
H	-8.62890500	3.55512500	3.06644200
C	3.65723100	0.93453700	-0.01701600
C	4.03610200	2.33540600	-0.03617600
C	3.09278500	3.38509600	-0.05935700
C	5.39696600	2.69090600	-0.03266800
C	3.52882900	4.70652900	-0.07877600
C	5.84696400	4.00354500	-0.05173900
C	4.87940000	5.01482900	-0.07540600
H	6.11294900	1.87720800	-0.01469200
H	5.17047200	6.05769500	-0.09152800
H	2.79655000	5.50864600	-0.09701500
C	7.35274800	4.29203400	-0.04159000
C	7.97215200	3.72002400	1.24604500
C	8.01198600	3.62748500	-1.26329200
C	7.65451700	5.79452300	-0.09286100
H	7.52588300	4.17979700	2.13357300
H	7.82614300	2.63864900	1.32071200
H	9.05001100	3.91389000	1.26750500
H	7.59258700	4.01785700	-2.19599300
H	9.08945000	3.82462300	-1.26568700
H	7.87221100	2.54280600	-1.26193700
H	8.73748200	5.95150200	-0.08914800
H	7.25706900	6.25928500	-1.00089400
H	7.24264400	6.32334200	0.77261900
C	4.63122600	-0.07793100	0.00202600
C	5.44315200	-0.99085700	0.01751100
S	1.33840000	3.16036300	-0.06641400
Si	6.63860200	-2.39808000	0.03501800
C	5.86984300	-3.71548300	-1.10329200
C	6.49087700	-5.11556300	-1.00574900
C	5.82631900	-3.23593600	-2.56086400

H	4.82989900	-3.79461700	-0.75573200
H	6.42526900	-5.52614800	0.00611100
H	5.97082400	-5.81205600	-1.67479500
H	7.54666300	-5.11987100	-1.29622200
H	6.83303400	-3.14387900	-2.98435700
H	5.27607400	-3.94749700	-3.18811900
H	5.33502900	-2.26245800	-2.65462400
C	8.27183400	-1.68237100	-0.63478300
C	9.32450000	-2.72072700	-1.04621200
C	8.87190200	-0.65502800	0.33492900
H	7.97528900	-1.14025200	-1.54390600
H	8.95668500	-3.39243100	-1.82720300
H	10.22064800	-2.22411100	-1.43816500
H	9.64397700	-3.34045000	-0.20195200
H	8.14766000	0.11872900	0.61153500
H	9.21548900	-1.12942900	1.26098800
H	9.73886100	-0.15406500	-0.11264100
C	6.75489900	-2.95124700	1.85333700
C	7.91739700	-3.89751800	2.18358100
C	5.42068200	-3.53171700	2.34298600
H	6.92671400	-2.01750600	2.40747900
H	8.89035200	-3.45298800	1.95384200
H	7.91694500	-4.14562700	3.25208000
H	7.84876700	-4.84235700	1.63472900
H	4.58651400	-2.84655000	2.16174500
H	5.18684700	-4.47925400	1.84425700
H	5.45628200	-3.73436400	3.42015900

8.4 Compound Th3-TIPS

Natural Orbital Coefficient
HOMO: 1.51981 LUMO: 0.48019
singlet biradical character y_0 = 0.182

Singlet Biradical Open Shell:

Sum of electronic and zero-point Energies = -4588.862593
Sum of electronic and thermal Energies = -4588.794885
Sum of electronic and thermal Enthalpies = -4588.793941
Sum of electronic and thermal Free Energies = -4588.971381

S	-1.93840700	-0.17705800	-0.06138000
S	1.94961400	-0.16603100	-0.03954600
S	0.01615600	-3.92688900	-0.03347500

C	-0.68544100	-1.38648900	-0.05152800
C	0.70347600	-1.38255800	-0.04420600
C	3.20269900	-1.41317100	-0.02209100
C	2.63862700	-2.70887900	-0.02133700
C	1.24843600	-2.67907800	-0.03321800
C	-1.22319400	-2.68612200	-0.04638300
C	-2.61314000	-2.72427800	-0.04923300
C	-3.18465300	-1.43152500	-0.05574800
C	-4.54125700	-1.13249900	-0.05384900
C	-5.21551700	-3.55853600	-0.03341300
C	-5.55049700	-2.19216400	-0.04124600
S	-3.55992200	-4.18825900	-0.04519500
C	-6.22256700	-4.51686500	-0.01640600
C	-6.91166300	-1.85741600	-0.03287500
C	-7.55919800	-4.14819100	-0.00696800
C	-7.93269500	-2.80201900	-0.01517700
C	4.55809400	-1.10637500	-0.00741300
C	5.57189400	-2.16033000	0.01627200
C	5.24507000	-3.52488600	0.01956100
S	3.59499300	-4.16665700	-0.00859900
C	6.25965200	-4.48109000	0.04542200
C	6.93521700	-1.81257800	0.03955200
C	7.95631900	-2.74938900	0.06493200
C	7.58799200	-4.10241300	0.06757700
H	-5.95715900	-5.57017100	-0.00949600
H	-8.30864900	-4.92978900	0.00739400
H	-7.15695200	-0.80146700	-0.03996900
H	7.16755600	-0.75583100	0.03764200
H	8.34844700	-4.87603900	0.08755600
H	5.99919700	-5.53562200	0.04846500
C	-4.93864100	0.22463100	-0.05531700
C	4.94492000	0.25398900	-0.00896000
C	-9.39536400	-2.34302700	-0.00731900
C	9.43838700	-2.36209000	0.09198700
C	-9.65252100	-1.46693600	1.23169200
C	-9.68121000	-1.52470700	-1.27911900
C	-10.37091800	-3.52501800	0.03236500
C	9.64421100	-0.84329300	0.07380800
C	10.14401900	-2.95798800	-1.13936100
C	10.08626600	-2.92066500	1.37171500
H	11.20760500	-2.69632900	-1.13049800
H	10.07098300	-4.04911600	-1.16380200
H	9.70652300	-2.57259500	-2.06570100

H	10.71502100	-0.61809500	0.09477200
H	9.22884000	-0.38697700	-0.83044400
H	9.18759800	-0.35879700	0.94256200
H	11.15000200	-2.66137700	1.40291600
H	9.60855700	-2.50585300	2.26489200
H	10.00812200	-4.01040800	1.42617600
H	-9.01783100	-0.57608700	1.24072600
H	-10.69541400	-1.13225800	1.24916300
H	-9.45900200	-2.02475500	2.15342600
H	-9.51395400	-2.12683500	-2.17788900
H	-10.72293200	-1.18576800	-1.28594400
H	-9.04225900	-0.63963700	-1.34563000
H	-10.23387000	-4.13562900	0.93054000
H	-11.39952000	-3.15175700	0.04072200
H	-10.26257400	-4.17237400	-0.84369700
C	-5.24746200	1.40367600	-0.05472000
C	5.23712900	1.43728600	-0.00700100
Si	-5.69572500	3.19455700	-0.02102300
Si	5.64495900	3.23856600	0.00264800
C	-6.95056800	3.49871900	-1.42680600
C	-7.77944800	4.77994000	-1.25131200
C	-7.87667600	2.30538100	-1.69960100
H	-6.32178900	3.63169700	-2.31996400
H	-7.16082400	5.66507300	-1.07505000
H	-8.38284500	4.97349900	-2.14645600
H	-8.47473900	4.69001900	-0.40981000
H	-7.31228400	1.39279700	-1.91004400
H	-8.53137400	2.10176800	-0.84450300
H	-8.52415800	2.50831300	-2.56141400
C	-4.07098400	4.11986700	-0.38156800
C	-3.06981300	3.99596400	0.77466300
C	-4.23786900	5.58343600	-0.81150300
H	-3.64826700	3.57639500	-1.23906700
H	-2.89408400	2.95187400	1.05310900
H	-2.10198000	4.43249500	0.50012200
H	-3.42007000	4.52415700	1.66872500
H	-4.86893300	5.68260500	-1.70034600
H	-4.68099000	6.19723400	-0.01986000
H	-3.26361200	6.02555700	-1.05293200
C	-6.34565300	3.51776100	1.74042900
C	-7.62185400	2.72368200	2.05154600
C	-6.52152800	4.99651400	2.11244800
H	-5.54947900	3.11349100	2.38200700

H	-7.49665300	1.65542200	1.84661900
H	-7.89646800	2.82788700	3.10817900
H	-8.47301400	3.08093600	1.46109300
H	-5.59568600	5.56643200	1.98982600
H	-7.29226400	5.48291400	1.50665200
H	-6.82708300	5.09342700	3.16146900
C	3.95903000	4.11891100	0.09698900
C	3.15508000	3.95572400	-1.19985500
C	4.01423200	5.58993900	0.53004700
H	3.42317300	3.56914800	0.88436500
H	3.05812500	2.90477700	-1.49081200
H	2.14281600	4.36157600	-1.08405500
H	3.62330000	4.49044500	-2.03409100
H	4.49033000	5.71369400	1.50766600
H	4.56307700	6.20974900	-0.18697300
H	3.00205700	6.00572000	0.60509600
C	6.55146600	3.56762600	-1.64079700
C	7.88146500	2.80794500	-1.73706800
C	6.74548800	5.04809000	-1.99668200
H	5.87712500	3.13684200	-2.39480000
H	7.75673700	1.74003300	-1.53054100
H	8.31258900	2.90374900	-2.74107300
H	8.62097500	3.20061300	-1.03032600
H	5.79764900	5.59349900	-2.02761200
H	7.39820900	5.55863400	-1.28178900
H	7.21085200	5.14596900	-2.98513800
C	6.66355100	3.58085000	1.58037900
C	7.45999500	4.89353400	1.53405800
C	7.58438000	2.42125500	1.98505200
H	5.90330900	3.68250500	2.36948500
H	6.84159700	5.75763500	1.27314400
H	7.91853600	5.09976200	2.50874200
H	8.27334200	4.83693800	0.80295100
H	7.03194100	1.48552300	2.10516600
H	8.36712000	2.25073600	1.23720400
H	8.08659300	2.64174400	2.93502200

Singlet Closed Shell:

Sum of electronic and zero-point Energies = -4588.856691

Sum of electronic and thermal Energies = -4588.789047

Sum of electronic and thermal Enthalpies = -4588.788103

Sum of electronic and thermal Free Energies = -4588.965702

S	-1.92984000	-0.16435200	-0.06649200
S	1.94048100	-0.15320300	-0.04434100
S	0.01601700	-3.94636700	-0.03783400
C	-0.66863000	-1.37655200	-0.05576200
C	0.68632800	-1.37276800	-0.04836000
C	3.19769700	-1.40618500	-0.02544900
C	2.61280300	-2.72522600	-0.02503100
C	1.25445600	-2.69329000	-0.03669200
C	-1.22937100	-2.70028600	-0.05024900
C	-2.58755200	-2.74053200	-0.05330300
C	-3.17993700	-1.42480400	-0.05959800
C	-4.51301600	-1.14015200	-0.05567200
C	-5.20435300	-3.56349800	-0.03481700
C	-5.53491100	-2.19907200	-0.04134700
S	-3.54976000	-4.19801900	-0.05251800
C	-6.21414400	-4.51793500	-0.01551900
C	-6.89051700	-1.85613100	-0.02952700
C	-7.54977800	-4.14291400	-0.00272700
C	-7.91760500	-2.79695300	-0.00962600
C	4.52934700	-1.11406800	-0.00928700
C	5.55625900	-2.16730600	0.01554200
C	5.23380600	-3.52962400	0.01769800
S	3.58478900	-4.17607800	-0.01459100
C	6.25109500	-4.48192300	0.04493800
C	6.91381300	-1.81126200	0.04122200
C	7.94110000	-2.74424100	0.06795000
C	7.57834400	-4.09680500	0.06942800
H	-5.95325000	-5.57235100	-0.00946100
H	-8.30228500	-4.92164500	0.01329000
H	-7.13115600	-0.79911300	-0.03559200
H	7.14139100	-0.75347200	0.04016800
H	8.34175100	-4.86760900	0.09036400
H	5.99525100	-5.53755500	0.04721700
C	-4.91699500	0.22311400	-0.05651000
C	4.92284600	0.25248400	-0.00981100
C	-9.37767300	-2.33055600	0.00180800
C	9.42123900	-2.34936400	0.09768700
C	-9.62757500	-1.45304500	1.24129100
C	-9.66261800	-1.51097000	-1.26939400
C	-10.35888700	-3.50770600	0.04403900
C	9.61900200	-0.82948100	0.07982400
C	10.13249300	-2.94139400	-1.13226300
C	10.06992400	-2.90432500	1.37855500

H	11.19463500	-2.67390700	-1.12159300
H	10.06545000	-4.03291300	-1.15654600
H	9.69451000	-2.55867900	-2.05948400
H	10.68855000	-0.59852500	0.10268400
H	9.20285500	-0.37539500	-0.82521300
H	9.15829900	-0.34737200	0.94775700
H	11.13219900	-2.63928400	1.41182400
H	9.58825600	-2.49229700	2.27089700
H	9.99753000	-3.99448800	1.43268900
H	-8.98835200	-0.56538100	1.24894600
H	-10.66866800	-1.11292400	1.26107600
H	-9.43486800	-2.01181200	2.16261500
H	-9.50091300	-2.11419500	-2.16843800
H	-10.70252300	-1.16642200	-1.27357800
H	-9.01917500	-0.62928400	-1.33785500
H	-10.22240600	-4.11892000	0.94189000
H	-11.38567900	-3.12952900	0.05498500
H	-10.25586500	-4.15564900	-0.83223700
C	-5.22529500	1.39985900	-0.05500900
C	5.21465500	1.43343000	-0.00696800
Si	-5.67043100	3.19273000	-0.02200400
Si	5.61949100	3.23671000	0.00343000
C	-6.92495700	3.49738800	-1.42765700
C	-7.75193500	4.77987500	-1.25218600
C	-7.85278100	2.30522800	-1.69970300
H	-6.29624000	3.62926200	-2.32100600
H	-7.13196500	5.66416500	-1.07640100
H	-8.35530800	4.97400900	-2.14720000
H	-8.44708900	4.69116800	-0.41044600
H	-7.28979200	1.39182300	-1.91029500
H	-8.50736500	2.10269200	-0.84428000
H	-8.50035200	2.50889600	-2.56125200
C	-4.04399400	4.11421300	-0.38354900
C	-3.04272400	3.98922300	0.77252300
C	-4.20846000	5.57783000	-0.81434800
H	-3.62254200	3.56936800	-1.24078700
H	-2.86852000	2.94505800	1.05153700
H	-2.07429700	4.42395500	0.49727100
H	-3.39175300	4.51868000	1.66631800
H	-4.83967000	5.67766900	-1.70301800
H	-4.65008600	6.19292800	-0.02286600
H	-3.23347500	6.01794900	-1.05641400
C	-6.31861400	3.51557200	1.73984500

C	-7.59569200	2.72318800	2.05163200
C	-6.49223800	4.99461100	2.11189100
H	-5.52250400	3.11024100	2.38082500
H	-7.47193300	1.65470700	1.84702200
H	-7.86967500	2.82804300	3.10834500
H	-8.44664000	3.08134700	1.46144100
H	-5.56577700	5.56334100	1.98841900
H	-7.26291500	5.48190600	1.50673500
H	-6.79674000	5.09193200	3.16115800
C	3.93192000	4.11353500	0.09621800
C	3.12962800	3.94974300	-1.20160900
C	3.98455000	5.58445000	0.53006600
H	3.39611400	3.56249200	0.88272000
H	3.03407800	2.89882100	-1.49302800
H	2.11677000	4.35426100	-1.08653900
H	3.59796100	4.48547300	-2.03513100
H	4.45942900	5.70847700	1.50824200
H	4.53313900	6.20544900	-0.18613000
H	2.97165800	5.99858000	0.60427600
C	6.52646000	3.56537200	-1.63951100
C	7.85744200	2.80732900	-1.73498200
C	6.71874500	5.04609800	-1.99539000
H	5.85292400	3.13370900	-2.39373100
H	7.73396000	1.73923300	-1.52873800
H	8.28895900	2.90382200	-2.73872800
H	8.59606300	3.20082000	-1.02778000
H	5.77027000	5.59038300	-2.02651900
H	7.37076400	5.55738300	-1.28038500
H	7.18416800	5.14443800	-2.98374600
C	6.63567200	3.57876900	1.58246700
C	7.43035400	4.89260300	1.53725300
C	7.55772700	2.42026800	1.98745500
H	5.87453500	3.67914100	2.37084700
H	6.81095000	5.75599800	1.27638600
H	7.88791200	5.09891800	2.51235600
H	8.24426100	4.83743400	0.80667100
H	7.00654700	1.48370200	2.10684600
H	8.34135700	2.25115200	1.24025100
H	8.05874200	2.64115800	2.93793700

Triplet:

Sum of electronic and zero-point Energies = -4588.852790

Sum of electronic and thermal Energies = -4588.785059

Sum of electronic and thermal Enthalpies = -4588.784114
Sum of electronic and thermal Free Energies = -4588.963190

S	1.94288300	-0.19020400	0.01593400
S	-1.96200400	-0.17720700	-0.02891300
S	-0.02194600	-3.91411600	-0.02187500
C	0.69640800	-1.39676100	-0.00450000
C	-0.72337100	-1.39194500	-0.02083700
C	-3.21200800	-1.41904200	-0.04480200
C	-2.66873700	-2.69426000	-0.04589200
C	-1.24841100	-2.66941600	-0.03160600
C	1.21297400	-2.67783900	-0.00276400
C	2.63288100	-2.71278400	0.01472400
C	3.18471500	-1.44071000	0.02591900
C	4.56670700	-1.12815000	0.04221600
C	5.22139500	-3.55711100	0.04176500
C	5.56159900	-2.18828000	0.05119300
S	3.56598200	-4.18198100	0.02012000
C	6.22581700	-4.51975200	0.04885100
C	6.92908100	-1.86266100	0.06875200
C	7.56318600	-4.15805300	0.06497200
C	7.94311300	-2.81135100	0.07549700
C	-4.59293500	-1.09680900	-0.05140200
C	-5.59323500	-2.15004300	-0.05396800
C	-5.26225900	-3.51777500	-0.05883100
S	-3.61275900	-4.15643400	-0.06369800
C	-6.27507700	-4.47760000	-0.06082300
C	-6.96322000	-1.81045400	-0.04904700
C	-7.97792900	-2.75096300	-0.04903000
C	-7.60415300	-4.10503500	-0.05629200
H	5.95562100	-5.57185700	0.04108600
H	8.30930300	-4.94283300	0.06924200
H	7.17926800	-0.80787700	0.07696800
H	-7.19999000	-0.75461400	-0.04409100
H	-8.36212100	-4.88120000	-0.05764900
H	-6.01043900	-5.53111500	-0.06511000
C	4.95748900	0.22267200	0.04435000
C	-4.97102500	0.25785000	-0.04834700
C	9.40860300	-2.36099500	0.09685400
C	-9.46231000	-2.37246400	-0.04129400
C	9.69669600	-1.48990500	-1.13887000
C	9.67239500	-1.54081000	1.37223100
C	10.37800600	-3.54864200	0.08116600

C	-9.67691400	-0.85499200	-0.02522200
C	-10.13281600	-2.96685900	1.21034300
C	-10.13849600	-2.94122700	-1.30175600
H	-11.19803700	-2.71248600	1.22640900
H	-10.05164400	-4.05731200	1.23887400
H	-9.67524400	-2.57326400	2.12344100
H	-10.74928200	-0.63642900	-0.01810800
H	-9.23950700	-0.39108800	0.86472200
H	-9.24715300	-0.37273700	-0.90858300
H	-11.20411400	-2.68794900	-1.30754200
H	-9.68569200	-2.52810900	-2.20857900
H	-10.05578200	-4.03076800	-1.35307700
H	9.06700400	-0.59590100	-1.16375200
H	10.74152300	-1.16090300	-1.13503400
H	9.51987500	-2.04935400	-2.06298000
H	9.48198300	-2.13916900	2.26891700
H	10.71597400	-1.20878500	1.40043400
H	9.03808100	-0.65136700	1.42258000
H	10.25673400	-4.16127100	-0.81790400
H	11.40859600	-3.18107700	0.09357600
H	10.24744600	-4.19255800	0.95670900
C	5.26574200	1.40448100	0.04423600
C	-5.25924800	1.44475400	-0.04546800
Si	5.71504500	3.19371500	0.00784300
Si	-5.66410700	3.24475500	-0.00857200
C	6.96320400	3.50248500	1.41888000
C	7.79435900	4.78217200	1.24315700
C	7.88666500	2.30940800	1.70164600
H	6.32978700	3.63949900	2.30815800
H	7.17758200	5.66719400	1.06002500
H	8.39296400	4.97863400	2.14090900
H	8.49424600	4.68841600	0.40588800
H	7.32002400	1.39833800	1.91272500
H	8.54594000	2.10152400	0.85107200
H	8.52957800	2.51519700	2.56623300
C	4.08957900	4.12206800	0.35810500
C	3.09332500	3.99541000	-0.80204000
C	4.25574700	5.58684900	0.78413500
H	3.66253400	3.58178200	1.21548200
H	2.91814500	2.95055200	-1.07804200
H	2.12455800	4.43345500	-0.53315700
H	3.44793600	4.52047200	-1.69623700
H	4.88263300	5.68827200	1.67568600

H	4.70330600	6.19765700	-0.00730600
H	3.28076500	6.03069800	1.01945400
C	6.37440500	3.51386200	-1.75099500
C	7.65180900	2.71835900	-2.05354300
C	6.55286000	4.99154300	-2.12585900
H	5.58139300	3.10835100	-2.39570400
H	7.52529700	1.65085100	-1.84553200
H	7.93152300	2.81896300	-3.10920900
H	8.50030300	3.07735700	-1.46027300
H	5.62655700	5.56203900	-2.00965000
H	7.32049100	5.47930300	-1.51723100
H	6.86405500	5.08568000	-3.17349800
C	-4.01052900	4.13025300	-0.33802500
C	-3.03138800	3.97549000	0.83328500
C	-4.13184000	5.59982800	-0.76303000
H	-3.58806800	3.58017700	-1.19143400
H	-2.88704300	2.92601500	1.10948900
H	-2.04838400	4.38835100	0.57637200
H	-3.38247900	4.50843500	1.72419300
H	-4.74345900	5.71993600	-1.66283400
H	-4.57342300	6.22103800	0.02370400
H	-3.14207400	6.01764000	-0.98397400
C	-6.33487700	3.57785500	1.74334800
C	-7.63807700	2.81811000	2.02696200
C	-6.47597200	5.05883500	2.12117300
H	-5.56140000	3.14796400	2.39599500
H	-7.54079500	1.74886400	1.81259500
H	-7.92646900	2.92049000	3.08015800
H	-8.46891200	3.20538700	1.42655200
H	-5.53275000	5.60353500	2.01763000
H	-7.22250800	5.56976800	1.50530800
H	-6.79658700	5.15863500	3.16546300
C	-6.88558900	3.59261000	-1.43334500
C	-7.68050600	4.89605100	-1.26416600
C	-7.84105600	2.42908500	-1.73213300
H	-6.23695300	3.71312000	-2.31406100
H	-7.04046200	5.76177900	-1.06929200
H	-8.26079800	5.11314500	-2.16917300
H	-8.39429800	4.82022600	-0.43683400
H	-7.30062300	1.50000900	-1.93311000
H	-8.52108300	2.24217100	-0.89340000
H	-8.46178300	2.65553300	-2.60779100
